# Structure of a monomeric photosystem II core complex from a cyanobacterium acclimated to far-red light reveals the functions of chlorophylls *d* and *f*

**DOI:** 10.1016/j.jbc.2021.101424

**Published:** 2021-11-19

**Authors:** Christopher J. Gisriel, Gaozhong Shen, Ming-Yang Ho, Vasily Kurashov, David A. Flesher, Jimin Wang, William H. Armstrong, John H. Golbeck, Marilyn R. Gunner, David J. Vinyard, Richard J. Debus, Gary W. Brudvig, Donald A. Bryant

**Affiliations:** 1 Department of Chemistry, Yale University, New Haven, Connecticut, USA; 2 Department of Biochemistry and Molecular Biology, The Pennsylvania State University, University Park, Pennsylvania, USA; 3 Intercollege Graduate Program in Plant Biology, The Pennsylvania State University, University Park, Pennsylvania, USA; 4 Department of Life Science, National Taiwan University, Taipei, Taiwan; 5 Department of Molecular Biophysics and Biochemistry, Yale University, New Haven, Connecticut, USA; 6 Department of Chemistry, Boston College, Chestnut Hill, Massachusetts, USA; 7 Department of Chemistry, The Pennsylvania State University, University Park, Pennsylvania, USA; 8 Department of Physics, City College of New York, New York, New York, USA; 9 Department of Biological Sciences, Louisiana State University, Baton Rouge, Louisiana, USA; 10 Department of Biochemistry, University of California, Riverside, California, USA

**Keywords:** photosystem II, far-red light photoacclimation, energy transfer, electron transfer, chlorophyll *f*, chlorophyll *d*, bicarbonate, photosynthesis, cyanobacteria, cryo-EM, Chl, chlorophyll, CTF, contrast transfer function, β-DM, *n*-dodecyl-β-d-maltoside, ESP, electrostatic potential, ETC, electron transfer chain, FaRLiP, far-red light photoacclimation, FRL, far-red light, FRL-AP, FRL-specific allophycocyanin, FRL-BC, far-red light bicylindrical core antenna complex(es), FRL-PBS, far-red light phycobilisom(es), FRL-PSII, far-red light–acclimated photosystem II, NH-Fe, non-heme Fe(II), OEC, oxygen-evolving complex, PBS, phycobilisome, PDB, Protein Data Bank, PIB, photosystem isolation buffer, PSI, photosystem I, PSII, photosystem II, WL, white light, XRD, X-ray diffraction

## Abstract

Far-red light (FRL) photoacclimation in cyanobacteria provides a selective growth advantage for some terrestrial cyanobacteria by expanding the range of photosynthetically active radiation to include far-red/near-infrared light (700–800 nm). During this photoacclimation process, photosystem II (PSII), the water:plastoquinone photooxidoreductase involved in oxygenic photosynthesis, is modified. The resulting FRL-PSII is comprised of FRL-specific core subunits and binds chlorophyll (Chl) *d* and Chl *f* molecules in place of several of the Chl *a* molecules found when cells are grown in visible light. These new Chls effectively lower the energy canonically thought to define the “red limit” for light required to drive photochemical catalysis of water oxidation. Changes to the architecture of FRL-PSII were previously unknown, and the positions of Chl *d* and Chl *f* molecules had only been proposed from indirect evidence. Here, we describe the 2.25 Å resolution cryo-EM structure of a monomeric FRL-PSII core complex from *Synechococcus* sp. PCC 7335 cells that were acclimated to FRL. We identify one Chl *d* molecule in the Chl_D1_ position of the electron transfer chain and four Chl *f* molecules in the core antenna. We also make observations that enhance our understanding of PSII biogenesis, especially on the acceptor side of the complex where a bicarbonate molecule is replaced by a glutamate side chain in the absence of the assembly factor Psb28. In conclusion, these results provide a structural basis for the lower energy limit required to drive water oxidation, which is the gateway for most solar energy utilization on earth.

Photosystem II (PSII) is a water:plastoquinone photooxidoreductase that, together with photosystem I (PSI), serves as the major entry point for solar energy into the biosphere (1). The mature PSII complex is a homodimeric membrane protein complex. Each ∼350 kDa monomer is comprised of ∼20 subunits that bind cofactors that perform energy and electron transfer (2). Light absorption by antenna pigments, including chlorophyll (Chl) molecules, initiates excitation energy transfer that ultimately results in charge separation in the reaction center core. The electron transfer chain (ETC) of PSII consists of a series of cofactors that spans the thylakoid membrane. On the lumenal side, water is oxidized at a Mn_4_CaO_5_ cluster called the oxygen-evolving complex (OEC) (3, 4). Plastoquinone is reduced on the stromal side of the intermembrane region.

When grown in far-red light (FRL, 700–800 nm), which is enriched in shaded environments, some terrestrial cyanobacteria alter PSII as part of a photoacclimation mechanism that extends their absorption into the far-red region of the solar spectrum, a phenomenon known as FRL photoacclimation (FaRLiP) (5, 6, 7). Specifically, the PSII subunits D1 (PsbA), D2 (PsbD), CP47 (PsbB), CP43 (PsbC), and the 10 kDa phosphoprotein (PsbH) are replaced with FRL-specific paralogs; in addition, one Chl *d* and approximately four Chl *f* molecules replace five of the 35 Chl *a* molecules that are found exclusively in white light (WL) PSII (5, 8, 9). Similar changes are observed in PSI, the other photochemical oxidoreductase involved in oxygenic photosynthesis (8, 9, 10, 11, 12, 13, 14). FRL-specific differences are also observed in the proteins that comprise the cores of phycobilisomes (PBSs), the main light-harvesting proteins of these cyanobacteria. FRL-specific allophycocyanins (FRL-APs) are produced during FaRLiP, and these form bicylindrical core antenna complexes (FRL-BCs) or FRL-PBSs, depending on the organism (5, 8, 9, 15, 16).

It has been suggested that, during FaRLiP, both PSI and PSII incorporate Chl *f* or Chl *d* in their ETC (17, 18, 19, 20). Because PSI and PSII canonically use Chl *a* (21), this could be significant because it would effectively lower the energy requirement to convert light energy into chemical energy in oxygenic photosynthesis and that would suggest that improved light wavelength utilization could be engineered into crops. Although the ETC of FRL-acclimated PSI (FRL-PSI) has been shown to be comprised of Chl *a* molecules only (10, 11, 13, 22), there is better spectroscopic evidence for the presence of Chl *f* or Chl *d* in the ETC of FRL-acclimated PSII (FRL-PSII) (17, 18, 21). Therefore, understanding the molecular basis of FaRLiP in PSII is of major interest in improving crop yields in shaded environments (23, 24). Molecular structures of FRL-PSI have been reported (10, 11, 13, 14), allowing for the identification of several Chl *f* molecules in the PSI antenna; however, the Chl *d-*binding and Chl *f*-binding sites in FRL-PSII have remained uncertain because no structural information has yet been reported.

Another important but poorly understood aspect of PSII is its biogenesis, which involves complicated mechanisms of assembly and repair (25), including the light-driven formation of the OEC, a process referred to as photoactivation (26). During PSII biogenesis, various assembly factors, such as Psb27 and Psb28, have been found to be associated with intermediate states of the immature complex (27). Recent cryo-EM studies have elucidated monomeric and dimeric PSII structures that are thought to exhibit characteristics similar to those involved in PSII biogenesis *in vivo* (27, 28, 29, 30, 31). On the donor side, the OEC-binding site of these structures lacks the OEC itself, with some of them instead exhibiting electrostatic potential (ESP) corresponding to a single cation (27, 28, 30), which was proposed to be the high-affinity Mn-binding site involved in the first step of photoactivation (26, 29, 32, 33, 34, 35, 36, 37, 38, 39, 40, 41). On the acceptor side, mature PSII features a tightly bound plastoquinone called Q_A_ and a mobile plastoquinone called Q_B_. Double reduction and protonation of Q_B_ initiates its diffusion from its binding pocket and its replacement by a new plastoquinone (42, 43). Between the Q_A-_ and Q_B_-binding sites, a hexacoordinate non-heme Fe(II) (NH-Fe) is found (44). A bicarbonate anion, which is involved in redox tuning of Q_A_ (45) and contributes to the coordination of the NH-Fe between Q_A_ and Q_B_ (44), has been the subject of extensive investigation (45, 46). Recent work showed that the assembly factor Psb28 binds to the stromal side of PSII during assembly and blocks the Q_B_-binding site (27, 31). The bicarbonate bound to the NH-Fe in mature PSII structures was absent in the structures with Psb28 bound, the bicarbonate being replaced by the carboxylate side chain of a D2 (PsbD) residue. Based on these observations, it was suggested that binding of Psb28 causes the displacement of bicarbonate during PSII biogenesis, which increases the redox potential of Q_A_^−⋅^ formation to favor safe charge recombination between P_680_^+⋅^ and Q_A_^−⋅^ (27, 31). However, bound Psb28 does not directly interact with the bicarbonate-binding site, and anoxygenic quinone-type photochemical oxidoreductases do not require an analogous subunit for maintaining a carboxylate side chain ligand to the NH-Fe, so more information is needed to understand whether Psb28 binding causes bicarbonate to be replaced by the nearby carboxylate side chain.

To provide insight into the characteristics of FRL-PSII, we employed cryo-EM to solve the structure of a PSII core complex from *Synechococcus* sp. PCC 7335 (hereafter, *Synechococcus* 7335) cells that were acclimated to FRL. The 2.25 Å global resolution structure reveals that this complex is monomeric and lacks all extrinsic subunits, some peripheral integral membrane subunits, and the OEC; however, it retains nearly all the cofactors associated with the core subunits. Among these cofactors, one Chl *d* and four Chl *f* molecules were identified. For convenience, we will hereafter refer to this complex as apo-FRL-PSII. The study reveals that a Chl *d* molecule is present in the active branch of the ETC, providing a structural basis for the red limit in oxygenic photosynthesis. In addition, the binding sites for four Chl *f* molecules that are involved in energy transfer to the ETC were identified. The structure exhibits important characteristics on both the donor and acceptor sides of the ETC that provide insights into PSII biogenesis, including a carboxylate side-chain ligand to the NH-Fe in the absence of the Psb28 assembly factor and a cation adjacent to the proposed high-affinity Mn-binding site.

## Results

### Preparation of the core complex

A monomeric apo-FRL-PSII core complex was isolated by immobilized metal affinity chromatography and sucrose density gradient centrifugation as described in the Experimental procedures section. The appearance of a typical sucrose gradient, absorbance and fluorescence spectra, and representative results for SDS-PAGE of apo-FRL-PSII are shown in Figure S1. Sucrose-gradient fraction 1 contained some residual contaminating monomeric FRL-PSI and some possible assembly or dissociation intermediates of FRL-PSII. The apo-FRL-PSII complexes in fraction 2 had absorbance maxima at 672 and 722 nm (Fig. S1*B*) and had a relatively narrow fluorescence emission band with a maximum at 739 nm at 77 K (Fig. S1*C*). Pigment analysis of the apo-FRL-PSII complex revealed that it contained 1.4 pheophytin *a* molecules and ∼35 total Chl molecules, including 0.7 Chl *d*, 4.2 Chl *f*, and 30.1 Chl *a*.

SDS-PAGE (Fig. S1*D*) showed that the apo-FRL-PSII complexes were free from contamination by FRL-PSI. Tryptic peptide fingerprinting by mass spectrometry showed that these complexes contained PsbA3, PsbB2, PsbC2, PsbD3, PsbE, PsbF1 and PsbF2 (*Synechococcus* 7335 has two different *psbF* genes that are not modulated by FaRLiP), PsbH2, PsbK, and PsbI (Table S1). Note that the genes encoding PsbA3, PsbB2, PsbC2, PsbD3, and PsbH2 subunits are in the FaRLiP gene cluster (5, 6) and are specifically transcribed when cells are grown in FRL. These FRL-specific subunits share ∼80, 70, 64, 83, and 48% sequence identity, respectively, compared with their paralogs expressed in WL. To differentiate among subunit paralogs, we hereafter refer to all PSII subunits according to their genetic designations; rather than the commonly used nomenclature for PSII subunits (D1, D2, CP47, and CP43), we will use PsbA, PsbD, PsbB, and PsbC, respectively, and related names (*e.g.*, PsbB1 [WL] or PsbB2 [FRL]) that specify paralogs in organisms that can perform FaRLiP. Notably, none of the lumenal extrinsic subunits associated with the OEC (PsbO1/PsbO2, PsbU, and PsbV) were detected, and several small membrane-intrinsic subunits normally found in dimeric PSII complexes were not present. Minor amounts of two WL-specific protein subunits, PsbC1 and PsbD1/PsbD2, were also present in fraction 2 (Table S1).

### Overall structure

**Figure 1:**
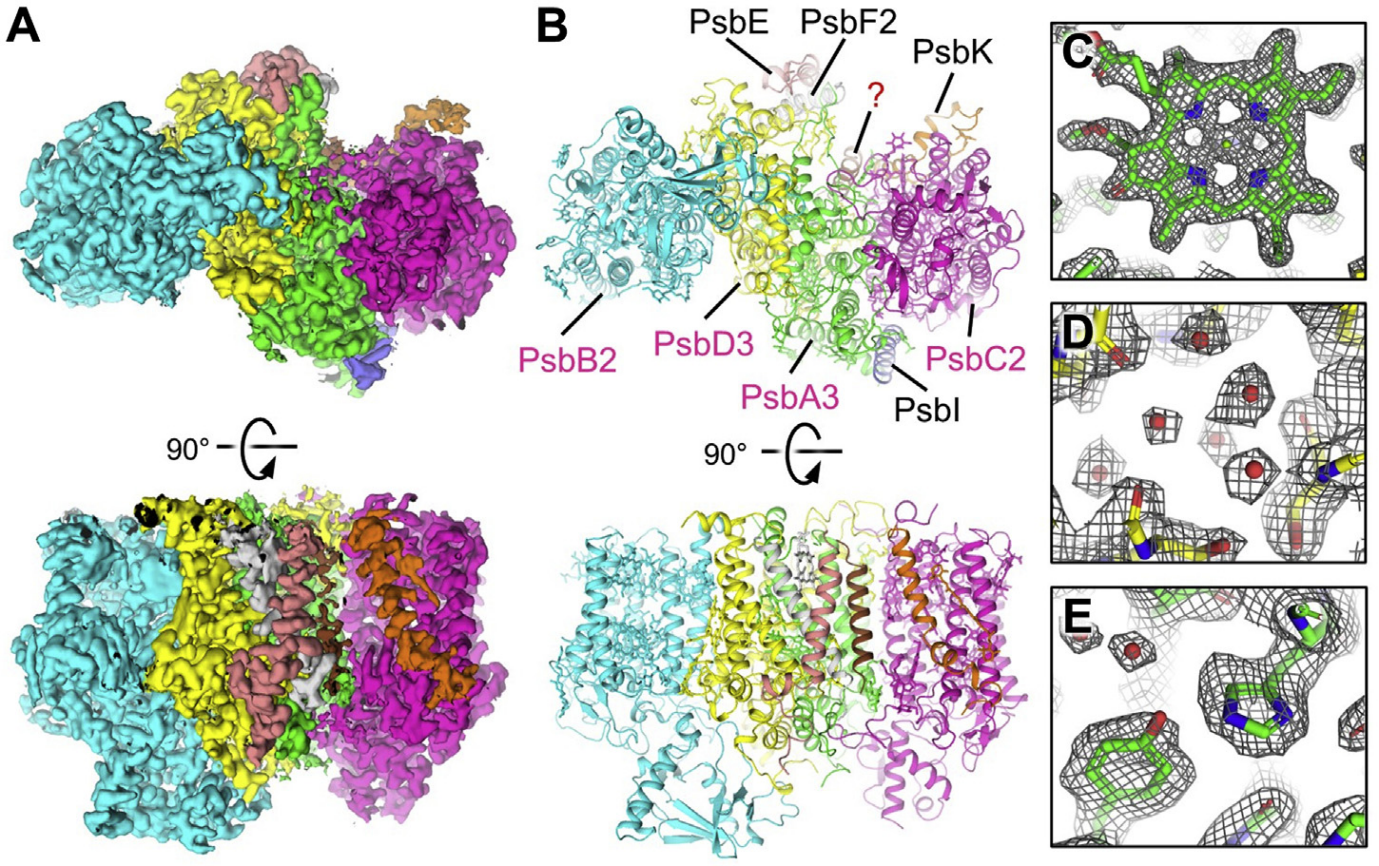
**Overview of monomeric apo-FRL-PSII from *Synechococcus* 7335 and map examples.***A*, unsharpened ESP map (7.5σ) in a lumenal view (*top*) and a membrane plane view (*bottom*). *B*, model built into the ESP map in the same views as *A*. In the lumenal view (*top*), subunits identified in the map are labeled. Subunits labeled in *pink font* correspond to those that are FRL-specific isoforms. The unidentified protein subunit is labeled “?”. *C*, model of Chl P_D1_ of the ETC cofactors in the ESP map (18σ). *D*, model of a water cluster associated with the lumenal side of PsbD3 subunit nearby PsbE in the ESP map (12σ). *E*, model of Tyr_Z_ hydrogen bonded to a water molecule and the ε^2^ nitrogen of PsbA3-His190 in the ESP map (12σ). Chl, chlorophyll; ESP, electrostatic potential; ETC, electron transfer chain; FRL, far-red light; PSII, photosystem II.

Cryo-EM sample preparation, data collection, and processing were performed as described in the Experimental procedures section. The processing procedure for the cryo-EM data is outlined in Figure S2. Data processing resulted in an ESP map having a global resolution of 2.25 Å (Fig. S3) with local resolutions ranging from 2.1 to 2.9 Å (Fig. S4). Cryo-EM data statistics are reported in Table S2. Nine protein subunits, 56 cofactors, and 230 water molecules were modeled (Fig. 1), corresponding to a total molecular mass of ∼226 kDa. The following protein subunits fit the ESP map: PsbA3, PsbB2, PsbC2, PsbD3, PsbE, PsbF2, PsbI, PsbK, and an unknown intrinsic subunit with a single transmembrane α-helix. Cofactors modeled include 28 Chl *a* molecules, six *n*-dodecyl-β-d-maltoside (β-DM) molecules, five β-carotenes, five diacyl lipids, four Chl *f* molecules, two pheophytin molecules, one Chl *d* molecule, one heme *b*, one plastoquinone, one NH-Fe ion, one chloride ion, and one calcium ion. Representative tetrapyrrole models within their corresponding ESP maps are shown in Figure S5. It is notable that ESP corresponding to the PsbH2 subunit, which was detected by proteomic analysis after the sucrose gradient centrifugation step, was not identified in the cryo-EM map.

To compare the apo-FRL-PSII structure to other cyanobacterial PSII structures, we performed C_α_ superpositions of the apo-FRL-PSII subunits onto the analogous subunits from the X-ray diffraction (XRD) structure of the dimeric PSII holocomplex from *Thermosynechococcus vulcanus* (Protein Data Bank [PDB] ID: 3WU2) (47) and the cryo-EM structure of apo-PSII from *Synechocystis* sp. PCC 6803 (PDB ID: 6WJ6) (29) (Table S3). All subunit superpositions exhibited low root-mean-square deviation, indicating that their structures are very similar, which is consistent with the relatively high sequence identity and similarity shared among them (Table S4). Unlike other PSII structures, the apo-FRL-PSII structure contains an additional subunit with a single transmembrane helix in a position that is not occupied by a subunit in other PSII structures. Relative to the PSII from *T. vulcanus*, the location of the unknown subunit is opposite the dimeric interface, somewhat near PsbJ (Fig. S6). By comparing map thresholds in which the α-helical secondary structure can be observed relative to that of other nearby transmembrane helices where *B*-factors may be similar, we estimate the occupancy of the unknown single transmembrane helix to be ∼40%. Amino acid side chains could not be resolved, and therefore, the subunit was not assigned to a protein sequence and was modeled as a poly-Ala helix of 23 residues.

Compared with the XRD structure of mature (active) dimeric PSII from *T. vulcanus*, two of the 35 Chl molecules and six of the 11 carotenoid molecules are not present in the apo-FRL-PSII structure (Fig. S7). These missing pigments correspond to peripheral binding sites, and their absence may be due to destabilization caused by the loss of the small transmembrane subunits. Note that hereafter we maintain the site numbering of pigments originally assigned in the mature dimeric PSII XRD structure from *T. vulcanus* (47) (Fig. S8). One of the missing Chls, PsbB2-615, is bound near the dimerization interface; the other, PsbB2-602, normally binds in one of the most peripheral regions of PSII near where PsbH is found in the mature PSII complex. The missing Chls almost certainly would be present in a mature holocomplex because the regions of PsbB2 where they should be bound are very similar in sequence to the corresponding regions of PsbB1 (Fig. S9). All six missing carotenoids in the apo-FRL-PSII structure are either associated with missing subunits or are bound at the dimerization interface.

### FRL-specific changes

**Figure 2:**
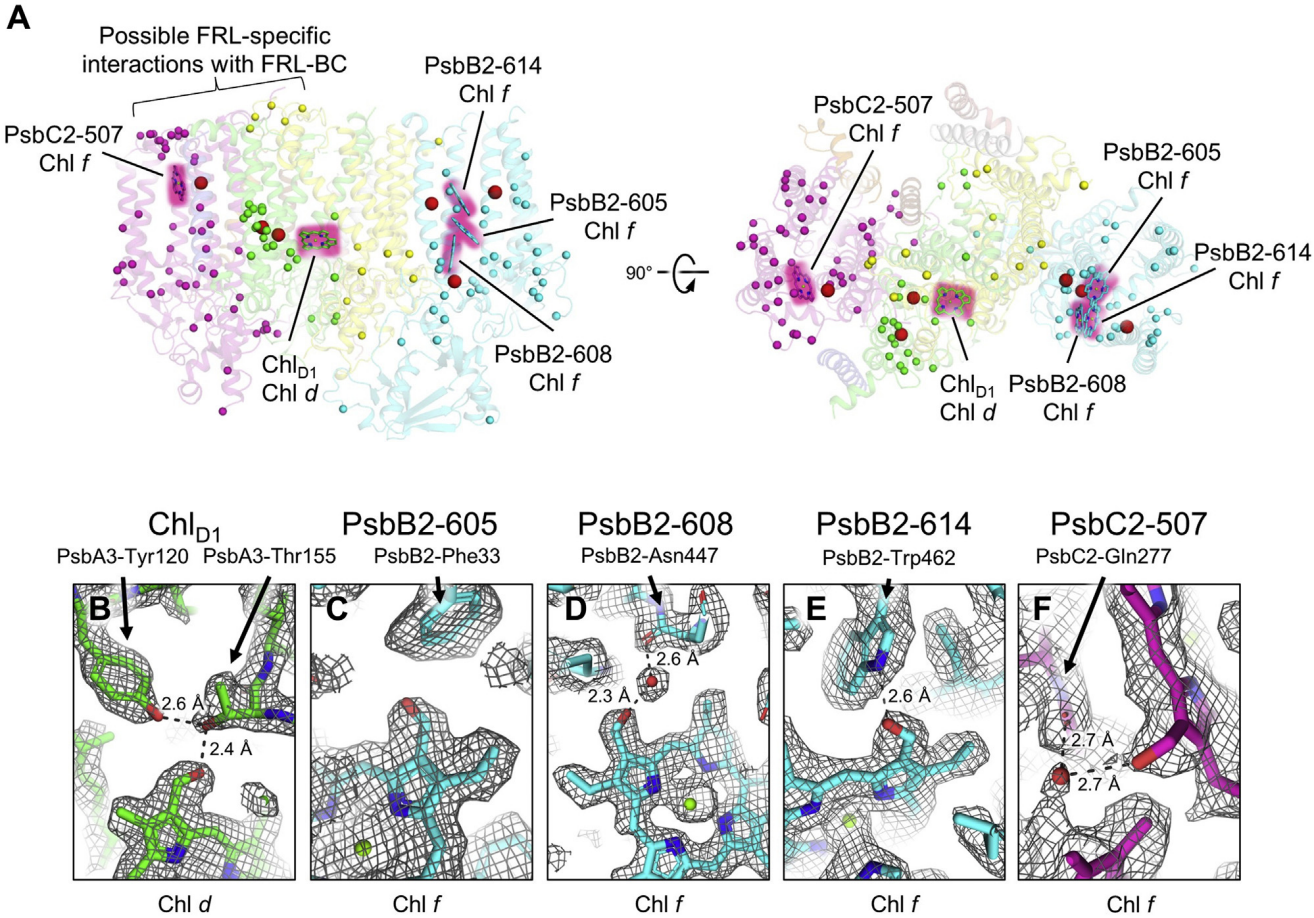
**FRL-specific sequence differences and identification of Chl *d* and Chl *f* molecules in *Synechococcus* 7335 apo-FRL-PSII.***A*, apo-FRL-PSII model showing a sphere for the C_α_ atom of each FRL-specific residue assigned in the sequence alignments (Fig. S9). *Spheres* are colored according to their subunit, except those that are involved in FRL-specific interactions near the C3 or C2 positions of the tetrapyrrole rings for assigned Chl *d* and Chl *f* molecules (*red spheres* correspond to *red arrows* in Fig. S9). The tetrapyrrole rings only are shown with a *pink glow* for assigned Chl *d* and Chl *f* molecules and are in addition labeled with their site name and identity. The *left view* shows a membrane plane view, and the *right view* shows a stromal side view. The possible PsbD3/PsbC2 region that may interact with FRL-BC is also labeled (Fig. S10). *B*, model in map (16σ) focusing on the C3 formyl substituent of Chl_D1_ that is assigned as Chl *d*. The FRL-specific hydroxyl moiety of PsbA3-Thr155 donates a hydrogen (H) bond to the C3 formyl moiety. *C*, model in map (14σ) focusing on the C2 formyl substituent of Chl PsbB2-605. The FRL-specific PsbB2-Phe33 is instead Trp in PsbB1 (the WL isoform). *D*, model in map (15σ) focusing on the C2 formyl substituent of Chl PsbB2-608. A water molecule H-bonded to the backbone carbonyl O of FRL-specific PsbB2-Asn447 donates an H-bond to the C2 formyl moiety. *E*, model in map (15σ) focusing on the C2 formyl substituent of Chl PsbB2-614. The indole N atom of the FRL-specific PsbB2-Trp462 side chain donates an H-bond to the C2 formyl moiety. *F*, the model in map (7σ) focusing on the C2 formyl substituent of Chl PsbC2-507. A water molecule H-bonded to the backbone carbonyl O of FRL-specific PsbC2-Gln277 donates an H-bond to the C2 formyl moiety. BC, bicylindrical core antenna complex; Chl, chlorophyll; FRL, far-red light; PSII, photosystem II; WL, white light.

Based on our pigment analyses, one Chl *d* molecule and about four Chl *f* molecules are expected to be bound by FRL-PSII. In structures of FRL-PSI, it was shown that Chl *f*-binding sites are often located near FRL-specific sequence differences that may provide binding specificity (10, 13, 14). To identify FRL-specific sequence differences in the apo-FRL-PSII structure, we created sequence alignments (48) using FRL-specific and WL-specific core subunit polypeptides from three cyanobacteria capable of FaRLiP, *Synechococcus* 7335, *Halomicronema hongdechloris*, and *Fischerella thermalis* PCC 7521 (Fig. S9). Regions that exhibit FRL-specific differences were mapped to the apo-FRL-PSII structure (Fig. 2*A*). One cluster of FRL-specific sequence differences is concentrated in PsbA3 near the ETC, suggesting that a Chl molecule other than Chl *a* may be bound nearby. Another cluster of FRL-specific differences is observed on the stroma-facing surfaces of PsbC2 and PsbD3 (Figs. 2*A* and S10). These surfaces are near the proposed interaction regions for the binding of PBS (49, 50), where FRL-BC probably binds in an equivalent position during FaRLiP in *Synechococcus* 7335 (8, 15). Otherwise, FRL-specific sequence differences are sparsely distributed throughout the structure, unlike FRL-PSI (10, 13, 14).

**Figure 3:**
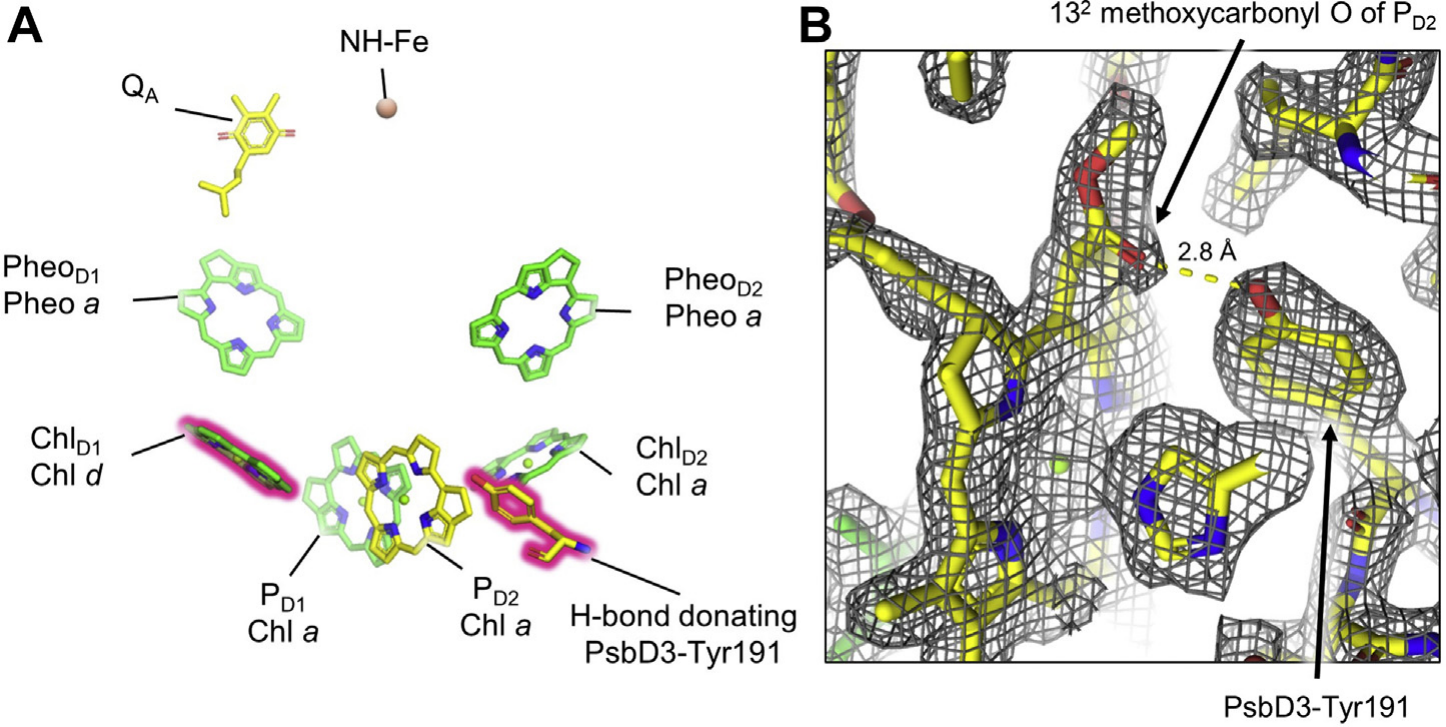
**FRL-specific changes in the ETC.***A*, ETC cofactors present in the apo-FRL-PSII structure are shown. The Chl *d* and the FRL-specific PsbD3-Tyr191 are shown with a *pink glow*. Isoprenoid tails are truncated for clarity. *B*, model in the sharpened ESP map (17σ) near P_D2_ showing the H-bond donation from FRL-specific PsbD3-Tyr191 to the 13^2^ methoxycarbonyl O atom of P_D2_. ESP, electrostatic potential; ETC, electron transfer chain; FRL, far-red light; PSII, photosystem II.

Whereas Chl *a* exhibits methyl and vinyl moieties at positions C2 and C3 on the tetrapyrrole ring, respectively, Chl *d* has a formyl moiety at C3, and Chl *f* has a formyl moiety at C2 (6, 51, 52) (Fig. S5). Based on our pigment analyses, we expected to find one Chl *d* molecule. We visually inspected the ESP map for possible H-bond donors to the C3 position of each of the 33 Chls in the structure to find the potential Chl *d*-binding site. The only Chl exhibiting an obvious H-bond donation to the C3 position is Chl_D1_ in the ETC (Fig. 2*B*), and the two residues involved in this H-bonding specifically occur in polypeptides expressed in FRL in the sequence alignment (Fig. S9). Furthermore, the cluster of FRL-specific sequence differences near the ETC is closest to Chl_D1_ (Fig. 2*A*). Based on these observations, we conclude that Chl_D1_ is Chl *d*, and it was assigned as such in the structural model. We also analyzed each of the ETC cofactors for other FRL-specific alterations. We observed that an H-bond is donated by the FRL-specific side chain of PsbD3-Tyr191 to the 13^2^ methoxycarbonyl O atom of the Chl *a* in site P_D2_ (Fig. 3).

To search for Chl *f* molecules, we visually inspected the C2 position of each Chl in the map for additional ESP and possible H-bond donors. Four Chl sites exhibit ESP signals greater than would be expected for a methyl moiety alone by visual inspection: PsbB2-605, PsbB2-608, PsbB2-614, and PsbC2-507 (Fig. 2). The latter three of these also have nearby H-bond donors to the C2 formyl moiety where the residues involved are FRL specific (Figs. 2, *C*–*F* and S9). Although it does not provide an H-bond, the Phe side chain near the assigned formyl moiety of PsbB2-605 is also FRL specific, whereas it is a bulkier conserved Trp in WL-specific PsbB1 sequences (Fig. S9). To provide a quantitative assessment of the ESP map for the identification of Chl *f*, we performed cone scans as described previously (13) (see the Experimental procedures section and Data S1). The Chls at sites PsbB2-605, PsbB2-608, and PsbB2-614 exhibit ESP in their C2 cone scans that are greater than the C7-derived methyl distribution, supporting their assignment as Chl *f* (Fig. S11). The Chl at site PsbC2-507 is found in a lower resolution region of the map, and its C2 cone scan does not exceed the methyl distribution, consistent with the observation that lower resolution limits the ability to distinguish between formyl and methyl substituents (13). However, a conserved FRL-specific H-bond donor to the C2 moiety provides strong evidence that Chl *f* is bound to this position, consistent with the visual inspection. Based on the observations described here, Chl *f* molecules were assigned in sites PsbB2-605, PsbB2-608, PsbB2-614, and PsbC2-507 (Fig. 2*A*). All these sites are in the core-intrinsic antenna domains of FRL-PSII.

### Acceptor-side perturbations

**Figure 4:**
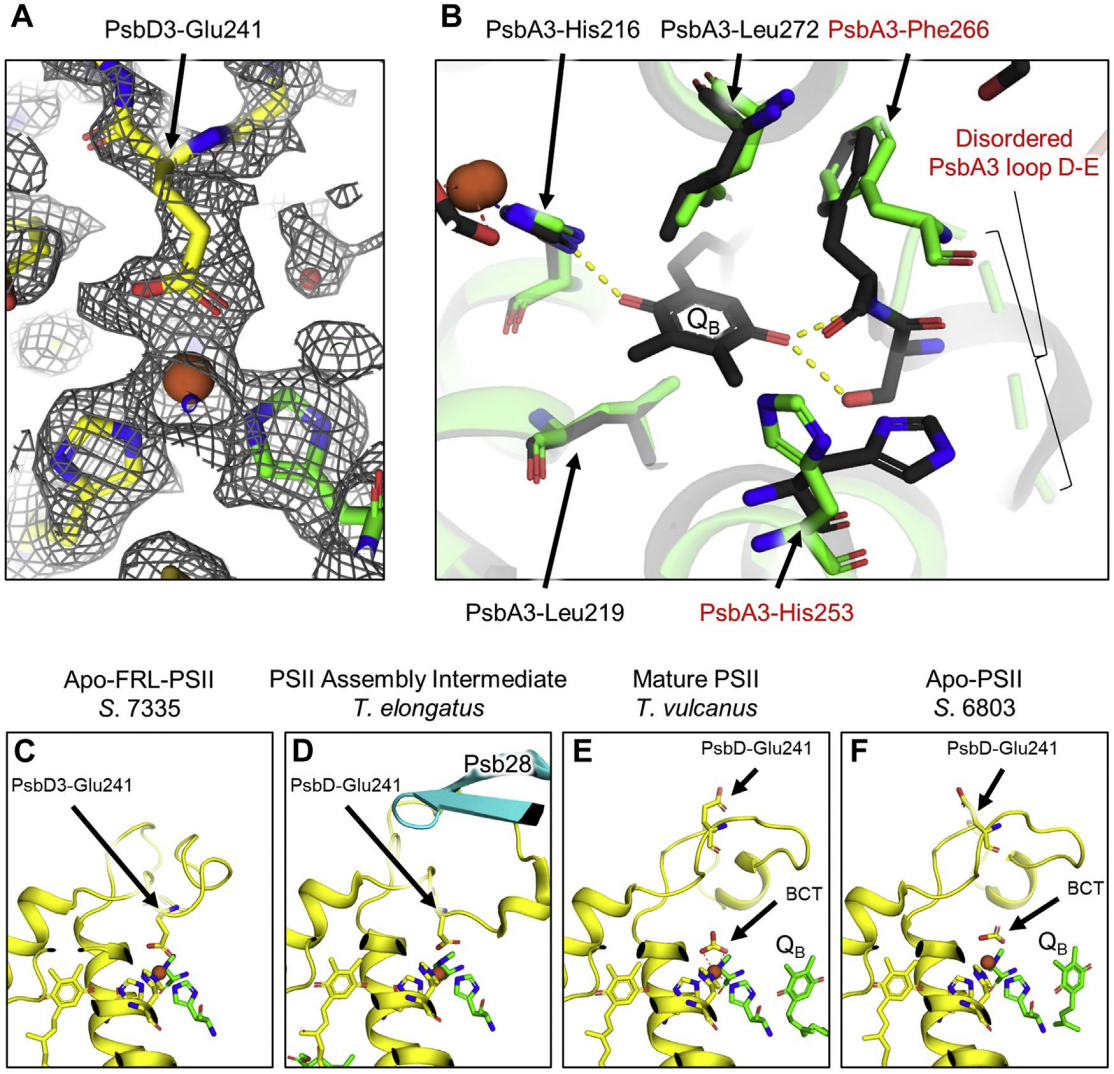
**Acceptor-side perturbations and comparison with other PSII structures.***A*, the apo-FRL-PSII model from *Synechococcus* 7335 is shown within the sharpened ESP map (9.5σ) focusing on PsbD3-Glu241 and its ligation to the NH-Fe. *B*, a structural superposition is shown of apo-FRL-PSII from *Synechococcus* 7335 (*colored*) and mature PSII from *Thermosynechococcus vulcanus* (*black*, PDB ID: 3WU2). Residues are labeled from the *Synechococcus* 7335 structure where *red font* denotes residues with significantly different positions compared with mature PSII. Note that the Ser side chain H-bonding to Q_B_ in mature PSII is PsbA-Ser264. The analogous residue in *Synechococcus* 7335 is PsbA3-Ser265, and it is not resolved in the ESP map (denoted by the *dashed cartoon*, labeled as “Disordered PsbA3 loop D–E”). Also note that all PsbA3 residues are numbered one greater in the *Synechococcus* 7335 PsbA3 sequence than they are in the sequence of *T. vulcanus* PsbA†. *C*–*F*, all panels show the cartoon representation of PsbD(3) and Psb28 when present. The stick representations of PsbD(3)-Glu241, Q_A_, the NH-Fe-coordinating His residues, and Q_B_ and bicarbonate (labeled “BCT”) when present. *C*, the structure of apo-FRL-PSII from *Synechococcus* 7335 reported here. *D*, the structure of a PSII assembly intermediate from *Thermosynechococcus elongatus* (PDB ID: 7NHP). *E*, the structure of mature PSII from *T. vulcanus* (PDB ID: 3WU2). *F*, the structure of apo-PSII from *Synechocystis* sp. PCC 6803 (PDB ID: 6WJ6). ESP, electrostatic potential; FRL, far-red light; NH-Fe, non-heme Fe(II); PDB, Protein Data Bank; PSII, photosystem II.

In mature PSII, a NH-Fe is located between the Q_A_ and Q_B_ plastoquinones. The NH-Fe typically has five ligands: four His side chains (two from PsbA [D1]) and two from PsbD [D2]) and a bidentate bicarbonate anion (47). In the apo-FRL-PSII structure, the carboxylate side chain from a conserved PsbD3-Glu241 (Fig. S12), which is a residue within loop D–E of PsbD3, replaces the bicarbonate as a bidentate ligand to the NH-Fe (Fig. 4). This arrangement was also observed in structures of PSII assembly intermediates with bound Psb28, a PSII assembly factor (27, 31). The authors of those studies suggest that bicarbonate is replaced by PsbD-Glu241 because of the binding of Psb28 (Fig. 4).

In mature PSII structures, the Q_B_ plastoquinone is bound to PsbA in a pocket adjacent to the NH-Fe. One of the keto O atoms on the Q_B_ headgroup accepts an H-bond from the N^δ^ atom of a His side chain (whose N^ε^ atom provides one of the ligands to the NH-Fe). The other keto O atom accepts H-bonds from the backbone amide N atom of a Phe side chain and the hydroxyl side chain of a Ser residue, both of which are in loop D–E of the PsbA subunit. Hydrophobic residues are found nearby the Q_B_ headgroup and through the channel occupied by its isoprenoid tail. The apo-FRL-PSII structure lacks Q_B_, and the PsbA loop D–E is disordered, some of its residues being found in different positions compared with mature PSII (Fig. 4). Specifically, PsbA3 residues Phe266 and Ser265 are further away from the Q_B_-binding site. Ser265 is unresolved in the ESP map along with two other residues (Fig. S9), and the side chain of PsbA3-His253 is rotated inward, toward the Q_B_-binding pocket.

### Vacant OEC-binding site and nearby cation

On the electron donor side, mature PSII contains the OEC that oxidizes water and reduces Tyr_Z_^+^^⋅^, which subsequently reduces P_680_^+^ in the ETC following charge separation. Our apo-FRL-PSII structure lacks the OEC as has been reported for some other recent cryo-EM structures of PSII (27, 28, 29, 30, 31). The map quality of the OEC-binding site is somewhat poor, likely because of heterogeneity and flexibility in that region. Some previous cryo-EM maps of PSII lacking the OEC contain an ESP signal proposed to arise from a cation bound between the carboxylate side chains of PsbA-Asp170† and PsbA-Asp189 (27, 28, 30) that have been suggested to comprise the high-affinity Mn-binding site (29, 53) involved in the initial steps of photoactivation (26). Although we observe an ESP signal between PsbA3-Asp170 and PsbA3-Glu189 in the apo-FRL-PSII structure, it corresponds better to two water molecules and is modeled as such. Relative to the mature PSII structure, the cyanobacterial cryo-EM PSII structures lacking the OEC also feature the movement of the PsbA-His332 side chain toward the Cl^–^1 site and the rest of the PsbA (D1) C terminus, changing its conformation relative to the mature structures (27, 28, 29). The analogous residue in the apo-FRL-PSII structure also exhibits this configuration. The high resolution achieved in our data allowed us to resolve all but the last four residues of the processed PsbA3 C-terminal region, although this region is modeled with lower confidence and is probably quite flexible (Fig. S13).

Although we observe no signal for a cation between PsbA3-Asp170† and PsbA3-Glu189, there is a large ESP signal nearby, ∼6 Å away from the proposed high-affinity Mn-binding site, that corresponds to a cation (Fig. S14). Unlike other PSII structures in which the OEC is also absent, PsbA3-Glu189 is shifted toward this cation in the apo-FRL-PSII structure (Fig. S15). PsbA3-Glu329 and Glu333 are also positioned toward the cation, although the latter is >3 Å away, beyond the range for a coordination bond. We note, however, that distance measurements in this region may be unreliable because of the limited quality of the ESP map. The cation is in addition bound by two water molecules and the backbone carbonyl O atom of PsbC2-Gly405, which is found within a looping region of the lumenal soluble domain of PsbC2 (CP43). PsbC-Gly405 was not modeled in the apo-PSII structure from *Synechocystis* sp. PCC 6803, presumably because of flexibility (Fig. S15). We cannot determine the chemical identity of this cation based on the ESP map, but it is most likely a calcium ion, which was present at a concentration of 15 mM in the sample buffer, and it was therefore modeled as such.

## Discussion

We assign Chl *d* as Chl_D1_, the primary electron donor in PSII (54, 55, 56), with high confidence, showing that changes occurring during FaRLiP occur at the very heart of the FRL-PSII ETC. Based on spectroscopic data, two reports suggested that either Chl *d* or Chl *f* is found in the Chl_D1_ site of the ETC in FRL-PSII (17, 18). Those authors favored Chl *f* occupying that site; however, we clearly observe Chl *d* in the Chl_D1_ site of FRL-PSII from *Synechococcus* 7335. More recently, an overlapping group of authors presented arguments for placing either Chl *d* or Chl *f* in the P_D1_ or P_D2_ positions (57), which our structural data do not support.

The identification of Chl *d* in the Chl_D1_ site of the ETC is highly significant. Chl *d* can be used as a spectroscopic marker for studies of electron transfer that had previously been challenging in systems where Chl *a* is the only Chl (18). The wavelength associated with maximal bleaching of the FRL-PSII primary donor was determined to be 727 nm in PSII from FRL-acclimated *Chroococcidiopsis thermalis* (17), and the FRL-specific Tyr120 and Thr155 residues involved in H-bonding to the C3 formyl substituent of Chl *d* in the Chl_D1_ site are well conserved among PsbA3 sequences (Fig. S9). Therefore, assuming there are no major species-specific differences, we can assign Chl *d* to the donor bleaching at 727 nm. This value is similar to the wavelength of the primary donor bleaching at 725 nm in *Acaryochloris marina*, which is also Chl *d* (58, 59). Thus, it appears that ∼727 nm is currently the known “red limit” for the energy requirement to achieve water oxidation in oxygenic photosynthesis.

This new “red limit” raises questions about the lower energy limit for photochemistry in oxygenic photosynthesis, and the Chl *d* position is crucial to its functional role in FRL-PSII. A photon of 727 nm has 120 meV less energy than the photon of 680 nm typically used by PSII. This change will either make Chl_D1_^+^ a poorer electron acceptor from the OEC or Chl_D1_∗ a poorer electron donor. The energy difference can be split between the redox potential of P∗/P^+^ and P/P^+^ redox couples. It is interesting that PsbD3-Tyr191 donates an H-bond to the 13^2^ moiety of P_D2_ (Fig. 3), which likely influences its electronic structure and possibly tunes its energy to accommodate the presence of Chl *d* at Chl_D1_. It has been shown in anoxygenic quinone-type reaction centers that engineered H-bond donors to P increased the P/P^+⋅^ midpoint potential (60), but it is unknown if that occurs here. The rate of formation of P^+⋅^ is known to be slower in FRL-PSII than in WL-PSII (18), which may be a consequence of P∗ being at lower potential because of the H-bond to P_D2_ and/or the presence of Chl *d* at Chl_D1_. The FRL-specific H-bond to P_D2_ could cause an increase in the rate of Tyr_Z_ oxidation and disfavor Tyr_D_ oxidation in FRL. This could be beneficial in low light environments, which are usually enriched in FRL and where protecting against photodamage is less of a priority than achieving water oxidation.

**Figure 5:**
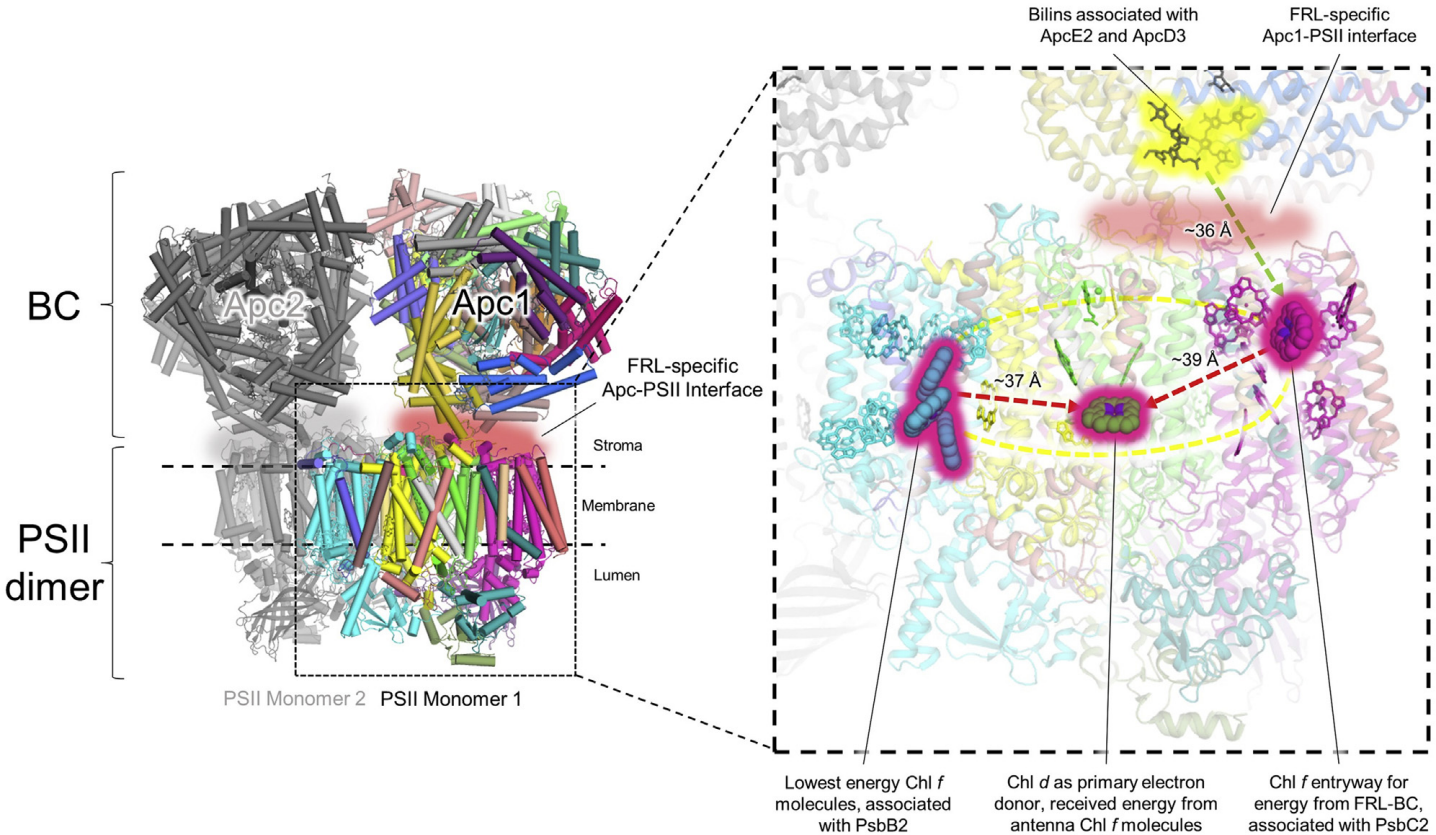
**Proposed pathways of energy transfer for FRL-AP and FRL-PSII.** The *left panel* shows a depiction of dimeric apo-FRL-PSII with a FRL-BC bound to its stromal side (8, 14). *Colored subunits* correspond to one PSII monomer and its associated FRL-AP cylinder (Apc1), and *gray subunits* correspond to a second FRL-PSII monomer and its corresponding FRL-AP cylinder (Apc2). The *right panel* shows a magnification of the *dashed box* region in the *left panel*. In the *right panel*, the bilins of ApcE2 and possibly ApcD3 that are closest to the stromal surface of FRL-PSII are shown with a *yellow glow*, and the suggested FRL-specific interface is shown as a *red shade*. The FRL-specific Chls of FRL-PSII are shown in *sphere representation* with a *pink glow* and are labeled. Proposed pathways of energy transfer are shown using *dashed arrows*. Distances between the bilins in ApcD3 and ApcE2 to the PsbC2 pigments are approximate and based on the models of Zlenko *et al.* (99). Distances between Chl *f* molecules and Chl *d* molecules are based on the apo-FRL-PSII structure presented here. The model shown was created using UCSF Chimera (93), by removing all but the bicylindrical core from the PBS structure from *Synechococcus* sp. PCC 7002 (PDB ID: 7EXT) (100) and manually placing a dimeric model of PSII (PDB ID: 3WU2) in the orientation shown. BC, bicylindrical cores; Chl, chlorophyll; FRL, far-red light; FRL-AP, FRL-specific allophycocyanin; PBS, phycobilisome; PDB, Protein Data Bank; PSII, photosystem II.

The arrangement of three Chl *f* molecules in PsbB2 and one Chl *f* molecule in PsbC2 may be related to energy transfer from the FRL-BC in *Synechococcus* 7335 or FRL-PBS in other organisms to the core antenna Chls and subsequently Chl *d* at Chl_D1_. The cluster of FRL-specific sequence differences on the stromal surface of PsbC2 (Figs. 2 and S10), most of which consist of polar and charged residues, is consistent with the idea that FRL-PSII discriminates against binding typical hemidiscoidal PBS when cells are grown in FRL or intermediate light conditions. In some organisms such as *Synechococcus* 7335, PBSs appear to be completely replaced by FRL-BC core substructures over time in FRL (8, 15), but in some other organisms, for example, *Leptolyngbya* sp. JSC-1, FRL-PBSs are produced that resemble PBS but have a different core substructure made with FRL-AP encoded in the FaRLiP gene cluster (5, 15, 16). Although other opinions exist (*e.g.*, Liu *et al.* (61)), it is generally believed that energy transfer from PBS to PSII occurs *via* the terminal emitter protein ApcE to the core antenna Chls of PsbC (CP43) (49, 50). However, during growth in FRL, the AP subunits normally expressed in visible light are replaced by paralogous FRL-AP subunits specific for FRL (6, 62, 63, 64). This includes the replacement of ApcE1 by ApcE2 (5, 8, 9, 15, 16, 65). If this general pattern concerning energy transfer is similar in FRL, we envision that the Chl *f* molecule at PsbC2-508 would likely be very important for energy transfer from FRL-BC to the ETC Chl *d* of FRL-PSII (Fig. 5). Assuming the absence of species-specific variation between FRL-PSII from different organisms, PsbC2-508 may correspond to the shortest wavelength Chl *f* observed in spectroscopic data of FRL-PSII, ∼712 to 721 nm (17, 18). This shorter wavelength “linker” Chl *f* could serve as a bridge between FRL-AP (710–715 nm) in the FRL-BC while still being able to participate in uphill energy transfer to Chl *a* and transfer of energy to Chl_D1_ (Chl *d*) in the ETC.

PsbB2 contains a cluster of three Chl *f* molecules (Fig. 2). One unique Chl *f* site, PsbB2-605, is coordinated by a His side chain and does not exhibit an H-bond to its C2 formyl moiety (Fig. 2). This is unlike any of the other Chl *f* sites identified in this apo-FRL-PSII structure or those assigned in FRL-PSI structures (12, 14), suggesting that it is a unique Chl *f*-binding site. The ability to bind Chl *f* at this site is instead conferred by a FRL-specific motif in which a Phe is present near the C2 moiety rather than the larger Trp side chain found in WL sequences (Fig. S9) and other non-FaRLiP sequences. This allows sufficient space for the formyl moiety by avoiding steric hindrance. This configuration is reminiscent of a “bump-and-hole” strategy used in protein engineering efforts to confer cofactor specificity to study enzyme–substrate interactions (66). It is also reminiscent of a recent cryo-EM structure of the PSI-like photochemical reaction center from the anoxygenic chlorophototroph, *Chlorobaculum tepidum*, which uses bacteriochlorophyll *a* as its major antenna pigment (67) but confers specificity to ETC sites to bind Chl *a* by steric hindrance near the C3 position (68). The unique coordination of the Chl *f* at PsbB2-605 may be related to its position, somewhat overlapping with the Chl *f* at site PsbB2-614. This pair of Chl molecules may contribute the longest wavelength absorption and thus be important for the lowest energy light harvesting in the complex and transfer of energy to Chl *d* in the ETC. Interestingly, all Chl *f* molecules are approximately equidistant to the Chl *d* molecule in the ETC (Fig. S16), suggesting that energy transfer kinetics and efficiency might be similar for all four Chl *f* molecules. These observations concerning the distribution of Chl *f* molecules between PsbC2 and PsbB2 imply that the two core antenna modules in FRL-PSII may have slightly different functions in energy harvesting and energy transfer to the ETC (Fig. 5). This is supported by the observation that the only Chl *f* molecule found in PsbC2 is close to the stromal side, where FRL-BC is expected to bind (Fig. 5), whereas the Chl *f* molecules found in PsbB2 are either in the center of the membrane or closer to the lumenal side. These ideas can be tested through future mutagenesis and spectroscopic studies.

Aside from the Chl *f* at site PsbB2-605, the other Chl *f*-binding sites are consistent with previously observed structural configurations of Chl *f* binding (10, 12, 13). All the residues participating in axial ligation of the Chl *d* and *f* molecules are conserved between the FRL and WL sequences, so it does not appear that FRL-specific changes in axial ligation are implemented in FRL-PSII. This is unlike the change in axial ligation from His in WL to water in FRL observed for the Chl *f* molecule at site A21 in FRL-PSI structures (10, 12, 13, 14) that could participate in fine-tuning its site energy. The Chl *f* in site PsbB2-614 is axially coordinated by a His side chain, but the C2 formyl moiety accepts an H-bond from the indole N atom of a Trp side chain, similar to the Chl *f* in site B30 of FRL-PSI (10, 13). The other two Chl *f* sites at PsbB2-608 and PsbC2-507 feature axial coordination by a water molecule, and both their formyl moieties accept an H-bond from a water molecule. Chl *f* in site PsbC2-507 was predicted previously by homology modeling (13). That study also suggested that PsbB2-611 binds Chl *f*. The region of the ESP map near the C2 position of PsbB2-611 is not well resolved, probably because of its destabilization due to subunit loss, so it could possibly bind Chl *f*. However, the number of Chl *f* sites we assigned is consistent with our cofactor analysis. Therefore, if PsbB2-611 does bind Chl *f*, it probably does so with low specificity. Indeed, some low-specificity Chl *f-*binding sites have recently been proposed in FRL-PSI (14). Nevertheless, the high resolution achieved here nicely resolves most of the C2 positions of Chls in the PSII complex, especially those closest to the ETC where higher specificity for binding Chl *f* in specific locations is more likely to result in efficient energy transfer and charge separation. It should be noted that these cells continue to make an excess of Chl *a*, so the sites that bind Chl *d* and Chl *f* must have higher affinity for these Chls.

Another interesting observation regarding the subunit content of apo-FRL-PSII is the presence of an unknown transmembrane helix (Fig. S6). We could find no unannotated hypothetical proteins identified by mass spectrometry of tryptic peptides that could account for this subunit. The most obvious possibilities are that it is either one of the PsbF paralogs or PsbH2, all of which are single transmembrane PSII subunits found in the peptide fingerprinting analysis (Table S1). While modeling the canonical position of PsbF as the β-subunit of cytochrome *b*_559_, we determined that PsbF2 fits the ESP map better than PsbF1 based on two residues that are distinct between the two very similar sequences (Fig. S17). This assignment should be considered tentative, as the differentiating residues are a challenge to resolve in this region of the map. The unknown subunit could be related to a relatively unique and recent gene duplication of PsbF1/PsbF2 in *Synechococcus* 7335. The duplicated gene occurs in an operon together with *psbO1*, an *nblA* paralog, and *psbV* (cytochrome *b*_550_). It is intriguing that the *psbF2* gene occurs just upstream from *psbV* because the unidentified subunit could interact with the N-terminus of that polypeptide. The *psbF* gene, encoding the β-subunit of cytochrome *b*_559_, is usually closely linked to *psbE*, encoding the α-subunit of cytochrome *b*_559_, in an operon, *psbEFLJ*, that is conserved in all oxygenic phototrophs. PsbF1 is encoded by such a gene in *Synechococcus* 7335. PsbF1 and PsbF2 share 75% sequence identity, and both contain the conserved His residue that normally participates in heme *b* coordination with PsbE. Thus, it may be that the β-subunit of cytochrome *b*_559_ contains partial occupancy of both PsbF1 and PsbF2. Alternatively, the duplication of *psbF1* may have allowed functional specialization and subunit exchange to occur so that the gene transcribed with *psbV* could become the β-subunit of cytochrome *b*_559_. This would allow PsbF1 to assume a different and unique binding site in FRL-PSII. This interpretation is supported by the fact that the proteomic analysis of the apo-FRL-PSII core complexes contained both PsbF1 and PsbF2 and no other unassigned hypothetical proteins. The FRL-specific PsbH2 is also present in the peptide fingerprinting analysis (Table S1), but it is unaccounted for in the structure, and so PsbH2 could be the unassigned subunit. However, the PsbH subunit in mature PSII structures is bound in a different location (47), and an alternate binding site would require a substantial structural rearrangement that seems unlikely. Further investigation will be required to determine the identity of this unidentified subunit.

The apo-FRL-PSII structure from *Synechococcus* 7335 provides only the second example of a PSII structure from a mesophilic cyanobacterium, whereas all other reported structures have been derived from thermophiles (29). Relative to the apo-PSII structure from the mesophilic cyanobacterium *Synechocystis* sp. PCC 6803 (29), the subunits that are retained through sample preparation are generally similar. In both, the core PsbA3/PsbA (D1) and PsbD3/PsbD (D2) subunits and the major core antenna subunits PsbB2/PsbB (CP47) and PsbC2/PsbC (CP43) are present in both ESP maps. In addition, both maintain cytochrome *b*_559_ (which comprises PsbE and PsbF), PsbI, and PsbK, all of which have been proposed to be involved in the earliest stages of PSII assembly (25, 69, 70, 71). Their presence in the apo-PSII structures suggests that they are the most stably bound peripheral subunits associated with the PSII core.

The replacement of bicarbonate in the apo-FRL-PSII structure with the side chain of PsbD3-Glu241 is surprising as it seems to contradict recent work on PSII assembly intermediate structures from *Thermosynechococcus elongatus* and *T. vulcanus* (27, 31). In the work by Zabret *et al.* (27), a structure is presented, referred to as PSII-M, in which Psb28 is not bound, and the bicarbonate ligand to the NH-Fe is maintained as in mature PSII structures. They present another structure that has Psb28 bound but lacks bicarbonate called PSII-I (27), which is essentially identical to a more recently reported structure by Xiao *et al.* (31). Like the apo-FRL-PSII structure, the NH-Fe in those structures lacking bicarbonate is replaced by PsbD-Glu241 of the D–E loop of PsbD. Notably, Psb28 interacts with the D–E loops from both PsbA and PsbD that compose the Q_B_-binding and Q_A_-binding sites, respectively, in between which the NH-Fe is bound. The structure corresponding to PSII–I (and equivalently from Xiao *et al*.) shows that the presence of Psb28 blocks Q_B_ binding and alters the structure of the D–E loop of PsbA, in which the Q_B_ headgroup is typically found (27, 31). The authors suggested that in addition to these changes in PsbA, Psb28 also causes the D–E loop of PsbD to adopt a structural configuration in which PsbD-Glu241 replaces bicarbonate (27, 31). The apo-FRL-PSII structure presented here clearly shows that Psb28 is not required to achieve the configuration in which PsbD3-Glu241 ligates the NH-Fe, at least in this organism under these conditions (see the Experimental procedures section). Because the isolation procedure used to obtain this core complex resulted in dissociation of trimers into monomers and removal of various subunits, more data are needed to determine whether the PsbD3-Glu241 ligation of the NH-Fe is physiologically relevant.

It is well established that removal of bicarbonate from the NH-Fe serves a protective role against the production of singlet oxygen (27, 45), so understanding when and how bicarbonate is replaced by D2-Glu241 is an important aspect of PSII photoprotection. It may be that D2-Glu241 is bound to the NH-Fe whenever loop D–E of PsbA(3) is in an immature configuration (whether during assembly, repair, or degradation) that cannot facilitate Q_B_ binding in order to minimize photoinhibition. This would be consistent with the lowered bicarbonate affinity of the NH-Fe in *Synechocystis* 6803 in which the *psbH* gene had been knocked out and the resulting PSII thus lacked PsbH, which the complex presented here also lacks (72), possibly resulting in long-range structural perturbations to the acceptor side of the complex. During PSII assembly, D2-Glu241 may be bound to the NH-Fe even before Psb28 is bound, essentially acting as the default configuration in all stages of acceptor-side maturation/repair. This is reasonable because the ETC, from which charge recombination can result in the production of singlet oxygen, is assembled before Psb28 is bound, prior to the insertion of the CP47 module (25, 70). It is also consistent with the observation that all structures featuring the unique configuration of D2-Glu241 bound to the NH-Fe also lack Q_B_ (Fig. 3).

**Figure 6:**
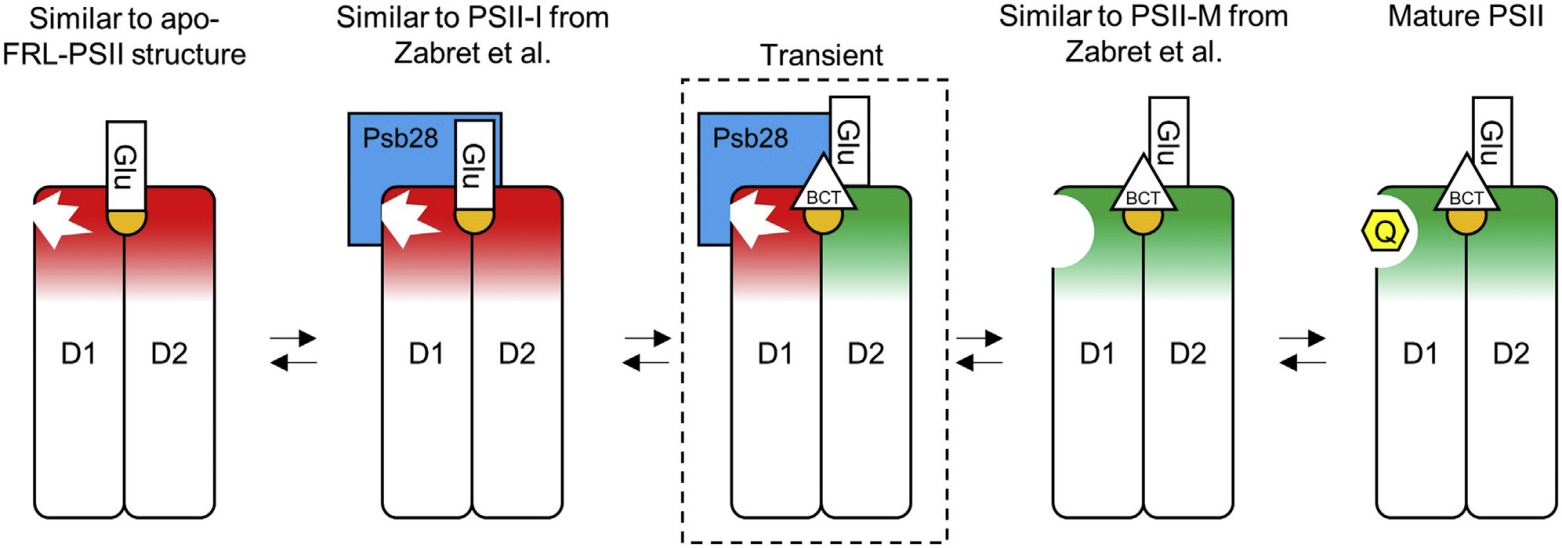
**Mechanistic model of acceptor-side maturation in PSII.** The *cartoon* shows the PsbA(3) (D1) and PsbD(3) (D2) subunits and whether their acceptor-side configuration is immature (*red*) or mature (*green*). The *jagged white* feature denotes an immature Q_B_-binding site. The *orange sphere* denotes the NH-Fe. The “Glu” denotes PsbD(3)-Glu241. The *triangle* labeled “BCT” denotes bicarbonate. The *yellow hexagon* labeled “Q” denotes Q_B_. The states shown are consistent with various published structures as noted except for the transient state (*dashed box*), where bicarbonate binds in the presence of Psb28, replacing PsbD(3)-Glu241 ligation to the NH-Fe and triggering the release of Psb28. NH-Fe, non-heme Fe(II). FRL, far-red light; PSII, photosystem II.

Although the apo-FRL-PSII structure suggests against the dependence of bicarbonate removal with bound Psb28, there is no question that Psb28 causes a major structural rearrangement in the Q_B_-binding site (27, 31). The apo-FRL-PSII structure showed disorder in the D–E loop of PsbA3 that would not facilitate Q_B_ binding (Fig. 4); hence, Psb28 may function to induce a conformational change that allows for the maturation of the Q_B_-binding site that is induced by the replacement of PsbD(3)-Glu241 with bicarbonate when it is available (bicarbonate was not present in the buffer of the cryo-EM assembly intermediates). A mechanistic model that integrates these observations is shown in Figure 6, in which a PSII precursor exhibits an immature acceptor side configuration similar to what is observed in the apo-FRL-PSII structure. *In vivo*, Psb28 binding may alter the structures of the D–E loops of both PsbA(3) and PsbD(3), inducing PsbD(3)-Glu241 replacement by bicarbonate (labeled “BCT” in Fig. 6), similar to the PSII–I state observed by Zabret *et al.* (27) and Xiao *et al.* (31). Bicarbonate displacement of PsbD(3)-Glu241 triggers a structural rearrangement in the loop D–E of PsbA(3) that alters the Q_B_-binding site into its mature configuration and releases Psb28, a state similar to PSII-M (27). We note that this model neglects the contributions of other subunits involved in the maturation of the acceptor side. Furthermore, these steps probably apply to both assembly and disassembly during repair. Future biochemical analyses could test the validity of this model, especially functional assays of PsbD(3)-Glu241 point mutants.

Although the OEC-binding site in apo-FRL-PSII is vacant, it is significant that a cation is present nearby and that the side chain of PsbA3-Glu333† is positioned differently compared with other PSII cryo-EM structures that lack the OEC, shifted toward that cation (Fig. S15). PsbA(3)-Glu333 was long thought to be directly involved in forming the high-affinity Mn-binding site (73, 74), but recent work has suggested that it instead plays a role in cation delivery to the high-affinity site (29). Thus, it is feasible that this cation in the apo-FRL-PSII structure implicates the involvement of PsbD(3)-Glu333 in cation delivery. It is known that an optimal ratio of Mn and Ca ions is important to facilitate and achieve photoactivation of the OEC (75), so it could be that the bound cation represents a highly occupied Ca-binding site when the concentration of Mn is not high enough.

Ligation of a cation in the OEC-binding pocket of an apo-PSII structure by the PsbC(2) lumenal domain has not been observed previously; the only OEC-coordinating interaction that does not come from a PsbA residue in mature PSII is also from the PsbC(2) lumenal domain, that of PsbC-Glu354. In the apo-PSII structure from *Synechocystis* sp. PCC 6803, the PsbC loop corresponding to that coordinating the cation in the *Synechococcus* 7335 apo-FRL-PSII structure was not resolved nor was it resolved in a recent structure of a *T. vulcanus* assembly intermediate with Psb28 bound (31). However, the loop is resolved in both PSII assembly intermediate structures where Psb27 is bound (27, 28), but in a configuration more similar to what is observed in the mature PSII structures. Our apo-FRL-PSII structure achieved higher global resolution than those structures, but the PsbC(2) loop that participates in cation binding is poorly resolved, suggesting high flexibility. Based on these observations, we suggest that the unique conformation of the cation-binding loop of PsbC(2) observed herein is only achieved when Psb27 is unbound to the PsbC(2) lumenal domain, and may, therefore, represent an unproductive PSII state. This supports the hypothesis that Psb27 stabilizes the lumenal domain of PsbC(2) during PSII assembly (53, 75, 76, 77, 78, 79). As is the case for the acceptor-side perturbations described previously, further data are needed to determine whether the lumenal domain of this core complex is representative of characteristics observed during PSII maturation *in vivo*.

In summary, the apo-FRL-PSII structure from *Synechococcus* 7335 provides important insights into PSII function, especially energy and electron transfer during FaRLiP and donor-side maturation. We have identified the Chl *f*-binding and Chl *d*-binding sites in FRL-PSII from *Synechococcus* 7335. Their positions show that Chl *f* molecules are exclusively used as antenna molecules, which is consistent with previous work (22, 80, 81, 82, 83), and that Chl *d* in the Chl_D1_ site of the ETC serves as the primary electron donor, defining the current lower energy limit for oxygenic photosynthesis for the photosystems that have been discovered so far. It is noteworthy that FRL-PSII exhibits FRL-specific sequence differences that do not appear to be associated with nearby Chl *f*-binding or Chl *d*-binding sites (Fig. 2). This could suggest differences in subunit assembly/stability that are specific to FaRLiP. PsbH2 was lost during sample preparation for cryo-EM, which must be an important aspect of FRL-PSII; therefore, a complete molecular structure of the mature dimeric FRL-PSII complex is desirable. The structure also shows that Psb28 is not required for the replacement of bicarbonate with the PsbD(3)-Glu241 side chain. The significance of this observation should be tested by biophysical and biochemical experimentation, especially in a PsbD(3)-Glu241 point mutant, to understand better the role of bicarbonate in PSII activity.

## Experimental procedures

### Strain and growth conditions

*Synechococcus* 7335 *psbC2::*[His]_6_*::aphAII* strain (hereafter, *Synechococcus* 7335 *psbC2*-[His]_6_) was generated by inserting a sequence encoding a [His]_6_-tag and an *aphAII* gene cassette (conferring kanamycin resistance) at the 3′ end of the *psbC2* gene (9). Cells of the *Synechococcus* 7335 *psbC2*-[His]_6_ strain were grown at room temperature (∼25 °C) in medium ASNIII (84) with the addition of 50 μg kanamycin ml^–1^. Continuous WL was provided by cool fluorescent bulbs (∼45–50 μmol photons m^–2^ s^–1^), and cultures were slowly sparged with 1% (v/v) CO_2_ in air. To grow cells in FRL, liquid cultures were first adapted to red light (∼35–40 μmol photons m^–2^ s^–1^), which was provided by using a red plastic filter as described previously (5, 85). Cultures grown in red light were then diluted to about 0.2 OD at 750 nm to initiate the FRL cultures (9, 14). FRL was provided by a light-emitting diode panel with emission centered at 720 nm and/or by filtering halogen light with a combination of green and red plastic filters to provide FRL at ∼20 to 28 μmol photons m^–2^ s^–1^ (85). For complete acclimation to FRL, cells were grown continuously in FRL by diluting the cultures and refreshing the medium at 2-week intervals. Cells fully adapted to FRL were harvested from liquid cultures grown in FRL for 8 to 12 weeks.

### Isolation of FRL-PSII complexes

Cells of *Synechococcus* 7335 *psbC2*-[His]_6_ grown in FRL were resuspended in MES buffer, which is composed of 50 mM MES, pH = 6.5, 15 mM CaCl_2_, and 10 mM MgCl_2_. Cells were lysed by three passages through a chilled French pressure cell operated at 138 MPa. Unbroken cells and cell debris were removed through centrifugation (4,284*g*). Total membranes were pelleted by ultracentrifugation (126,000*g*, 1 h), resuspended in the photosystem isolation buffer (PIB), which is composed of 50 mM MES, pH = 6.5, 15 mM CaCl_2_ and 10 mM MgCl_2_, 100 mM NaCl, and 5 mM imidazole, and solubilized at 4 °C in the dark for 1 h by addition of β-DM to a final concentration of 1% (w/v). Note that the isolation buffer did not contain betaine, which is commonly added at high concentration to stabilize the OEC and PSII dimers during isolation. After removal of insoluble debris by centrifugation (10,967*g*), the solubilized membranes were loaded onto a pre-equilibrated column for immobilized metal affinity chromatography on columns packed with Ni^2+^–nitrilotriacetate affinity resin equilibrated with PIB (Goldbio). The column was washed with five column volumes of PIB buffer with 15 mM imidazole and 0.03% (w/v) β-DM. FRL-PSII complexes were eluted with the PIB buffer with addition of 100 mM imidazole and 0.03% (w/v) β-DM. The eluate was concentrated with Millipore Centriprep 100 kDa centrifugal Filtration Devices (EMD Millipore) and loaded onto 5 to 20% sucrose gradients, which were prepared with the MES buffer containing 0.03% (w/v) β-DM. Gradients were centrifuged for ∼18 h at 108,000*g*. Two green-colored Chl-containing fractions were collected. Fraction 2 (at higher sucrose concentration; Fig. S1*A*) contained apo-FRL-PSII core complexes. Aliquots of the purified apo-FRL-PSII core complexes were dialyzed against PIB, concentrated, and resuspended in PIB containing 0.03% (w/v) β-DM and 5% (w/v) glycerol for storage at −80 °C.

### Analytical methods

Absorption spectroscopy and the low-temperature fluorescence emission spectroscopy were performed as described previously (85). The protein composition of FRL-PSII fractions was analyzed by SDS-PAGE as previously described (86). Protein compositions of fractions were also evaluated by tryptic peptide fingerprinting by mass spectrometry as previously described (8, 85). Pigments were extracted and analyzed by high-performance liquid chromatography as described previously (85, 87).

### Cryo-EM grid preparation

FRL-PSII was plunge frozen in a dark room under low green light using a Thermo Fisher Vitrobot system. FRL-PSII complexes (3 μl of a solution at ∼3 mg Chl ml^–1^) was applied to a holey-carbon Quantifoil 2/1 Cu 300-mesh electron microscopy grid (Electron Microscopy Sciences) that was glow-discharged for 30 s at 25 mA. The grid was blotted for 3 s and plunged into liquid ethane. It was stored in liquid nitrogen until data collection. The Vitrobot system temperature was 4 °C and set to 100% humidity.

### Cryo-EM data collection

A Titan Krios G2 transmission electron microscope (Thermo Fisher/FEI) was operated at 300 kV with a Gatan K3 direct electron detector. The defocus range was set to −1.0 to −2.0 μm, and the nominal magnification was 105,000×. The super-resolution pixel size was 0.413 Å. The dose rate was 22 e^−^ physical pixel^−1^ s^−1^. The GIF setting was a slit size of 15 eV. The total exposure time was 1.9 s per exposure with a total dose of 40.8 e^−^ (Å) ^−2^. SerialEM was used to collect 5,176 micrograph movies with 50 images per stack.

### Cryo-EM data processing

A flowchart of cryo-EM data processing is shown in Figure S2. All cryo-EM data processing was performed within RELION 3.1 (88). Frames were corrected, aligned, and dose weighted using MotionCor2 (89), and Ctffind-4.1.13 (90) was used to estimate the contrast transfer function (CTF). Initially, a set of ∼1000 manually selected particles was used to create 2D classes for autopicking templates. The initial selection yielded 958,755 particles after deselecting incorrectly chosen particles manually. 2D classification filtered out some bad classes leaving a total of 955,095 particles. These were used to create an *ab initio* model with the InitialModel function. The best of five classes was chosen from 3D classification, yielding 318,567 particles. Rounds of CTF refinement and Bayesian polishing led to a 3D reconstruction at a resolution of 2.40 Å based on the gold-standard Fourier shell correlation (0.143) cutoff criterion (88, 91). Particles were selected based on metadata value CtfMaxResolution ≤5.0, which led to 315,307 particles that reconstructed to a resolution of 2.38 Å. One round of CTF refinement with per-particle astigmatism and detergent micelle subtraction led to a final resolution of 2.25 Å.

### Model building

An initial model was generated by individually creating homology models of each subunit present in the complex (Fig. 1) using the corresponding subunits of the *T. vulcanus* PSII XRD (PDB: 3WU2) (47) structures as templates. These homology models were created using SwissModel (92), and the components were fit into the ESP map using UCSF Chimera (93). The structure was edited using Coot (94) and refined using Phenix real_spce_refine (95, 96). Pigment site numbering used herein corresponds to those first assigned in the high-resolution XRD structure of PSII from Umena *et al.* (47) (Fig. S8).

### Cone scans

Cone scans were performed as described previously (13). In short, each Chl molecule was least-squares aligned to a reference Chl, and the corresponding ESP map was inverted using the program suites CCP4 (97), Rave (98), and Phenix (96). Direct Fourier summation of corresponding structure factors was used to extract rescaled experimental ESP values for each Chl molecule. The experimental ESP values were extracted along the C2 and C7 axis, sampling every 5° at an expected bond length. The values of the C7 axis were used to generate a null distribution modeled by the equation *μ* + 3*σ*, where *μ* is the distribution mean and *σ* is the distribution standard deviation. This describes a methyl moiety with a significance level of 0.002 assuming a normal distribution at each sampled angle.

## Data availability

The cryo-EM structure has been deposited in the PDB and the map into the Electron Microscopy Data Bank with accession codes 7SA3 and EMD-24943, respectively.

## Supporting information

This article contains supporting information (48, 88).

## Conflict of interest

The authors declare that they have no conflicts of interest with the contents of this article.

## Footnotes

† All PsbA(3) residues are numbered one greater in the *Synechococcus* 7335 PsbA3 sequence than they are in the *T. vulcanus* PsbA sequence, but for consistency in comparison with other work, we maintain the numbering of residues from *T. vulcanus* PsbA that are found near the OEC-binding site. For example, we refer to PsbA3-Asp170 and PsbA3-Glu189 as comprising the high-affinity site, but their true positions in the PsbA3 sequence are PsbA3-Asp171 and PsbA3-Glu190.
