# Supplementary material for: Structure of a monomeric photosystem II core complex from a cyanobacterium acclimated to far-red light reveals the functions of chlorophylls d and f
Source: J Biol Chem. 2021 Nov 19;298(1):101424. doi: 10.1016/j.jbc.2021.101424 (PMC8689208; doi:10.1016/j.jbc.2021.101424)
Supplement: Figures S1–S17 and Tables S1–S4 [file mmc1.docx]

**Supporting Information for**

Structure of a monomeric photosystem II core complex from a cyanobacterium acclimated to far-red light reveals the functions of chlorophylls *d* and *f*

Christopher J. Gisriel^1^, Gaozhong Shen^2^, Ming-Yang Ho^2,3,4^, Vasily Kurashov^2^, David A. Flesher^5^, Jimin Wang^5^, William H. Armstrong^6,‡^, John H. Golbeck^2,7^, M. R. Gunner^8^, David J. Vinyard^9^, Richard J. Debus^10^, Gary W. Brudvig^1,5,^*, and Donald A. Bryant^2,3,^*

**Affiliations:**

^1^Department of Chemistry, Yale University, New Haven, CT 06520, USA.

^2^Department of Biochemistry and Molecular Biology, The Pennsylvania State University, University Park, PA 16802, USA.

^3^Intercollege Graduate Program in Plant Biology, The Pennsylvania State University, University Park, PA 16802 USA.

^4^Department of Life Science, National Taiwan University, Taipei 10617, Taiwan.

^5^Department of Molecular Biophysics and Biochemistry, Yale University, New Haven, CT 06520, USA.

^6^Department of Chemistry, Boston College, Chestnut Hill, MA 02467, USA.

^7^Department of Chemistry, The Pennsylvania State University, University Park, PA 16802 USA.

^8^Department of Physics, City College of New York, New York, NY 100031, USA.

^9^Department of Biological Sciences, Louisiana State University, Baton Rouge, LA 70803, USA.

^10^Department of Biochemistry, University of California, Riverside, CA 92521, USA.

^‡^Retired

*To whom correspondence should be addressed: [dab14@psu.edu](mailto:dab14@psu.edu) and [gary.brudvig@yale.edu](mailto:gary.brudvig@yale.edu)

**Supplemental Figures S1 to S17 and Supplemental Tables S1 to S4**

**Figure S1. Isolation and characterization of apo-FRL-PSII from *Synechococcus* 7335.**

**Figure S2. Cryo-EM data processing of *Synechococcus* 7335 apo-FRL-PSII.**

**Figure S3. Map-map and map-model Fourier shell correlations of the cryo-EM data.**

**Figure S4. Local resolution of the apo-FRL-PSII cryo-EM structure from *Synechococcus* 7335.**

**Figure S5. Examples of tetrapyrroles from the ESP map.**

**Figure S6. Location and map of unknown subunit.**

**Figure S7. Pigments missing in apo-FRL-PSII compared to mature PSII from *T. vulcanus*.**

**Figure S8. Chl site nomenclature from the structure of *T. vulcanus* mature PSII.**

**Figure S9. Sequence alignments of FRL- and WL-specific core polypeptides.**

**Figure S10. FRL-specific sequence differences that may interact with FRL-BC.**

**Figure S11. C2 cone scans of proposed Chl *f* sites.**

**Figure S12. Partial sequence alignment of PsbD(3).**

**Figure S13. Orientation of apo-FRL-PSII PsbA3 (D1) C-terminus.**

**Figure S14. Map region corresponding to a cation near the OEC-binding site and comparison with nearby water molecules.**

**Figure S15. Cation bound near the vacant OEC-binding pocket and nearby residue positions.**

**Figure S16. Distances of Chl *f* molecules to Chl *d* in the ETC.**

**Figure S17. Differentiation of PsbF1 and PsbF2 assignment in the apo-FRL-PSII ESP map.**

**Table S1. Identification of subunits in apo-FRL-PSII complexes from *Synechococcus* 7335 based upon tryptic peptide fingerprinting and MS/MS mass spectrometry.**

**Table S2.** **Cryo-EM data collection, refinement, and validation statistics for apo-FRL-PSII from *Synechococcus* 7335 (PDB 7SA3).**

**Table S3. Root-mean-square deviation of α-carbons in core subunits of *Synechococcus* 7335 apo-FRL-PSII with homologous subunits from other cyanobacterial PSII structures.**

**Table S4. Sequence identity of *Synechococcus* 7335 apo-FRL-PSII subunits compared to homologous subunits from other cyanobacterial PSII structures.**

**Supplementary Data 1. Jupyter Notebook for cone scans (external file).**

**Supporting Figures**


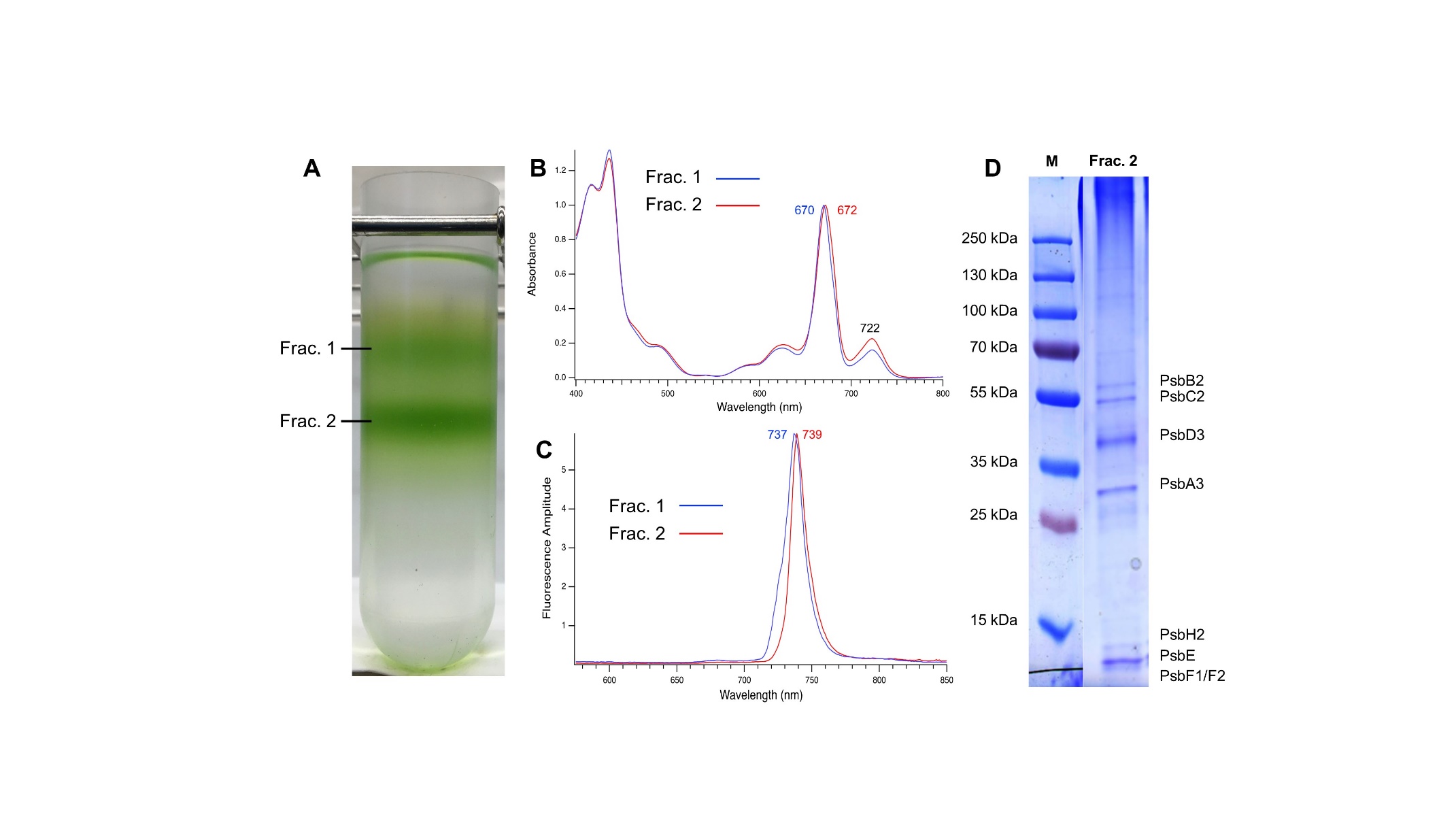


**Figure S1. Isolation and characterization of apo-FRL-PSII from *Synechococcus* 7335. A.** Sucrose gradient centrifugation following isolation of apo-FRL-PSII by immobilized metal affinity chromatography. Two fractions were observed and are labeled. Fraction 2 contained the apo-FRL-PSII complexes characterized in this study. **B.** Absorbance spectra for both fractions at room temperature. Maxima are labeled. **C.** Steady-state fluorescence emission spectra for both fractions at 77 K. Maxima are labeled. **D.** SDS-PAGE of fraction two. The left lane labeled “M” contains molecular mass standards. The right lane labeled “Frac. 2” was loaded with proteins corresponding to 10 µg Chl. Polypeptides assigned to each band are labeled.

**
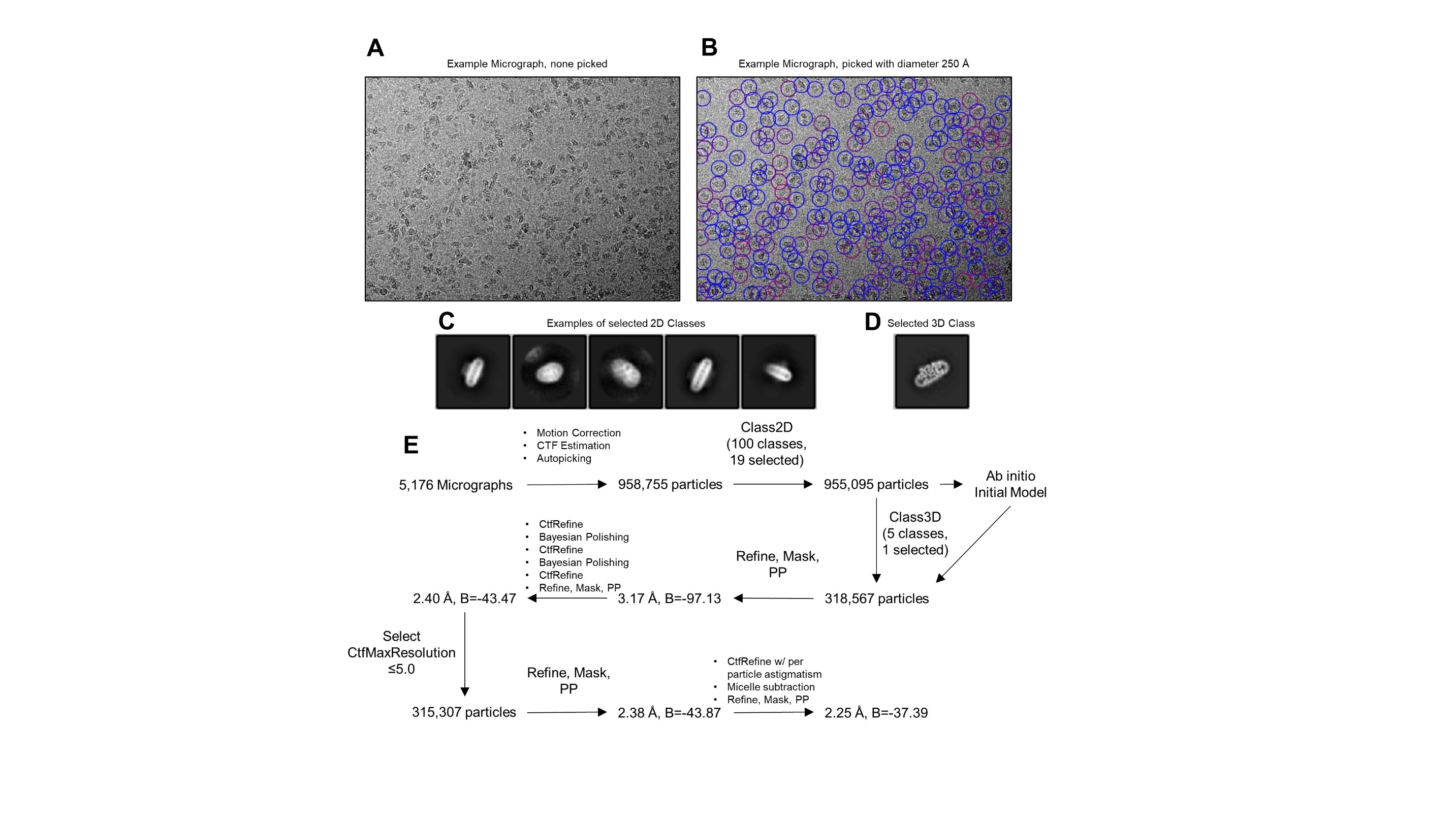
**

**Figure S2. Cryo-EM data processing of *Synechococcus* 7335 apo-FRL-PSII. A.** Example micrograph. **B.** Same as panel A but with particles picked from autopicking. **C.** Examples of 2D classes chosen from the Class2D job. **D.** Slice through the class selected from the Class3D job. **E** Workflow of cryo-EM data processing in RELION 3.1 (91). PP = PostProcess job type. The B-factor determined in postprocessing is listed in units of Å^2^.

**
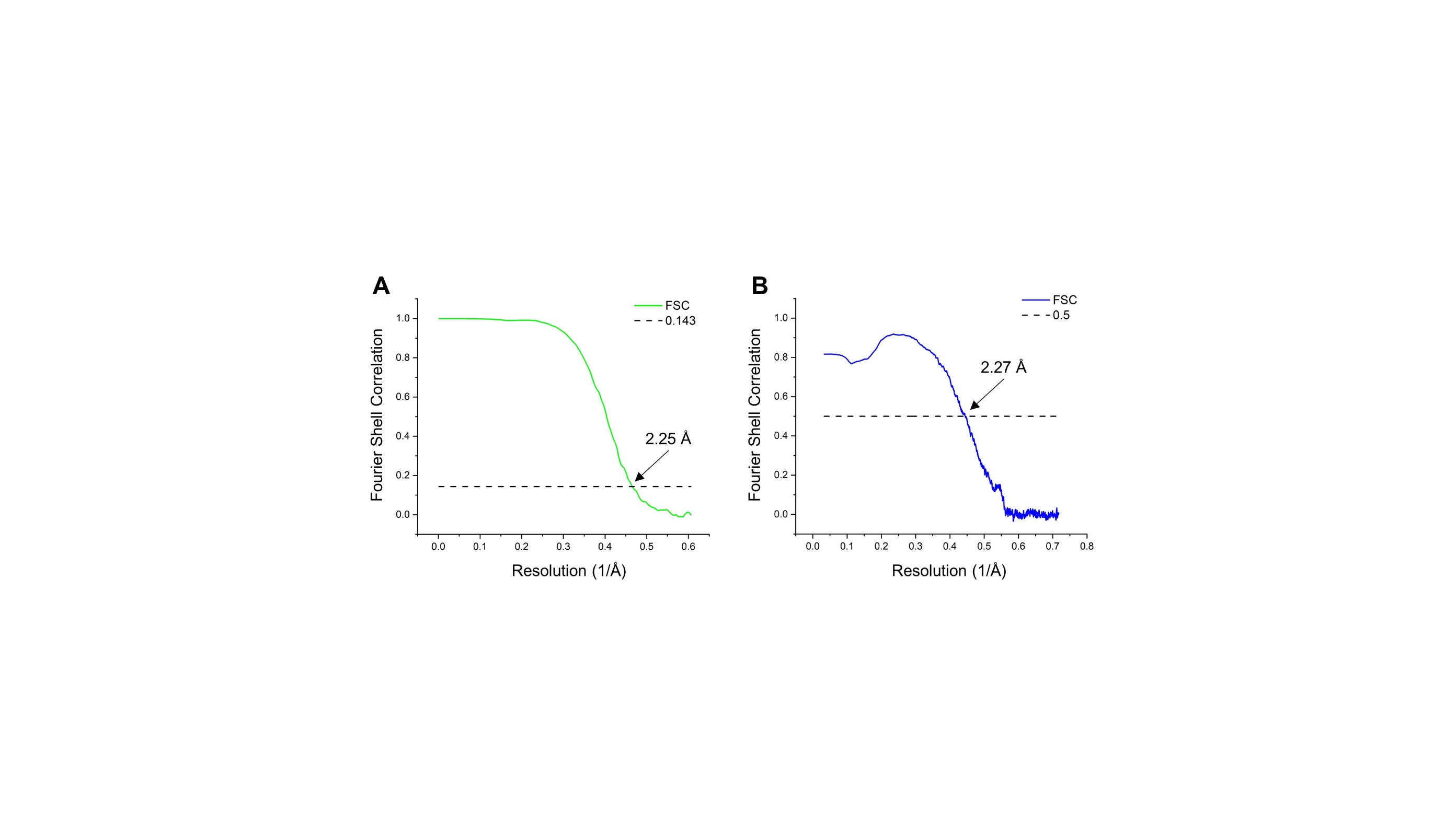
**

**Figure S3. Map-map and map-model Fourier shell correlations of the cryo-EM data. A.** Map-map Fourier shell correlation. The FSC value at 0.143 corresponds to 2.25 Å resolution. **B.** Map-model Fourier shell correlation. The FSC value at 0.5 corresponds to 2.27 Å resolution.

**
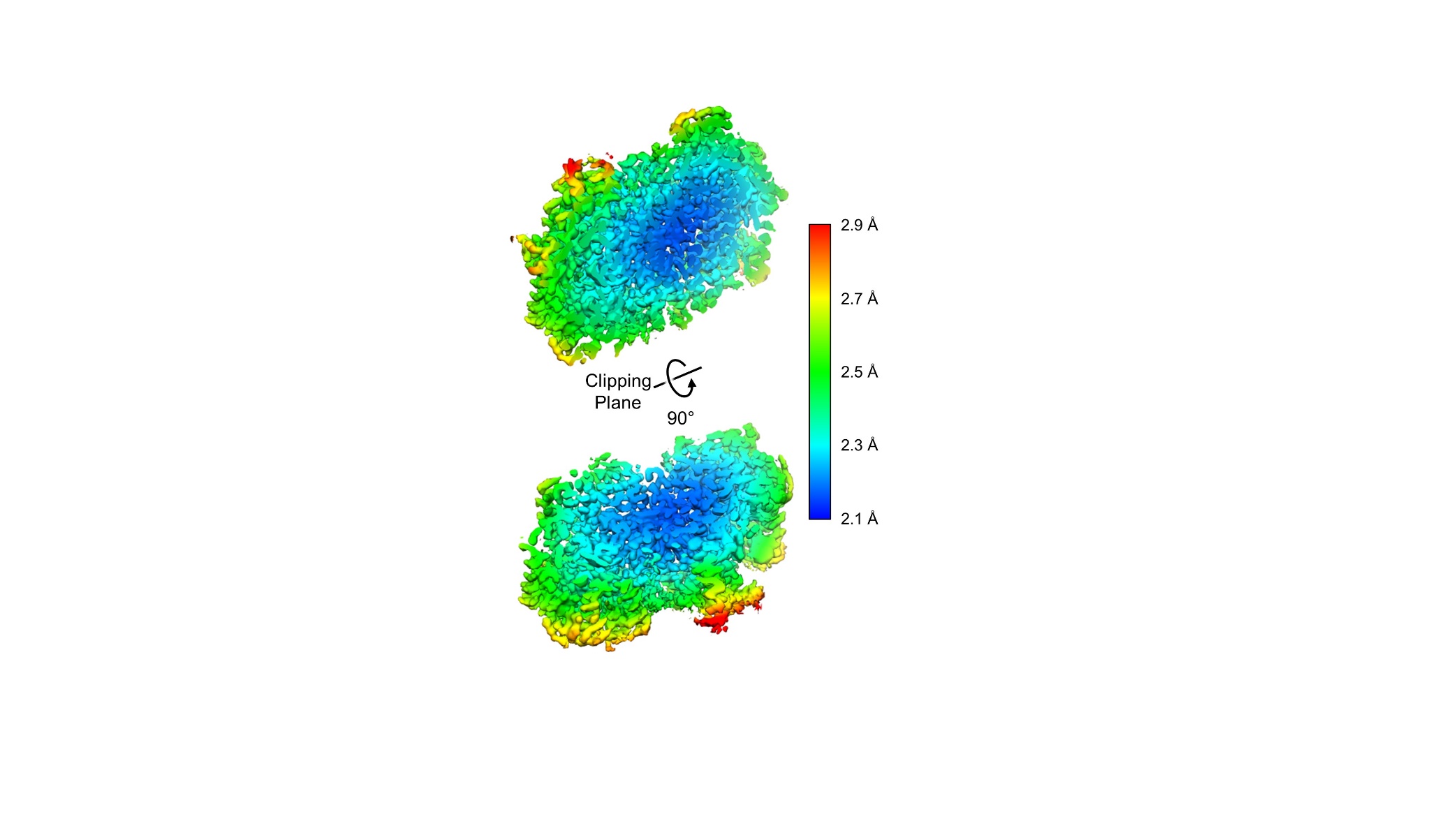
**

**Figure S4. Local resolution of the apo-FRL-PSII cryo-EM structure from *Synechococcus* 7335.** The unsharpened map is shown and colored according to the key. The local resolution map was generated in RELION 3.1 (91).

**
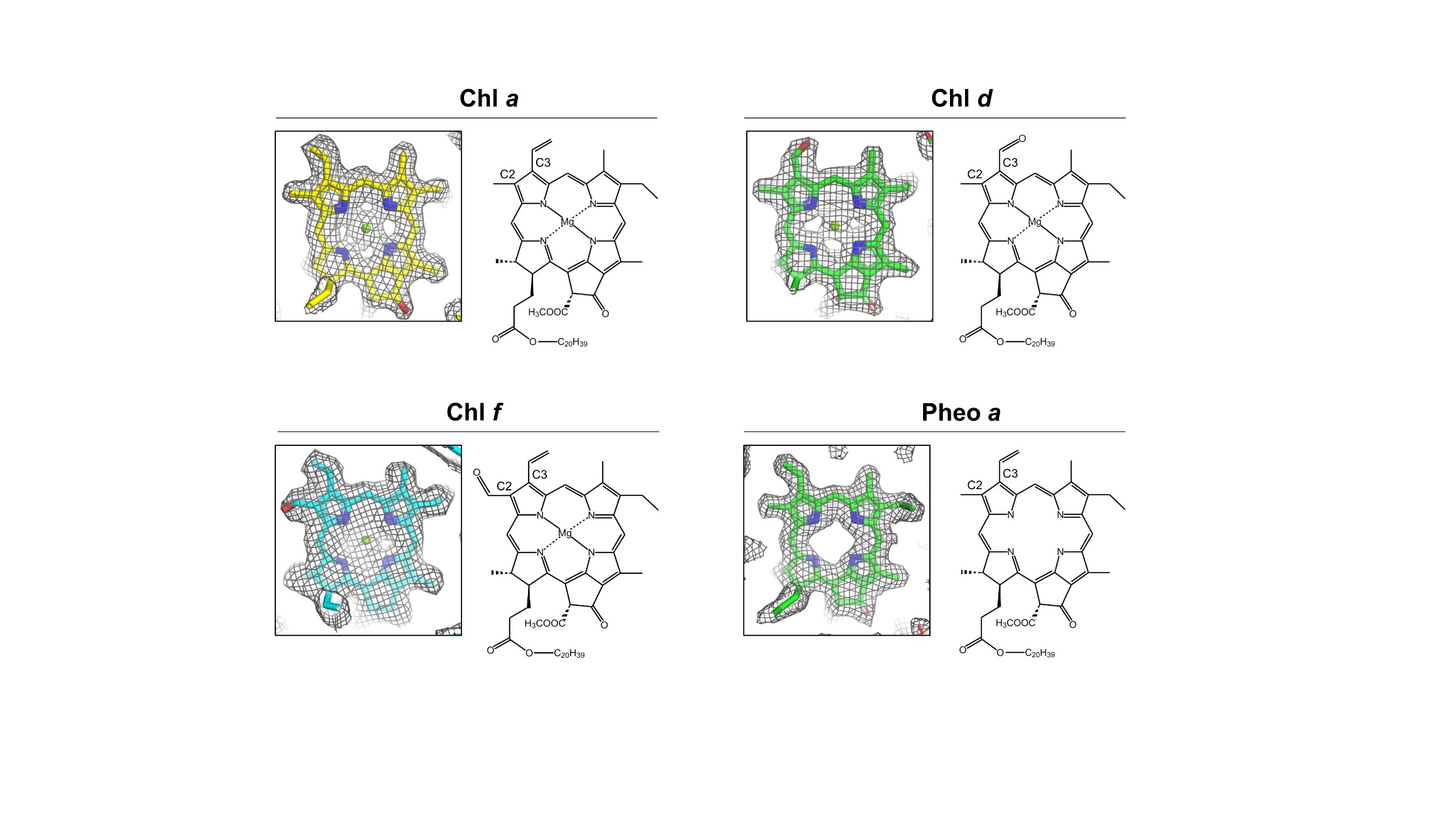
**

**Figure S5. Examples of tetrapyrroles from the ESP map.** The model within the map for an example of each tetrapyrrole is shown, with its chemical structure, labeled accordingly.

**
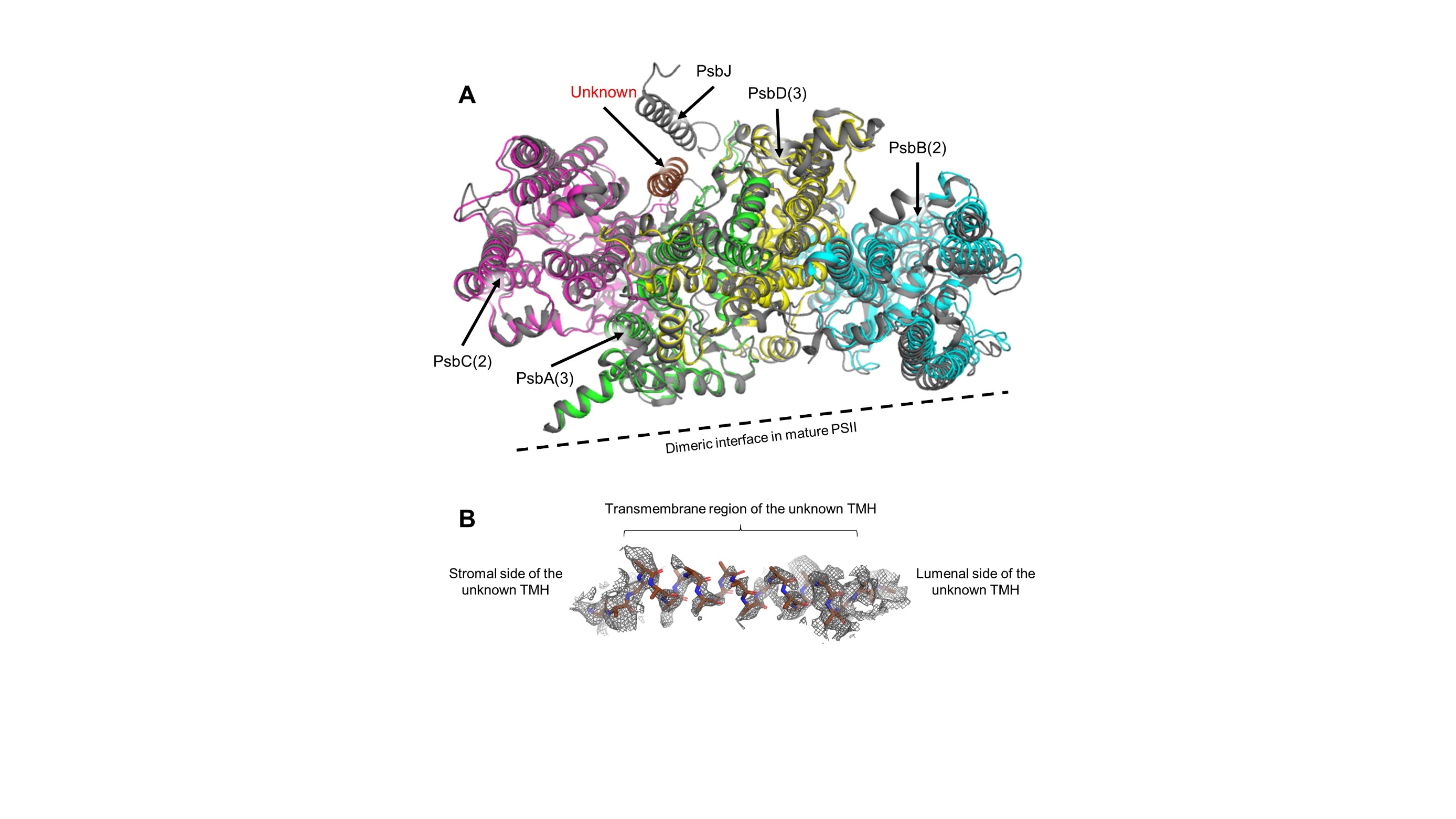
**

**Figure S6. Location and map of unknown subunit.** **A.** Superposition of *Synechococcus* 7335 apo-FRL-PSII with mature PSII from *T. vulcanus* (PDB 3WU2). A stromal view is shown. For *Synechococcus* 7335 apo-FRL-PSII (colors), the core subunits and an unknown transmembrane subunit are shown. *T. vulcanus* PSII (grey), the core subunits and PsbJ are shown. The location of the dimeric interface is shown with a dashed black line. **B.** Unsharpened ESP map and model of the unknown subunit. The map is shown at 6.5σ.

**
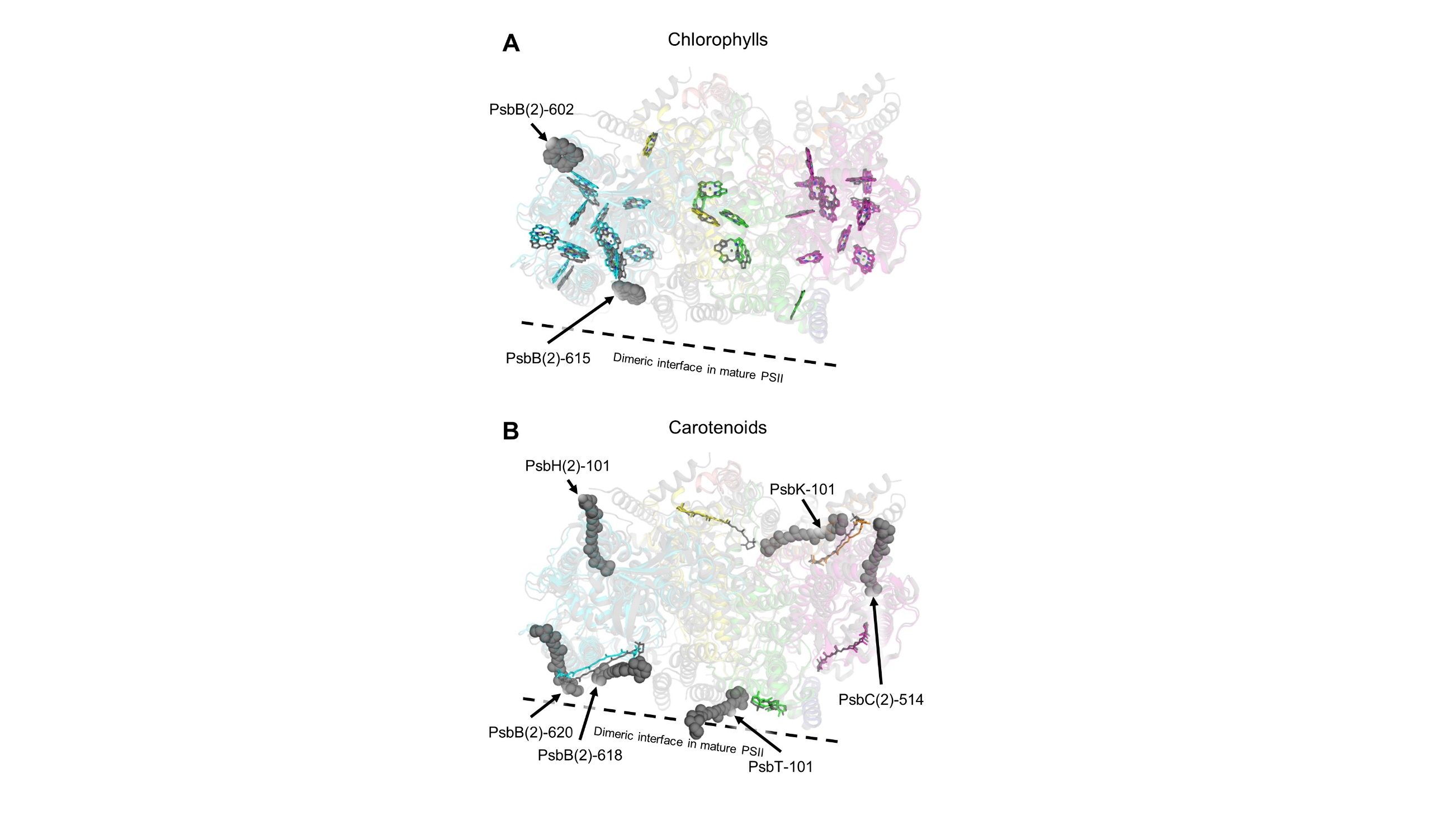
**

**Figure S7. Pigments missing in apo-FRL-PSII compared to mature PSII from *T. vulcanus*.** A superposition of the *Synechococcus* 7335 apo-FRL-PSII structure and *T. vulcanus* mature PSII structure (PDB 3WU2) is shown with transparent cartoons. The view is from the lumenal side of the complex and PsbO, PsbU, and PsbV from the mature PSII structure are hidden for clarity. **A.** Tetrapyrrole rings only are shown. The two Chls not present in the *Synechococcus* 7335 apo-FRL-PSII structure are shown in sphere representation. **B.** Carotenoids only are shown. The six carotenoids not present in the *Synechococcus* 7335 apo-FRL-PSII structure are shown in sphere representation.

**
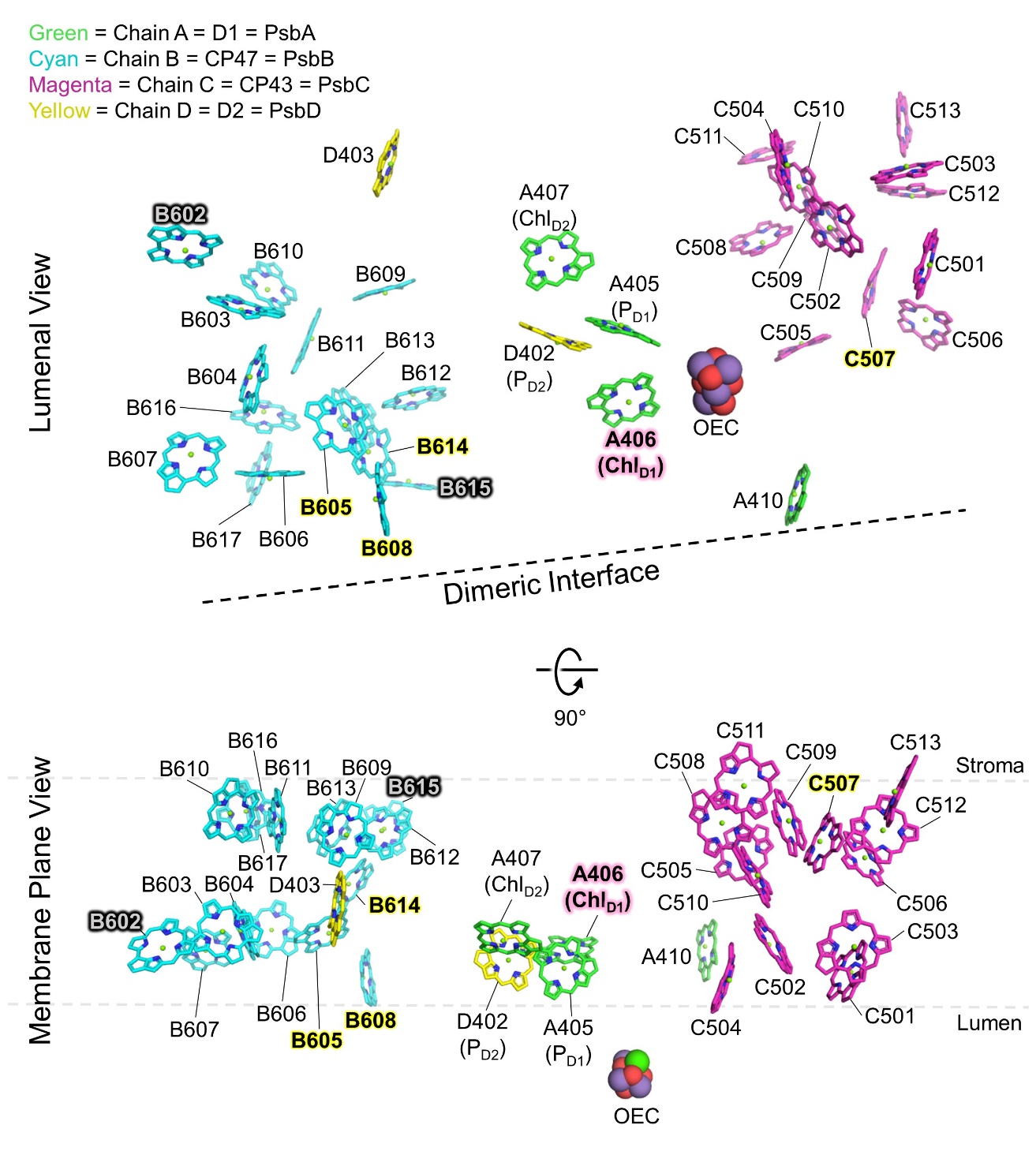
**

**Figure S8. Chl site nomenclature from the structure of *T. vulcanus* mature PSII.** The tetrapyrrole rings of Chl sites and their site names are shown for a monomer of the XRD structure of mature dimeric PSII of *T. vulcanus* (PDB 3WU2). Top shows a lumenal view and bottom shows a membrane plane view. In the top view, the location of the dimerization interface is shown, and in the bottom view, the approximate boundaries of the lipid bilayer are shown by the dashed lines. The OEC is shown in sphere representation in both panels for orientation. Chls assigned to different subunits are colored individually. Note that the first letter of each site label corresponds to its assigned “chain” in the PDB file. The polypeptide corresponding to each chain is also shown in the key. Chl sites assigned as Chl *d* or Chl *f* in the apo-FRL-PSII structure from *Synechococcus* 7335 are labeled bold black font with pink and yellow glows, respectively. Chl sites that are missing in the apo-FRL-PSII structure relative to the mature *T. vulcanus* PSII structure are labeled in bold grey font with a black glow.

PsbA3/PsbA sequences

FRL *S*. 7335 ---MTTISTRPTSRFPTWDRFCNWVTSTENRLYIGWFGVLMLPLLGVSITVFVTAFIAAP 57

FRL *H. hongdechloris* ---MTTTLQ-RPGTIDLWERFCAWVTSTENRLYVGWFGVLMIPLLGVSTAVFVTAFIAAP 56

FRL *F*. 7521 ---MTTISTRPTSRFPTWDRFCNWVTSTENRLYIGWFGVLMIPLLGVSICVFTIAFIAAP 57

WL *S*. 7335 ---MTTTLQ-QRQSASLWEQFCQWVTSTENRLYVGWFGVLMIPTLLAATACFVIAFIAAP 56

WL *S*. 7335 MVSTTTTLQ-RSENASLWEQFCQWVTSTENRLYVGWFGVLMIPTLLAATACFVIAFIAAP 59

WL *S*. 7335 ----------------MWEQFCQWVTSTENRLYVGWFGVLMIPTLLAATACFVIAFIAAP 44

WL *H. hongdechloris* ---MTTTLQ-QQQQQSLWERFCNWITSTENRLYIGWFGVLMIPTLLTATTCFVIAFIAAP 56

WL *H. hongdechloris* ---MTSTLQ-ARESVGAWEQFCRWITSTDNRLYIGWFGVLMVPTLLTATTCFVIAFIGAP 56

WL *H. hongdechloris* ---MTATLQ-RRDSANLWEQFCQWVTSTDNRLYIGWFGVLMLPTLLAATTCFVIAFVAAP 56

WL *F*. 7521 ---MTTTLT-RGESGSLWDRFCEWITSTNNRLYIGWFGVLMIPTLLTATICFIIAFIAAP 56

WL *F*. 7521 ---MTTTLQ-RRASGNVWERFCDWVTSTENRLYIGWFGVLMIPTLLAATTCFIIAFIAAP 56

WL *F*. 7521 ---MTATLQ-RAQSANVWERFCNWITSTENRLYIGWFGVLMIPTLLAATTCFIIAFIAAP 56

*::** *:***:****:*******:* * .: * **:.**

FRL *S*. 7335 PVDIDGIREPLSGSLLYGNNIITAAVVPTSNAIGLHFYPIWEAATLDEWLYNGGPYQMIA 117

FRL *H. hongdechloris* PVDIDGLREPLSGSLLYGNNIITGAVVPSSNAIGLHFYPMWEAATLDEWLYNGGPYQMIA 116

FRL *F*. 7521 PVDIDGIREPVSGSLLYGNNIITAAVVPMSNAIGLHFYPIWEAASMDEWLYNGGPYQMIG 117

WL *S*. 7335 PVDIDGIREPVAGSLMYGNNIISGAVVPSSNAIGLHFYPIWEAASLDEWLYNGGPYQLVI 116

WL *S*. 7335 PVDIDGIREPVAGSLMYGNNIISGAVVPSSNAIGLHFYPIWEAASLDEWLYNGGPYQLVI 119

WL *S*. 7335 PVDIDGIREPVAGSLMYGNNIISGAVVPSSNAIGLHFYPIWEAASLDEWLYNGGPYQLVI 104

WL *H. hongdechloris* PVDIDGIREPVSGSLLYGNNIISGAIVPTSNAVGLHLYPLWQAASLDEWLYNGGPYQLIV 116

WL *H. hongdechloris* AVDIDGIREPVAGSLIYGNNIVSGAVVPSSNAIGLHFYPIWEAASIDEWLYNGGPYQFIV 116

WL *H. hongdechloris* PVDIDGIREPVAGSLMYGNNIISGAVVPSSNAIGLHFYPIWEAASIDEWLYNGGPYQFIV 116

WL *F*. 7521 PVDIDGIREPVSGSLLYGNNIISGAIVPTSNAIGLHFYPIWEASSLDEWLYNGGPYELIV 116

WL *F*. 7521 PVDIDGIREPVAGSLLYGNNIISGAVVPSSNAIGLHFYPIWEAASLDEWLYNGGPYQLVI 116

WL *F*. 7521 PVDIDGIREPVAGSLLYGNNIISGAVVPSSNAIGLHFYPVWEAASLDEWLYNGGPYQLVI 116

*****:***::***:*****::.*:** ***:***:**:*:*:::**********:::

▼ ▼

FRL *S*. 7335 FHYIPALLCYLGREWELSYRLGMRPWICIAYSAPVAATISVFLIYPIGQGSFSDGLPMGI 177

FRL *H. hongdechloris* FHYIPALLCYMGREWELSYRLGMRPWICVAYSAPVAATSSVFLIYPIGQGSFSDGLPMGI 176

FRL *F*. 7521 FHYIPALACYMGREWELSYRLGMRPWIAVAYSAPLAATTSVFLIYPIGQGSFSDGLPMGI 177

WL *S*. 7335 FHFLIGVFCYMGREWELSYRLGMRPWICVAYSAPVAAATAVFLIYPLGQGSFSDGMPLGI 176

WL *S*. 7335 FHFLIGVFCYMGREWELSYRLGMRPWICVAYSAPVAAATAVFLIYPLGQGSFSDGMPLGI 179

WL *S*. 7335 FHFLIGVFCYMGREWELSYRLGMRPWICVAYSAPVAAATAVFLIYPLGQGSFSDGMPLGI 164

WL *H. hongdechloris* FHFLIGIFCWMGRQWEMSYRLGMRPWICVAYSAPVASATAVFLIYPIGQGSFSDGMPLGI 176

WL *H. hongdechloris* FHFLIGIFAYMGRQWELSYRLGMRPWICVAYSAPLSAATAVFLIYPLGQGSFSDGMPLGI 176

WL *H. hongdechloris* FHFLIGVFCYMGREWELSYRLGMRPWICVAYSAPVAAASAVFLIYPLGQGSFSDGMPLGI 176

WL *F*. 7521 FHFLIGIFCWMGRQWELSYRLGMRPWICVAYSAPVAAATSVFLIYPIGQGSFSDGMPLGI 176

WL *F*. 7521 FHFLIGCFCYMGRQWELSYRLGMRPWICVAYSAPLASATAVFLIYPLGQGSFSDGMPLGI 176

WL *F*. 7521 FHFLIGVFCYMGREWELSYRLGMRPWIAVAYSAPVAAATAVFLIYPLGQGSFSDGMPLGI 176

**:: . .::**:**:**********.:*****:::: :******:********:*:**

FRL *S*. 7335 SGTFNFMFVFQAEHNILMHPFHMLGVAGVLGGSLFCAMHGSLVTSSLVRETSDSQSQNEG 237

FRL *H. hongdechloris* SGTFNFMFVFQAEHNILMHPFHMLGVAGVLGGSLFCAMHGSLVTSSLIRETTDSESQNAG 236

FRL *F*. 7521 SGTFNFMFVFQAEHNILMHPLHMIGVAGVLGGSLFCAMHGSLVTSSLIRETTELESQNYG 237

WL *S*. 7335 SGTFNFMLVFQAEHNILMHPFHMLGVAGVFGGSLFSAMHGSLVTSSLVRETTETESQNYG 236

WL *S*. 7335 SGTFNFMLVFQAEHNILMHPFHMLGVAGVFGGSLFSAMHGSLVTSSLVRETTETESQNYG 239

WL *S*. 7335 SGTFNFMLVFQAEHNILMHPFHMLGVAGVFGGSLFSAMHGSLVTSSLVRETTETESQNYG 224

WL *H. hongdechloris* SGTFNFMFVFQAEHNILMHPFHMLGVAGVFGGALFSAMHGSLVTSSLVRETTENESQNYG 236

WL *H. hongdechloris* SGTFNFMFVFQAEHNILMHPFHMLGVAAVFGGSLFSAMHGSLVTSSLVRETTETESQNYG 236

WL *H. hongdechloris* SGTFNFMFVFQAEHNILMHPFHMLGVAGVFGGSLFSAMHGSLVTSSLVRETTETESQNYG 236

WL *F*. 7521 SGTFNFMLVFQAEHNILMHPFHQLGVAGVFGGSLFCAMHGSLVTSSLVRETTENESVNYG 236

WL *F*. 7521 SGTFNFMIVFQAEHNILMHPFHQLGVAGVFGGSLFCAMHGSLVTSSLVRETTETESQNYG 236

WL *F*. 7521 SGTFNFMLVFQAEHNILMHPFHQLGVAGVFGGSLFSAMHGSLVTSSLVRETTETESQNYG 236

*******:************:* :***.*:**:**.***********:***:: :* * *

FRL *S*. 7335 YKFGQEEETYNILAAHGYFGRLIFQYASFNNSRQLHFFLAAWPVVCIWFVALGISTMAFN 297

FRL *H. hongdechloris* YKFGQESETYNILAAHGYFGRLIFQYASFNNSRWLHFFLAAWPVVCIWFVALGISTMAFN 296

FRL *F*. 7521 YKFGQEQETYNIVAAHGYFGRLIFQYASFNNSRSLHFFLAAWPVICIWGTAIGISTMAFN 297

WL *S*. 7335 YKFGQEEETYNIVAAHGYFGRLIFQYASFNNSRSLHFLLGAWPVVGIWFTALGISTMAFN 296

WL *S*. 7335 YKFGQEEETYNIVAAHGYFGRLIFQYASFNNSRSLHFLLGAWPVVGIWFTALGISTMAFN 299

WL *S*. 7335 YKFGQEEETYNIVAAHGYFGRLIFQYASFNNSRSLHFLLGAWPVVGIWFTALGISTMAFN 284

WL *H. hongdechloris* YKFGQEEETYSIVAAHGYFGRLIWQYASFNNSRALHFFLGAWPVIGIWFTALGISTMAFN 296

WL *H. hongdechloris* YKFGQEEETYNIVAAHGYFGRLIFQYASFNNSRSLHFFLGAWPVIGIWFTALGISTMAFN 296

WL *H. hongdechloris* YKFGQEEETYNIVAAHGYFGRLIFQYASFNNSRSLHFFLGAWPVIGIWFTALGISTMAFN 296

WL *F*. 7521 YKFGQEQETYNIVAAHGYFGRLIWQYASFNNSRSLHFFLAAWPVVCIWFTALGISTMAFN 296

WL *F*. 7521 YKFGQEEETYNIVAAHGYFGRLIFQYASFNNSRSLHFFLAAWPVVGIWFTALGISTMAFN 296

WL *F*. 7521 YKFGQEEETYNIVAAHGYFGRLIFQYASFNNSRSLHFFLAAWPVIGIWFTALGISTMAFN 296

******.***.*:**********:********* ***:*.****: ** .*:********

FRL *S*. 7335 LNGFNFNHSVLDSQGRVLPSWADVVNRASLGFEVMHERNAHNFPLDLAS-GESVQVAMRA 356

FRL *H. hongdechloris* LNGFNFNHSVLDSQGRVLPSWADVVNRANIGFEVMHERNAHNFPLDLAS-GPATPVAYQA 355

FRL *F*. 7521 LNGFNFNNSILDSQGRVLPSWADVLNRANLGFEVMHERNAHNFPLDLAG-GEAVPVAITA 356

WL *S*. 7335 LNGFNFNQSIIDSQGRVIGSWADVLNRANLGMEVMHERNAHNFPLDLAA-GEAAPVALTA 355

WL *S*. 7335 LNGFNFNQSIIDSQGRVIGSWADVLNRANLGMEVMHERNAHNFPLDLAA-GEAAPVALTA 358

WL *S*. 7335 LNGFNFNQSIIDSQGRVIGSWADVLNRANLGMEVMHERNAHNFPLDLAA-GEAAPVALTA 343

WL *H. hongdechloris* LNGFNFNQSVLDSQGRVIGTWADVLNRANLGFEVMHERNAHNFPLDLAVEGDAQPVALQA 356

WL *H. hongdechloris* LNGFNFNQSVLDSQGRVIGTWADVLNRANLGMEVMHERNAHNFPLDLAS-DDAAPVALTA 355

WL *H. hongdechloris* LNGFNFNQSVMDSQGRVIGTWADVINRANLGMEVMHERNAHNFPLDLAS-SEAPDIVG-- 353

WL *F*. 7521 LNGFNFNQSILDSHGRIVNTWADILNRANLGMEVMHERNAHNFPLDLAS-GEAVPVAMNA 355

WL *F*. 7521 LNGFNFNQSVIDSQGRVINTWADIINRANLGMEVMHERNAHNFPLDLAS-GDVAPVALTA 355

WL *F*. 7521 LNGFNFNQSVIDSQGRVINTWADIINRANLGMEVMHERNAHNFPLDLAS-GDVAPVALTA 355

*******:*::**:**:: :***::***.:*:**************** . :.

FRL *S*. 7335 PHIGA 361

FRL *H. hongdechloris* PAIHG 360

FRL *F*. 7521 PSITA 361

WL *S*. 7335 PSINA 360

WL *S*. 7335 PSINA 363

WL *S*. 7335 PSINA 348

WL *H. hongdechloris* PAIQG 361

WL *H. hongdechloris* PAINS 360

WL *H. hongdechloris* ----- 353

WL *F*. 7521 PAIHG 360

WL *F*. 7521 PAING 360

WL *F*. 7521 PAING 360

PsbB2/PsbB1 sequences ▼

FRL *S*. 7335 MGLPWYRVHTSVLNDPGRLIAVHIMHNALCAGFAGSMLLFELALFDPSDPVLNPMWRQGC 60

FRL *H. hongdechloris* MGLPWYRVHTVVLNDPGRLLSVHLMHNALCAGFAGSMLLYELALYDPTDPVLNPMWRQGC 60

FRL *F*. 7521 MGLPWYRVHTVVLNDPGRLIAVHLMHNALCAGFAGSMLLFELALYDPSDPVLNPMWRQGC 60

WL *S*. 7335 MGLPWYRVHTVVLNDPGRLISVHLMHTALVAGWAGSMALFELATFDPSDPVLNPMWRQGM 60

WL *H. hongdechloris* MGLPWYRVHTVVLNDPGRLISVHLMHTALVAGWAGSMALFELATYDPSDPVLNPMWRQGM 60

WL *F*. 7521 MGLPWYRVHTVVLNDPGRLISVHLMHTALVAGWAGSMALYELAIYDPSDPVLNPMWRQGM 60

********** ********::**:**.** **:**** *:*** :**:***********

FRL *S*. 7335 FLMPFVSRLGVVNSWQGWSVTGETFTNPGFWTFETVAIAHIIFSGLSFLAACWHWVYWDV 120

FRL *H. hongdechloris* FLMPFVARLGVTNSWQGWSITGESFSDPGFWTFETVAIAHIIFSGLEFLAACWHWVYWDV 120

FRL *F*. 7521 FLMPFVARLGVTNSWQGWSVTGETFADPGFWTFETVAIAHIVFSGLEFLAACWHWFYWDL 120

WL *S*. 7335 FVLPFMTRLGVTGSWGGWDVTGATGISPGFWSFEGVALAHIVLSGLLFLAACWHWVFWDL 120

WL *H. hongdechloris* FVLPFMARLGVTQSWGGWSVTGETAVDPGFWSFEGVAAAHIVLAGLLFLAACWHWVYWDL 120

WL *F*. 7521 FVLPFMARLGVVGSWGGWNVTGAANYDPGFWSFEGVAAAHIVLSGLLFLAAVWHWVYWDL 120

*::**::****. ** **.:** : .****:** ** ***:::** **** ***.:**:

FRL *S*. 7335 ATFFDPKTDEPVIDLPKVFGIHLTLAGILCFGFGAFHLTGLFGPGMWVSDPLGLTGHIQG 180

FRL *H. hongdechloris* ATFFDPKTDEPVLDLPKVFGIHLLLAGLICFGFGAFHQTGIFGPGMWVSDPFGLTGHVQG 180

FRL *F*. 7521 ATFFDSKTGEPTLDLPKIFGIHLFLAGLLCFGFGAFHLTGIFGPGMWVSDPYGLTGHVQG 180

WL *S*. 7335 ELFRDPRTGEPALDLPKMFGIHLFLSGLLCFGFGAFHLTGLWGPGMWVSDPYGLTGHVQG 180

WL *H. hongdechloris* DLFQDPRTGEPALDLPKMFGIHLFLSGLLCFGFGAFHLTGLWGPGMWVSDPYGLTGHIQP 180

WL *F*. 7521 ELFQDPRTGEPALDLPKMFGIHLFLSGLLCFGFGAFHLTGLWGPGMWVSDAYGLTGHIAP 180

* * :*.**.:****:***** *:*::******** **::******** *****:

FRL *S*. 7335 VAPEWGAAGFDPHNPGGVVAHHIALGIVAIIGGLFHIFVRPPEYLYKGLRMGNIEGTLAS 240

FRL *H. hongdechloris* VAPDWGPAGFNPQNPGGVVAHHIALGIVAIIGGLFHISVRPPEYLYRGLRMGNIESVLAS 240

FRL *F*. 7521 VAPVWGPEGFNPQNPGGVVAHHIAAGIVGIIGGLFHIVVRPPEVLYRGLRMGNIETVLAS 240

WL *S*. 7335 VAPEWGPAGFNPFNPGGVVAHHIAAGIVGIVAGLFHLTVRPPQRLYKALRMGNIETVLSS 240

WL *H. hongdechloris* VAPEWGAAGFNPFNPGGIVAHHIAAGIVGIIAGLFHLTVRPPQRLYKALRMGNIETVLSS 240

WL *F*. 7521 VAPEWGPDGFNPFNPGGVVAHHIAAGIVGIIAGLFHLSVRPPERLYKALRMGNIETVLSS 240

*** ** **:* ****:****** ***.*:.****: ****: **:.******* .*:*

FRL *S*. 7335 GLAVFFSGAFIAAGTMWYGTATTPIELWGPTRYQWDQGFFQQAISRQVKASISDGKSPSE 300

FRL *H. hongdechloris* SLAVFFGAGFIAAGTMWYGTATTPIELWGPTRYQWDQGYFQTEINRRVETAIDEGKSRSQ 300

FRL *F*. 7521 ALATFFFAGFVAAGSMWYGTATTPIELWGPTRYQWDQNYFKQEIDRRVQAGLDEGKTLSQ 300

WL *S*. 7335 SIAAVFFAAFIVAGTMWYGSATTPIELFGPTRYQWDGSYFAEEIDRRVQRDIANGASEED 300

WL *H. hongdechloris* SIAAVFFAAFVVAGTMWYGSTATPIELFGPTRYQWDSGYFQQEIERRVQAEVADGASLEE 300

WL *F*. 7521 SIAAVFFAAFVVAGTMWYGSATTPIELFGPTRYQWDQGYFKQEIQRRVQASLASGANLSE 300

.:*..* ..*:.**:****:::*****:******** .:* *.*:*: : .* . .:

FRL *S*. 7335 AWSEIPTKLAFYDYIGNSPAKGGLFRVGRMVDGDGLPTGWLGHPVFKDGEGRELTVRRMP 360

FRL *H. hongdechloris* AWSSIPAKLAFYDYVGNSPAKGGLFRVGRMVDGDGLPTRWLGHPVFKDKEGRELSVRRMP 360

FRL *F*. 7521 SWSAIPEKLAFYDYIGNNPAKGGLFRVGRMVDGDGVAQSWLGHPVFKDREGRELTVRRMP 360

WL *S*. 7335 AYAAIPEKLAFYDYVGNSPAKGGLFRVGPMNTGDGIATAWLGHPVFHDGDGRELTVRRLP 360

WL *H. hongdechloris* AYNAIPEKLAFYDYVGNSPSKGGLFRVGPMNQGDGLAQAWLGHPVFKDGEGRILSVRRIP 360

WL *F*. 7521 AWSQIPEKLAFYDYVGNSPAKGGLFRTGPMVKGDGIAQSWDGHPVFKDAEGRELEVRRLP 360

:: ** *******:**.*:******.* * ***: * *****:* :** * ***:*

FRL *S*. 7335 NFFENFPVVLFDQDGIVRADIPFRQAESKYGIEQTGVTVSFYGGELDGQTFSDPKDVKKY 420

FRL *H. hongdechloris* NFFENFPVLLTDRDGIVRADIPFRRTEAKYSLAQMGVTVSFFGGDLDGQTFTDLEKVRQY 420

FRL *F*. 7521 TFFETFPVVLTDKDGVVRADIPFQRAEAKYSFEQTGVNVSFFGGILDGQTFTDPMTVKKY 420

WL *S*. 7335 NFFETFPVVLVDKDDNLRADIPFRRAESKYSFEQTGVVVDFYGGQLDGQHITDPAAVKRY 420

WL *H. hongdechloris* NFFETFPVVLTDQDGVVRADIPFRRAESRYSFEQKGVTVSFYGGELGGQTFTDPAVVKRY 420

WL *F*. 7521 NFFETFPVILTDKDGIVRADIPFRRAESQNSFEQTGVTVSFYGGNLDGQTFTDPADVKKW 420

.***.***:* *:*. :******:::*:: .: * ** *.*:** *.** ::* *:::

▼ ▼

FRL *S*. 7335 ARRAQLGEPFEFDRSVYDSDGLFRTSNRGFFAFFHVIFGLLWFFGHIWHGLRALFQDVFS 480

FRL *H. hongdechloris* ARKAQLGEPFDFDRTVYDSDGTFRTSNRGFFAFFHVCFALVWFFGHIWHGLRALFQDVFS 480

FRL *F*. 7521 ARQAQLGEPFKFDRTIHNSDGVFRTSNRGFFAFFHTCFALVWFFGHIWHGSRTIYRDVFA 480

WL *S*. 7335 ARKAQLGEPFSFDRETLDSDGVFRSSPRGWFTYGHAVFALLFFFGHIWHGARTLFRDVFA 480

WL *H. hongdechloris* ARKTQLGEPFEFDRETLGSDGVFRTSPRGWFTYAHAVFALIFFFGHIWHGSRTLFRDVFA 480

WL *F*. 7521 ARKAQLGEVFEFDRETLNSDGVFRTSPRGWFTFGHACFALLFFFGHIWHGARTIYRDVFA 480

**::**** *.*** .*** **:* **:*:: *. *.*::******** *::::***:

FRL *S*. 7335 GIDPSLSAEQVEWGYFKKVGDPTSQQTPA--- 509

FRL *H. hongdechloris* GIDPRLDVEQIEWGYFQKVGDPTSRGEPTTSI 512

FRL *F*. 7521 GIDPELD-EQVEFGVFQKVGDTTTRKKQPVI- 510

WL *S*. 7335 GVDPDLSPQQVEWGFYQKVGDFSTKASK---- 508

WL *H. hongdechloris* GIDPDLSPEQVEWGFFAKVGDVSTRKEETV-- 510

WL *F*. 7521 GIDPDLE-EQVEFGVFAKVGDLSTRKKEAV-- 509

*:** *. :*:*:* : **** :::

PsbC2/PsbC1 sequences

FRL *S*. 7335 METPLE-------TIPDLSLSPTAEVGSILAPASPGYDEATSGYAWWAGNARLITPELTG 53

FRL *H. hongdechloris* METPIEPTASALDNISLNTLTPVVDEKDRMTPPKAGYDEESSGYAWWAGNARLITTELSG 60

FRL *F*. 7521 METPFDSKVSKP-------KDEVQKPAYIVNSPSEGRDEASTGYAWWAGNARFIN--QSG 51

WL *S*. 7335 -----------------------MVTLSNNSFVGGGRDQPSTGYAWWSGNARLID--LSG 35

WL *H. hongdechloris* -----------------------MVTLSS-NTFITGRDQESTGFAWWAGNARLIN--LSG 34

WL *F*. 7521 -----------------------MVTLSRPGVLGAGRDQDSTGFAWWAGNARLIN--LSG 35

* *: ::*:***:****:* :*

FRL *S*. 7335 RFLGAHVAHAGLVALWAGGMLLFEVSHFNLSKPMYEQGCILMPHIATLGIGVGQSGEITS 113

FRL *H. hongdechloris* RFLGAHIAHAALIAFWAGGMLLFEVAHYEPTRSMYEQGCILMAHVATQGFGVGYGGEVTD 120

FRL *F*. 7521 RFLGAHVAHAGLIAFWAGAMLLFEVAHYVPEKPMYDQGLILMPHIAALGFGVGPGGQVVD 111

WL *S*. 7335 KLLGAHVAHAGLIVLWTGAMTLFEVSHYIPEKPMYEQGCILLPHLATLGWGVGPGGEVIN 95

WL *H. hongdechloris* KLLGAHVAHAGLIVFWAGAMTLFEVAHFIPEKPLYEQGFILIPHLATLGWGVGPGGEIID 94

WL *F*. 7521 KLLGAHVAHAGLIVFWAGAMTLFEVAHFVPEKPLYEQGLILLPHLATLGWGVGAGGEVVD 95

::****:***.*:.:*:*.* ****:*: : :*:** **: *:*: * *** .*:: .

FRL *S*. 7335 MFPFFAIGVAHLIGSAVLGIGGMYHAIKGPEKL---YGFFQFDWTDRAKVAQILGFHIAI 170

FRL *H. hongdechloris* IFPFFAIGVAHLIGSAVLGFGGVYHALRGPEKL---SGFFDFDWGDRAKVAQILGFHILV 177

FRL *F*. 7521 IFPFFAIAVAHLIGSAVLGFGGIYHSLKGPQKL---PGFFNFDWSDKDKVTSILGYHLIA 168

WL *S*. 7335 LFPYFVVGVLHLVSSAVLGLGGVYHALRGPETLEEYSSFFSQDWKDKNQMTNIIGYHLIL 155

WL *H. hongdechloris* TFPYFVVGVLHLVSSAVLGLGGIYHAVRGPETLEEYSAFFGYDWKDKNKMTTIIGIHLVL 154

WL *F*. 7521 TYPYFVIGVLHLISSAVLGFGGIYHAIRGPEVLEEYSSFFGYDWKDKNKMTNIIGFHLII 155

:*:*.:.* **:.*****:**:**:::**: * .** ** *: ::: *:* *:

FRL *S*. 7335 LGIFALLFAAKAMYWGGLYDPWAPGGGDVRLVTNPTLDPRIIFGYLIKRPTGGEGWIVSV 230

FRL *H. hongdechloris* LGLFALLFVGKAMFWGGLYDPWAPGGGDVRLITNPTLDPRVIFGYLFKNPLGGGGWLVSV 237

FRL *F*. 7521 LGVAAFLLVGKAMLWGGLYDTWAPGGGGVRLVTNPTLDPRVIFGYLFKGFTGGAGNIASV 228

WL *S*. 7335 LGLGAFLLVIKACFLGGVYDTWAPGGGDVRVITNPTLNPGVIFGYLASSPFGGEGWIVGV 215

WL *H. hongdechloris* LGCGALLLVLKACVFGGVYDSWAPGGGDVRIITNPTLNPAKILGYLTDTPFGGKGWIIGV 214

WL *F*. 7521 LGLGAFLLVLKAMFFGGVYDTWAPGGGDVRVITNPTLNPAVIFGYLLKSPFGGDGWIIGV 215

** *:*:. ** **:** ******.**::*****:* *:*** . ** * : .*

▼

FRL *S*. 7335 NNLEDIIGGHIWIGCILIAGGIWHILVPPLRWTYNLFPWTGETYLSQSLGNVAGQAFIAA 290

FRL *H. hongdechloris* DNLEDVVGGHIWIGSLLVIGGLWHILVPPLRWTYNLYPWSGETYLAQSLGNISGQAFIAT 297

FRL *F*. 7521 DNLEDLVGGHIWIGSLLILGGIWHIVTKPFKWTHKAFIWSGEAYLSQSLGNVAGQAFIAT 288

WL *S*. 7335 NNMEDIIGGHIWIGLICIFGGVFHILTKPFGWARRALIWNGEAYLSYSIGAVSLMAFICS 275

WL *H. hongdechloris* DNMEDIIGGHIWIALICIAGGVWHILTKPFGWARRALIWSGEAYLSYSLGALSLMGFIAA 274

WL *F*. 7521 DNMEDIIGGHIWVGLICIFGGIFHILTKPFGWARRAFIWSGEAYLSYSLGALSLMGFIAS 275

:*:**::*****:. : : **::**:. *: *: . *.**:**: *:* :: .**.:

FRL *S*. 7335 AFIWFNNTAYPSVFYGPTVPESSQAQSFVFLMRDQGMGADVASAQGPTGLGKYLQRSPTG 350

FRL *H. hongdechloris* AFIWFNNTVYPSVFYGPTIPEASQAQSFVFLVRDQSLGADVASAQGPTGLGKYLQRSPTG 357

FRL *F*. 7521 MFIWFNNTAYPSEFYGPTVAESSNAQALVFLVRDQNLGANVASAQGPTGLGKYLMRSPTG 348

WL *S*. 7335 CYVWFNNTAYPSEFYGPTNAEASQAQAMTFLVRDQRLGANIGSAQGPTGLGKYLMRSPTG 335

WL *H. hongdechloris* FYVWFNNTAYPSEFYGPTNAEASQAQAFTFLVRDMRLGANIGAAQGPTGLGKYLMRSPTG 334

WL *F*. 7521 CFVWFNNTAYPSEFYGPTNAEASQAQSFIFLVRDQKLGANVASAQGPTGLGKYLMRSPSG 335

::*****.*** ***** *:*:**:: **:** :**::.:*********** ***:*

FRL *S*. 7335 EIIFGGETMRFWDARAPWLEPLRGKNGLDLDKLQHDVQPWQLRRAAEYMTHSPIGSLNSV 410

FRL *H. hongdechloris* EIIFGGETMRFWDAKAPWLEPLRGTNGLDLEKIRNDIQPWQVRRSAEYMTHAPLGSINSV 417

FRL *F*. 7521 EIIFGGETMRFWDVKAPWLEPLRGPNGLDIDKLQHDVQPWQIRRASEYMTHAPIGSLNSV 408

WL *S*. 7335 EIIFGGETMRFWDFDGPWLAPLRGTNGLDLDKLKNDIQPWQVRRAAEYMTHAPNASINSV 395

WL *H. hongdechloris* EIIFGGETMRFWDFKGPWLEPLRGPNGLDLDKIQNDVQNWQVRRAAEYMTHAPNASINSV 394

WL *F*. 7521 EIIFGGETMRFWDFRGPWLEPLRGPNGLDLDKIKNDVQPWQIRRASEYMTHAPNGSINSV 395

************* .*** **** ****::*:::*:* **:**::*****:* .*:***

FRL *S*. 7335 AGLATESNAFNYVSPRTWLASAHFIFGFFFLVGHLWHAGRARAAAAGFETGLDREDEPVL 470

FRL *H. hongdechloris* AGLATEVNSFNYVSPRTWLASAHFILAFFFLVGHLWHAGLARATAAGFVTGLDRENEPVL 477

FRL *F*. 7521 GGLATELNSFNFVGPRAWLASAHFVFALLFLVGHLWHAGRARAAAAGFERGIDREDEPVL 468

WL *S*. 7335 GGIITEVNSVNFVNPRQWLASFHFVMAFFFLVGHLWHAGRARAAEGGFERGLNREAEPVL 455

WL *H. hongdechloris* GGIITEINSVNFVNPRQWLATSHFVLAFFFLVGHLWHAGRARASAAGFEKGIERETEPVL 454

WL *F*. 7521 GGVITEPNSFNYVNPRAWLATSHFVLAFFFLVGHLWHAGRARAAAGGFEKGIDRETEPVL 455

.*: ** *:.*:*.** ***: **::.::********** ***: .** *::** ****

FRL *S*. 7335 SMAPIDPSLRSD 482

FRL *H. hongdechloris* SMPPIDPSERST 489

FRL *F*. 7521 SMPPLD------ 474

WL *S*. 7335 SMPDLD------ 461

WL *H. hongdechloris* AMGDID------ 460

WL *F*. 7521 FMKDLD------ 461

* :*

PsbD3/PsbD1 sequences

FRL *S*. 7335 MTITMGSL-GSARDWIKQLDDWLKRDRFVFIGWSGLLLFPCSFLAIGAWFTGTTFVTSWY 59

FRL *H. hongdechloris* MTTTIGGL-KTTQNWVSVLDDWLKRDRFVFIGWSGLLLFPCAYLSIGAWFTGTTFVTSWY 59

FRL *F*. 7521 MTIAIRPSRTRGFEWFYVLDDWLKRDRFVFIGWSGLLLFPCAYLAIGGWFTGTTFVTSWY 60

WL *S*. 7335 MTIAMGQA-PAARGRFDVLDDWLKRDRFVFVGWSGLLLFPCAFLAIGGWMTGTTFVTSWY 59

WL *H. hongdechloris* MTIAMGRA-QAQRGWFDVLDDWLKRDRFVFIGWSGLLLFPCAYLAIGGWLTGTTFVTSWY 59

WL *F*. 7521 MTIAIGR--STTRGWFDVLDDWLKRDRFVFVGWSGLLLFPCAYLALGGWLTGTTFVTSWY 58

** :: . ************:**********::*::*.*:**********

FRL *S*. 7335 THGLVSSYLEGCNFLTVAVSTPAESMGHSLLLLWGPEASGDFVRWCQIGGLWTFTALHGV 119

FRL *H. hongdechloris* THGLVSSYLEGCNFLTVAVSSPAESMGHSLLLLWGPEANWDFVRWCQIGGLWNFTAFHGV 119

FRL *F*. 7521 THGIVSSYLEGCNFLTAAVSTPADSMGHSLLFLWGPEAQGDFTRWCQIGGLWNFVALHGV 120

WL *S*. 7335 THGLASSYLEGCNFLTVAVSTPADSMGHSLLLLWGPEAQGDFVRWCQIGGLWAFVALHGA 119

WL *H. hongdechloris* THGLASSYLEGCNFLTVAISTPPDSMGHSLLLLWGPEAQGDFVRWCQIGGLWTFTALHGA 119

WL *F*. 7521 THGLASSYLEGCNFLTVAVSTPADAMGHSLLLLWGPEAQGDFTRWCQLGGLWTFVALHGA 118

***:.***********.*:*:* ::******:******. **.****:**** *.*:**.

FRL *S*. 7335 FGLIGFMLRQIEIARLVGIRPYNAIAFSAPIAVYCATFLIYPLGQSSWFFGPGFGVSAIF 179

FRL *H. hongdechloris* LGLMGFMLRQIEIARLVGVRPYNAIAFSAPIAVYVSVFLIYPLGQSNWFFAPSFGVAGIF 179

FRL *F*. 7521 FGLIGFMLRQFEIARAVGVRPYNAIAFSAPISVFVSVFLIYPLGQSSWFFAPSFGVAAIF 180

WL *S*. 7335 FGLIGFMLRQFEISRLVGIRPYNAIAFSGPIAVFVSVFLMYPLGQSSWFFAPSFGVAAIF 179

WL *H. hongdechloris* FGLIGFMLRQFEVARLVGLRPYNAIAFSAPIAVFVSVFLMYPLGQSSWFFAPSFGVAAIF 179

WL *F*. 7521 FGLIGFMLRQFEIARLVGIRPYNAIAFSAPIAVFVSVFLMYPLGQSSWFFAPSFGVAAIF 178

:**:******:*::* **:*********.**:*: :.**:******.***.*.***:.**

FRL *S*. 7335 RFLLFFQGFHNYTLNPFHMMGVTGVLGGALLCAIHGATVQNTLFRDNQSKNTFKGFSTDQ 239

FRL *H. hongdechloris* RFLLFFQGFHNYTLNPFHMMGVTGVLGAALLCAIHGATVQNTLFRDTESKNTFKGFSTTQ 239

FRL *F*. 7521 RFLLFFQAFHNYTLNPFHMMGVAGVLGGALLCAIHGATVENTLFRDTKSFNTFGGFSPTQ 240

WL *S*. 7335 RFLLFLQGFHNWTLNPFHMMGVAGILGGALLCAIHGATVENTLFQDDENANTFRAFEPTQ 239

WL *H. hongdechloris* RFLLFFQGFHNWTLNPFHMMGVAGVLGGALLCAIHGATVENTLFKDGENANTFRAFEPTQ 239

WL *F*. 7521 RFLLFLQGFHNWTLNPFHMMGVAGVLGGALLCAIHGATVENTLFEDGEGANTFRAFNPTQ 238

*****:*.***:**********:*:**.***********:****.* :. *** .*. *

FRL *S*. 7335 GEETYSMVTANRFWSQIFGIAFSNKRWLHFFMLFVPVTGLWMSAIGMAGLAFNLRAYDFV 299

FRL *H. hongdechloris* AEETYSMVTANRFWSQIFGIAFSNKRWLHFFMLFVPVTGLWMSAIGMIGLSFNLRAYDFV 299

FRL *F*. 7521 AEETYSFVTANRYWSQIFGIAFSNKRWLHFFMLFVPVTGLWMSSIGMVGLAFNLRAYDFV 300

WL *S*. 7335 SEETYSMVTANRFWSQIFGIAFSNKRWLHFFMLFVPVTGLWMSSIGIVGLALNLRAYDFV 299

WL *H. hongdechloris* AEETYSMVTANRFWSQIFGIAFSNKRWLHFFMLFVPVTGLWMASVGVVGLAVNLRAYDFV 299

WL *F*. 7521 AEETYSMVTANRFWSQIFGIAFSNKRWLHFFMLFVPVTGLWMSAIGIVGLALNLRAYDFV 298

.*****:*****:*****************************:::*: **:.********

FRL *S*. 7335 SQEIRAAEDPEFETFYTKNILLNEGLRAWLSEMDQPAKKFVFPDEVLPRGFSE 352

FRL *H. hongdechloris* SQEIRAAEDPEFETFYTKNILLNEGLRAWMAELDQPAKKFVFPEEVLPRGFGE 352

FRL *F*. 7521 SQEIRAAEDPEFETFYTKNILLNEGARAWMAAQDQPHEKFQFPEEVMPRGNAE 353

WL *S*. 7335 SQEIRAAEDPEFETFYTKNILLNEGIRAWLSPQDQPHEKFVFPEEVLPRGNAL 352

WL *H. hongdechloris* SQEIRAAEDPEFETFYTKNILLNEGIRAWMAPTDQPHQKFVFPEEVLPRGNAL 352

WL *F*. 7521 SQELRAAEDPEFETFYTKNILLNEGIRAWMAPQDQPHEKFVFPEEVLPRGNAL 351

***:********************* ***:: *** :** **:**:*** .

**Figure S9. Sequence alignments of FRL- and WL-specific core polypeptides.** Sequences that are FRL-specific are colored in red font. Unmodeled residues in the reported structure are highlighted in grey. Residues that appear to be FRL-specific are highlighted in green. Residues that confer specificity for binding Chl *f* or Chl *d* near the C2 formyl moiety of Chl *f* or the C3 formyl moiety of Chl *d* sites are indicated by a red arrowhead. The sequence alignments were performed using the Clustal Omega server (48). Abbreviations: *S*. 7335, *Synechococcus* sp. PCC 7335; *H*. *hongdechloris*, *Halomicronema hongdechloris*; *F*. 7521, *Fischerella thermalis* PCC 7521.


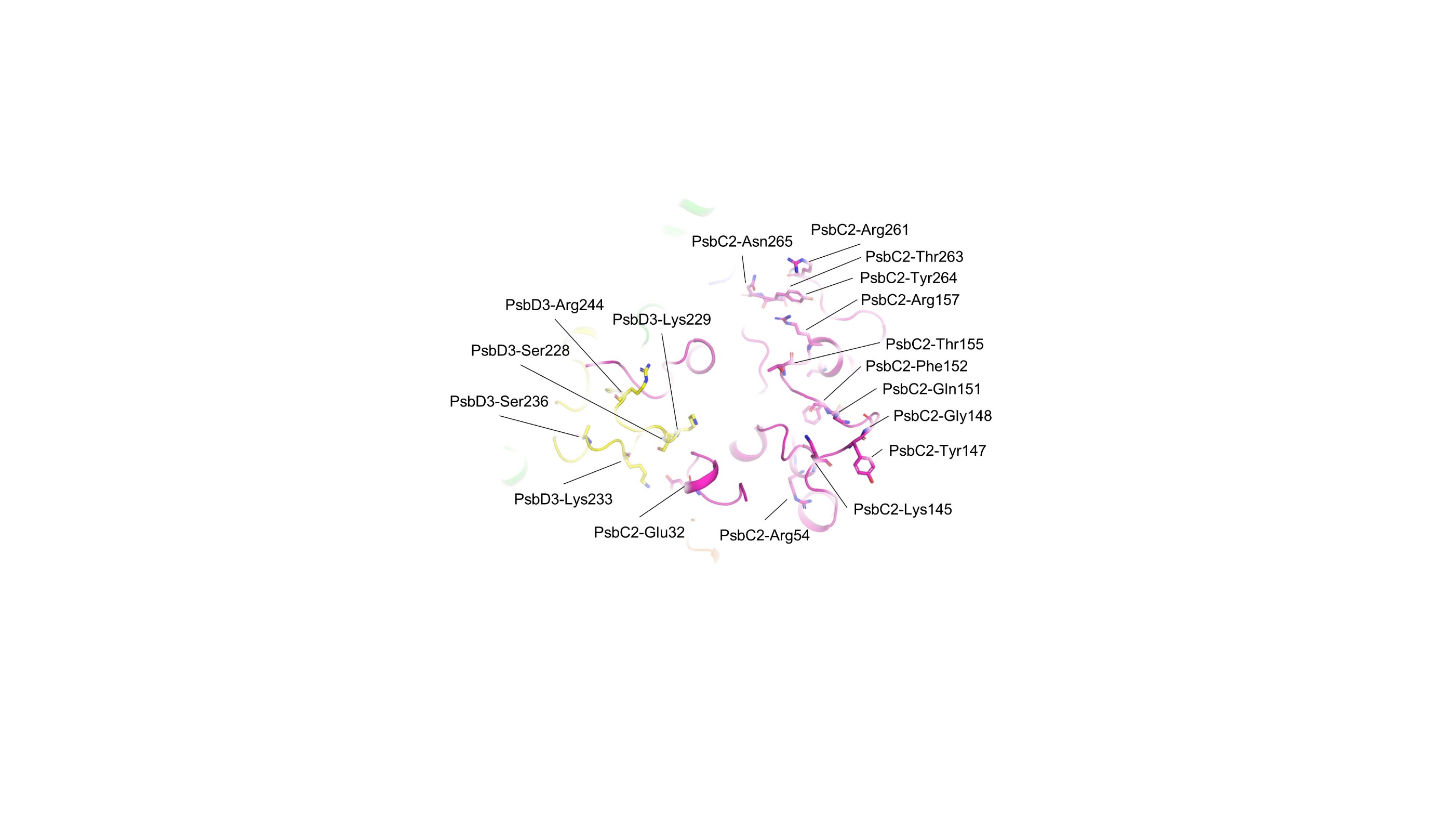


**Figure S10. FRL-specific sequence differences that may interact with FRL-BC.** A stromal view of the stromal surface near PsbD3 and PsbC2 is shown. Residues that may interact with ApcE2, the FRL-specific isoform of ApcE in BCs, during FaRLiP are labeled and correspond to the region of spheres shown in **Fig. 2**.

**
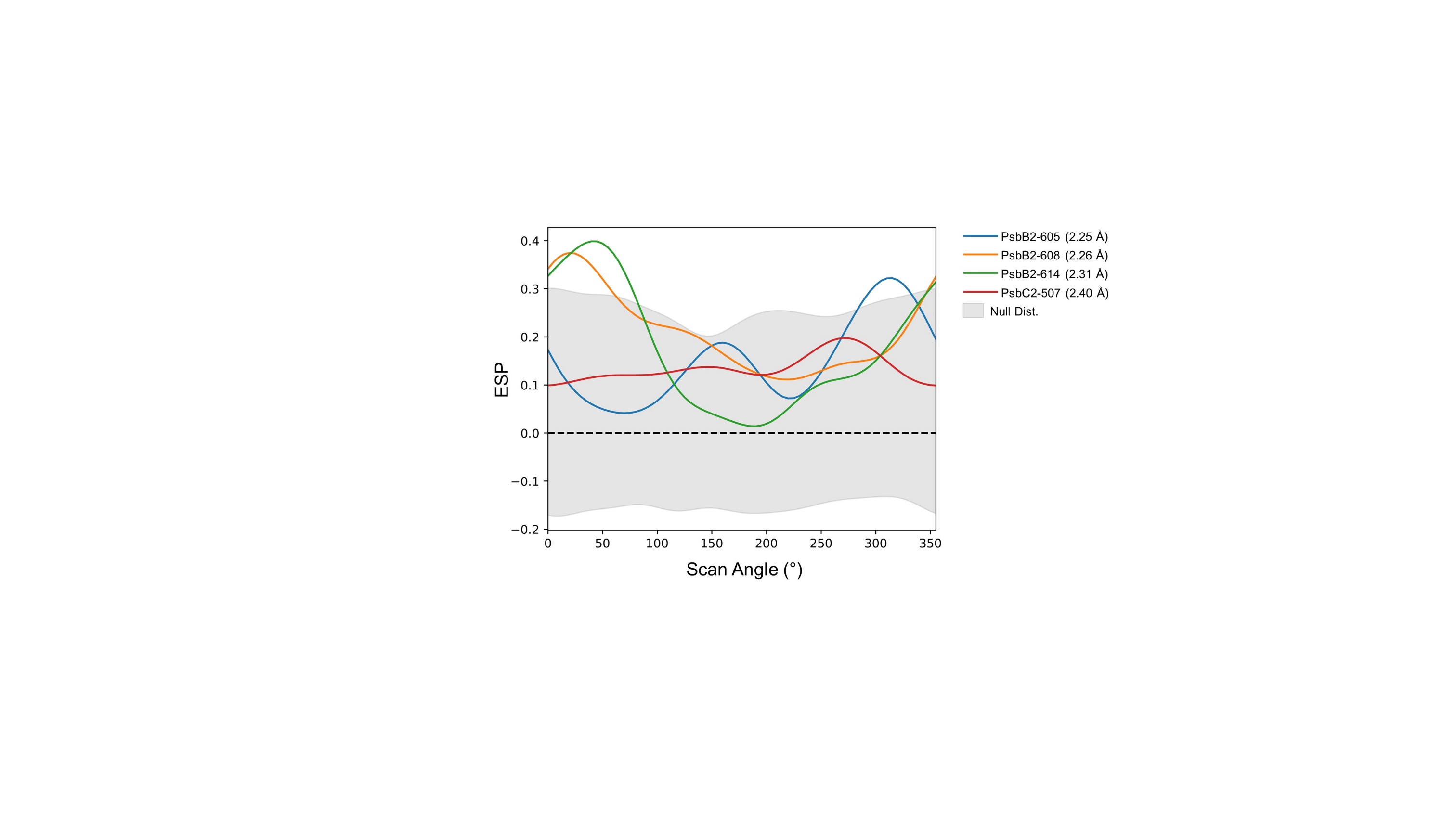
**

**Figure S11. C2 cone scans of proposed Chl *f* sites.** The C2 cone scan for each proposed Chl *f* site is shown, colored according to the key. The local resolution at the central Mg of each Chl site is labeled in the key. The null distribution derived from cone scans of the C7 substituents (see **Experimental Procedures**) is shaded grey.

▼

*S*. 7335 PsaD3 FSTDQGEETYSMV 247

*S*. 7335 PsbD2 FEPTQSEETYSMV 247

*S*. 6803 PsaD FEPTQAEETYSMV 247

*T. vulcanus* PsaD FNPTQAEETYSMV 237

*T. elongatus* PsaD FNPTQAEETYSMV 247

*. *.*******

**Figure S12. Partial sequence alignment of PsbD(3).** The *Synechococcus* 7335 sequence colored in red is the FRL-specific sequence found in the apo-FRL-PSII structure. A WL-specific *Synechococcus* 7335 PsbD sequence is shown (PsbD2) and the three PsbD sequences from *Synechocystis* sp. PCC 6803, *T. vulcanus*, and *T. elongatus*. A red arrow signifies the highly conserved D2-Glu241 residue that ligates the NH-Fe in the apo-FRL-PSII structure. Abbreviations: S. 7335, *Synechococcus* sp. PCC 7335; S. 6803, *Synechocystis* sp. PCC 6803; T., *Thermosynechococcus*.


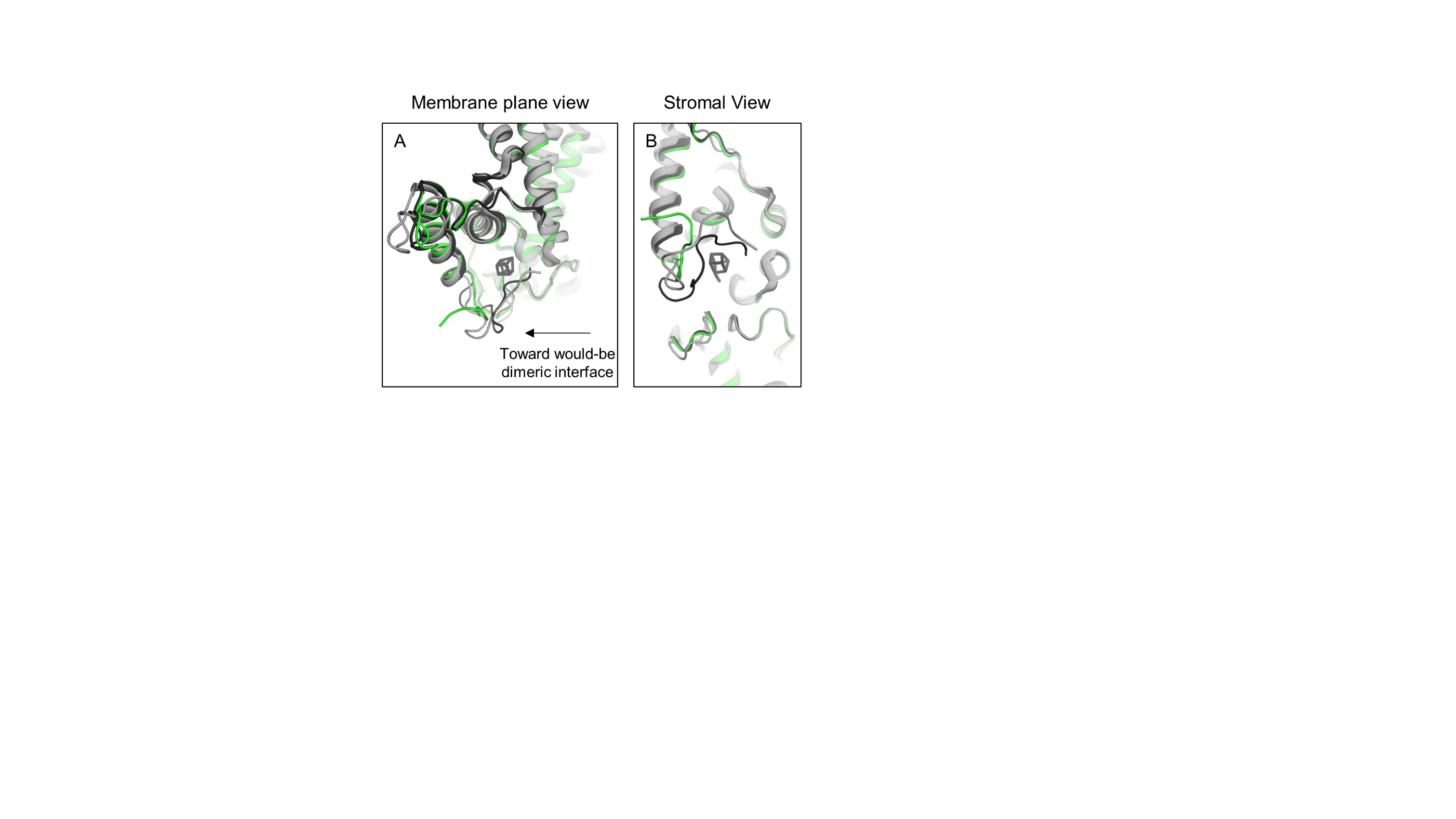


**Figure S13. Orientation of apo-FRL-PSII PsbA3 (D1) C-terminus.** The superposition of the PsbA subunit (D1) from *Synechococcus* 7335 apo-FRL-PSII (green), a *T. elongatus* PSII assembly intermediate (light grey, PDB 7NHP), and *T. vulcanus* mature PSII (dark grey, PDB 3WU2) is shown. In the latter, the OEC is also shown in stick representation. **A.** Membrane plane view. The direction toward the (would-be) dimeric interface is labeled. **B.** Stromal view.


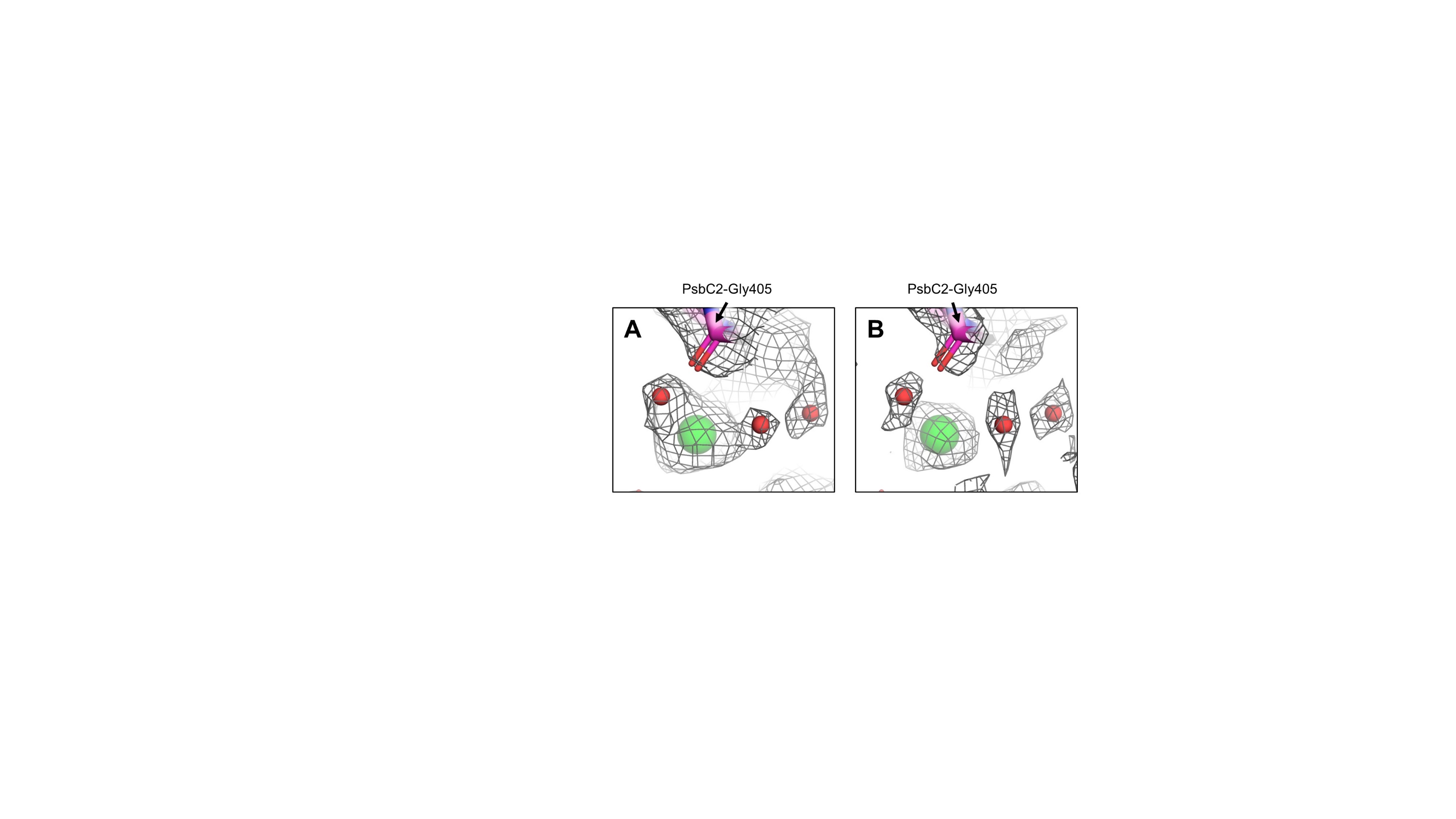


**Figure S14. Map region corresponding to a cation near the OEC-binding site and comparison with nearby water molecules. A.** Unsharpened map at 6.5σ. **B.** Sharpened at 8.5σ. In both images, the cation is shown as a green sphere, waters are shown as red spheres, and PsbC2-Gly405 is labeled.


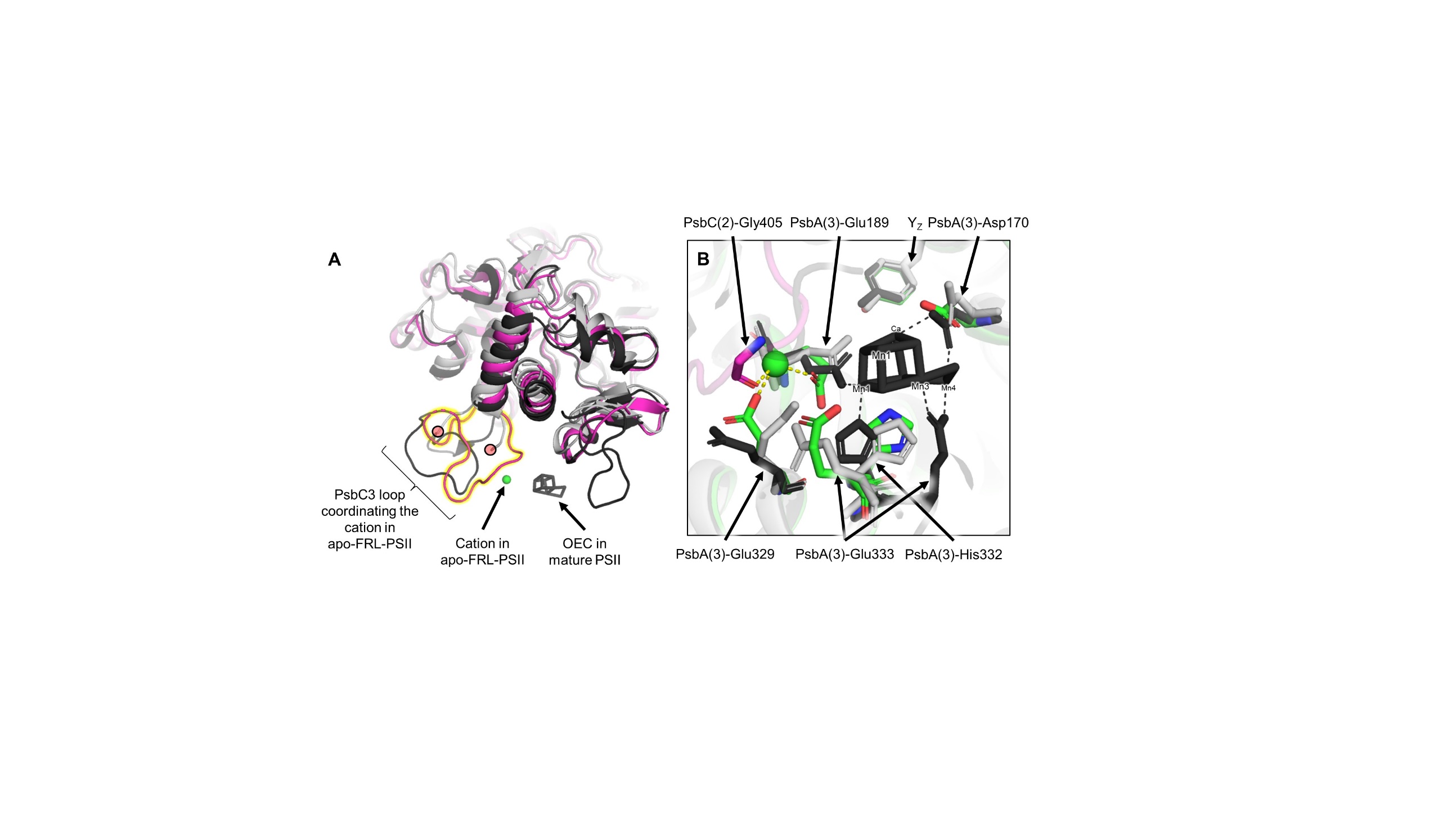


**Figure S15. Cation bound near the vacant OEC-binding pocket and nearby residue positions. A.** The cartoon representation of PsbC(2) (CP43) is shown from a lumenal view for three structures: *Synechococcus* 7335 apo-FRL-PSII (colored), *Synechocystis* sp. PCC 6803 apo-PSII (light grey, PDB 6WJ6), and *T. vulcanus* mature PSII (dark grey, PDB 3WU2). The loop identified in the *Synechococcus* 7335 apo-FRL-PSII structure to bind the cation (green sphere) is highlighted yellow. The last modeled residues before and after the same loop incompletely modeled in the *Synechocystis* sp. PCC 6803 apo-PSII structure are designated with red transparent circles. The OEC found in *T. vulcanus* mature PSII is shown in stick representation. **B.** View near the OEC-binding site where cartoons are made transparent and residues of interest are shown in stick representation and labeled. Ligands that coordinate the cation in apo-FRL-PSII are shown as yellow dashed lines. The OEC from mature PSII is shown in stick representation, and ions are labeled, adjusting font size to denote depth from the perspective shown (e.g., smaller = further away). Please note the footnote regarding residue numbering in the main text.^


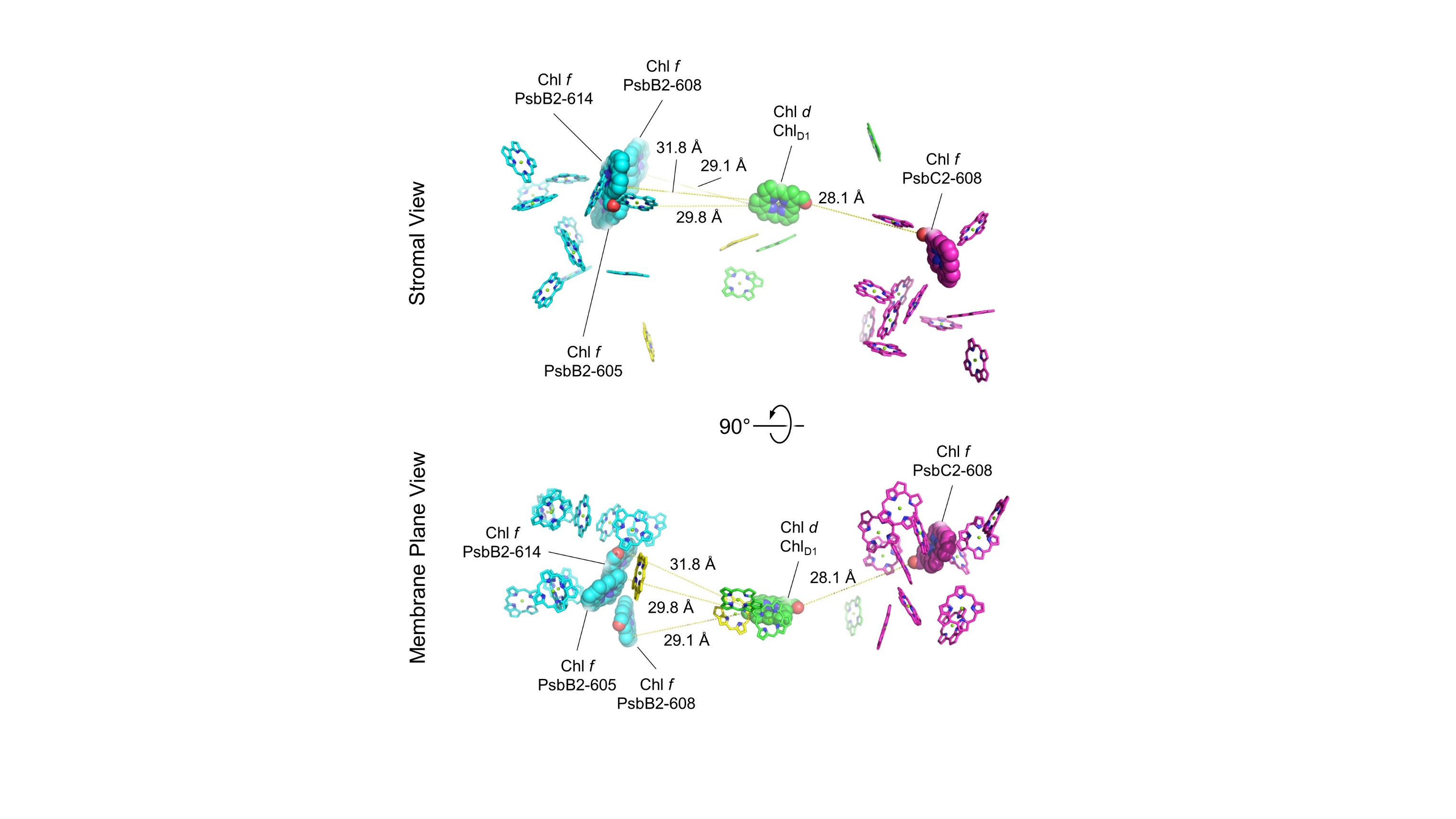


**Figure S16. Distances of Chl *f* molecules to Chl *d* in the ETC.** Stromal and membrane plane views of the apo-FRL-PSII structure are shown in which only the tetrapyrrole rings of Chls are displayed. Tetrapyrrole rings of Chl *d* and Chl *f* molecules, and additionally their formyl moieties, are shown in sphere representation. The edge-to-edge distance is labeled from each Chl *f* to the Chl *d* in the ETC.


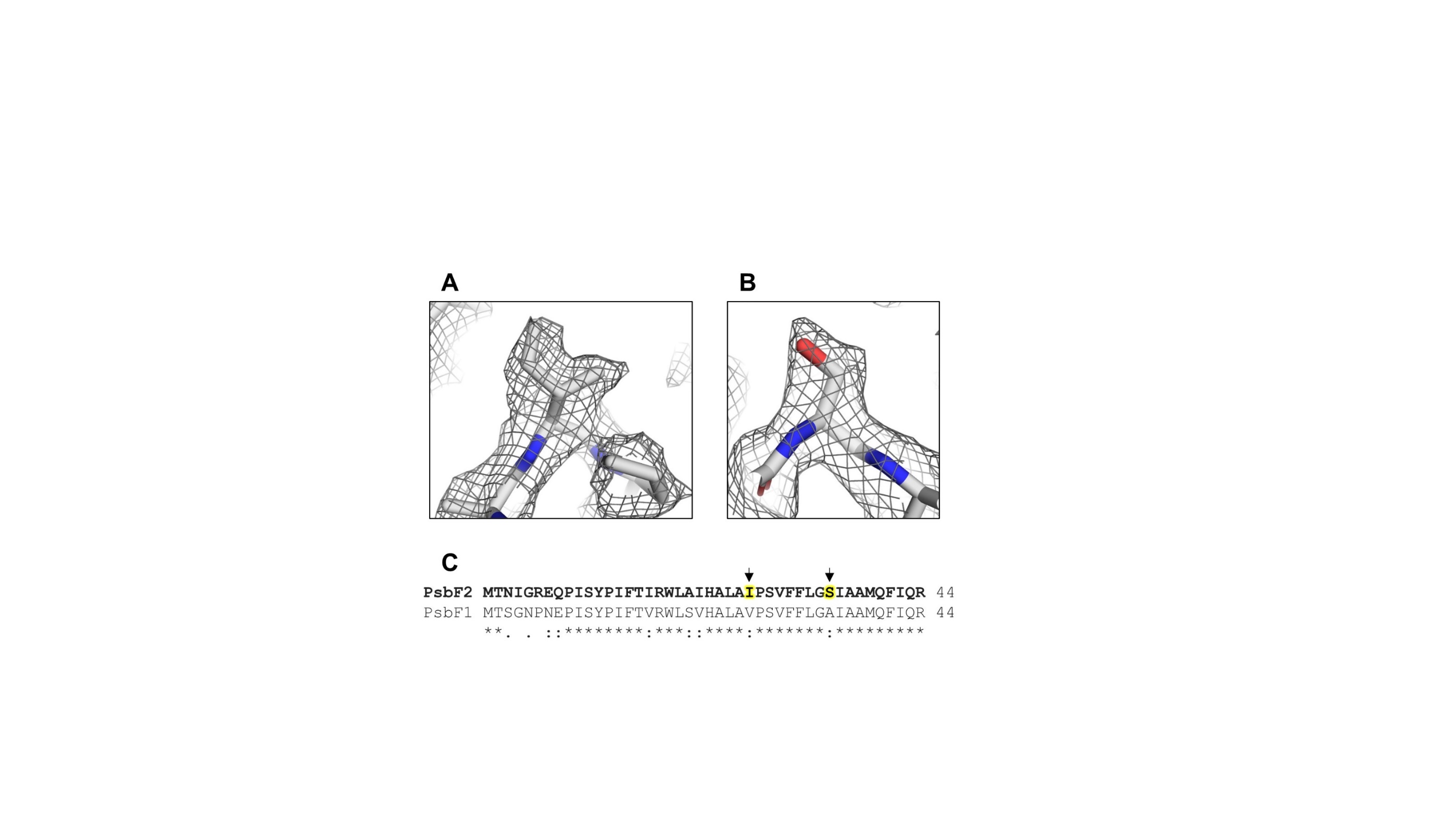


**Figure S17. Differentiation of PsbF1 and PsbF2 assignments in the apo-FRL-PSII ESP map. A.** The ESP map and model of PsbF2-Ile27. **B.** The ESP map and model of PsbF2-Ser35. **C** The sequence alignment of PsbF1 and PsbF2. The residues corresponding to panels A and B are labeled with an arrow. The PsbF2 residues that fit the ESP more convincingly than the analogous residues in PsbF1 are highlighted. Thus, the sequence for PsbF2 was used in modeling, which is in bold. The ESP for other sequence differences was too poor to differentiate between sidechains.

**Supporting Tables**

**Table S1. Identification of subunits in apo-FRL-PSII complexes from *Synechococcus* 7335 based upon tryptic peptide fingerprinting and MS/MS mass spectrometry.** A few minor contaminating proteins detected by single peptides were not included here. If the subunit exhibits FRL or WL specificity, it is noted in parentheses. The green shading indicates subunits whose sequences were used in the structural model of the apo-FRL-PSII core complexes.

| **Protein** | **–10lgP** | **Coverage**  **(%)** | **# Peptides** | **Locus tag/Description** |
| --- | --- | --- | --- | --- |
| PsbC2 (FRL) | 357.21 | 70 | 60 | S7335_3753 photosystem II 44 kDa subunit |
| PsbB2 (FRL) | 350.64 | 51 | 56 | S7335_4830 photosystem II chlorophyll-binding protein |
| PsbD3 (FRL) | 334.43 | 51 | 43 | S7335_2548 photosystem II D2 protein |
| PsbA3 (FRL) | 285.41 | 37 | 17 | S7335_4273 photosystem II D1 protein |
| PsbD2 (WL) | 267.05 | 17 | 17 | S7335_1444 photosystem II D2 protein |
| PsbF2 | 191.22 | 86 | 7 | S7335_3212 cytochrome *b*_559_ beta subunit |
| PsbF1 | 186.15 | 93 | 6 | S7335_3021 cytochrome *b*_559_ beta subunit |
| PsbE | 178.87 | 54 | 7 | S7335_3478 cytochrome *b*_559_ alpha subunit |
| PsbK | 177.92 | 82 | 3 | S7335_3963 photosystem II 4 kDa reaction center component |
| PsbC1 (WL) | 149.01 | 7 | 4 | S7335_4424 photosystem II 44 kDa subunit |
| PsbH2 (FRL) | 89.32 | 36 | 3 | S7335_1601 photosystem II 10 kDa phosphoprotein |

**Table S2.** **Cryo-EM data collection, refinement, and validation statistics for apo-FRL-PSII from *Synechococcus* 7335 (PDB 7SA3).**

| **Data collection and processing** |  |  |
| --- | --- | --- |
| Magnification | ×105,000 | |
| Voltage (kV) | 300 | |
| Electron exposure (e^-^Å^-2^) | 40.8 | |
| Defocus range (µm) | –1.0 to –2.0 | |
| Pixel size (Å) | 0.413 | |
| Symmetry imposed | C1 | |
| Initial particle images (no.) | 958,755 | |
| Final particle images (no.) | 315,307 | |
| Map resolution (Å) | 2.25 | |
| FSC threshold | 0.143 | |
| **Refinement** |  | |
| Initial model used (PDB code) | 3WU2 | |
| Model resolution (Å) | 2.27 | |
| FSC threshold | 0.5 | |
| Map resolution range (Å) | 2.10-2.90 | |
| Map-sharpening *B* factor (Å^2^) | –37.39 | |
| Model composition |  | |
| Non-hydrogen atoms | 15,963 | |
| Protein residues | 1,667 | |
| Ligands | 286 | |
| *B* factors (Å^2^) |  | |
| Protein | 31 | |
| Ligands | 31 | |
| R.m.s. deviations |  | |
| Bond lengths (Å) | 0.008 | |
| Bond angles (°) | 1.619 | |
| **Validation** |  | |
| MolProbity | 2.28 | |
| Clashscore | 10.70 | |
| Rotamer outliers (%) | 4.04 | |
| Ramachandran plot |  | |
| Favored (%) | 96.09 | |
| Allowed (%) | 3.67 | |
| Disallowed (%) | 0.24 | |

**Table S3. Root-mean-square deviation of α-carbons in core subunits of *Synechococcus* 7335 apo-FRL-PSII with homologous subunits from other cyanobacterial PSII structures.**

Values are reported in units of Å.

|  | **PsbA** | **PsbD** | **PsbB** | **PsbC** | **PsbE** | **PsbF** | **PsbI** | **PsbK** |
| --- | --- | --- | --- | --- | --- | --- | --- | --- |
| **Mature PSII from *T. vulcanus***  **(PDB 3WU2)** | 0.465 | 0.373 | 0.528 | 0.499 | 0.296 | 0.392 | 0.343 | 0.486 |
| **Apo-PSII from *Synechocystis* sp, PCC 6803 (PDB 6WJ6)** | 0.440 | 0.390 | 0.517 | 0.539 | 0.479 | 0.260 | 0.349 | 0.543 |

**Table S4. Sequence identity of *Synechococcus* 7335 apo-FRL-PSII subunits compared to homologous subunits from other cyanobacterial PSII structures.**

Values are reported in units of %.

|  | **PsbA** | **PsbD** | **PsbB** | **PsbC** | **PsbE** | **PsbF** | **PsbI** | **PsbK** |
| --- | --- | --- | --- | --- | --- | --- | --- | --- |
| **Mature PSII from *T. vulcanus***  **(PDB 3WU2)** | 77.50 | 82.16 | 70.89 | 70.76 | 67.50 | 63.64 | 68.42 | 64.86 |
| **Apo-PSII from *Synechocystis* sp, PCC 6803 (PDB 6WJ6)** | 76.67 | 77.56 | 70.61 | 67.61 | 67.50 | 69.77 | 63.16 | 64.44 |

**Supporting Data**

**Supplementary Data 1. Jupyter Notebook for cone scans (external file).** A Jupyter Notebook containing the raw data and cone scans for every Chl in the apo-FRL-PSII structure from *Synechococcus* 7335 is provided.
