# Supplementary material for: Structure of a monomeric photosystem II core complex from a cyanobacterium acclimated to far-red light reveals the functions of chlorophylls d and f
Source: J Biol Chem. 2021 Nov 19;298(1):101424. doi: 10.1016/j.jbc.2021.101424 (PMC8689208; doi:10.1016/j.jbc.2021.101424)
Supplement: Data S1 [file mmc2.zip › FRL-PSII_7335_cone_scans.html]

FRL-PSII\_7335\_cone\_scans


# Cone Scan data for FRL-PSII from *Synechococcus* sp. PCC 7335¶

This report contains the raw data for the cone scans produced for FRL-PSII from *Synechococcus* sp. PCC 7335, as referred to throughout the manuscript.

In [1]:

```
import numpy as np
import pandas as pd
import matplotlib.pyplot as plt
import matplotlib.patches as mpatches
import matplotlib.lines as mlines
```

#### Data availability¶

The raw data is contained in the following dictionary object. This is used to create a pandas DataFrame with the following columns:

- chl\_id: The chlorophyll binding sites of FRL-PSII from *Synechococcus* sp. PCC 7335
- loc\_res\_Mg: The local resolution of the ESP map at the central Mg of the chlorophyll
- scan\_angles: The different angles as which the ESP is sampled
- scan\_amp\_C2: The ESP sampled on the C2 substituent
- scan\_amp\_C7: The ESP sampled on the C7 substituent
- ci: The upper and lower bounds of the null distrubution

In [2]:

```
data = {'chl_id': {'A405': 'A405', 'A407': 'A407', 'A410': 'A410', 'B603': 'B603', 'B604': 'B604', 'B605': 'B605', 'B606': 'B606', 'B607': 'B607', 'B608': 'B608', 'B609': 'B609', 'B610': 'B610', 'B611': 'B611', 'B612': 'B612', 'B613': 'B613', 'B614': 'B614', 'B616': 'B616', 'B617': 'B617', 'C501': 'C501', 'C502': 'C502', 'C503': 'C503', 'C504': 'C504', 'C505': 'C505', 'C506': 'C506', 'C507': 'C507', 'C508': 'C508', 'C509': 'C509', 'C510': 'C510', 'C511': 'C511', 'C512': 'C512', 'C513': 'C513', 'D402': 'D402', 'D403': 'D403'}, 'loc_res_Mg': {'A405': 2.18, 'A407': 2.18, 'A410': 2.36, 'B603': 2.37, 'B604': 2.29, 'B605': 2.25, 'B606': 2.29, 'B607': 2.39, 'B608': 2.26, 'B609': 2.3, 'B610': 2.52, 'B611': 2.37, 'B612': 2.33, 'B613': 2.36, 'B614': 2.31, 'B616': 2.49, 'B617': 2.59, 'C501': 2.42, 'C502': 2.34, 'C503': 2.46, 'C504': 2.38, 'C505': 2.34, 'C506': 2.5, 'C507': 2.4, 'C508': 2.34, 'C509': 2.38, 'C510': 2.37, 'C511': 2.47, 'C512': 2.49, 'C513': 2.64, 'D402': 2.17, 'D403': 2.35}, 'scan_amp_C2': {'A405': [0.01096, 0.01136, 0.01185, 0.0124, 0.01301, 0.01364, 0.01426, 0.01484, 0.01532, 0.01569, 0.01592, 0.01598, 0.01587, 0.01561, 0.01521, 0.01471, 0.01414, 0.01355, 0.01299, 0.01251, 0.01215, 0.01193, 0.01187, 0.01197, 0.01224, 0.01263, 0.01314, 0.01374, 0.01439, 0.01505, 0.01573, 0.01638, 0.01699, 0.01757, 0.01811, 0.01861, 0.01905, 0.01945, 0.0198, 0.0201, 0.02034, 0.02052, 0.02063, 0.02067, 0.02061, 0.02044, 0.02014, 0.01969, 0.01908, 0.01831, 0.01738, 0.01632, 0.01516, 0.01396, 0.01277, 0.01165, 0.01066, 0.00984, 0.00922, 0.00881, 0.0086, 0.00856, 0.00865, 0.00884, 0.00907, 0.00932, 0.00955, 0.00976, 0.00996, 0.01015, 0.01037, 0.01063], 'A407': [0.01362, 0.01394, 0.01404, 0.01393, 0.01359, 0.01306, 0.01238, 0.01159, 0.01074, 0.00989, 0.0091, 0.00841, 0.00785, 0.00747, 0.00728, 0.00731, 0.00756, 0.00801, 0.00869, 0.00956, 0.01061, 0.01181, 0.01313, 0.01452, 0.01594, 0.01733, 0.01865, 0.01984, 0.02084, 0.02161, 0.02213, 0.02236, 0.02231, 0.02198, 0.02139, 0.02059, 0.01962, 0.01853, 0.01738, 0.01624, 0.01515, 0.01417, 0.01332, 0.01264, 0.01213, 0.01179, 0.01162, 0.01157, 0.01162, 0.01172, 0.01184, 0.01193, 0.01196, 0.01189, 0.0117, 0.0114, 0.011, 0.01051, 0.00998, 0.00946, 0.00899, 0.00863, 0.00842, 0.0084, 0.00857, 0.00893, 0.00947, 0.01014, 0.01091, 0.0117, 0.01245, 0.01311], 'A410': [0.01437, 0.01299, 0.0117, 0.01052, 0.00946, 0.00854, 0.00777, 0.00715, 0.00668, 0.00636, 0.00618, 0.00611, 0.00614, 0.00622, 0.00632, 0.00641, 0.00643, 0.00636, 0.00616, 0.00583, 0.00536, 0.00476, 0.00403, 0.00321, 0.00231, 0.00138, 0.00044, -0.00051, -0.00142, -0.0023, -0.00313, -0.0039, -0.00461, -0.00525, -0.00579, -0.00622, -0.00651, -0.00661, -0.00651, -0.00617, -0.00556, -0.00468, -0.00351, -0.00207, -0.0004, 0.00149, 0.00355, 0.00572, 0.00795, 0.01018, 0.01239, 0.01453, 0.01656, 0.01844, 0.02017, 0.0217, 0.02302, 0.02413, 0.02499, 0.0256, 0.02595, 0.02603, 0.02584, 0.0254, 0.02472, 0.02383, 0.02274, 0.02151, 0.02017, 0.01873, 0.01727, 0.01581], 'B603': [0.00214, 0.00039, -0.00138, -0.00312, -0.00473, -0.00615, -0.00734, -0.00824, -0.00885, -0.00915, -0.00915, -0.00888, -0.00836, -0.00763, -0.00673, -0.0057, -0.00458, -0.00339, -0.00219, -0.001, 0.00016, 0.00126, 0.00229, 0.00324, 0.00411, 0.0049, 0.00561, 0.00625, 0.00685, 0.0074, 0.00792, 0.00843, 0.00893, 0.00943, 0.00993, 0.01042, 0.0109, 0.01135, 0.01176, 0.01212, 0.01239, 0.01259, 0.01269, 0.01268, 0.01257, 0.01236, 0.01207, 0.01169, 0.01124, 0.01076, 0.01024, 0.00972, 0.00922, 0.00877, 0.00839, 0.00811, 0.00793, 0.00787, 0.00793, 0.00808, 0.00831, 0.00857, 0.00883, 0.00903, 0.00912, 0.00906, 0.00879, 0.0083, 0.00754, 0.00653, 0.00527, 0.00379], 'B604': [0.00302, 0.00199, 0.00105, 0.0002, -0.00056, -0.00122, -0.00178, -0.00224, -0.00259, -0.00284, -0.00299, -0.00306, -0.00306, -0.00302, -0.00296, -0.00289, -0.00284, -0.00281, -0.00279, -0.00278, -0.00276, -0.0027, -0.00258, -0.00238, -0.00208, -0.00168, -0.00117, -0.00058, 8e-05, 0.00079, 0.00152, 0.00226, 0.00298, 0.00369, 0.00436, 0.005, 0.00564, 0.00627, 0.00692, 0.00762, 0.00837, 0.00919, 0.0101, 0.01108, 0.01213, 0.01323, 0.01436, 0.01546, 0.01652, 0.01749, 0.01833, 0.01903, 0.01954, 0.01986, 0.01999, 0.01992, 0.01968, 0.01927, 0.01871, 0.01802, 0.01721, 0.0163, 0.01529, 0.01419, 0.01302, 0.01178, 0.0105, 0.0092, 0.00788, 0.00658, 0.00533, 0.00414], 'B605': [0.0173, 0.01517, 0.01321, 0.01146, 0.00994, 0.00865, 0.00758, 0.0067, 0.00599, 0.00542, 0.00496, 0.00461, 0.00435, 0.00418, 0.00413, 0.00419, 0.00438, 0.00472, 0.00522, 0.00588, 0.0067, 0.00768, 0.00879, 0.01002, 0.01134, 0.01269, 0.01403, 0.01531, 0.01647, 0.01744, 0.01819, 0.01864, 0.01878, 0.01858, 0.01805, 0.01721, 0.01609, 0.01477, 0.01333, 0.01185, 0.01045, 0.00921, 0.00821, 0.00752, 0.0072, 0.00725, 0.00768, 0.00848, 0.00959, 0.01098, 0.01259, 0.0144, 0.01631, 0.01832, 0.02037, 0.02242, 0.02441, 0.0263, 0.02804, 0.02955, 0.03078, 0.03167, 0.03215, 0.03221, 0.03181, 0.03098, 0.02973, 0.02812, 0.02621, 0.02408, 0.02183, 0.01954], 'B606': [0.01064, 0.01021, 0.00963, 0.0089, 0.00805, 0.0071, 0.00607, 0.00499, 0.00388, 0.00278, 0.00172, 0.00074, -0.00012, -0.00081, -0.0013, -0.00155, -0.00154, -0.00125, -0.00068, 0.00013, 0.00117, 0.0024, 0.00377, 0.00521, 0.00669, 0.00816, 0.00956, 0.01087, 0.01205, 0.01308, 0.01395, 0.01466, 0.0152, 0.01558, 0.01582, 0.01593, 0.01593, 0.01587, 0.01578, 0.0157, 0.01566, 0.0157, 0.01583, 0.01607, 0.01642, 0.01685, 0.01732, 0.01779, 0.01818, 0.01846, 0.01854, 0.01841, 0.01803, 0.01741, 0.01657, 0.01557, 0.01446, 0.01333, 0.01224, 0.01128, 0.01049, 0.00993, 0.0096, 0.00949, 0.00957, 0.00979, 0.01011, 0.01044, 0.01073, 0.01092, 0.01099, 0.0109], 'B607': [0.00714, 0.0085, 0.00991, 0.01131, 0.01261, 0.01376, 0.0147, 0.01539, 0.01582, 0.016, 0.01594, 0.01567, 0.01525, 0.01472, 0.01413, 0.01352, 0.01293, 0.01237, 0.01187, 0.01142, 0.01102, 0.01065, 0.01029, 0.00994, 0.00956, 0.00916, 0.00873, 0.00826, 0.00779, 0.00732, 0.0069, 0.00654, 0.00629, 0.00617, 0.00621, 0.00641, 0.0068, 0.00733, 0.00801, 0.0088, 0.00964, 0.01052, 0.01136, 0.01213, 0.01278, 0.01329, 0.01361, 0.01374, 0.01366, 0.01337, 0.01289, 0.01224, 0.01145, 0.01056, 0.0096, 0.00862, 0.00765, 0.00671, 0.00584, 0.00504, 0.00434, 0.00374, 0.00324, 0.00286, 0.00261, 0.0025, 0.00256, 0.00281, 0.00326, 0.00394, 0.00482, 0.0059], 'B608': [0.03414, 0.03551, 0.03655, 0.0372, 0.03746, 0.03731, 0.03678, 0.03593, 0.0348, 0.03347, 0.03202, 0.03052, 0.02906, 0.0277, 0.02646, 0.0254, 0.02451, 0.0238, 0.02325, 0.02284, 0.02253, 0.02228, 0.02205, 0.02182, 0.02153, 0.02117, 0.02073, 0.0202, 0.01957, 0.01888, 0.01812, 0.01733, 0.01652, 0.01573, 0.01498, 0.01427, 0.01363, 0.01305, 0.01255, 0.01211, 0.01176, 0.01148, 0.01127, 0.01115, 0.01113, 0.01121, 0.01139, 0.01168, 0.01205, 0.01247, 0.01293, 0.01338, 0.0138, 0.01416, 0.01443, 0.01463, 0.01477, 0.01489, 0.01504, 0.01527, 0.01563, 0.01619, 0.01697, 0.01801, 0.0193, 0.02085, 0.02262, 0.02453, 0.02656, 0.02861, 0.03061, 0.03248], 'B609': [0.01031, 0.00975, 0.00918, 0.00861, 0.00804, 0.00746, 0.00685, 0.00619, 0.00546, 0.00464, 0.00372, 0.0027, 0.00158, 0.00041, -0.00082, -0.00205, -0.00324, -0.00437, -0.00538, -0.00624, -0.00692, -0.00741, -0.00766, -0.00767, -0.00744, -0.00696, -0.00627, -0.00536, -0.0043, -0.00312, -0.00187, -0.00062, 0.00061, 0.00176, 0.00282, 0.00375, 0.00458, 0.00531, 0.00596, 0.00657, 0.00716, 0.00776, 0.00835, 0.00894, 0.00951, 0.01002, 0.01045, 0.01076, 0.01094, 0.01099, 0.01092, 0.01076, 0.01055, 0.01032, 0.01013, 0.01001, 0.00999, 0.01008, 0.01028, 0.01057, 0.01092, 0.0113, 0.01167, 0.012, 0.01224, 0.01239, 0.01241, 0.01231, 0.01209, 0.01175, 0.01133, 0.01084], 'B610': [0.01314, 0.01235, 0.01144, 0.01047, 0.00948, 0.00852, 0.00764, 0.00686, 0.00622, 0.00572, 0.00538, 0.00517, 0.00507, 0.00505, 0.00507, 0.0051, 0.00509, 0.00502, 0.00486, 0.0046, 0.00424, 0.00379, 0.00327, 0.0027, 0.00211, 0.00155, 0.00103, 0.00059, 0.00026, 3e-05, -7e-05, -7e-05, 5e-05, 0.00024, 0.00051, 0.00082, 0.00117, 0.00153, 0.0019, 0.00226, 0.00262, 0.00296, 0.00329, 0.00361, 0.00393, 0.00425, 0.00458, 0.00493, 0.00531, 0.00573, 0.00619, 0.00671, 0.00729, 0.00791, 0.00858, 0.00927, 0.00998, 0.01068, 0.01136, 0.01201, 0.01261, 0.01315, 0.01364, 0.01405, 0.0144, 0.01467, 0.01485, 0.01492, 0.01486, 0.01467, 0.01432, 0.01381], 'B611': [0.00119, 0.00142, 0.00183, 0.00241, 0.00311, 0.0039, 0.00474, 0.00557, 0.00636, 0.00708, 0.00768, 0.00815, 0.00848, 0.00866, 0.00871, 0.00865, 0.00849, 0.00825, 0.00796, 0.00765, 0.00732, 0.00699, 0.00668, 0.0064, 0.00616, 0.00596, 0.00581, 0.00573, 0.00572, 0.00577, 0.00589, 0.00606, 0.00627, 0.0065, 0.00671, 0.00688, 0.00699, 0.00701, 0.00691, 0.00671, 0.00639, 0.00599, 0.00552, 0.00502, 0.00453, 0.00408, 0.00371, 0.00346, 0.00333, 0.00334, 0.00349, 0.00377, 0.00413, 0.00457, 0.00505, 0.00552, 0.00595, 0.00629, 0.00652, 0.00661, 0.00655, 0.00632, 0.00594, 0.00542, 0.0048, 0.00412, 0.00341, 0.00273, 0.00213, 0.00165, 0.00131, 0.00116], 'B612': [0.00985, 0.00932, 0.00859, 0.00773, 0.0068, 0.00587, 0.00503, 0.00434, 0.00384, 0.00358, 0.00355, 0.00375, 0.00417, 0.00478, 0.00553, 0.00642, 0.00741, 0.00848, 0.00964, 0.01085, 0.01211, 0.01343, 0.01477, 0.01612, 0.01747, 0.01878, 0.02005, 0.02124, 0.02235, 0.02334, 0.0242, 0.0249, 0.02545, 0.02581, 0.02597, 0.02593, 0.0257, 0.02527, 0.02467, 0.02394, 0.02311, 0.02223, 0.02133, 0.02045, 0.01962, 0.01885, 0.01814, 0.01751, 0.01693, 0.01638, 0.01584, 0.01529, 0.0147, 0.01407, 0.01338, 0.01265, 0.01187, 0.01108, 0.01032, 0.00962, 0.00902, 0.00857, 0.0083, 0.00822, 0.00833, 0.00859, 0.00896, 0.00939, 0.00978, 0.01008, 0.01022, 0.01015], 'B613': [-0.00771, -0.00874, -0.00963, -0.01037, -0.01093, -0.0113, -0.01146, -0.01137, -0.011, -0.01033, -0.00935, -0.00805, -0.00646, -0.00462, -0.00256, -0.00035, 0.00193, 0.00423, 0.00647, 0.00859, 0.01054, 0.01229, 0.01377, 0.015, 0.01594, 0.01658, 0.01691, 0.01695, 0.0167, 0.01616, 0.01538, 0.01437, 0.01319, 0.01189, 0.01052, 0.00914, 0.00781, 0.0066, 0.00555, 0.00471, 0.00412, 0.00378, 0.00371, 0.00389, 0.00429, 0.00489, 0.00564, 0.0065, 0.00741, 0.00833, 0.00923, 0.01007, 0.0108, 0.0114, 0.01186, 0.01212, 0.01219, 0.01203, 0.01163, 0.01099, 0.0101, 0.00899, 0.00767, 0.00617, 0.00455, 0.00285, 0.00113, -0.00057, -0.00222, -0.00378, -0.00521, -0.00653], 'B614': [0.03265, 0.03387, 0.03504, 0.03617, 0.03723, 0.03818, 0.03898, 0.03956, 0.03986, 0.03983, 0.03941, 0.03857, 0.03729, 0.03559, 0.03349, 0.03104, 0.02835, 0.0255, 0.02258, 0.0197, 0.01697, 0.01446, 0.01222, 0.01031, 0.00871, 0.00742, 0.00641, 0.00561, 0.00498, 0.00446, 0.00399, 0.00356, 0.00313, 0.0027, 0.00229, 0.00191, 0.00162, 0.00143, 0.00139, 0.00155, 0.0019, 0.00246, 0.0032, 0.00408, 0.00508, 0.00613, 0.00716, 0.00812, 0.00897, 0.00968, 0.01023, 0.01064, 0.01094, 0.01116, 0.01137, 0.01162, 0.01197, 0.01245, 0.01312, 0.01398, 0.01504, 0.01629, 0.01769, 0.01923, 0.02083, 0.02247, 0.0241, 0.02569, 0.02722, 0.02868, 0.03006, 0.03138], 'B616': [0.01137, 0.01188, 0.01238, 0.01283, 0.01321, 0.01349, 0.01367, 0.01373, 0.01364, 0.01341, 0.01304, 0.01254, 0.01191, 0.01119, 0.01038, 0.00951, 0.00861, 0.0077, 0.00682, 0.00599, 0.00525, 0.00462, 0.00412, 0.00378, 0.00359, 0.00355, 0.00365, 0.00386, 0.00417, 0.00452, 0.00489, 0.00525, 0.00558, 0.00585, 0.00606, 0.00621, 0.00632, 0.00639, 0.00646, 0.00652, 0.0066, 0.00669, 0.00679, 0.00688, 0.00694, 0.00694, 0.00686, 0.00669, 0.0064, 0.00601, 0.00553, 0.00501, 0.00446, 0.00396, 0.00353, 0.00324, 0.0031, 0.00314, 0.00336, 0.00373, 0.00424, 0.00484, 0.0055, 0.00617, 0.00684, 0.00749, 0.0081, 0.00868, 0.00923, 0.00977, 0.0103, 0.01084], 'B617': [-0.0024, -0.0022, -0.00183, -0.00131, -0.00067, 7e-05, 0.00087, 0.0017, 0.00253, 0.00332, 0.00405, 0.00469, 0.00523, 0.00565, 0.00595, 0.00613, 0.00619, 0.00616, 0.00605, 0.00587, 0.00566, 0.00543, 0.00522, 0.00505, 0.00494, 0.00491, 0.00497, 0.00513, 0.00539, 0.00574, 0.00616, 0.00664, 0.00716, 0.00767, 0.00817, 0.00861, 0.00897, 0.00923, 0.00938, 0.0094, 0.00931, 0.00911, 0.00882, 0.00846, 0.00805, 0.00762, 0.00719, 0.00677, 0.0064, 0.00607, 0.00578, 0.00555, 0.00537, 0.00523, 0.00511, 0.00499, 0.00486, 0.00469, 0.00445, 0.00414, 0.00373, 0.00322, 0.00263, 0.00196, 0.00123, 0.00049, -0.00024, -0.00091, -0.0015, -0.00196, -0.00227, -0.00242], 'C501': [0.00788, 0.00695, 0.00612, 0.00542, 0.00486, 0.00443, 0.00414, 0.00395, 0.00386, 0.00384, 0.00386, 0.00388, 0.00387, 0.00382, 0.00369, 0.00349, 0.00321, 0.00286, 0.00248, 0.00208, 0.00172, 0.00143, 0.00124, 0.00118, 0.00128, 0.00153, 0.00195, 0.00252, 0.00323, 0.00406, 0.00498, 0.00596, 0.00697, 0.00801, 0.00905, 0.01005, 0.01102, 0.01193, 0.01277, 0.01354, 0.01422, 0.01479, 0.01525, 0.01558, 0.01577, 0.01584, 0.01578, 0.0156, 0.01532, 0.01497, 0.01458, 0.01419, 0.01384, 0.01356, 0.01337, 0.01331, 0.01336, 0.01353, 0.01378, 0.01408, 0.01438, 0.01462, 0.01476, 0.01476, 0.01457, 0.0142, 0.01363, 0.01288, 0.01199, 0.011, 0.00995, 0.0089], 'C502': [0.01259, 0.01218, 0.01164, 0.011, 0.0103, 0.00958, 0.00887, 0.00822, 0.00765, 0.00718, 0.00684, 0.00665, 0.0066, 0.00671, 0.00696, 0.00735, 0.00787, 0.00848, 0.00919, 0.00996, 0.01078, 0.01164, 0.0125, 0.01336, 0.0142, 0.01498, 0.01571, 0.01635, 0.01689, 0.0173, 0.01759, 0.01772, 0.01771, 0.01755, 0.01724, 0.01679, 0.01623, 0.01556, 0.01481, 0.01399, 0.01313, 0.01223, 0.01132, 0.01038, 0.00944, 0.00848, 0.00751, 0.00654, 0.00557, 0.00463, 0.00373, 0.00292, 0.00222, 0.00168, 0.00135, 0.00124, 0.00138, 0.00178, 0.00243, 0.0033, 0.00435, 0.00552, 0.00676, 0.008, 0.00918, 0.01027, 0.01119, 0.01193, 0.01247, 0.01281, 0.01293, 0.01285], 'C503': [0.00717, 0.00729, 0.00746, 0.00767, 0.00789, 0.00809, 0.00825, 0.00836, 0.0084, 0.00837, 0.00827, 0.00811, 0.00791, 0.00767, 0.0074, 0.00711, 0.0068, 0.00646, 0.00609, 0.00568, 0.00523, 0.00473, 0.00419, 0.00361, 0.00301, 0.00241, 0.00182, 0.00126, 0.00075, 0.00032, -2e-05, -0.00026, -0.00039, -0.00039, -0.00026, -0.0, 0.00039, 0.00091, 0.00154, 0.00229, 0.00313, 0.00404, 0.00501, 0.00599, 0.00698, 0.00793, 0.00884, 0.00967, 0.0104, 0.011, 0.01149, 0.01184, 0.01205, 0.01213, 0.01209, 0.01194, 0.01171, 0.0114, 0.01104, 0.01065, 0.01024, 0.00982, 0.00941, 0.009, 0.00861, 0.00825, 0.00792, 0.00764, 0.00741, 0.00724, 0.00714, 0.00712], 'C504': [0.00041, 0.0003, 0.00035, 0.00055, 0.00087, 0.00128, 0.00177, 0.00229, 0.00282, 0.00333, 0.00382, 0.00427, 0.00469, 0.00508, 0.00547, 0.00586, 0.00627, 0.00671, 0.00716, 0.00763, 0.00808, 0.00849, 0.00883, 0.00908, 0.00921, 0.0092, 0.00906, 0.0088, 0.00843, 0.008, 0.00754, 0.0071, 0.0067, 0.00639, 0.0062, 0.00615, 0.00623, 0.00645, 0.00677, 0.00719, 0.00766, 0.00813, 0.00858, 0.00897, 0.00928, 0.00951, 0.00965, 0.00973, 0.00975, 0.00976, 0.00977, 0.00978, 0.00982, 0.00987, 0.00992, 0.00995, 0.00992, 0.00981, 0.00959, 0.00925, 0.00878, 0.00819, 0.00748, 0.00668, 0.00581, 0.00491, 0.00401, 0.00315, 0.00236, 0.00167, 0.0011, 0.00068], 'C505': [0.01056, 0.00985, 0.009, 0.008, 0.00689, 0.00567, 0.00439, 0.00309, 0.0018, 0.00057, -0.00054, -0.00151, -0.00232, -0.00293, -0.00334, -0.00356, -0.0036, -0.00345, -0.00316, -0.00275, -0.00224, -0.00166, -0.00104, -0.00042, 0.00019, 0.00076, 0.00126, 0.00167, 0.00199, 0.00219, 0.00227, 0.00223, 0.00209, 0.00185, 0.00156, 0.00125, 0.00095, 0.00073, 0.00061, 0.00064, 0.00085, 0.00127, 0.00189, 0.00271, 0.0037, 0.00483, 0.00606, 0.00733, 0.0086, 0.00981, 0.01092, 0.01189, 0.0127, 0.01333, 0.01378, 0.01407, 0.01419, 0.0142, 0.01411, 0.01396, 0.01376, 0.01355, 0.01334, 0.01314, 0.01296, 0.01279, 0.01262, 0.01244, 0.01222, 0.01195, 0.01159, 0.01114], 'C506': [0.00927, 0.00915, 0.00914, 0.00924, 0.00945, 0.00978, 0.01019, 0.01068, 0.01121, 0.01177, 0.01231, 0.01281, 0.01325, 0.01361, 0.01386, 0.01403, 0.01409, 0.01406, 0.01395, 0.01378, 0.01355, 0.0133, 0.01304, 0.01279, 0.01255, 0.01235, 0.01219, 0.01209, 0.01204, 0.01205, 0.01212, 0.01224, 0.01239, 0.01257, 0.01274, 0.01291, 0.01304, 0.01315, 0.01322, 0.01327, 0.0133, 0.01333, 0.01337, 0.01342, 0.0135, 0.01361, 0.01374, 0.0139, 0.01406, 0.01421, 0.01436, 0.0145, 0.0146, 0.01468, 0.01472, 0.01472, 0.01468, 0.01459, 0.01445, 0.01426, 0.01401, 0.0137, 0.01334, 0.01293, 0.01248, 0.01201, 0.01152, 0.01104, 0.01058, 0.01016, 0.00979, 0.00949], 'C507': [0.00989, 0.00999, 0.01015, 0.01035, 0.01059, 0.01084, 0.01109, 0.01133, 0.01154, 0.01171, 0.01184, 0.01193, 0.01199, 0.01202, 0.01202, 0.01202, 0.01203, 0.01204, 0.01209, 0.01216, 0.01227, 0.01241, 0.01259, 0.01279, 0.013, 0.01321, 0.0134, 0.01357, 0.01368, 0.01374, 0.01373, 0.01365, 0.0135, 0.01329, 0.01304, 0.01277, 0.01251, 0.01229, 0.01214, 0.01209, 0.01214, 0.01232, 0.01263, 0.01307, 0.01362, 0.01428, 0.01501, 0.01579, 0.01659, 0.01737, 0.0181, 0.01873, 0.01924, 0.01958, 0.01975, 0.01971, 0.01948, 0.01906, 0.01846, 0.01771, 0.01684, 0.0159, 0.01494, 0.01398, 0.01308, 0.01226, 0.01154, 0.01096, 0.0105, 0.01017, 0.00997, 0.00988], 'C508': [0.0034, 0.00376, 0.0042, 0.00472, 0.00533, 0.00602, 0.0068, 0.00763, 0.0085, 0.0094, 0.01031, 0.0112, 0.01205, 0.01285, 0.01357, 0.01421, 0.01477, 0.01522, 0.01558, 0.01584, 0.01601, 0.0161, 0.01608, 0.01598, 0.01579, 0.01551, 0.01512, 0.01464, 0.01406, 0.01339, 0.01263, 0.01182, 0.01095, 0.01006, 0.00919, 0.00837, 0.00762, 0.00698, 0.00648, 0.00615, 0.00601, 0.00606, 0.0063, 0.00671, 0.00727, 0.00794, 0.00869, 0.00946, 0.0102, 0.01084, 0.01135, 0.01166, 0.01178, 0.01167, 0.01133, 0.01079, 0.01007, 0.0092, 0.00826, 0.00729, 0.00633, 0.00544, 0.00466, 0.00401, 0.00349, 0.00311, 0.00286, 0.00273, 0.00271, 0.00277, 0.00291, 0.00312], 'C509': [0.00092, -0.00064, -0.00203, -0.00325, -0.00426, -0.00503, -0.00553, -0.00575, -0.00563, -0.00518, -0.00436, -0.00316, -0.00161, 0.00026, 0.00241, 0.00477, 0.00721, 0.00966, 0.01198, 0.01407, 0.01584, 0.01721, 0.01812, 0.01856, 0.01854, 0.0181, 0.01731, 0.01626, 0.01504, 0.01376, 0.01252, 0.01142, 0.0105, 0.00981, 0.00938, 0.00919, 0.00923, 0.00945, 0.00981, 0.01026, 0.01076, 0.01128, 0.0118, 0.01231, 0.01283, 0.01333, 0.01386, 0.01442, 0.01501, 0.01563, 0.01629, 0.01697, 0.01765, 0.01831, 0.01891, 0.01942, 0.0198, 0.02002, 0.02005, 0.01984, 0.0194, 0.0187, 0.01776, 0.01657, 0.01517, 0.01359, 0.01186, 0.01004, 0.00815, 0.00626, 0.0044, 0.00261], 'C510': [-0.00302, -0.00303, -0.00288, -0.00255, -0.00201, -0.00126, -0.00028, 0.00089, 0.00219, 0.00357, 0.00493, 0.00619, 0.00728, 0.00809, 0.0086, 0.00875, 0.00853, 0.00796, 0.00708, 0.00595, 0.00463, 0.00321, 0.00177, 0.00038, -0.00088, -0.00196, -0.00281, -0.00338, -0.00367, -0.00366, -0.00334, -0.00275, -0.00191, -0.00084, 0.00038, 0.00173, 0.00314, 0.00456, 0.00593, 0.00721, 0.00837, 0.00936, 0.01018, 0.01079, 0.01121, 0.01143, 0.01146, 0.01131, 0.01102, 0.01059, 0.01004, 0.00941, 0.00871, 0.00794, 0.00714, 0.00631, 0.00546, 0.00459, 0.00374, 0.0029, 0.00208, 0.00131, 0.00061, -3e-05, -0.00059, -0.00108, -0.0015, -0.00187, -0.0022, -0.00248, -0.00272, -0.00291], 'C511': [0.01257, 0.01149, 0.01025, 0.00888, 0.00744, 0.00597, 0.00451, 0.00311, 0.00182, 0.00066, -0.00034, -0.00115, -0.00178, -0.00221, -0.00247, -0.00256, -0.0025, -0.0023, -0.00198, -0.00156, -0.00104, -0.00044, 0.00024, 0.00098, 0.0018, 0.00266, 0.00358, 0.00453, 0.00549, 0.00646, 0.0074, 0.00829, 0.0091, 0.0098, 0.01038, 0.01084, 0.01116, 0.01135, 0.01144, 0.01145, 0.0114, 0.01134, 0.01127, 0.01122, 0.01121, 0.01123, 0.01128, 0.01136, 0.01144, 0.01153, 0.01161, 0.01168, 0.01173, 0.01178, 0.01184, 0.01192, 0.01203, 0.0122, 0.01242, 0.0127, 0.01303, 0.01341, 0.01381, 0.01421, 0.01457, 0.01485, 0.01504, 0.01509, 0.01497, 0.01468, 0.01418, 0.01347], 'C512': [0.0096, 0.01009, 0.01057, 0.011, 0.01137, 0.01164, 0.01179, 0.0118, 0.01164, 0.01131, 0.0108, 0.01012, 0.00928, 0.00829, 0.00718, 0.00597, 0.00472, 0.00343, 0.00215, 0.00092, -0.00025, -0.00133, -0.00229, -0.00312, -0.0038, -0.00433, -0.00469, -0.00487, -0.00488, -0.00471, -0.00436, -0.00382, -0.00309, -0.00217, -0.00108, 0.00019, 0.00161, 0.00316, 0.0048, 0.00651, 0.00822, 0.00987, 0.01142, 0.0128, 0.01396, 0.01486, 0.01545, 0.01573, 0.0157, 0.01537, 0.01479, 0.01399, 0.01305, 0.01203, 0.01099, 0.00999, 0.00907, 0.00828, 0.00764, 0.00715, 0.00681, 0.00661, 0.00654, 0.00657, 0.00669, 0.00688, 0.00714, 0.00744, 0.0078, 0.0082, 0.00864, 0.00911], 'C513': [0.01438, 0.0144, 0.01435, 0.01421, 0.01399, 0.01369, 0.01332, 0.01288, 0.01238, 0.01182, 0.01122, 0.01059, 0.00993, 0.00928, 0.00864, 0.00803, 0.00748, 0.00699, 0.00657, 0.00622, 0.00592, 0.00566, 0.0054, 0.00513, 0.00479, 0.00438, 0.00387, 0.00324, 0.00252, 0.00172, 0.00088, 5e-05, -0.00071, -0.00134, -0.00177, -0.00196, -0.00185, -0.00142, -0.00067, 0.00039, 0.00171, 0.00324, 0.0049, 0.0066, 0.00826, 0.00979, 0.01114, 0.01225, 0.01309, 0.01365, 0.01396, 0.01404, 0.01392, 0.01368, 0.01335, 0.01298, 0.01263, 0.01232, 0.01207, 0.01192, 0.01185, 0.01188, 0.01199, 0.01217, 0.01241, 0.01269, 0.013, 0.01332, 0.01362, 0.01389, 0.01412, 0.01428], 'D402': [0.00833, 0.00855, 0.00887, 0.00924, 0.00962, 0.00994, 0.01016, 0.01022, 0.0101, 0.00978, 0.00926, 0.00858, 0.00776, 0.0069, 0.00604, 0.00527, 0.00466, 0.00427, 0.00411, 0.00423, 0.00458, 0.00513, 0.00583, 0.0066, 0.00737, 0.00806, 0.00861, 0.00901, 0.00921, 0.00925, 0.00915, 0.00897, 0.00878, 0.00864, 0.00863, 0.00879, 0.00918, 0.0098, 0.01066, 0.01173, 0.01296, 0.01431, 0.01571, 0.01709, 0.0184, 0.01958, 0.02059, 0.02138, 0.02195, 0.02229, 0.02239, 0.02228, 0.02197, 0.02149, 0.02087, 0.02013, 0.0193, 0.0184, 0.01746, 0.01649, 0.0155, 0.01451, 0.01354, 0.01258, 0.01168, 0.01083, 0.01007, 0.00942, 0.0089, 0.00852, 0.0083, 0.00824], 'D403': [0.00734, 0.00705, 0.00668, 0.00626, 0.00582, 0.0054, 0.00501, 0.00468, 0.00443, 0.00425, 0.00412, 0.00402, 0.00392, 0.0038, 0.00363, 0.00342, 0.00314, 0.00282, 0.00248, 0.00214, 0.00184, 0.00161, 0.0015, 0.00152, 0.0017, 0.00203, 0.00253, 0.00315, 0.00387, 0.00467, 0.00548, 0.00628, 0.00702, 0.00769, 0.00825, 0.0087, 0.00904, 0.00927, 0.0094, 0.00944, 0.00942, 0.00933, 0.00919, 0.009, 0.00877, 0.00849, 0.00817, 0.00783, 0.00746, 0.00709, 0.00673, 0.0064, 0.00612, 0.00589, 0.00573, 0.00563, 0.0056, 0.00563, 0.0057, 0.00581, 0.00595, 0.00612, 0.0063, 0.00651, 0.00673, 0.00695, 0.00717, 0.00737, 0.00752, 0.00762, 0.00763, 0.00754]}, 'scan_amp_C7': {'A405': [0.0101, 0.00925, 0.00841, 0.0076, 0.00682, 0.00608, 0.00538, 0.00472, 0.00408, 0.00346, 0.00286, 0.00225, 0.00162, 0.00098, 0.00032, -0.00038, -0.00112, -0.00189, -0.00271, -0.00356, -0.00443, -0.00531, -0.00617, -0.00699, -0.00772, -0.00832, -0.00875, -0.00897, -0.00895, -0.00866, -0.00808, -0.00723, -0.00611, -0.00474, -0.00317, -0.00143, 0.00045, 0.0024, 0.00438, 0.00638, 0.00831, 0.01017, 0.01191, 0.0135, 0.01493, 0.01618, 0.01724, 0.0181, 0.01878, 0.01927, 0.01959, 0.01976, 0.01979, 0.01971, 0.01954, 0.01929, 0.019, 0.01866, 0.01831, 0.01794, 0.01756, 0.01717, 0.01676, 0.01631, 0.01583, 0.0153, 0.01471, 0.01407, 0.01336, 0.0126, 0.01179, 0.01096], 'A407': [0.00427, 0.00445, 0.00483, 0.00537, 0.00606, 0.00685, 0.00773, 0.00865, 0.00959, 0.01052, 0.01142, 0.01225, 0.01301, 0.01365, 0.01419, 0.0146, 0.01487, 0.01499, 0.01495, 0.01474, 0.01434, 0.01375, 0.01295, 0.01195, 0.01075, 0.00936, 0.00781, 0.00614, 0.00439, 0.00264, 0.00093, -0.00066, -0.0021, -0.00332, -0.00428, -0.00496, -0.00533, -0.0054, -0.00519, -0.0047, -0.00397, -0.00305, -0.00197, -0.00077, 0.0005, 0.00182, 0.00313, 0.00443, 0.00567, 0.00684, 0.00792, 0.0089, 0.00975, 0.01047, 0.01104, 0.01146, 0.01172, 0.01182, 0.01177, 0.01155, 0.01119, 0.0107, 0.01008, 0.00937, 0.00858, 0.00776, 0.00694, 0.00616, 0.00547, 0.0049, 0.0045, 0.00428], 'A410': [-0.00086, -0.00038, 7e-05, 0.00049, 0.00091, 0.00135, 0.00183, 0.00239, 0.00303, 0.00376, 0.00457, 0.00545, 0.00636, 0.00724, 0.00808, 0.00879, 0.00935, 0.00969, 0.0098, 0.00964, 0.00921, 0.00851, 0.00757, 0.00643, 0.00514, 0.00378, 0.00241, 0.00111, -5e-05, -0.00099, -0.00167, -0.00206, -0.00215, -0.00194, -0.00147, -0.00079, 5e-05, 0.00098, 0.00193, 0.00284, 0.00365, 0.00431, 0.00481, 0.00512, 0.00523, 0.00515, 0.0049, 0.00451, 0.00399, 0.00338, 0.0027, 0.00196, 0.0012, 0.00043, -0.00035, -0.0011, -0.00183, -0.00251, -0.00313, -0.00366, -0.00409, -0.00441, -0.00459, -0.00463, -0.00454, -0.00431, -0.00397, -0.00353, -0.00303, -0.00248, -0.00192, -0.00137], 'B603': [0.01075, 0.00985, 0.00901, 0.00828, 0.00771, 0.00732, 0.00711, 0.00709, 0.00724, 0.00753, 0.0079, 0.00832, 0.00873, 0.00908, 0.00932, 0.00942, 0.00936, 0.00912, 0.00872, 0.00818, 0.00752, 0.00678, 0.006, 0.00523, 0.00449, 0.00384, 0.00329, 0.00287, 0.00258, 0.00244, 0.00244, 0.00256, 0.00281, 0.00316, 0.00359, 0.00409, 0.00464, 0.00522, 0.0058, 0.00638, 0.00693, 0.00745, 0.00792, 0.00832, 0.00864, 0.0089, 0.00909, 0.00922, 0.00931, 0.00936, 0.00941, 0.00948, 0.00959, 0.00977, 0.01002, 0.01037, 0.01081, 0.01134, 0.01194, 0.01258, 0.01322, 0.01383, 0.01435, 0.01475, 0.01499, 0.01505, 0.01492, 0.01458, 0.01406, 0.01338, 0.01257, 0.01168], 'B604': [0.00504, 0.00596, 0.00687, 0.00773, 0.00852, 0.00921, 0.00979, 0.01022, 0.01051, 0.01063, 0.01058, 0.01034, 0.00992, 0.00933, 0.00858, 0.00769, 0.0067, 0.00566, 0.00462, 0.00365, 0.00278, 0.00208, 0.0016, 0.00137, 0.0014, 0.00169, 0.00223, 0.00298, 0.00387, 0.00487, 0.00588, 0.00684, 0.00769, 0.00837, 0.00883, 0.00904, 0.009, 0.00871, 0.00819, 0.0075, 0.00666, 0.00575, 0.00481, 0.00391, 0.00307, 0.00235, 0.00177, 0.00135, 0.00107, 0.00094, 0.00093, 0.001, 0.00113, 0.00128, 0.00142, 0.00151, 0.00154, 0.00151, 0.00141, 0.00127, 0.00108, 0.0009, 0.00075, 0.00064, 0.00064, 0.00075, 0.00098, 0.00136, 0.00189, 0.00254, 0.0033, 0.00414], 'B605': [0.01748, 0.01751, 0.01742, 0.01721, 0.0169, 0.01651, 0.01608, 0.01565, 0.01526, 0.01494, 0.01472, 0.01462, 0.01464, 0.01477, 0.01498, 0.01525, 0.01553, 0.01578, 0.01597, 0.01605, 0.01598, 0.01574, 0.01529, 0.01463, 0.01374, 0.01264, 0.01134, 0.00984, 0.00822, 0.00649, 0.0047, 0.00292, 0.00118, -0.00047, -0.00199, -0.00333, -0.00448, -0.00541, -0.00612, -0.0066, -0.00686, -0.0069, -0.00674, -0.00641, -0.0059, -0.00525, -0.00446, -0.00356, -0.00256, -0.00149, -0.00036, 0.00081, 0.002, 0.00319, 0.00439, 0.00555, 0.00669, 0.00779, 0.00884, 0.00984, 0.0108, 0.01169, 0.01253, 0.0133, 0.01403, 0.01469, 0.0153, 0.01585, 0.01634, 0.01675, 0.01709, 0.01734], 'B606': [0.00035, 0.00022, 0.00022, 0.00033, 0.00052, 0.00074, 0.00096, 0.00116, 0.00129, 0.00137, 0.00138, 0.00135, 0.00131, 0.00127, 0.00127, 0.00133, 0.00145, 0.00164, 0.00189, 0.00217, 0.00244, 0.00268, 0.00286, 0.00294, 0.00293, 0.00282, 0.0026, 0.00231, 0.00195, 0.00155, 0.00113, 0.00071, 0.0003, -0.0001, -0.00048, -0.00083, -0.00117, -0.00149, -0.00178, -0.00204, -0.00227, -0.00245, -0.00257, -0.00262, -0.00257, -0.00241, -0.00214, -0.00174, -0.00122, -0.00058, 0.00017, 0.001, 0.00189, 0.00279, 0.00369, 0.00452, 0.00527, 0.00589, 0.00636, 0.00665, 0.00675, 0.00666, 0.00638, 0.00592, 0.00533, 0.00462, 0.00385, 0.00307, 0.00232, 0.00164, 0.00107, 0.00064], 'B607': [0.01662, 0.01572, 0.01471, 0.01359, 0.01239, 0.01116, 0.00993, 0.00876, 0.00769, 0.00678, 0.00608, 0.00563, 0.00543, 0.0055, 0.00579, 0.00627, 0.00688, 0.00752, 0.00812, 0.00861, 0.00892, 0.00901, 0.00887, 0.0085, 0.00793, 0.0072, 0.00637, 0.00548, 0.0046, 0.00373, 0.00291, 0.00215, 0.00143, 0.00075, 8e-05, -0.00058, -0.00125, -0.00192, -0.00259, -0.00323, -0.0038, -0.00428, -0.00463, -0.00481, -0.00479, -0.00454, -0.00406, -0.00334, -0.00239, -0.00124, 9e-05, 0.00158, 0.00316, 0.00482, 0.00652, 0.00819, 0.00983, 0.01141, 0.01288, 0.01423, 0.01543, 0.01647, 0.01734, 0.01803, 0.01854, 0.01887, 0.01902, 0.019, 0.01883, 0.0185, 0.01802, 0.01739], 'B608': [0.00953, 0.00946, 0.00944, 0.00947, 0.0095, 0.00951, 0.00947, 0.00936, 0.00914, 0.00881, 0.00833, 0.00772, 0.00695, 0.00604, 0.00501, 0.00388, 0.00271, 0.00153, 0.00039, -0.00063, -0.0015, -0.00217, -0.00259, -0.00277, -0.00268, -0.00234, -0.00178, -0.00104, -0.00017, 0.00077, 0.00174, 0.00265, 0.00348, 0.00417, 0.00469, 0.00503, 0.00519, 0.00518, 0.00503, 0.00477, 0.00446, 0.00414, 0.00387, 0.00369, 0.00365, 0.00376, 0.00406, 0.00454, 0.0052, 0.00601, 0.00694, 0.00797, 0.00904, 0.0101, 0.01112, 0.01204, 0.01282, 0.01345, 0.0139, 0.01415, 0.01422, 0.01411, 0.01384, 0.01343, 0.01293, 0.01237, 0.01179, 0.01123, 0.01072, 0.01028, 0.00994, 0.00969], 'B609': [0.01116, 0.01191, 0.01246, 0.0128, 0.01289, 0.01274, 0.01236, 0.01179, 0.01105, 0.01017, 0.00922, 0.00823, 0.00724, 0.0063, 0.00544, 0.00468, 0.00404, 0.00353, 0.00314, 0.00288, 0.00273, 0.00266, 0.00266, 0.00272, 0.0028, 0.00289, 0.00299, 0.00309, 0.00319, 0.00329, 0.0034, 0.00354, 0.0037, 0.0039, 0.00413, 0.00437, 0.00461, 0.00482, 0.00496, 0.005, 0.0049, 0.00463, 0.00417, 0.00351, 0.00266, 0.00164, 0.0005, -0.00072, -0.00196, -0.00313, -0.00419, -0.00507, -0.00572, -0.00612, -0.00624, -0.0061, -0.0057, -0.00508, -0.00429, -0.00335, -0.00232, -0.00123, -0.00011, 0.00104, 0.0022, 0.00337, 0.00455, 0.00574, 0.00692, 0.00809, 0.00921, 0.01024], 'B610': [-0.00747, -0.00776, -0.00826, -0.00893, -0.00976, -0.01071, -0.01171, -0.0127, -0.0136, -0.01434, -0.01484, -0.01503, -0.01489, -0.01437, -0.01348, -0.01223, -0.01067, -0.00885, -0.00683, -0.00468, -0.00252, -0.00037, 0.00167, 0.00353, 0.00518, 0.00657, 0.00769, 0.00854, 0.00915, 0.00953, 0.00972, 0.00978, 0.00976, 0.00969, 0.00962, 0.00959, 0.00962, 0.00972, 0.00988, 0.0101, 0.01034, 0.01059, 0.0108, 0.01093, 0.01095, 0.01083, 0.01052, 0.01002, 0.0093, 0.00838, 0.00726, 0.00594, 0.00449, 0.00292, 0.00129, -0.00035, -0.00194, -0.00345, -0.00481, -0.00599, -0.00696, -0.00771, -0.00823, -0.00853, -0.00863, -0.00857, -0.00838, -0.00812, -0.00783, -0.00758, -0.0074, -0.00735], 'B611': [0.00325, 0.00318, 0.00311, 0.00303, 0.00291, 0.00273, 0.00247, 0.00211, 0.00165, 0.0011, 0.00048, -0.00019, -0.00087, -0.00153, -0.00215, -0.00269, -0.00312, -0.00344, -0.00364, -0.00372, -0.00372, -0.00364, -0.00351, -0.00336, -0.00321, -0.00306, -0.00294, -0.00282, -0.0027, -0.00255, -0.00237, -0.00212, -0.00178, -0.00134, -0.00079, -0.00012, 0.00063, 0.00148, 0.00238, 0.00332, 0.00426, 0.00517, 0.00604, 0.00682, 0.00751, 0.00808, 0.00852, 0.00883, 0.00901, 0.00906, 0.00899, 0.00883, 0.0086, 0.00831, 0.00799, 0.00767, 0.00734, 0.00703, 0.00673, 0.00646, 0.00618, 0.00592, 0.00564, 0.00536, 0.00506, 0.00476, 0.00445, 0.00416, 0.00389, 0.00367, 0.00348, 0.00334], 'B612': [0.01135, 0.01159, 0.01187, 0.01217, 0.01247, 0.01271, 0.01284, 0.01283, 0.0126, 0.01213, 0.01142, 0.01045, 0.00922, 0.00776, 0.00612, 0.00432, 0.00243, 0.00052, -0.00137, -0.00318, -0.00483, -0.00627, -0.00744, -0.00829, -0.00879, -0.00889, -0.00861, -0.00793, -0.0069, -0.00555, -0.00393, -0.00216, -0.00027, 0.00166, 0.00353, 0.00531, 0.00694, 0.00837, 0.00959, 0.01059, 0.01135, 0.01188, 0.01221, 0.01233, 0.01228, 0.01208, 0.01178, 0.01139, 0.01097, 0.01053, 0.01013, 0.00976, 0.00948, 0.00928, 0.00917, 0.00915, 0.0092, 0.00932, 0.0095, 0.0097, 0.00992, 0.01014, 0.01034, 0.01052, 0.01066, 0.01076, 0.01083, 0.01089, 0.01093, 0.01097, 0.01106, 0.01118], 'B613': [0.00577, 0.00639, 0.00709, 0.00786, 0.00865, 0.00944, 0.01017, 0.01082, 0.01135, 0.01176, 0.01203, 0.01218, 0.0122, 0.01212, 0.01196, 0.01172, 0.01142, 0.01106, 0.01063, 0.01014, 0.00955, 0.00886, 0.00806, 0.00712, 0.00606, 0.00488, 0.00361, 0.00226, 0.00089, -0.00046, -0.00176, -0.00294, -0.00398, -0.00484, -0.00548, -0.00592, -0.00614, -0.00615, -0.00599, -0.00566, -0.00522, -0.00467, -0.00406, -0.00342, -0.00277, -0.00215, -0.00157, -0.00105, -0.00062, -0.00028, -1e-05, 0.00017, 0.00028, 0.00036, 0.0004, 0.00047, 0.00056, 0.00071, 0.00093, 0.0012, 0.00154, 0.00192, 0.00231, 0.0027, 0.00305, 0.00337, 0.00366, 0.00392, 0.00419, 0.00448, 0.00482, 0.00525], 'B614': [0.02189, 0.02118, 0.02036, 0.01944, 0.01844, 0.01735, 0.01618, 0.01495, 0.01368, 0.01238, 0.01107, 0.00977, 0.00849, 0.00723, 0.006, 0.00478, 0.00359, 0.00243, 0.00132, 0.00027, -0.00069, -0.00154, -0.00223, -0.00274, -0.00304, -0.00312, -0.00296, -0.00258, -0.00199, -0.0012, -0.00025, 0.00083, 0.002, 0.00323, 0.00449, 0.00572, 0.0069, 0.00798, 0.00895, 0.00977, 0.01043, 0.01091, 0.01122, 0.01137, 0.01138, 0.01127, 0.0111, 0.0109, 0.01072, 0.01061, 0.01061, 0.01076, 0.01108, 0.01159, 0.01229, 0.01318, 0.01423, 0.01541, 0.01666, 0.01793, 0.01918, 0.02034, 0.02137, 0.02223, 0.02291, 0.02338, 0.02365, 0.02373, 0.02364, 0.02339, 0.02301, 0.02251], 'B616': [0.00653, 0.00669, 0.00689, 0.00711, 0.0073, 0.00745, 0.00754, 0.00755, 0.00748, 0.00733, 0.00712, 0.00686, 0.00657, 0.00626, 0.00596, 0.00568, 0.00543, 0.00524, 0.0051, 0.00504, 0.00505, 0.00513, 0.00529, 0.00552, 0.0058, 0.00611, 0.00644, 0.00674, 0.007, 0.00718, 0.00726, 0.00722, 0.00705, 0.00675, 0.00632, 0.00579, 0.00517, 0.00449, 0.00378, 0.00308, 0.00242, 0.00183, 0.00132, 0.00092, 0.00064, 0.00048, 0.00045, 0.00053, 0.00072, 0.00102, 0.0014, 0.00186, 0.00239, 0.00296, 0.00357, 0.00418, 0.00479, 0.00537, 0.0059, 0.00635, 0.00672, 0.00698, 0.00714, 0.00719, 0.00715, 0.00704, 0.00689, 0.00672, 0.00656, 0.00645, 0.00641, 0.00643], 'B617': [-0.00123, -0.0023, -0.00303, -0.00338, -0.00335, -0.00295, -0.0022, -0.00114, 0.00018, 0.00171, 0.0034, 0.00521, 0.00708, 0.00894, 0.01078, 0.01255, 0.01418, 0.01566, 0.01692, 0.01794, 0.01868, 0.01913, 0.01928, 0.01914, 0.01873, 0.01808, 0.01725, 0.01628, 0.01523, 0.01416, 0.01312, 0.01216, 0.0113, 0.01056, 0.00997, 0.00952, 0.00922, 0.00907, 0.00906, 0.00919, 0.00946, 0.00987, 0.01042, 0.01108, 0.01187, 0.01275, 0.01372, 0.01472, 0.01575, 0.01674, 0.01769, 0.01854, 0.01927, 0.01986, 0.02027, 0.0205, 0.02053, 0.02035, 0.01996, 0.01936, 0.01855, 0.01752, 0.0163, 0.01486, 0.01324, 0.01146, 0.00956, 0.00759, 0.00559, 0.00364, 0.00181, 0.00017], 'C501': [0.01483, 0.01423, 0.01359, 0.01296, 0.01238, 0.01186, 0.01142, 0.01104, 0.01072, 0.0104, 0.01007, 0.00967, 0.00917, 0.00854, 0.00775, 0.0068, 0.00569, 0.00443, 0.00305, 0.00158, 6e-05, -0.00146, -0.00293, -0.00432, -0.00557, -0.00664, -0.0075, -0.00813, -0.00851, -0.00864, -0.00854, -0.00822, -0.00772, -0.00707, -0.00631, -0.00549, -0.00464, -0.00381, -0.00301, -0.00227, -0.00161, -0.00102, -0.0005, -6e-05, 0.00032, 0.00065, 0.00096, 0.00124, 0.00153, 0.00183, 0.00217, 0.00256, 0.00304, 0.00361, 0.00428, 0.00507, 0.00597, 0.00698, 0.00806, 0.0092, 0.01037, 0.01151, 0.0126, 0.01359, 0.01446, 0.01516, 0.01569, 0.016, 0.01612, 0.01604, 0.01578, 0.01537], 'C502': [0.0007, 0.00081, 0.00101, 0.00132, 0.00169, 0.00212, 0.00258, 0.00307, 0.00357, 0.00406, 0.00454, 0.005, 0.00543, 0.00582, 0.00616, 0.00644, 0.00662, 0.0067, 0.00664, 0.00645, 0.00611, 0.00562, 0.00499, 0.00423, 0.00338, 0.00248, 0.00154, 0.00062, -0.00027, -0.00108, -0.0018, -0.00241, -0.00291, -0.00329, -0.00356, -0.00374, -0.00383, -0.00386, -0.00385, -0.00383, -0.00383, -0.00387, -0.00396, -0.00412, -0.00433, -0.0046, -0.00488, -0.00516, -0.00539, -0.00553, -0.00555, -0.00543, -0.00513, -0.00467, -0.00406, -0.00332, -0.0025, -0.00163, -0.00076, 3e-05, 0.00072, 0.00127, 0.00165, 0.00187, 0.00193, 0.00185, 0.00167, 0.00144, 0.00118, 0.00095, 0.00078, 0.00069], 'C503': [-0.00259, -0.00401, -0.00527, -0.00636, -0.00724, -0.0079, -0.00832, -0.00851, -0.00848, -0.00823, -0.00779, -0.00718, -0.00644, -0.0056, -0.00469, -0.00375, -0.00281, -0.00189, -0.00102, -0.00019, 0.00058, 0.00129, 0.00196, 0.00258, 0.00318, 0.00376, 0.00431, 0.00486, 0.0054, 0.00591, 0.00638, 0.00681, 0.00716, 0.00743, 0.00759, 0.00763, 0.00754, 0.00734, 0.00702, 0.00663, 0.00618, 0.00573, 0.0053, 0.00493, 0.00467, 0.00452, 0.00453, 0.00469, 0.00502, 0.00549, 0.0061, 0.00683, 0.00764, 0.0085, 0.00936, 0.01019, 0.01092, 0.01152, 0.01196, 0.01218, 0.01216, 0.0119, 0.01138, 0.01061, 0.0096, 0.00839, 0.007, 0.00549, 0.00388, 0.00223, 0.00057, -0.00105], 'C504': [-0.00826, -0.00767, -0.0067, -0.00537, -0.00376, -0.00194, -1e-05, 0.00192, 0.00376, 0.00541, 0.00679, 0.00783, 0.0085, 0.0088, 0.00873, 0.00835, 0.00772, 0.00693, 0.00605, 0.00518, 0.00439, 0.00373, 0.00326, 0.003, 0.00295, 0.00308, 0.00338, 0.00379, 0.00426, 0.00475, 0.00521, 0.00558, 0.00584, 0.00596, 0.00594, 0.00578, 0.00548, 0.00507, 0.00457, 0.00401, 0.00344, 0.00287, 0.00233, 0.00187, 0.0015, 0.00123, 0.00108, 0.00105, 0.00114, 0.00134, 0.00162, 0.00197, 0.00236, 0.00275, 0.00311, 0.00339, 0.00357, 0.0036, 0.00345, 0.0031, 0.00253, 0.00175, 0.00077, -0.00039, -0.00168, -0.00304, -0.0044, -0.00567, -0.00678, -0.00766, -0.00823, -0.00844], 'C505': [0.00412, 0.00438, 0.00463, 0.00486, 0.0051, 0.00536, 0.00567, 0.00604, 0.00649, 0.00703, 0.00765, 0.00834, 0.00909, 0.00985, 0.01061, 0.01132, 0.01194, 0.01244, 0.01279, 0.01298, 0.01301, 0.01287, 0.01258, 0.01217, 0.01169, 0.01116, 0.01063, 0.01014, 0.00971, 0.00935, 0.00908, 0.00888, 0.00874, 0.00863, 0.00852, 0.00838, 0.00818, 0.00791, 0.00755, 0.00711, 0.00659, 0.00602, 0.00542, 0.00482, 0.00424, 0.00371, 0.00328, 0.00294, 0.00272, 0.00262, 0.00263, 0.00272, 0.00288, 0.00308, 0.00328, 0.00344, 0.00354, 0.00357, 0.00353, 0.00341, 0.00324, 0.00304, 0.00285, 0.0027, 0.00261, 0.00259, 0.00265, 0.0028, 0.00301, 0.00327, 0.00355, 0.00384], 'C506': [0.01941, 0.0204, 0.02133, 0.02221, 0.02301, 0.02372, 0.02431, 0.02474, 0.02497, 0.02497, 0.0247, 0.02414, 0.02324, 0.02203, 0.02049, 0.01865, 0.01658, 0.01431, 0.01193, 0.0095, 0.00709, 0.00474, 0.00252, 0.00045, -0.00145, -0.00317, -0.00471, -0.00609, -0.00732, -0.0084, -0.00936, -0.01018, -0.01089, -0.01147, -0.01192, -0.01223, -0.01241, -0.01246, -0.01239, -0.0122, -0.01191, -0.01155, -0.01113, -0.01068, -0.01022, -0.00977, -0.00933, -0.00891, -0.00851, -0.0081, -0.00767, -0.0072, -0.00665, -0.00599, -0.00521, -0.00428, -0.00321, -0.00196, -0.00059, 0.00091, 0.0025, 0.00415, 0.00582, 0.00749, 0.00911, 0.01067, 0.01216, 0.01355, 0.01487, 0.01611, 0.01727, 0.01837], 'C507': [0.00862, 0.00794, 0.00734, 0.00685, 0.00651, 0.00635, 0.0064, 0.00666, 0.00712, 0.00775, 0.00849, 0.00928, 0.01004, 0.0107, 0.01118, 0.01145, 0.01145, 0.0112, 0.01069, 0.00996, 0.00907, 0.00807, 0.00702, 0.00598, 0.00501, 0.00415, 0.00343, 0.00287, 0.00246, 0.0022, 0.00208, 0.00207, 0.00214, 0.00228, 0.00245, 0.00263, 0.00282, 0.00302, 0.0032, 0.00338, 0.00356, 0.00375, 0.00395, 0.00417, 0.00441, 0.00469, 0.00501, 0.00539, 0.00583, 0.00633, 0.0069, 0.00755, 0.00826, 0.00901, 0.0098, 0.01059, 0.01136, 0.01207, 0.0127, 0.0132, 0.01357, 0.01378, 0.01384, 0.01374, 0.01351, 0.01314, 0.01267, 0.01211, 0.01148, 0.01079, 0.01007, 0.00934], 'C508': [-0.00443, -0.00439, -0.00415, -0.0037, -0.00309, -0.00237, -0.00159, -0.0008, -9e-05, 0.0005, 0.00091, 0.00111, 0.00106, 0.00077, 0.00021, -0.00056, -0.00154, -0.00265, -0.00385, -0.00507, -0.00623, -0.00729, -0.00816, -0.0088, -0.00915, -0.00921, -0.00894, -0.00837, -0.00753, -0.00643, -0.00514, -0.00372, -0.0022, -0.00064, 0.00091, 0.00243, 0.00388, 0.00524, 0.00651, 0.00768, 0.00874, 0.00968, 0.01052, 0.01124, 0.01183, 0.01231, 0.01267, 0.01291, 0.01304, 0.01305, 0.01294, 0.01273, 0.01241, 0.01199, 0.01147, 0.01085, 0.01013, 0.00932, 0.00842, 0.00745, 0.00639, 0.00529, 0.00414, 0.00296, 0.00177, 0.0006, -0.00052, -0.00154, -0.00247, -0.00325, -0.00385, -0.00425], 'C509': [0.00443, 0.00524, 0.00616, 0.00718, 0.00827, 0.00943, 0.01064, 0.01186, 0.01308, 0.01429, 0.01543, 0.01648, 0.0174, 0.01815, 0.01869, 0.01899, 0.01901, 0.01873, 0.01813, 0.01721, 0.01598, 0.01446, 0.0127, 0.01074, 0.00863, 0.00646, 0.00428, 0.00214, 0.00016, -0.00164, -0.0032, -0.00446, -0.00542, -0.00606, -0.00638, -0.00641, -0.00617, -0.0057, -0.00506, -0.00429, -0.00343, -0.00255, -0.00168, -0.00086, -0.00011, 0.00055, 0.0011, 0.00155, 0.0019, 0.00214, 0.0023, 0.00236, 0.00236, 0.00229, 0.00218, 0.00202, 0.00185, 0.00166, 0.00149, 0.00133, 0.0012, 0.00112, 0.00109, 0.00111, 0.00119, 0.00133, 0.00154, 0.00181, 0.00216, 0.00259, 0.0031, 0.00371], 'C510': [0.02062, 0.02036, 0.0196, 0.01838, 0.01675, 0.01481, 0.01263, 0.01033, 0.00801, 0.00576, 0.00367, 0.00182, 0.00025, -0.00099, -0.00189, -0.00244, -0.00263, -0.00249, -0.00202, -0.00125, -0.00023, 0.00102, 0.00243, 0.00394, 0.00551, 0.00706, 0.00851, 0.00985, 0.011, 0.01193, 0.01261, 0.01301, 0.01316, 0.01304, 0.01266, 0.01207, 0.01125, 0.01027, 0.00915, 0.00791, 0.00659, 0.00524, 0.00387, 0.00254, 0.00127, 8e-05, -0.00098, -0.00189, -0.00265, -0.00322, -0.0036, -0.0038, -0.00381, -0.00365, -0.0033, -0.00279, -0.00211, -0.00126, -0.00024, 0.00095, 0.00233, 0.00388, 0.00559, 0.00745, 0.00942, 0.01144, 0.01346, 0.01536, 0.0171, 0.01857, 0.01969, 0.02039], 'C511': [-0.00336, -0.00385, -0.00415, -0.00426, -0.00419, -0.00397, -0.00362, -0.00319, -0.00273, -0.00228, -0.00188, -0.00157, -0.00138, -0.00131, -0.00136, -0.00151, -0.00174, -0.00199, -0.00223, -0.00238, -0.00241, -0.00227, -0.00192, -0.00134, -0.00052, 0.00053, 0.0018, 0.00324, 0.0048, 0.00645, 0.00811, 0.00974, 0.01128, 0.01268, 0.01391, 0.01494, 0.01573, 0.01628, 0.01658, 0.01665, 0.01649, 0.01614, 0.01561, 0.01495, 0.0142, 0.0134, 0.0126, 0.01182, 0.01111, 0.01048, 0.00996, 0.00953, 0.00921, 0.00897, 0.00878, 0.00862, 0.00845, 0.00824, 0.00796, 0.0076, 0.00713, 0.00654, 0.00584, 0.00503, 0.00412, 0.00314, 0.00211, 0.00106, 1e-05, -0.00099, -0.00191, -0.00271], 'C512': [0.00153, 0.00015, -0.00115, -0.00231, -0.00328, -0.00404, -0.00455, -0.00483, -0.00487, -0.00471, -0.00439, -0.00397, -0.00348, -0.003, -0.00256, -0.00221, -0.00198, -0.00189, -0.00194, -0.00211, -0.00239, -0.00274, -0.00311, -0.00345, -0.0037, -0.00383, -0.00378, -0.00354, -0.00307, -0.00239, -0.0015, -0.00045, 0.00074, 0.00202, 0.0033, 0.00454, 0.00568, 0.00667, 0.00748, 0.00808, 0.00848, 0.00869, 0.00873, 0.00863, 0.00844, 0.00821, 0.00796, 0.00773, 0.00755, 0.00744, 0.0074, 0.00744, 0.00755, 0.00772, 0.00795, 0.00821, 0.0085, 0.00879, 0.00908, 0.00934, 0.00956, 0.0097, 0.00975, 0.00968, 0.00946, 0.00907, 0.00848, 0.0077, 0.00673, 0.00559, 0.00431, 0.00295], 'C513': [0.00585, 0.00658, 0.00737, 0.0082, 0.00904, 0.00984, 0.01059, 0.01123, 0.01174, 0.01212, 0.01234, 0.0124, 0.01231, 0.01207, 0.01171, 0.01122, 0.01065, 0.01001, 0.00932, 0.00861, 0.0079, 0.00722, 0.00658, 0.00601, 0.00553, 0.00515, 0.00487, 0.0047, 0.00462, 0.00464, 0.00472, 0.00483, 0.00496, 0.00507, 0.00514, 0.00512, 0.005, 0.00477, 0.00441, 0.00392, 0.0033, 0.00257, 0.00176, 0.00089, -1e-05, -0.0009, -0.00174, -0.00249, -0.00313, -0.00361, -0.00394, -0.00409, -0.00406, -0.00387, -0.00352, -0.00303, -0.00245, -0.0018, -0.00112, -0.00042, 0.00024, 0.00086, 0.00142, 0.00191, 0.00234, 0.00272, 0.00306, 0.0034, 0.00376, 0.00416, 0.00464, 0.0052], 'D402': [0.01168, 0.01233, 0.0129, 0.01344, 0.01398, 0.01452, 0.01506, 0.01561, 0.01613, 0.01659, 0.01697, 0.01722, 0.01732, 0.01726, 0.01703, 0.01663, 0.0161, 0.01545, 0.01472, 0.01394, 0.01317, 0.01242, 0.01174, 0.01116, 0.01071, 0.01042, 0.01031, 0.01039, 0.01067, 0.01114, 0.0118, 0.01261, 0.01355, 0.01457, 0.01562, 0.01665, 0.01761, 0.01845, 0.01913, 0.01962, 0.01989, 0.01993, 0.01973, 0.01928, 0.01859, 0.01766, 0.0165, 0.01512, 0.01356, 0.01184, 0.01, 0.00809, 0.00619, 0.00434, 0.00265, 0.00116, -5e-05, -0.00095, -0.00147, -0.00161, -0.00138, -0.00081, 7e-05, 0.00119, 0.00248, 0.00386, 0.00527, 0.00664, 0.00791, 0.00907, 0.01007, 0.01094], 'D403': [0.01095, 0.00984, 0.00871, 0.00759, 0.00651, 0.0055, 0.00462, 0.00388, 0.00332, 0.00297, 0.00284, 0.00294, 0.00325, 0.00374, 0.00438, 0.00512, 0.00588, 0.00663, 0.00729, 0.00783, 0.00823, 0.00846, 0.00854, 0.00849, 0.00835, 0.00815, 0.00797, 0.00784, 0.0078, 0.0079, 0.00814, 0.00855, 0.0091, 0.00979, 0.01057, 0.01141, 0.01227, 0.0131, 0.01386, 0.01452, 0.01505, 0.01543, 0.01566, 0.01573, 0.01564, 0.01541, 0.01506, 0.0146, 0.01408, 0.01351, 0.01295, 0.01242, 0.01196, 0.01161, 0.0114, 0.01134, 0.01144, 0.01171, 0.01211, 0.01263, 0.01321, 0.01379, 0.01434, 0.01479, 0.0151, 0.01523, 0.01517, 0.01489, 0.01442, 0.01376, 0.01294, 0.01199]}, 'scan_angles': {'A405': [0.0, 5.0, 10.0, 15.0, 20.0, 25.0, 30.0, 35.0, 40.0, 45.0, 50.0, 55.0, 60.0, 65.0, 70.0, 75.0, 80.0, 85.0, 90.0, 95.0, 100.0, 105.0, 110.0, 115.0, 120.0, 125.0, 130.0, 135.0, 140.0, 145.0, 150.0, 155.0, 160.0, 165.0, 170.0, 175.0, 180.0, 185.0, 190.0, 195.0, 200.0, 205.0, 210.0, 215.0, 220.0, 225.0, 230.0, 235.0, 240.0, 245.0, 250.0, 255.0, 260.0, 265.0, 270.0, 275.0, 280.0, 285.0, 290.0, 295.0, 300.0, 305.0, 310.0, 315.0, 320.0, 325.0, 330.0, 335.0, 340.0, 345.0, 350.0, 355.0], 'A407': [0.0, 5.0, 10.0, 15.0, 20.0, 25.0, 30.0, 35.0, 40.0, 45.0, 50.0, 55.0, 60.0, 65.0, 70.0, 75.0, 80.0, 85.0, 90.0, 95.0, 100.0, 105.0, 110.0, 115.0, 120.0, 125.0, 130.0, 135.0, 140.0, 145.0, 150.0, 155.0, 160.0, 165.0, 170.0, 175.0, 180.0, 185.0, 190.0, 195.0, 200.0, 205.0, 210.0, 215.0, 220.0, 225.0, 230.0, 235.0, 240.0, 245.0, 250.0, 255.0, 260.0, 265.0, 270.0, 275.0, 280.0, 285.0, 290.0, 295.0, 300.0, 305.0, 310.0, 315.0, 320.0, 325.0, 330.0, 335.0, 340.0, 345.0, 350.0, 355.0], 'A410': [0.0, 5.0, 10.0, 15.0, 20.0, 25.0, 30.0, 35.0, 40.0, 45.0, 50.0, 55.0, 60.0, 65.0, 70.0, 75.0, 80.0, 85.0, 90.0, 95.0, 100.0, 105.0, 110.0, 115.0, 120.0, 125.0, 130.0, 135.0, 140.0, 145.0, 150.0, 155.0, 160.0, 165.0, 170.0, 175.0, 180.0, 185.0, 190.0, 195.0, 200.0, 205.0, 210.0, 215.0, 220.0, 225.0, 230.0, 235.0, 240.0, 245.0, 250.0, 255.0, 260.0, 265.0, 270.0, 275.0, 280.0, 285.0, 290.0, 295.0, 300.0, 305.0, 310.0, 315.0, 320.0, 325.0, 330.0, 335.0, 340.0, 345.0, 350.0, 355.0], 'B603': [0.0, 5.0, 10.0, 15.0, 20.0, 25.0, 30.0, 35.0, 40.0, 45.0, 50.0, 55.0, 60.0, 65.0, 70.0, 75.0, 80.0, 85.0, 90.0, 95.0, 100.0, 105.0, 110.0, 115.0, 120.0, 125.0, 130.0, 135.0, 140.0, 145.0, 150.0, 155.0, 160.0, 165.0, 170.0, 175.0, 180.0, 185.0, 190.0, 195.0, 200.0, 205.0, 210.0, 215.0, 220.0, 225.0, 230.0, 235.0, 240.0, 245.0, 250.0, 255.0, 260.0, 265.0, 270.0, 275.0, 280.0, 285.0, 290.0, 295.0, 300.0, 305.0, 310.0, 315.0, 320.0, 325.0, 330.0, 335.0, 340.0, 345.0, 350.0, 355.0], 'B604': [0.0, 5.0, 10.0, 15.0, 20.0, 25.0, 30.0, 35.0, 40.0, 45.0, 50.0, 55.0, 60.0, 65.0, 70.0, 75.0, 80.0, 85.0, 90.0, 95.0, 100.0, 105.0, 110.0, 115.0, 120.0, 125.0, 130.0, 135.0, 140.0, 145.0, 150.0, 155.0, 160.0, 165.0, 170.0, 175.0, 180.0, 185.0, 190.0, 195.0, 200.0, 205.0, 210.0, 215.0, 220.0, 225.0, 230.0, 235.0, 240.0, 245.0, 250.0, 255.0, 260.0, 265.0, 270.0, 275.0, 280.0, 285.0, 290.0, 295.0, 300.0, 305.0, 310.0, 315.0, 320.0, 325.0, 330.0, 335.0, 340.0, 345.0, 350.0, 355.0], 'B605': [0.0, 5.0, 10.0, 15.0, 20.0, 25.0, 30.0, 35.0, 40.0, 45.0, 50.0, 55.0, 60.0, 65.0, 70.0, 75.0, 80.0, 85.0, 90.0, 95.0, 100.0, 105.0, 110.0, 115.0, 120.0, 125.0, 130.0, 135.0, 140.0, 145.0, 150.0, 155.0, 160.0, 165.0, 170.0, 175.0, 180.0, 185.0, 190.0, 195.0, 200.0, 205.0, 210.0, 215.0, 220.0, 225.0, 230.0, 235.0, 240.0, 245.0, 250.0, 255.0, 260.0, 265.0, 270.0, 275.0, 280.0, 285.0, 290.0, 295.0, 300.0, 305.0, 310.0, 315.0, 320.0, 325.0, 330.0, 335.0, 340.0, 345.0, 350.0, 355.0], 'B606': [0.0, 5.0, 10.0, 15.0, 20.0, 25.0, 30.0, 35.0, 40.0, 45.0, 50.0, 55.0, 60.0, 65.0, 70.0, 75.0, 80.0, 85.0, 90.0, 95.0, 100.0, 105.0, 110.0, 115.0, 120.0, 125.0, 130.0, 135.0, 140.0, 145.0, 150.0, 155.0, 160.0, 165.0, 170.0, 175.0, 180.0, 185.0, 190.0, 195.0, 200.0, 205.0, 210.0, 215.0, 220.0, 225.0, 230.0, 235.0, 240.0, 245.0, 250.0, 255.0, 260.0, 265.0, 270.0, 275.0, 280.0, 285.0, 290.0, 295.0, 300.0, 305.0, 310.0, 315.0, 320.0, 325.0, 330.0, 335.0, 340.0, 345.0, 350.0, 355.0], 'B607': [0.0, 5.0, 10.0, 15.0, 20.0, 25.0, 30.0, 35.0, 40.0, 45.0, 50.0, 55.0, 60.0, 65.0, 70.0, 75.0, 80.0, 85.0, 90.0, 95.0, 100.0, 105.0, 110.0, 115.0, 120.0, 125.0, 130.0, 135.0, 140.0, 145.0, 150.0, 155.0, 160.0, 165.0, 170.0, 175.0, 180.0, 185.0, 190.0, 195.0, 200.0, 205.0, 210.0, 215.0, 220.0, 225.0, 230.0, 235.0, 240.0, 245.0, 250.0, 255.0, 260.0, 265.0, 270.0, 275.0, 280.0, 285.0, 290.0, 295.0, 300.0, 305.0, 310.0, 315.0, 320.0, 325.0, 330.0, 335.0, 340.0, 345.0, 350.0, 355.0], 'B608': [0.0, 5.0, 10.0, 15.0, 20.0, 25.0, 30.0, 35.0, 40.0, 45.0, 50.0, 55.0, 60.0, 65.0, 70.0, 75.0, 80.0, 85.0, 90.0, 95.0, 100.0, 105.0, 110.0, 115.0, 120.0, 125.0, 130.0, 135.0, 140.0, 145.0, 150.0, 155.0, 160.0, 165.0, 170.0, 175.0, 180.0, 185.0, 190.0, 195.0, 200.0, 205.0, 210.0, 215.0, 220.0, 225.0, 230.0, 235.0, 240.0, 245.0, 250.0, 255.0, 260.0, 265.0, 270.0, 275.0, 280.0, 285.0, 290.0, 295.0, 300.0, 305.0, 310.0, 315.0, 320.0, 325.0, 330.0, 335.0, 340.0, 345.0, 350.0, 355.0], 'B609': [0.0, 5.0, 10.0, 15.0, 20.0, 25.0, 30.0, 35.0, 40.0, 45.0, 50.0, 55.0, 60.0, 65.0, 70.0, 75.0, 80.0, 85.0, 90.0, 95.0, 100.0, 105.0, 110.0, 115.0, 120.0, 125.0, 130.0, 135.0, 140.0, 145.0, 150.0, 155.0, 160.0, 165.0, 170.0, 175.0, 180.0, 185.0, 190.0, 195.0, 200.0, 205.0, 210.0, 215.0, 220.0, 225.0, 230.0, 235.0, 240.0, 245.0, 250.0, 255.0, 260.0, 265.0, 270.0, 275.0, 280.0, 285.0, 290.0, 295.0, 300.0, 305.0, 310.0, 315.0, 320.0, 325.0, 330.0, 335.0, 340.0, 345.0, 350.0, 355.0], 'B610': [0.0, 5.0, 10.0, 15.0, 20.0, 25.0, 30.0, 35.0, 40.0, 45.0, 50.0, 55.0, 60.0, 65.0, 70.0, 75.0, 80.0, 85.0, 90.0, 95.0, 100.0, 105.0, 110.0, 115.0, 120.0, 125.0, 130.0, 135.0, 140.0, 145.0, 150.0, 155.0, 160.0, 165.0, 170.0, 175.0, 180.0, 185.0, 190.0, 195.0, 200.0, 205.0, 210.0, 215.0, 220.0, 225.0, 230.0, 235.0, 240.0, 245.0, 250.0, 255.0, 260.0, 265.0, 270.0, 275.0, 280.0, 285.0, 290.0, 295.0, 300.0, 305.0, 310.0, 315.0, 320.0, 325.0, 330.0, 335.0, 340.0, 345.0, 350.0, 355.0], 'B611': [0.0, 5.0, 10.0, 15.0, 20.0, 25.0, 30.0, 35.0, 40.0, 45.0, 50.0, 55.0, 60.0, 65.0, 70.0, 75.0, 80.0, 85.0, 90.0, 95.0, 100.0, 105.0, 110.0, 115.0, 120.0, 125.0, 130.0, 135.0, 140.0, 145.0, 150.0, 155.0, 160.0, 165.0, 170.0, 175.0, 180.0, 185.0, 190.0, 195.0, 200.0, 205.0, 210.0, 215.0, 220.0, 225.0, 230.0, 235.0, 240.0, 245.0, 250.0, 255.0, 260.0, 265.0, 270.0, 275.0, 280.0, 285.0, 290.0, 295.0, 300.0, 305.0, 310.0, 315.0, 320.0, 325.0, 330.0, 335.0, 340.0, 345.0, 350.0, 355.0], 'B612': [0.0, 5.0, 10.0, 15.0, 20.0, 25.0, 30.0, 35.0, 40.0, 45.0, 50.0, 55.0, 60.0, 65.0, 70.0, 75.0, 80.0, 85.0, 90.0, 95.0, 100.0, 105.0, 110.0, 115.0, 120.0, 125.0, 130.0, 135.0, 140.0, 145.0, 150.0, 155.0, 160.0, 165.0, 170.0, 175.0, 180.0, 185.0, 190.0, 195.0, 200.0, 205.0, 210.0, 215.0, 220.0, 225.0, 230.0, 235.0, 240.0, 245.0, 250.0, 255.0, 260.0, 265.0, 270.0, 275.0, 280.0, 285.0, 290.0, 295.0, 300.0, 305.0, 310.0, 315.0, 320.0, 325.0, 330.0, 335.0, 340.0, 345.0, 350.0, 355.0], 'B613': [0.0, 5.0, 10.0, 15.0, 20.0, 25.0, 30.0, 35.0, 40.0, 45.0, 50.0, 55.0, 60.0, 65.0, 70.0, 75.0, 80.0, 85.0, 90.0, 95.0, 100.0, 105.0, 110.0, 115.0, 120.0, 125.0, 130.0, 135.0, 140.0, 145.0, 150.0, 155.0, 160.0, 165.0, 170.0, 175.0, 180.0, 185.0, 190.0, 195.0, 200.0, 205.0, 210.0, 215.0, 220.0, 225.0, 230.0, 235.0, 240.0, 245.0, 250.0, 255.0, 260.0, 265.0, 270.0, 275.0, 280.0, 285.0, 290.0, 295.0, 300.0, 305.0, 310.0, 315.0, 320.0, 325.0, 330.0, 335.0, 340.0, 345.0, 350.0, 355.0], 'B614': [0.0, 5.0, 10.0, 15.0, 20.0, 25.0, 30.0, 35.0, 40.0, 45.0, 50.0, 55.0, 60.0, 65.0, 70.0, 75.0, 80.0, 85.0, 90.0, 95.0, 100.0, 105.0, 110.0, 115.0, 120.0, 125.0, 130.0, 135.0, 140.0, 145.0, 150.0, 155.0, 160.0, 165.0, 170.0, 175.0, 180.0, 185.0, 190.0, 195.0, 200.0, 205.0, 210.0, 215.0, 220.0, 225.0, 230.0, 235.0, 240.0, 245.0, 250.0, 255.0, 260.0, 265.0, 270.0, 275.0, 280.0, 285.0, 290.0, 295.0, 300.0, 305.0, 310.0, 315.0, 320.0, 325.0, 330.0, 335.0, 340.0, 345.0, 350.0, 355.0], 'B616': [0.0, 5.0, 10.0, 15.0, 20.0, 25.0, 30.0, 35.0, 40.0, 45.0, 50.0, 55.0, 60.0, 65.0, 70.0, 75.0, 80.0, 85.0, 90.0, 95.0, 100.0, 105.0, 110.0, 115.0, 120.0, 125.0, 130.0, 135.0, 140.0, 145.0, 150.0, 155.0, 160.0, 165.0, 170.0, 175.0, 180.0, 185.0, 190.0, 195.0, 200.0, 205.0, 210.0, 215.0, 220.0, 225.0, 230.0, 235.0, 240.0, 245.0, 250.0, 255.0, 260.0, 265.0, 270.0, 275.0, 280.0, 285.0, 290.0, 295.0, 300.0, 305.0, 310.0, 315.0, 320.0, 325.0, 330.0, 335.0, 340.0, 345.0, 350.0, 355.0], 'B617': [0.0, 5.0, 10.0, 15.0, 20.0, 25.0, 30.0, 35.0, 40.0, 45.0, 50.0, 55.0, 60.0, 65.0, 70.0, 75.0, 80.0, 85.0, 90.0, 95.0, 100.0, 105.0, 110.0, 115.0, 120.0, 125.0, 130.0, 135.0, 140.0, 145.0, 150.0, 155.0, 160.0, 165.0, 170.0, 175.0, 180.0, 185.0, 190.0, 195.0, 200.0, 205.0, 210.0, 215.0, 220.0, 225.0, 230.0, 235.0, 240.0, 245.0, 250.0, 255.0, 260.0, 265.0, 270.0, 275.0, 280.0, 285.0, 290.0, 295.0, 300.0, 305.0, 310.0, 315.0, 320.0, 325.0, 330.0, 335.0, 340.0, 345.0, 350.0, 355.0], 'C501': [0.0, 5.0, 10.0, 15.0, 20.0, 25.0, 30.0, 35.0, 40.0, 45.0, 50.0, 55.0, 60.0, 65.0, 70.0, 75.0, 80.0, 85.0, 90.0, 95.0, 100.0, 105.0, 110.0, 115.0, 120.0, 125.0, 130.0, 135.0, 140.0, 145.0, 150.0, 155.0, 160.0, 165.0, 170.0, 175.0, 180.0, 185.0, 190.0, 195.0, 200.0, 205.0, 210.0, 215.0, 220.0, 225.0, 230.0, 235.0, 240.0, 245.0, 250.0, 255.0, 260.0, 265.0, 270.0, 275.0, 280.0, 285.0, 290.0, 295.0, 300.0, 305.0, 310.0, 315.0, 320.0, 325.0, 330.0, 335.0, 340.0, 345.0, 350.0, 355.0], 'C502': [0.0, 5.0, 10.0, 15.0, 20.0, 25.0, 30.0, 35.0, 40.0, 45.0, 50.0, 55.0, 60.0, 65.0, 70.0, 75.0, 80.0, 85.0, 90.0, 95.0, 100.0, 105.0, 110.0, 115.0, 120.0, 125.0, 130.0, 135.0, 140.0, 145.0, 150.0, 155.0, 160.0, 165.0, 170.0, 175.0, 180.0, 185.0, 190.0, 195.0, 200.0, 205.0, 210.0, 215.0, 220.0, 225.0, 230.0, 235.0, 240.0, 245.0, 250.0, 255.0, 260.0, 265.0, 270.0, 275.0, 280.0, 285.0, 290.0, 295.0, 300.0, 305.0, 310.0, 315.0, 320.0, 325.0, 330.0, 335.0, 340.0, 345.0, 350.0, 355.0], 'C503': [0.0, 5.0, 10.0, 15.0, 20.0, 25.0, 30.0, 35.0, 40.0, 45.0, 50.0, 55.0, 60.0, 65.0, 70.0, 75.0, 80.0, 85.0, 90.0, 95.0, 100.0, 105.0, 110.0, 115.0, 120.0, 125.0, 130.0, 135.0, 140.0, 145.0, 150.0, 155.0, 160.0, 165.0, 170.0, 175.0, 180.0, 185.0, 190.0, 195.0, 200.0, 205.0, 210.0, 215.0, 220.0, 225.0, 230.0, 235.0, 240.0, 245.0, 250.0, 255.0, 260.0, 265.0, 270.0, 275.0, 280.0, 285.0, 290.0, 295.0, 300.0, 305.0, 310.0, 315.0, 320.0, 325.0, 330.0, 335.0, 340.0, 345.0, 350.0, 355.0], 'C504': [0.0, 5.0, 10.0, 15.0, 20.0, 25.0, 30.0, 35.0, 40.0, 45.0, 50.0, 55.0, 60.0, 65.0, 70.0, 75.0, 80.0, 85.0, 90.0, 95.0, 100.0, 105.0, 110.0, 115.0, 120.0, 125.0, 130.0, 135.0, 140.0, 145.0, 150.0, 155.0, 160.0, 165.0, 170.0, 175.0, 180.0, 185.0, 190.0, 195.0, 200.0, 205.0, 210.0, 215.0, 220.0, 225.0, 230.0, 235.0, 240.0, 245.0, 250.0, 255.0, 260.0, 265.0, 270.0, 275.0, 280.0, 285.0, 290.0, 295.0, 300.0, 305.0, 310.0, 315.0, 320.0, 325.0, 330.0, 335.0, 340.0, 345.0, 350.0, 355.0], 'C505': [0.0, 5.0, 10.0, 15.0, 20.0, 25.0, 30.0, 35.0, 40.0, 45.0, 50.0, 55.0, 60.0, 65.0, 70.0, 75.0, 80.0, 85.0, 90.0, 95.0, 100.0, 105.0, 110.0, 115.0, 120.0, 125.0, 130.0, 135.0, 140.0, 145.0, 150.0, 155.0, 160.0, 165.0, 170.0, 175.0, 180.0, 185.0, 190.0, 195.0, 200.0, 205.0, 210.0, 215.0, 220.0, 225.0, 230.0, 235.0, 240.0, 245.0, 250.0, 255.0, 260.0, 265.0, 270.0, 275.0, 280.0, 285.0, 290.0, 295.0, 300.0, 305.0, 310.0, 315.0, 320.0, 325.0, 330.0, 335.0, 340.0, 345.0, 350.0, 355.0], 'C506': [0.0, 5.0, 10.0, 15.0, 20.0, 25.0, 30.0, 35.0, 40.0, 45.0, 50.0, 55.0, 60.0, 65.0, 70.0, 75.0, 80.0, 85.0, 90.0, 95.0, 100.0, 105.0, 110.0, 115.0, 120.0, 125.0, 130.0, 135.0, 140.0, 145.0, 150.0, 155.0, 160.0, 165.0, 170.0, 175.0, 180.0, 185.0, 190.0, 195.0, 200.0, 205.0, 210.0, 215.0, 220.0, 225.0, 230.0, 235.0, 240.0, 245.0, 250.0, 255.0, 260.0, 265.0, 270.0, 275.0, 280.0, 285.0, 290.0, 295.0, 300.0, 305.0, 310.0, 315.0, 320.0, 325.0, 330.0, 335.0, 340.0, 345.0, 350.0, 355.0], 'C507': [0.0, 5.0, 10.0, 15.0, 20.0, 25.0, 30.0, 35.0, 40.0, 45.0, 50.0, 55.0, 60.0, 65.0, 70.0, 75.0, 80.0, 85.0, 90.0, 95.0, 100.0, 105.0, 110.0, 115.0, 120.0, 125.0, 130.0, 135.0, 140.0, 145.0, 150.0, 155.0, 160.0, 165.0, 170.0, 175.0, 180.0, 185.0, 190.0, 195.0, 200.0, 205.0, 210.0, 215.0, 220.0, 225.0, 230.0, 235.0, 240.0, 245.0, 250.0, 255.0, 260.0, 265.0, 270.0, 275.0, 280.0, 285.0, 290.0, 295.0, 300.0, 305.0, 310.0, 315.0, 320.0, 325.0, 330.0, 335.0, 340.0, 345.0, 350.0, 355.0], 'C508': [0.0, 5.0, 10.0, 15.0, 20.0, 25.0, 30.0, 35.0, 40.0, 45.0, 50.0, 55.0, 60.0, 65.0, 70.0, 75.0, 80.0, 85.0, 90.0, 95.0, 100.0, 105.0, 110.0, 115.0, 120.0, 125.0, 130.0, 135.0, 140.0, 145.0, 150.0, 155.0, 160.0, 165.0, 170.0, 175.0, 180.0, 185.0, 190.0, 195.0, 200.0, 205.0, 210.0, 215.0, 220.0, 225.0, 230.0, 235.0, 240.0, 245.0, 250.0, 255.0, 260.0, 265.0, 270.0, 275.0, 280.0, 285.0, 290.0, 295.0, 300.0, 305.0, 310.0, 315.0, 320.0, 325.0, 330.0, 335.0, 340.0, 345.0, 350.0, 355.0], 'C509': [0.0, 5.0, 10.0, 15.0, 20.0, 25.0, 30.0, 35.0, 40.0, 45.0, 50.0, 55.0, 60.0, 65.0, 70.0, 75.0, 80.0, 85.0, 90.0, 95.0, 100.0, 105.0, 110.0, 115.0, 120.0, 125.0, 130.0, 135.0, 140.0, 145.0, 150.0, 155.0, 160.0, 165.0, 170.0, 175.0, 180.0, 185.0, 190.0, 195.0, 200.0, 205.0, 210.0, 215.0, 220.0, 225.0, 230.0, 235.0, 240.0, 245.0, 250.0, 255.0, 260.0, 265.0, 270.0, 275.0, 280.0, 285.0, 290.0, 295.0, 300.0, 305.0, 310.0, 315.0, 320.0, 325.0, 330.0, 335.0, 340.0, 345.0, 350.0, 355.0], 'C510': [0.0, 5.0, 10.0, 15.0, 20.0, 25.0, 30.0, 35.0, 40.0, 45.0, 50.0, 55.0, 60.0, 65.0, 70.0, 75.0, 80.0, 85.0, 90.0, 95.0, 100.0, 105.0, 110.0, 115.0, 120.0, 125.0, 130.0, 135.0, 140.0, 145.0, 150.0, 155.0, 160.0, 165.0, 170.0, 175.0, 180.0, 185.0, 190.0, 195.0, 200.0, 205.0, 210.0, 215.0, 220.0, 225.0, 230.0, 235.0, 240.0, 245.0, 250.0, 255.0, 260.0, 265.0, 270.0, 275.0, 280.0, 285.0, 290.0, 295.0, 300.0, 305.0, 310.0, 315.0, 320.0, 325.0, 330.0, 335.0, 340.0, 345.0, 350.0, 355.0], 'C511': [0.0, 5.0, 10.0, 15.0, 20.0, 25.0, 30.0, 35.0, 40.0, 45.0, 50.0, 55.0, 60.0, 65.0, 70.0, 75.0, 80.0, 85.0, 90.0, 95.0, 100.0, 105.0, 110.0, 115.0, 120.0, 125.0, 130.0, 135.0, 140.0, 145.0, 150.0, 155.0, 160.0, 165.0, 170.0, 175.0, 180.0, 185.0, 190.0, 195.0, 200.0, 205.0, 210.0, 215.0, 220.0, 225.0, 230.0, 235.0, 240.0, 245.0, 250.0, 255.0, 260.0, 265.0, 270.0, 275.0, 280.0, 285.0, 290.0, 295.0, 300.0, 305.0, 310.0, 315.0, 320.0, 325.0, 330.0, 335.0, 340.0, 345.0, 350.0, 355.0], 'C512': [0.0, 5.0, 10.0, 15.0, 20.0, 25.0, 30.0, 35.0, 40.0, 45.0, 50.0, 55.0, 60.0, 65.0, 70.0, 75.0, 80.0, 85.0, 90.0, 95.0, 100.0, 105.0, 110.0, 115.0, 120.0, 125.0, 130.0, 135.0, 140.0, 145.0, 150.0, 155.0, 160.0, 165.0, 170.0, 175.0, 180.0, 185.0, 190.0, 195.0, 200.0, 205.0, 210.0, 215.0, 220.0, 225.0, 230.0, 235.0, 240.0, 245.0, 250.0, 255.0, 260.0, 265.0, 270.0, 275.0, 280.0, 285.0, 290.0, 295.0, 300.0, 305.0, 310.0, 315.0, 320.0, 325.0, 330.0, 335.0, 340.0, 345.0, 350.0, 355.0], 'C513': [0.0, 5.0, 10.0, 15.0, 20.0, 25.0, 30.0, 35.0, 40.0, 45.0, 50.0, 55.0, 60.0, 65.0, 70.0, 75.0, 80.0, 85.0, 90.0, 95.0, 100.0, 105.0, 110.0, 115.0, 120.0, 125.0, 130.0, 135.0, 140.0, 145.0, 150.0, 155.0, 160.0, 165.0, 170.0, 175.0, 180.0, 185.0, 190.0, 195.0, 200.0, 205.0, 210.0, 215.0, 220.0, 225.0, 230.0, 235.0, 240.0, 245.0, 250.0, 255.0, 260.0, 265.0, 270.0, 275.0, 280.0, 285.0, 290.0, 295.0, 300.0, 305.0, 310.0, 315.0, 320.0, 325.0, 330.0, 335.0, 340.0, 345.0, 350.0, 355.0], 'D402': [0.0, 5.0, 10.0, 15.0, 20.0, 25.0, 30.0, 35.0, 40.0, 45.0, 50.0, 55.0, 60.0, 65.0, 70.0, 75.0, 80.0, 85.0, 90.0, 95.0, 100.0, 105.0, 110.0, 115.0, 120.0, 125.0, 130.0, 135.0, 140.0, 145.0, 150.0, 155.0, 160.0, 165.0, 170.0, 175.0, 180.0, 185.0, 190.0, 195.0, 200.0, 205.0, 210.0, 215.0, 220.0, 225.0, 230.0, 235.0, 240.0, 245.0, 250.0, 255.0, 260.0, 265.0, 270.0, 275.0, 280.0, 285.0, 290.0, 295.0, 300.0, 305.0, 310.0, 315.0, 320.0, 325.0, 330.0, 335.0, 340.0, 345.0, 350.0, 355.0], 'D403': [0.0, 5.0, 10.0, 15.0, 20.0, 25.0, 30.0, 35.0, 40.0, 45.0, 50.0, 55.0, 60.0, 65.0, 70.0, 75.0, 80.0, 85.0, 90.0, 95.0, 100.0, 105.0, 110.0, 115.0, 120.0, 125.0, 130.0, 135.0, 140.0, 145.0, 150.0, 155.0, 160.0, 165.0, 170.0, 175.0, 180.0, 185.0, 190.0, 195.0, 200.0, 205.0, 210.0, 215.0, 220.0, 225.0, 230.0, 235.0, 240.0, 245.0, 250.0, 255.0, 260.0, 265.0, 270.0, 275.0, 280.0, 285.0, 290.0, 295.0, 300.0, 305.0, 310.0, 315.0, 320.0, 325.0, 330.0, 335.0, 340.0, 345.0, 350.0, 355.0]}, 'ci': {'A405': [[-0.017095228781793037, 0.030134603781793036], [-0.017275271002723955, 0.030104021002723955], [-0.017301088723121957, 0.02996921372312196], [-0.017184445791507758, 0.02975694579150776], [-0.016972606362708413, 0.02950760636270841], [-0.016718333958153996, 0.029267083958153993], [-0.016453702731043345, 0.029063702731043348], [-0.01622278542622513, 0.02892653542622513], [-0.016039195797577604, 0.0288491957975776], [-0.015894771299909325, 0.028811646299909326], [-0.01577220959406654, 0.028777209594066544], [-0.01564259436468614, 0.02870009436468614], [-0.015494096729141833, 0.028542221729141833], [-0.015321928201526034, 0.028284428201526036], [-0.015141591474046014, 0.02792971647404601], [-0.014983124917233161, 0.02749312491723316], [-0.014893165186189192, 0.02701629018618919], [-0.014893308885125746, 0.026525183885125744], [-0.015005350269383131, 0.02604097526938313], [-0.015205165461357516, 0.025566415461357517], [-0.015477730464552282, 0.02509273046455228], [-0.015764327038068543, 0.024587452038068544], [-0.016003453462392347, 0.024025953462392342], [-0.0161680499732053, 0.023403674973205304], [-0.01621968566188263, 0.022723435661882625], [-0.016162490014402835, 0.022017490014402834], [-0.016023158030890963, 0.021341283030890962], [-0.015845786646416803, 0.0207589116464168], [-0.01569263328551246, 0.020348258285512455], [-0.015594981746192402, 0.020153106746192403], [-0.015595432431927446, 0.020205432431927448], [-0.015695162776436462, 0.020497037776436466], [-0.015877475589894337, 0.020992475589894338], [-0.016093826101258365, 0.021620701101258365], [-0.01631060911633407, 0.022312484116334068], [-0.016498137966657136, 0.023011262966657137], [-0.016627616598061314, 0.023655116598061313], [-0.016696522350849653, 0.024217772350849653], [-0.01671467100159018, 0.02467779600159018], [-0.016681901608578686, 0.025032526608578687], [-0.016614765518784203, 0.025276015518784205], [-0.01652684358098709, 0.025427468580987093], [-0.01642030501358744, 0.025491555013587443], [-0.0162960274498826, 0.025471027449882597], [-0.016144268531277102, 0.025376768531277107], [-0.015969679247991888, 0.025225304247991884], [-0.01575823179357756, 0.02503135679357756], [-0.015509353828164756, 0.024804353828164753], [-0.0152479232181379, 0.0245941732181379], [-0.014959212742292349, 0.02439858774229235], [-0.014675974088108168, 0.024268474088108165], [-0.014415899445869455, 0.024224024445869456], [-0.014179583689370914, 0.02428145868937091], [-0.01399063771621728, 0.024453762716217284], [-0.013836594108523368, 0.02473034410852337], [-0.013719088564294097, 0.025093463564294094], [-0.013625344990931543, 0.025517219990931546], [-0.013538554901586706, 0.025968554901586702], [-0.013455770047825114, 0.02642077004782511], [-0.013366275141652107, 0.026835650141652106], [-0.013288391555980564, 0.027209641555980567], [-0.01322850562563373, 0.027526630625633725], [-0.01321332237072971, 0.027797697370729714], [-0.013263388823922789, 0.028027138823922788], [-0.013418119014989315, 0.028254994014989314], [-0.013687281107159499, 0.0284885311071595], [-0.014081995398199633, 0.028754495398199638], [-0.014569917353290399, 0.0290361673532904], [-0.015141302752835232, 0.02934317775283523], [-0.015733081257448427, 0.02963620625744843], [-0.016290725114489785, 0.02988697511448979], [-0.016758548684960826, 0.03006229868496083]], 'A407': [[-0.017095228781793037, 0.030134603781793036], [-0.017275271002723955, 0.030104021002723955], [-0.017301088723121957, 0.02996921372312196], [-0.017184445791507758, 0.02975694579150776], [-0.016972606362708413, 0.02950760636270841], [-0.016718333958153996, 0.029267083958153993], [-0.016453702731043345, 0.029063702731043348], [-0.01622278542622513, 0.02892653542622513], [-0.016039195797577604, 0.0288491957975776], [-0.015894771299909325, 0.028811646299909326], [-0.01577220959406654, 0.028777209594066544], [-0.01564259436468614, 0.02870009436468614], [-0.015494096729141833, 0.028542221729141833], [-0.015321928201526034, 0.028284428201526036], [-0.015141591474046014, 0.02792971647404601], [-0.014983124917233161, 0.02749312491723316], [-0.014893165186189192, 0.02701629018618919], [-0.014893308885125746, 0.026525183885125744], [-0.015005350269383131, 0.02604097526938313], [-0.015205165461357516, 0.025566415461357517], [-0.015477730464552282, 0.02509273046455228], [-0.015764327038068543, 0.024587452038068544], [-0.016003453462392347, 0.024025953462392342], [-0.0161680499732053, 0.023403674973205304], [-0.01621968566188263, 0.022723435661882625], [-0.016162490014402835, 0.022017490014402834], [-0.016023158030890963, 0.021341283030890962], [-0.015845786646416803, 0.0207589116464168], [-0.01569263328551246, 0.020348258285512455], [-0.015594981746192402, 0.020153106746192403], [-0.015595432431927446, 0.020205432431927448], [-0.015695162776436462, 0.020497037776436466], [-0.015877475589894337, 0.020992475589894338], [-0.016093826101258365, 0.021620701101258365], [-0.01631060911633407, 0.022312484116334068], [-0.016498137966657136, 0.023011262966657137], [-0.016627616598061314, 0.023655116598061313], [-0.016696522350849653, 0.024217772350849653], [-0.01671467100159018, 0.02467779600159018], [-0.016681901608578686, 0.025032526608578687], [-0.016614765518784203, 0.025276015518784205], [-0.01652684358098709, 0.025427468580987093], [-0.01642030501358744, 0.025491555013587443], [-0.0162960274498826, 0.025471027449882597], [-0.016144268531277102, 0.025376768531277107], [-0.015969679247991888, 0.025225304247991884], [-0.01575823179357756, 0.02503135679357756], [-0.015509353828164756, 0.024804353828164753], [-0.0152479232181379, 0.0245941732181379], [-0.014959212742292349, 0.02439858774229235], [-0.014675974088108168, 0.024268474088108165], [-0.014415899445869455, 0.024224024445869456], [-0.014179583689370914, 0.02428145868937091], [-0.01399063771621728, 0.024453762716217284], [-0.013836594108523368, 0.02473034410852337], [-0.013719088564294097, 0.025093463564294094], [-0.013625344990931543, 0.025517219990931546], [-0.013538554901586706, 0.025968554901586702], [-0.013455770047825114, 0.02642077004782511], [-0.013366275141652107, 0.026835650141652106], [-0.013288391555980564, 0.027209641555980567], [-0.01322850562563373, 0.027526630625633725], [-0.01321332237072971, 0.027797697370729714], [-0.013263388823922789, 0.028027138823922788], [-0.013418119014989315, 0.028254994014989314], [-0.013687281107159499, 0.0284885311071595], [-0.014081995398199633, 0.028754495398199638], [-0.014569917353290399, 0.0290361673532904], [-0.015141302752835232, 0.02934317775283523], [-0.015733081257448427, 0.02963620625744843], [-0.016290725114489785, 0.02988697511448979], [-0.016758548684960826, 0.03006229868496083]], 'A410': [[-0.017095228781793037, 0.030134603781793036], [-0.017275271002723955, 0.030104021002723955], [-0.017301088723121957, 0.02996921372312196], [-0.017184445791507758, 0.02975694579150776], [-0.016972606362708413, 0.02950760636270841], [-0.016718333958153996, 0.029267083958153993], [-0.016453702731043345, 0.029063702731043348], [-0.01622278542622513, 0.02892653542622513], [-0.016039195797577604, 0.0288491957975776], [-0.015894771299909325, 0.028811646299909326], [-0.01577220959406654, 0.028777209594066544], [-0.01564259436468614, 0.02870009436468614], [-0.015494096729141833, 0.028542221729141833], [-0.015321928201526034, 0.028284428201526036], [-0.015141591474046014, 0.02792971647404601], [-0.014983124917233161, 0.02749312491723316], [-0.014893165186189192, 0.02701629018618919], [-0.014893308885125746, 0.026525183885125744], [-0.015005350269383131, 0.02604097526938313], [-0.015205165461357516, 0.025566415461357517], [-0.015477730464552282, 0.02509273046455228], [-0.015764327038068543, 0.024587452038068544], [-0.016003453462392347, 0.024025953462392342], [-0.0161680499732053, 0.023403674973205304], [-0.01621968566188263, 0.022723435661882625], [-0.016162490014402835, 0.022017490014402834], [-0.016023158030890963, 0.021341283030890962], [-0.015845786646416803, 0.0207589116464168], [-0.01569263328551246, 0.020348258285512455], [-0.015594981746192402, 0.020153106746192403], [-0.015595432431927446, 0.020205432431927448], [-0.015695162776436462, 0.020497037776436466], [-0.015877475589894337, 0.020992475589894338], [-0.016093826101258365, 0.021620701101258365], [-0.01631060911633407, 0.022312484116334068], [-0.016498137966657136, 0.023011262966657137], [-0.016627616598061314, 0.023655116598061313], [-0.016696522350849653, 0.024217772350849653], [-0.01671467100159018, 0.02467779600159018], [-0.016681901608578686, 0.025032526608578687], [-0.016614765518784203, 0.025276015518784205], [-0.01652684358098709, 0.025427468580987093], [-0.01642030501358744, 0.025491555013587443], [-0.0162960274498826, 0.025471027449882597], [-0.016144268531277102, 0.025376768531277107], [-0.015969679247991888, 0.025225304247991884], [-0.01575823179357756, 0.02503135679357756], [-0.015509353828164756, 0.024804353828164753], [-0.0152479232181379, 0.0245941732181379], [-0.014959212742292349, 0.02439858774229235], [-0.014675974088108168, 0.024268474088108165], [-0.014415899445869455, 0.024224024445869456], [-0.014179583689370914, 0.02428145868937091], [-0.01399063771621728, 0.024453762716217284], [-0.013836594108523368, 0.02473034410852337], [-0.013719088564294097, 0.025093463564294094], [-0.013625344990931543, 0.025517219990931546], [-0.013538554901586706, 0.025968554901586702], [-0.013455770047825114, 0.02642077004782511], [-0.013366275141652107, 0.026835650141652106], [-0.013288391555980564, 0.027209641555980567], [-0.01322850562563373, 0.027526630625633725], [-0.01321332237072971, 0.027797697370729714], [-0.013263388823922789, 0.028027138823922788], [-0.013418119014989315, 0.028254994014989314], [-0.013687281107159499, 0.0284885311071595], [-0.014081995398199633, 0.028754495398199638], [-0.014569917353290399, 0.0290361673532904], [-0.015141302752835232, 0.02934317775283523], [-0.015733081257448427, 0.02963620625744843], [-0.016290725114489785, 0.02988697511448979], [-0.016758548684960826, 0.03006229868496083]], 'B603': [[-0.017095228781793037, 0.030134603781793036], [-0.017275271002723955, 0.030104021002723955], [-0.017301088723121957, 0.02996921372312196], [-0.017184445791507758, 0.02975694579150776], [-0.016972606362708413, 0.02950760636270841], [-0.016718333958153996, 0.029267083958153993], [-0.016453702731043345, 0.029063702731043348], [-0.01622278542622513, 0.02892653542622513], [-0.016039195797577604, 0.0288491957975776], [-0.015894771299909325, 0.028811646299909326], [-0.01577220959406654, 0.028777209594066544], [-0.01564259436468614, 0.02870009436468614], [-0.015494096729141833, 0.028542221729141833], [-0.015321928201526034, 0.028284428201526036], [-0.015141591474046014, 0.02792971647404601], [-0.014983124917233161, 0.02749312491723316], [-0.014893165186189192, 0.02701629018618919], [-0.014893308885125746, 0.026525183885125744], [-0.015005350269383131, 0.02604097526938313], [-0.015205165461357516, 0.025566415461357517], [-0.015477730464552282, 0.02509273046455228], [-0.015764327038068543, 0.024587452038068544], [-0.016003453462392347, 0.024025953462392342], [-0.0161680499732053, 0.023403674973205304], [-0.01621968566188263, 0.022723435661882625], [-0.016162490014402835, 0.022017490014402834], [-0.016023158030890963, 0.021341283030890962], [-0.015845786646416803, 0.0207589116464168], [-0.01569263328551246, 0.020348258285512455], [-0.015594981746192402, 0.020153106746192403], [-0.015595432431927446, 0.020205432431927448], [-0.015695162776436462, 0.020497037776436466], [-0.015877475589894337, 0.020992475589894338], [-0.016093826101258365, 0.021620701101258365], [-0.01631060911633407, 0.022312484116334068], [-0.016498137966657136, 0.023011262966657137], [-0.016627616598061314, 0.023655116598061313], [-0.016696522350849653, 0.024217772350849653], [-0.01671467100159018, 0.02467779600159018], [-0.016681901608578686, 0.025032526608578687], [-0.016614765518784203, 0.025276015518784205], [-0.01652684358098709, 0.025427468580987093], [-0.01642030501358744, 0.025491555013587443], [-0.0162960274498826, 0.025471027449882597], [-0.016144268531277102, 0.025376768531277107], [-0.015969679247991888, 0.025225304247991884], [-0.01575823179357756, 0.02503135679357756], [-0.015509353828164756, 0.024804353828164753], [-0.0152479232181379, 0.0245941732181379], [-0.014959212742292349, 0.02439858774229235], [-0.014675974088108168, 0.024268474088108165], [-0.014415899445869455, 0.024224024445869456], [-0.014179583689370914, 0.02428145868937091], [-0.01399063771621728, 0.024453762716217284], [-0.013836594108523368, 0.02473034410852337], [-0.013719088564294097, 0.025093463564294094], [-0.013625344990931543, 0.025517219990931546], [-0.013538554901586706, 0.025968554901586702], [-0.013455770047825114, 0.02642077004782511], [-0.013366275141652107, 0.026835650141652106], [-0.013288391555980564, 0.027209641555980567], [-0.01322850562563373, 0.027526630625633725], [-0.01321332237072971, 0.027797697370729714], [-0.013263388823922789, 0.028027138823922788], [-0.013418119014989315, 0.028254994014989314], [-0.013687281107159499, 0.0284885311071595], [-0.014081995398199633, 0.028754495398199638], [-0.014569917353290399, 0.0290361673532904], [-0.015141302752835232, 0.02934317775283523], [-0.015733081257448427, 0.02963620625744843], [-0.016290725114489785, 0.02988697511448979], [-0.016758548684960826, 0.03006229868496083]], 'B604': [[-0.017095228781793037, 0.030134603781793036], [-0.017275271002723955, 0.030104021002723955], [-0.017301088723121957, 0.02996921372312196], [-0.017184445791507758, 0.02975694579150776], [-0.016972606362708413, 0.02950760636270841], [-0.016718333958153996, 0.029267083958153993], [-0.016453702731043345, 0.029063702731043348], [-0.01622278542622513, 0.02892653542622513], [-0.016039195797577604, 0.0288491957975776], [-0.015894771299909325, 0.028811646299909326], [-0.01577220959406654, 0.028777209594066544], [-0.01564259436468614, 0.02870009436468614], [-0.015494096729141833, 0.028542221729141833], [-0.015321928201526034, 0.028284428201526036], [-0.015141591474046014, 0.02792971647404601], [-0.014983124917233161, 0.02749312491723316], [-0.014893165186189192, 0.02701629018618919], [-0.014893308885125746, 0.026525183885125744], [-0.015005350269383131, 0.02604097526938313], [-0.015205165461357516, 0.025566415461357517], [-0.015477730464552282, 0.02509273046455228], [-0.015764327038068543, 0.024587452038068544], [-0.016003453462392347, 0.024025953462392342], [-0.0161680499732053, 0.023403674973205304], [-0.01621968566188263, 0.022723435661882625], [-0.016162490014402835, 0.022017490014402834], [-0.016023158030890963, 0.021341283030890962], [-0.015845786646416803, 0.0207589116464168], [-0.01569263328551246, 0.020348258285512455], [-0.015594981746192402, 0.020153106746192403], [-0.015595432431927446, 0.020205432431927448], [-0.015695162776436462, 0.020497037776436466], [-0.015877475589894337, 0.020992475589894338], [-0.016093826101258365, 0.021620701101258365], [-0.01631060911633407, 0.022312484116334068], [-0.016498137966657136, 0.023011262966657137], [-0.016627616598061314, 0.023655116598061313], [-0.016696522350849653, 0.024217772350849653], [-0.01671467100159018, 0.02467779600159018], [-0.016681901608578686, 0.025032526608578687], [-0.016614765518784203, 0.025276015518784205], [-0.01652684358098709, 0.025427468580987093], [-0.01642030501358744, 0.025491555013587443], [-0.0162960274498826, 0.025471027449882597], [-0.016144268531277102, 0.025376768531277107], [-0.015969679247991888, 0.025225304247991884], [-0.01575823179357756, 0.02503135679357756], [-0.015509353828164756, 0.024804353828164753], [-0.0152479232181379, 0.0245941732181379], [-0.014959212742292349, 0.02439858774229235], [-0.014675974088108168, 0.024268474088108165], [-0.014415899445869455, 0.024224024445869456], [-0.014179583689370914, 0.02428145868937091], [-0.01399063771621728, 0.024453762716217284], [-0.013836594108523368, 0.02473034410852337], [-0.013719088564294097, 0.025093463564294094], [-0.013625344990931543, 0.025517219990931546], [-0.013538554901586706, 0.025968554901586702], [-0.013455770047825114, 0.02642077004782511], [-0.013366275141652107, 0.026835650141652106], [-0.013288391555980564, 0.027209641555980567], [-0.01322850562563373, 0.027526630625633725], [-0.01321332237072971, 0.027797697370729714], [-0.013263388823922789, 0.028027138823922788], [-0.013418119014989315, 0.028254994014989314], [-0.013687281107159499, 0.0284885311071595], [-0.014081995398199633, 0.028754495398199638], [-0.014569917353290399, 0.0290361673532904], [-0.015141302752835232, 0.02934317775283523], [-0.015733081257448427, 0.02963620625744843], [-0.016290725114489785, 0.02988697511448979], [-0.016758548684960826, 0.03006229868496083]], 'B605': [[-0.017095228781793037, 0.030134603781793036], [-0.017275271002723955, 0.030104021002723955], [-0.017301088723121957, 0.02996921372312196], [-0.017184445791507758, 0.02975694579150776], [-0.016972606362708413, 0.02950760636270841], [-0.016718333958153996, 0.029267083958153993], [-0.016453702731043345, 0.029063702731043348], [-0.01622278542622513, 0.02892653542622513], [-0.016039195797577604, 0.0288491957975776], [-0.015894771299909325, 0.028811646299909326], [-0.01577220959406654, 0.028777209594066544], [-0.01564259436468614, 0.02870009436468614], [-0.015494096729141833, 0.028542221729141833], [-0.015321928201526034, 0.028284428201526036], [-0.015141591474046014, 0.02792971647404601], [-0.014983124917233161, 0.02749312491723316], [-0.014893165186189192, 0.02701629018618919], [-0.014893308885125746, 0.026525183885125744], [-0.015005350269383131, 0.02604097526938313], [-0.015205165461357516, 0.025566415461357517], [-0.015477730464552282, 0.02509273046455228], [-0.015764327038068543, 0.024587452038068544], [-0.016003453462392347, 0.024025953462392342], [-0.0161680499732053, 0.023403674973205304], [-0.01621968566188263, 0.022723435661882625], [-0.016162490014402835, 0.022017490014402834], [-0.016023158030890963, 0.021341283030890962], [-0.015845786646416803, 0.0207589116464168], [-0.01569263328551246, 0.020348258285512455], [-0.015594981746192402, 0.020153106746192403], [-0.015595432431927446, 0.020205432431927448], [-0.015695162776436462, 0.020497037776436466], [-0.015877475589894337, 0.020992475589894338], [-0.016093826101258365, 0.021620701101258365], [-0.01631060911633407, 0.022312484116334068], [-0.016498137966657136, 0.023011262966657137], [-0.016627616598061314, 0.023655116598061313], [-0.016696522350849653, 0.024217772350849653], [-0.01671467100159018, 0.02467779600159018], [-0.016681901608578686, 0.025032526608578687], [-0.016614765518784203, 0.025276015518784205], [-0.01652684358098709, 0.025427468580987093], [-0.01642030501358744, 0.025491555013587443], [-0.0162960274498826, 0.025471027449882597], [-0.016144268531277102, 0.025376768531277107], [-0.015969679247991888, 0.025225304247991884], [-0.01575823179357756, 0.02503135679357756], [-0.015509353828164756, 0.024804353828164753], [-0.0152479232181379, 0.0245941732181379], [-0.014959212742292349, 0.02439858774229235], [-0.014675974088108168, 0.024268474088108165], [-0.014415899445869455, 0.024224024445869456], [-0.014179583689370914, 0.02428145868937091], [-0.01399063771621728, 0.024453762716217284], [-0.013836594108523368, 0.02473034410852337], [-0.013719088564294097, 0.025093463564294094], [-0.013625344990931543, 0.025517219990931546], [-0.013538554901586706, 0.025968554901586702], [-0.013455770047825114, 0.02642077004782511], [-0.013366275141652107, 0.026835650141652106], [-0.013288391555980564, 0.027209641555980567], [-0.01322850562563373, 0.027526630625633725], [-0.01321332237072971, 0.027797697370729714], [-0.013263388823922789, 0.028027138823922788], [-0.013418119014989315, 0.028254994014989314], [-0.013687281107159499, 0.0284885311071595], [-0.014081995398199633, 0.028754495398199638], [-0.014569917353290399, 0.0290361673532904], [-0.015141302752835232, 0.02934317775283523], [-0.015733081257448427, 0.02963620625744843], [-0.016290725114489785, 0.02988697511448979], [-0.016758548684960826, 0.03006229868496083]], 'B606': [[-0.017095228781793037, 0.030134603781793036], [-0.017275271002723955, 0.030104021002723955], [-0.017301088723121957, 0.02996921372312196], [-0.017184445791507758, 0.02975694579150776], [-0.016972606362708413, 0.02950760636270841], [-0.016718333958153996, 0.029267083958153993], [-0.016453702731043345, 0.029063702731043348], [-0.01622278542622513, 0.02892653542622513], [-0.016039195797577604, 0.0288491957975776], [-0.015894771299909325, 0.028811646299909326], [-0.01577220959406654, 0.028777209594066544], [-0.01564259436468614, 0.02870009436468614], [-0.015494096729141833, 0.028542221729141833], [-0.015321928201526034, 0.028284428201526036], [-0.015141591474046014, 0.02792971647404601], [-0.014983124917233161, 0.02749312491723316], [-0.014893165186189192, 0.02701629018618919], [-0.014893308885125746, 0.026525183885125744], [-0.015005350269383131, 0.02604097526938313], [-0.015205165461357516, 0.025566415461357517], [-0.015477730464552282, 0.02509273046455228], [-0.015764327038068543, 0.024587452038068544], [-0.016003453462392347, 0.024025953462392342], [-0.0161680499732053, 0.023403674973205304], [-0.01621968566188263, 0.022723435661882625], [-0.016162490014402835, 0.022017490014402834], [-0.016023158030890963, 0.021341283030890962], [-0.015845786646416803, 0.0207589116464168], [-0.01569263328551246, 0.020348258285512455], [-0.015594981746192402, 0.020153106746192403], [-0.015595432431927446, 0.020205432431927448], [-0.015695162776436462, 0.020497037776436466], [-0.015877475589894337, 0.020992475589894338], [-0.016093826101258365, 0.021620701101258365], [-0.01631060911633407, 0.022312484116334068], [-0.016498137966657136, 0.023011262966657137], [-0.016627616598061314, 0.023655116598061313], [-0.016696522350849653, 0.024217772350849653], [-0.01671467100159018, 0.02467779600159018], [-0.016681901608578686, 0.025032526608578687], [-0.016614765518784203, 0.025276015518784205], [-0.01652684358098709, 0.025427468580987093], [-0.01642030501358744, 0.025491555013587443], [-0.0162960274498826, 0.025471027449882597], [-0.016144268531277102, 0.025376768531277107], [-0.015969679247991888, 0.025225304247991884], [-0.01575823179357756, 0.02503135679357756], [-0.015509353828164756, 0.024804353828164753], [-0.0152479232181379, 0.0245941732181379], [-0.014959212742292349, 0.02439858774229235], [-0.014675974088108168, 0.024268474088108165], [-0.014415899445869455, 0.024224024445869456], [-0.014179583689370914, 0.02428145868937091], [-0.01399063771621728, 0.024453762716217284], [-0.013836594108523368, 0.02473034410852337], [-0.013719088564294097, 0.025093463564294094], [-0.013625344990931543, 0.025517219990931546], [-0.013538554901586706, 0.025968554901586702], [-0.013455770047825114, 0.02642077004782511], [-0.013366275141652107, 0.026835650141652106], [-0.013288391555980564, 0.027209641555980567], [-0.01322850562563373, 0.027526630625633725], [-0.01321332237072971, 0.027797697370729714], [-0.013263388823922789, 0.028027138823922788], [-0.013418119014989315, 0.028254994014989314], [-0.013687281107159499, 0.0284885311071595], [-0.014081995398199633, 0.028754495398199638], [-0.014569917353290399, 0.0290361673532904], [-0.015141302752835232, 0.02934317775283523], [-0.015733081257448427, 0.02963620625744843], [-0.016290725114489785, 0.02988697511448979], [-0.016758548684960826, 0.03006229868496083]], 'B607': [[-0.017095228781793037, 0.030134603781793036], [-0.017275271002723955, 0.030104021002723955], [-0.017301088723121957, 0.02996921372312196], [-0.017184445791507758, 0.02975694579150776], [-0.016972606362708413, 0.02950760636270841], [-0.016718333958153996, 0.029267083958153993], [-0.016453702731043345, 0.029063702731043348], [-0.01622278542622513, 0.02892653542622513], [-0.016039195797577604, 0.0288491957975776], [-0.015894771299909325, 0.028811646299909326], [-0.01577220959406654, 0.028777209594066544], [-0.01564259436468614, 0.02870009436468614], [-0.015494096729141833, 0.028542221729141833], [-0.015321928201526034, 0.028284428201526036], [-0.015141591474046014, 0.02792971647404601], [-0.014983124917233161, 0.02749312491723316], [-0.014893165186189192, 0.02701629018618919], [-0.014893308885125746, 0.026525183885125744], [-0.015005350269383131, 0.02604097526938313], [-0.015205165461357516, 0.025566415461357517], [-0.015477730464552282, 0.02509273046455228], [-0.015764327038068543, 0.024587452038068544], [-0.016003453462392347, 0.024025953462392342], [-0.0161680499732053, 0.023403674973205304], [-0.01621968566188263, 0.022723435661882625], [-0.016162490014402835, 0.022017490014402834], [-0.016023158030890963, 0.021341283030890962], [-0.015845786646416803, 0.0207589116464168], [-0.01569263328551246, 0.020348258285512455], [-0.015594981746192402, 0.020153106746192403], [-0.015595432431927446, 0.020205432431927448], [-0.015695162776436462, 0.020497037776436466], [-0.015877475589894337, 0.020992475589894338], [-0.016093826101258365, 0.021620701101258365], [-0.01631060911633407, 0.022312484116334068], [-0.016498137966657136, 0.023011262966657137], [-0.016627616598061314, 0.023655116598061313], [-0.016696522350849653, 0.024217772350849653], [-0.01671467100159018, 0.02467779600159018], [-0.016681901608578686, 0.025032526608578687], [-0.016614765518784203, 0.025276015518784205], [-0.01652684358098709, 0.025427468580987093], [-0.01642030501358744, 0.025491555013587443], [-0.0162960274498826, 0.025471027449882597], [-0.016144268531277102, 0.025376768531277107], [-0.015969679247991888, 0.025225304247991884], [-0.01575823179357756, 0.02503135679357756], [-0.015509353828164756, 0.024804353828164753], [-0.0152479232181379, 0.0245941732181379], [-0.014959212742292349, 0.02439858774229235], [-0.014675974088108168, 0.024268474088108165], [-0.014415899445869455, 0.024224024445869456], [-0.014179583689370914, 0.02428145868937091], [-0.01399063771621728, 0.024453762716217284], [-0.013836594108523368, 0.02473034410852337], [-0.013719088564294097, 0.025093463564294094], [-0.013625344990931543, 0.025517219990931546], [-0.013538554901586706, 0.025968554901586702], [-0.013455770047825114, 0.02642077004782511], [-0.013366275141652107, 0.026835650141652106], [-0.013288391555980564, 0.027209641555980567], [-0.01322850562563373, 0.027526630625633725], [-0.01321332237072971, 0.027797697370729714], [-0.013263388823922789, 0.028027138823922788], [-0.013418119014989315, 0.028254994014989314], [-0.013687281107159499, 0.0284885311071595], [-0.014081995398199633, 0.028754495398199638], [-0.014569917353290399, 0.0290361673532904], [-0.015141302752835232, 0.02934317775283523], [-0.015733081257448427, 0.02963620625744843], [-0.016290725114489785, 0.02988697511448979], [-0.016758548684960826, 0.03006229868496083]], 'B608': [[-0.017095228781793037, 0.030134603781793036], [-0.017275271002723955, 0.030104021002723955], [-0.017301088723121957, 0.02996921372312196], [-0.017184445791507758, 0.02975694579150776], [-0.016972606362708413, 0.02950760636270841], [-0.016718333958153996, 0.029267083958153993], [-0.016453702731043345, 0.029063702731043348], [-0.01622278542622513, 0.02892653542622513], [-0.016039195797577604, 0.0288491957975776], [-0.015894771299909325, 0.028811646299909326], [-0.01577220959406654, 0.028777209594066544], [-0.01564259436468614, 0.02870009436468614], [-0.015494096729141833, 0.028542221729141833], [-0.015321928201526034, 0.028284428201526036], [-0.015141591474046014, 0.02792971647404601], [-0.014983124917233161, 0.02749312491723316], [-0.014893165186189192, 0.02701629018618919], [-0.014893308885125746, 0.026525183885125744], [-0.015005350269383131, 0.02604097526938313], [-0.015205165461357516, 0.025566415461357517], [-0.015477730464552282, 0.02509273046455228], [-0.015764327038068543, 0.024587452038068544], [-0.016003453462392347, 0.024025953462392342], [-0.0161680499732053, 0.023403674973205304], [-0.01621968566188263, 0.022723435661882625], [-0.016162490014402835, 0.022017490014402834], [-0.016023158030890963, 0.021341283030890962], [-0.015845786646416803, 0.0207589116464168], [-0.01569263328551246, 0.020348258285512455], [-0.015594981746192402, 0.020153106746192403], [-0.015595432431927446, 0.020205432431927448], [-0.015695162776436462, 0.020497037776436466], [-0.015877475589894337, 0.020992475589894338], [-0.016093826101258365, 0.021620701101258365], [-0.01631060911633407, 0.022312484116334068], [-0.016498137966657136, 0.023011262966657137], [-0.016627616598061314, 0.023655116598061313], [-0.016696522350849653, 0.024217772350849653], [-0.01671467100159018, 0.02467779600159018], [-0.016681901608578686, 0.025032526608578687], [-0.016614765518784203, 0.025276015518784205], [-0.01652684358098709, 0.025427468580987093], [-0.01642030501358744, 0.025491555013587443], [-0.0162960274498826, 0.025471027449882597], [-0.016144268531277102, 0.025376768531277107], [-0.015969679247991888, 0.025225304247991884], [-0.01575823179357756, 0.02503135679357756], [-0.015509353828164756, 0.024804353828164753], [-0.0152479232181379, 0.0245941732181379], [-0.014959212742292349, 0.02439858774229235], [-0.014675974088108168, 0.024268474088108165], [-0.014415899445869455, 0.024224024445869456], [-0.014179583689370914, 0.02428145868937091], [-0.01399063771621728, 0.024453762716217284], [-0.013836594108523368, 0.02473034410852337], [-0.013719088564294097, 0.025093463564294094], [-0.013625344990931543, 0.025517219990931546], [-0.013538554901586706, 0.025968554901586702], [-0.013455770047825114, 0.02642077004782511], [-0.013366275141652107, 0.026835650141652106], [-0.013288391555980564, 0.027209641555980567], [-0.01322850562563373, 0.027526630625633725], [-0.01321332237072971, 0.027797697370729714], [-0.013263388823922789, 0.028027138823922788], [-0.013418119014989315, 0.028254994014989314], [-0.013687281107159499, 0.0284885311071595], [-0.014081995398199633, 0.028754495398199638], [-0.014569917353290399, 0.0290361673532904], [-0.015141302752835232, 0.02934317775283523], [-0.015733081257448427, 0.02963620625744843], [-0.016290725114489785, 0.02988697511448979], [-0.016758548684960826, 0.03006229868496083]], 'B609': [[-0.017095228781793037, 0.030134603781793036], [-0.017275271002723955, 0.030104021002723955], [-0.017301088723121957, 0.02996921372312196], [-0.017184445791507758, 0.02975694579150776], [-0.016972606362708413, 0.02950760636270841], [-0.016718333958153996, 0.029267083958153993], [-0.016453702731043345, 0.029063702731043348], [-0.01622278542622513, 0.02892653542622513], [-0.016039195797577604, 0.0288491957975776], [-0.015894771299909325, 0.028811646299909326], [-0.01577220959406654, 0.028777209594066544], [-0.01564259436468614, 0.02870009436468614], [-0.015494096729141833, 0.028542221729141833], [-0.015321928201526034, 0.028284428201526036], [-0.015141591474046014, 0.02792971647404601], [-0.014983124917233161, 0.02749312491723316], [-0.014893165186189192, 0.02701629018618919], [-0.014893308885125746, 0.026525183885125744], [-0.015005350269383131, 0.02604097526938313], [-0.015205165461357516, 0.025566415461357517], [-0.015477730464552282, 0.02509273046455228], [-0.015764327038068543, 0.024587452038068544], [-0.016003453462392347, 0.024025953462392342], [-0.0161680499732053, 0.023403674973205304], [-0.01621968566188263, 0.022723435661882625], [-0.016162490014402835, 0.022017490014402834], [-0.016023158030890963, 0.021341283030890962], [-0.015845786646416803, 0.0207589116464168], [-0.01569263328551246, 0.020348258285512455], [-0.015594981746192402, 0.020153106746192403], [-0.015595432431927446, 0.020205432431927448], [-0.015695162776436462, 0.020497037776436466], [-0.015877475589894337, 0.020992475589894338], [-0.016093826101258365, 0.021620701101258365], [-0.01631060911633407, 0.022312484116334068], [-0.016498137966657136, 0.023011262966657137], [-0.016627616598061314, 0.023655116598061313], [-0.016696522350849653, 0.024217772350849653], [-0.01671467100159018, 0.02467779600159018], [-0.016681901608578686, 0.025032526608578687], [-0.016614765518784203, 0.025276015518784205], [-0.01652684358098709, 0.025427468580987093], [-0.01642030501358744, 0.025491555013587443], [-0.0162960274498826, 0.025471027449882597], [-0.016144268531277102, 0.025376768531277107], [-0.015969679247991888, 0.025225304247991884], [-0.01575823179357756, 0.02503135679357756], [-0.015509353828164756, 0.024804353828164753], [-0.0152479232181379, 0.0245941732181379], [-0.014959212742292349, 0.02439858774229235], [-0.014675974088108168, 0.024268474088108165], [-0.014415899445869455, 0.024224024445869456], [-0.014179583689370914, 0.02428145868937091], [-0.01399063771621728, 0.024453762716217284], [-0.013836594108523368, 0.02473034410852337], [-0.013719088564294097, 0.025093463564294094], [-0.013625344990931543, 0.025517219990931546], [-0.013538554901586706, 0.025968554901586702], [-0.013455770047825114, 0.02642077004782511], [-0.013366275141652107, 0.026835650141652106], [-0.013288391555980564, 0.027209641555980567], [-0.01322850562563373, 0.027526630625633725], [-0.01321332237072971, 0.027797697370729714], [-0.013263388823922789, 0.028027138823922788], [-0.013418119014989315, 0.028254994014989314], [-0.013687281107159499, 0.0284885311071595], [-0.014081995398199633, 0.028754495398199638], [-0.014569917353290399, 0.0290361673532904], [-0.015141302752835232, 0.02934317775283523], [-0.015733081257448427, 0.02963620625744843], [-0.016290725114489785, 0.02988697511448979], [-0.016758548684960826, 0.03006229868496083]], 'B610': [[-0.017095228781793037, 0.030134603781793036], [-0.017275271002723955, 0.030104021002723955], [-0.017301088723121957, 0.02996921372312196], [-0.017184445791507758, 0.02975694579150776], [-0.016972606362708413, 0.02950760636270841], [-0.016718333958153996, 0.029267083958153993], [-0.016453702731043345, 0.029063702731043348], [-0.01622278542622513, 0.02892653542622513], [-0.016039195797577604, 0.0288491957975776], [-0.015894771299909325, 0.028811646299909326], [-0.01577220959406654, 0.028777209594066544], [-0.01564259436468614, 0.02870009436468614], [-0.015494096729141833, 0.028542221729141833], [-0.015321928201526034, 0.028284428201526036], [-0.015141591474046014, 0.02792971647404601], [-0.014983124917233161, 0.02749312491723316], [-0.014893165186189192, 0.02701629018618919], [-0.014893308885125746, 0.026525183885125744], [-0.015005350269383131, 0.02604097526938313], [-0.015205165461357516, 0.025566415461357517], [-0.015477730464552282, 0.02509273046455228], [-0.015764327038068543, 0.024587452038068544], [-0.016003453462392347, 0.024025953462392342], [-0.0161680499732053, 0.023403674973205304], [-0.01621968566188263, 0.022723435661882625], [-0.016162490014402835, 0.022017490014402834], [-0.016023158030890963, 0.021341283030890962], [-0.015845786646416803, 0.0207589116464168], [-0.01569263328551246, 0.020348258285512455], [-0.015594981746192402, 0.020153106746192403], [-0.015595432431927446, 0.020205432431927448], [-0.015695162776436462, 0.020497037776436466], [-0.015877475589894337, 0.020992475589894338], [-0.016093826101258365, 0.021620701101258365], [-0.01631060911633407, 0.022312484116334068], [-0.016498137966657136, 0.023011262966657137], [-0.016627616598061314, 0.023655116598061313], [-0.016696522350849653, 0.024217772350849653], [-0.01671467100159018, 0.02467779600159018], [-0.016681901608578686, 0.025032526608578687], [-0.016614765518784203, 0.025276015518784205], [-0.01652684358098709, 0.025427468580987093], [-0.01642030501358744, 0.025491555013587443], [-0.0162960274498826, 0.025471027449882597], [-0.016144268531277102, 0.025376768531277107], [-0.015969679247991888, 0.025225304247991884], [-0.01575823179357756, 0.02503135679357756], [-0.015509353828164756, 0.024804353828164753], [-0.0152479232181379, 0.0245941732181379], [-0.014959212742292349, 0.02439858774229235], [-0.014675974088108168, 0.024268474088108165], [-0.014415899445869455, 0.024224024445869456], [-0.014179583689370914, 0.02428145868937091], [-0.01399063771621728, 0.024453762716217284], [-0.013836594108523368, 0.02473034410852337], [-0.013719088564294097, 0.025093463564294094], [-0.013625344990931543, 0.025517219990931546], [-0.013538554901586706, 0.025968554901586702], [-0.013455770047825114, 0.02642077004782511], [-0.013366275141652107, 0.026835650141652106], [-0.013288391555980564, 0.027209641555980567], [-0.01322850562563373, 0.027526630625633725], [-0.01321332237072971, 0.027797697370729714], [-0.013263388823922789, 0.028027138823922788], [-0.013418119014989315, 0.028254994014989314], [-0.013687281107159499, 0.0284885311071595], [-0.014081995398199633, 0.028754495398199638], [-0.014569917353290399, 0.0290361673532904], [-0.015141302752835232, 0.02934317775283523], [-0.015733081257448427, 0.02963620625744843], [-0.016290725114489785, 0.02988697511448979], [-0.016758548684960826, 0.03006229868496083]], 'B611': [[-0.017095228781793037, 0.030134603781793036], [-0.017275271002723955, 0.030104021002723955], [-0.017301088723121957, 0.02996921372312196], [-0.017184445791507758, 0.02975694579150776], [-0.016972606362708413, 0.02950760636270841], [-0.016718333958153996, 0.029267083958153993], [-0.016453702731043345, 0.029063702731043348], [-0.01622278542622513, 0.02892653542622513], [-0.016039195797577604, 0.0288491957975776], [-0.015894771299909325, 0.028811646299909326], [-0.01577220959406654, 0.028777209594066544], [-0.01564259436468614, 0.02870009436468614], [-0.015494096729141833, 0.028542221729141833], [-0.015321928201526034, 0.028284428201526036], [-0.015141591474046014, 0.02792971647404601], [-0.014983124917233161, 0.02749312491723316], [-0.014893165186189192, 0.02701629018618919], [-0.014893308885125746, 0.026525183885125744], [-0.015005350269383131, 0.02604097526938313], [-0.015205165461357516, 0.025566415461357517], [-0.015477730464552282, 0.02509273046455228], [-0.015764327038068543, 0.024587452038068544], [-0.016003453462392347, 0.024025953462392342], [-0.0161680499732053, 0.023403674973205304], [-0.01621968566188263, 0.022723435661882625], [-0.016162490014402835, 0.022017490014402834], [-0.016023158030890963, 0.021341283030890962], [-0.015845786646416803, 0.0207589116464168], [-0.01569263328551246, 0.020348258285512455], [-0.015594981746192402, 0.020153106746192403], [-0.015595432431927446, 0.020205432431927448], [-0.015695162776436462, 0.020497037776436466], [-0.015877475589894337, 0.020992475589894338], [-0.016093826101258365, 0.021620701101258365], [-0.01631060911633407, 0.022312484116334068], [-0.016498137966657136, 0.023011262966657137], [-0.016627616598061314, 0.023655116598061313], [-0.016696522350849653, 0.024217772350849653], [-0.01671467100159018, 0.02467779600159018], [-0.016681901608578686, 0.025032526608578687], [-0.016614765518784203, 0.025276015518784205], [-0.01652684358098709, 0.025427468580987093], [-0.01642030501358744, 0.025491555013587443], [-0.0162960274498826, 0.025471027449882597], [-0.016144268531277102, 0.025376768531277107], [-0.015969679247991888, 0.025225304247991884], [-0.01575823179357756, 0.02503135679357756], [-0.015509353828164756, 0.024804353828164753], [-0.0152479232181379, 0.0245941732181379], [-0.014959212742292349, 0.02439858774229235], [-0.014675974088108168, 0.024268474088108165], [-0.014415899445869455, 0.024224024445869456], [-0.014179583689370914, 0.02428145868937091], [-0.01399063771621728, 0.024453762716217284], [-0.013836594108523368, 0.02473034410852337], [-0.013719088564294097, 0.025093463564294094], [-0.013625344990931543, 0.025517219990931546], [-0.013538554901586706, 0.025968554901586702], [-0.013455770047825114, 0.02642077004782511], [-0.013366275141652107, 0.026835650141652106], [-0.013288391555980564, 0.027209641555980567], [-0.01322850562563373, 0.027526630625633725], [-0.01321332237072971, 0.027797697370729714], [-0.013263388823922789, 0.028027138823922788], [-0.013418119014989315, 0.028254994014989314], [-0.013687281107159499, 0.0284885311071595], [-0.014081995398199633, 0.028754495398199638], [-0.014569917353290399, 0.0290361673532904], [-0.015141302752835232, 0.02934317775283523], [-0.015733081257448427, 0.02963620625744843], [-0.016290725114489785, 0.02988697511448979], [-0.016758548684960826, 0.03006229868496083]], 'B612': [[-0.017095228781793037, 0.030134603781793036], [-0.017275271002723955, 0.030104021002723955], [-0.017301088723121957, 0.02996921372312196], [-0.017184445791507758, 0.02975694579150776], [-0.016972606362708413, 0.02950760636270841], [-0.016718333958153996, 0.029267083958153993], [-0.016453702731043345, 0.029063702731043348], [-0.01622278542622513, 0.02892653542622513], [-0.016039195797577604, 0.0288491957975776], [-0.015894771299909325, 0.028811646299909326], [-0.01577220959406654, 0.028777209594066544], [-0.01564259436468614, 0.02870009436468614], [-0.015494096729141833, 0.028542221729141833], [-0.015321928201526034, 0.028284428201526036], [-0.015141591474046014, 0.02792971647404601], [-0.014983124917233161, 0.02749312491723316], [-0.014893165186189192, 0.02701629018618919], [-0.014893308885125746, 0.026525183885125744], [-0.015005350269383131, 0.02604097526938313], [-0.015205165461357516, 0.025566415461357517], [-0.015477730464552282, 0.02509273046455228], [-0.015764327038068543, 0.024587452038068544], [-0.016003453462392347, 0.024025953462392342], [-0.0161680499732053, 0.023403674973205304], [-0.01621968566188263, 0.022723435661882625], [-0.016162490014402835, 0.022017490014402834], [-0.016023158030890963, 0.021341283030890962], [-0.015845786646416803, 0.0207589116464168], [-0.01569263328551246, 0.020348258285512455], [-0.015594981746192402, 0.020153106746192403], [-0.015595432431927446, 0.020205432431927448], [-0.015695162776436462, 0.020497037776436466], [-0.015877475589894337, 0.020992475589894338], [-0.016093826101258365, 0.021620701101258365], [-0.01631060911633407, 0.022312484116334068], [-0.016498137966657136, 0.023011262966657137], [-0.016627616598061314, 0.023655116598061313], [-0.016696522350849653, 0.024217772350849653], [-0.01671467100159018, 0.02467779600159018], [-0.016681901608578686, 0.025032526608578687], [-0.016614765518784203, 0.025276015518784205], [-0.01652684358098709, 0.025427468580987093], [-0.01642030501358744, 0.025491555013587443], [-0.0162960274498826, 0.025471027449882597], [-0.016144268531277102, 0.025376768531277107], [-0.015969679247991888, 0.025225304247991884], [-0.01575823179357756, 0.02503135679357756], [-0.015509353828164756, 0.024804353828164753], [-0.0152479232181379, 0.0245941732181379], [-0.014959212742292349, 0.02439858774229235], [-0.014675974088108168, 0.024268474088108165], [-0.014415899445869455, 0.024224024445869456], [-0.014179583689370914, 0.02428145868937091], [-0.01399063771621728, 0.024453762716217284], [-0.013836594108523368, 0.02473034410852337], [-0.013719088564294097, 0.025093463564294094], [-0.013625344990931543, 0.025517219990931546], [-0.013538554901586706, 0.025968554901586702], [-0.013455770047825114, 0.02642077004782511], [-0.013366275141652107, 0.026835650141652106], [-0.013288391555980564, 0.027209641555980567], [-0.01322850562563373, 0.027526630625633725], [-0.01321332237072971, 0.027797697370729714], [-0.013263388823922789, 0.028027138823922788], [-0.013418119014989315, 0.028254994014989314], [-0.013687281107159499, 0.0284885311071595], [-0.014081995398199633, 0.028754495398199638], [-0.014569917353290399, 0.0290361673532904], [-0.015141302752835232, 0.02934317775283523], [-0.015733081257448427, 0.02963620625744843], [-0.016290725114489785, 0.02988697511448979], [-0.016758548684960826, 0.03006229868496083]], 'B613': [[-0.017095228781793037, 0.030134603781793036], [-0.017275271002723955, 0.030104021002723955], [-0.017301088723121957, 0.02996921372312196], [-0.017184445791507758, 0.02975694579150776], [-0.016972606362708413, 0.02950760636270841], [-0.016718333958153996, 0.029267083958153993], [-0.016453702731043345, 0.029063702731043348], [-0.01622278542622513, 0.02892653542622513], [-0.016039195797577604, 0.0288491957975776], [-0.015894771299909325, 0.028811646299909326], [-0.01577220959406654, 0.028777209594066544], [-0.01564259436468614, 0.02870009436468614], [-0.015494096729141833, 0.028542221729141833], [-0.015321928201526034, 0.028284428201526036], [-0.015141591474046014, 0.02792971647404601], [-0.014983124917233161, 0.02749312491723316], [-0.014893165186189192, 0.02701629018618919], [-0.014893308885125746, 0.026525183885125744], [-0.015005350269383131, 0.02604097526938313], [-0.015205165461357516, 0.025566415461357517], [-0.015477730464552282, 0.02509273046455228], [-0.015764327038068543, 0.024587452038068544], [-0.016003453462392347, 0.024025953462392342], [-0.0161680499732053, 0.023403674973205304], [-0.01621968566188263, 0.022723435661882625], [-0.016162490014402835, 0.022017490014402834], [-0.016023158030890963, 0.021341283030890962], [-0.015845786646416803, 0.0207589116464168], [-0.01569263328551246, 0.020348258285512455], [-0.015594981746192402, 0.020153106746192403], [-0.015595432431927446, 0.020205432431927448], [-0.015695162776436462, 0.020497037776436466], [-0.015877475589894337, 0.020992475589894338], [-0.016093826101258365, 0.021620701101258365], [-0.01631060911633407, 0.022312484116334068], [-0.016498137966657136, 0.023011262966657137], [-0.016627616598061314, 0.023655116598061313], [-0.016696522350849653, 0.024217772350849653], [-0.01671467100159018, 0.02467779600159018], [-0.016681901608578686, 0.025032526608578687], [-0.016614765518784203, 0.025276015518784205], [-0.01652684358098709, 0.025427468580987093], [-0.01642030501358744, 0.025491555013587443], [-0.0162960274498826, 0.025471027449882597], [-0.016144268531277102, 0.025376768531277107], [-0.015969679247991888, 0.025225304247991884], [-0.01575823179357756, 0.02503135679357756], [-0.015509353828164756, 0.024804353828164753], [-0.0152479232181379, 0.0245941732181379], [-0.014959212742292349, 0.02439858774229235], [-0.014675974088108168, 0.024268474088108165], [-0.014415899445869455, 0.024224024445869456], [-0.014179583689370914, 0.02428145868937091], [-0.01399063771621728, 0.024453762716217284], [-0.013836594108523368, 0.02473034410852337], [-0.013719088564294097, 0.025093463564294094], [-0.013625344990931543, 0.025517219990931546], [-0.013538554901586706, 0.025968554901586702], [-0.013455770047825114, 0.02642077004782511], [-0.013366275141652107, 0.026835650141652106], [-0.013288391555980564, 0.027209641555980567], [-0.01322850562563373, 0.027526630625633725], [-0.01321332237072971, 0.027797697370729714], [-0.013263388823922789, 0.028027138823922788], [-0.013418119014989315, 0.028254994014989314], [-0.013687281107159499, 0.0284885311071595], [-0.014081995398199633, 0.028754495398199638], [-0.014569917353290399, 0.0290361673532904], [-0.015141302752835232, 0.02934317775283523], [-0.015733081257448427, 0.02963620625744843], [-0.016290725114489785, 0.02988697511448979], [-0.016758548684960826, 0.03006229868496083]], 'B614': [[-0.017095228781793037, 0.030134603781793036], [-0.017275271002723955, 0.030104021002723955], [-0.017301088723121957, 0.02996921372312196], [-0.017184445791507758, 0.02975694579150776], [-0.016972606362708413, 0.02950760636270841], [-0.016718333958153996, 0.029267083958153993], [-0.016453702731043345, 0.029063702731043348], [-0.01622278542622513, 0.02892653542622513], [-0.016039195797577604, 0.0288491957975776], [-0.015894771299909325, 0.028811646299909326], [-0.01577220959406654, 0.028777209594066544], [-0.01564259436468614, 0.02870009436468614], [-0.015494096729141833, 0.028542221729141833], [-0.015321928201526034, 0.028284428201526036], [-0.015141591474046014, 0.02792971647404601], [-0.014983124917233161, 0.02749312491723316], [-0.014893165186189192, 0.02701629018618919], [-0.014893308885125746, 0.026525183885125744], [-0.015005350269383131, 0.02604097526938313], [-0.015205165461357516, 0.025566415461357517], [-0.015477730464552282, 0.02509273046455228], [-0.015764327038068543, 0.024587452038068544], [-0.016003453462392347, 0.024025953462392342], [-0.0161680499732053, 0.023403674973205304], [-0.01621968566188263, 0.022723435661882625], [-0.016162490014402835, 0.022017490014402834], [-0.016023158030890963, 0.021341283030890962], [-0.015845786646416803, 0.0207589116464168], [-0.01569263328551246, 0.020348258285512455], [-0.015594981746192402, 0.020153106746192403], [-0.015595432431927446, 0.020205432431927448], [-0.015695162776436462, 0.020497037776436466], [-0.015877475589894337, 0.020992475589894338], [-0.016093826101258365, 0.021620701101258365], [-0.01631060911633407, 0.022312484116334068], [-0.016498137966657136, 0.023011262966657137], [-0.016627616598061314, 0.023655116598061313], [-0.016696522350849653, 0.024217772350849653], [-0.01671467100159018, 0.02467779600159018], [-0.016681901608578686, 0.025032526608578687], [-0.016614765518784203, 0.025276015518784205], [-0.01652684358098709, 0.025427468580987093], [-0.01642030501358744, 0.025491555013587443], [-0.0162960274498826, 0.025471027449882597], [-0.016144268531277102, 0.025376768531277107], [-0.015969679247991888, 0.025225304247991884], [-0.01575823179357756, 0.02503135679357756], [-0.015509353828164756, 0.024804353828164753], [-0.0152479232181379, 0.0245941732181379], [-0.014959212742292349, 0.02439858774229235], [-0.014675974088108168, 0.024268474088108165], [-0.014415899445869455, 0.024224024445869456], [-0.014179583689370914, 0.02428145868937091], [-0.01399063771621728, 0.024453762716217284], [-0.013836594108523368, 0.02473034410852337], [-0.013719088564294097, 0.025093463564294094], [-0.013625344990931543, 0.025517219990931546], [-0.013538554901586706, 0.025968554901586702], [-0.013455770047825114, 0.02642077004782511], [-0.013366275141652107, 0.026835650141652106], [-0.013288391555980564, 0.027209641555980567], [-0.01322850562563373, 0.027526630625633725], [-0.01321332237072971, 0.027797697370729714], [-0.013263388823922789, 0.028027138823922788], [-0.013418119014989315, 0.028254994014989314], [-0.013687281107159499, 0.0284885311071595], [-0.014081995398199633, 0.028754495398199638], [-0.014569917353290399, 0.0290361673532904], [-0.015141302752835232, 0.02934317775283523], [-0.015733081257448427, 0.02963620625744843], [-0.016290725114489785, 0.02988697511448979], [-0.016758548684960826, 0.03006229868496083]], 'B616': [[-0.017095228781793037, 0.030134603781793036], [-0.017275271002723955, 0.030104021002723955], [-0.017301088723121957, 0.02996921372312196], [-0.017184445791507758, 0.02975694579150776], [-0.016972606362708413, 0.02950760636270841], [-0.016718333958153996, 0.029267083958153993], [-0.016453702731043345, 0.029063702731043348], [-0.01622278542622513, 0.02892653542622513], [-0.016039195797577604, 0.0288491957975776], [-0.015894771299909325, 0.028811646299909326], [-0.01577220959406654, 0.028777209594066544], [-0.01564259436468614, 0.02870009436468614], [-0.015494096729141833, 0.028542221729141833], [-0.015321928201526034, 0.028284428201526036], [-0.015141591474046014, 0.02792971647404601], [-0.014983124917233161, 0.02749312491723316], [-0.014893165186189192, 0.02701629018618919], [-0.014893308885125746, 0.026525183885125744], [-0.015005350269383131, 0.02604097526938313], [-0.015205165461357516, 0.025566415461357517], [-0.015477730464552282, 0.02509273046455228], [-0.015764327038068543, 0.024587452038068544], [-0.016003453462392347, 0.024025953462392342], [-0.0161680499732053, 0.023403674973205304], [-0.01621968566188263, 0.022723435661882625], [-0.016162490014402835, 0.022017490014402834], [-0.016023158030890963, 0.021341283030890962], [-0.015845786646416803, 0.0207589116464168], [-0.01569263328551246, 0.020348258285512455], [-0.015594981746192402, 0.020153106746192403], [-0.015595432431927446, 0.020205432431927448], [-0.015695162776436462, 0.020497037776436466], [-0.015877475589894337, 0.020992475589894338], [-0.016093826101258365, 0.021620701101258365], [-0.01631060911633407, 0.022312484116334068], [-0.016498137966657136, 0.023011262966657137], [-0.016627616598061314, 0.023655116598061313], [-0.016696522350849653, 0.024217772350849653], [-0.01671467100159018, 0.02467779600159018], [-0.016681901608578686, 0.025032526608578687], [-0.016614765518784203, 0.025276015518784205], [-0.01652684358098709, 0.025427468580987093], [-0.01642030501358744, 0.025491555013587443], [-0.0162960274498826, 0.025471027449882597], [-0.016144268531277102, 0.025376768531277107], [-0.015969679247991888, 0.025225304247991884], [-0.01575823179357756, 0.02503135679357756], [-0.015509353828164756, 0.024804353828164753], [-0.0152479232181379, 0.0245941732181379], [-0.014959212742292349, 0.02439858774229235], [-0.014675974088108168, 0.024268474088108165], [-0.014415899445869455, 0.024224024445869456], [-0.014179583689370914, 0.02428145868937091], [-0.01399063771621728, 0.024453762716217284], [-0.013836594108523368, 0.02473034410852337], [-0.013719088564294097, 0.025093463564294094], [-0.013625344990931543, 0.025517219990931546], [-0.013538554901586706, 0.025968554901586702], [-0.013455770047825114, 0.02642077004782511], [-0.013366275141652107, 0.026835650141652106], [-0.013288391555980564, 0.027209641555980567], [-0.01322850562563373, 0.027526630625633725], [-0.01321332237072971, 0.027797697370729714], [-0.013263388823922789, 0.028027138823922788], [-0.013418119014989315, 0.028254994014989314], [-0.013687281107159499, 0.0284885311071595], [-0.014081995398199633, 0.028754495398199638], [-0.014569917353290399, 0.0290361673532904], [-0.015141302752835232, 0.02934317775283523], [-0.015733081257448427, 0.02963620625744843], [-0.016290725114489785, 0.02988697511448979], [-0.016758548684960826, 0.03006229868496083]], 'B617': [[-0.017095228781793037, 0.030134603781793036], [-0.017275271002723955, 0.030104021002723955], [-0.017301088723121957, 0.02996921372312196], [-0.017184445791507758, 0.02975694579150776], [-0.016972606362708413, 0.02950760636270841], [-0.016718333958153996, 0.029267083958153993], [-0.016453702731043345, 0.029063702731043348], [-0.01622278542622513, 0.02892653542622513], [-0.016039195797577604, 0.0288491957975776], [-0.015894771299909325, 0.028811646299909326], [-0.01577220959406654, 0.028777209594066544], [-0.01564259436468614, 0.02870009436468614], [-0.015494096729141833, 0.028542221729141833], [-0.015321928201526034, 0.028284428201526036], [-0.015141591474046014, 0.02792971647404601], [-0.014983124917233161, 0.02749312491723316], [-0.014893165186189192, 0.02701629018618919], [-0.014893308885125746, 0.026525183885125744], [-0.015005350269383131, 0.02604097526938313], [-0.015205165461357516, 0.025566415461357517], [-0.015477730464552282, 0.02509273046455228], [-0.015764327038068543, 0.024587452038068544], [-0.016003453462392347, 0.024025953462392342], [-0.0161680499732053, 0.023403674973205304], [-0.01621968566188263, 0.022723435661882625], [-0.016162490014402835, 0.022017490014402834], [-0.016023158030890963, 0.021341283030890962], [-0.015845786646416803, 0.0207589116464168], [-0.01569263328551246, 0.020348258285512455], [-0.015594981746192402, 0.020153106746192403], [-0.015595432431927446, 0.020205432431927448], [-0.015695162776436462, 0.020497037776436466], [-0.015877475589894337, 0.020992475589894338], [-0.016093826101258365, 0.021620701101258365], [-0.01631060911633407, 0.022312484116334068], [-0.016498137966657136, 0.023011262966657137], [-0.016627616598061314, 0.023655116598061313], [-0.016696522350849653, 0.024217772350849653], [-0.01671467100159018, 0.02467779600159018], [-0.016681901608578686, 0.025032526608578687], [-0.016614765518784203, 0.025276015518784205], [-0.01652684358098709, 0.025427468580987093], [-0.01642030501358744, 0.025491555013587443], [-0.0162960274498826, 0.025471027449882597], [-0.016144268531277102, 0.025376768531277107], [-0.015969679247991888, 0.025225304247991884], [-0.01575823179357756, 0.02503135679357756], [-0.015509353828164756, 0.024804353828164753], [-0.0152479232181379, 0.0245941732181379], [-0.014959212742292349, 0.02439858774229235], [-0.014675974088108168, 0.024268474088108165], [-0.014415899445869455, 0.024224024445869456], [-0.014179583689370914, 0.02428145868937091], [-0.01399063771621728, 0.024453762716217284], [-0.013836594108523368, 0.02473034410852337], [-0.013719088564294097, 0.025093463564294094], [-0.013625344990931543, 0.025517219990931546], [-0.013538554901586706, 0.025968554901586702], [-0.013455770047825114, 0.02642077004782511], [-0.013366275141652107, 0.026835650141652106], [-0.013288391555980564, 0.027209641555980567], [-0.01322850562563373, 0.027526630625633725], [-0.01321332237072971, 0.027797697370729714], [-0.013263388823922789, 0.028027138823922788], [-0.013418119014989315, 0.028254994014989314], [-0.013687281107159499, 0.0284885311071595], [-0.014081995398199633, 0.028754495398199638], [-0.014569917353290399, 0.0290361673532904], [-0.015141302752835232, 0.02934317775283523], [-0.015733081257448427, 0.02963620625744843], [-0.016290725114489785, 0.02988697511448979], [-0.016758548684960826, 0.03006229868496083]], 'C501': [[-0.017095228781793037, 0.030134603781793036], [-0.017275271002723955, 0.030104021002723955], [-0.017301088723121957, 0.02996921372312196], [-0.017184445791507758, 0.02975694579150776], [-0.016972606362708413, 0.02950760636270841], [-0.016718333958153996, 0.029267083958153993], [-0.016453702731043345, 0.029063702731043348], [-0.01622278542622513, 0.02892653542622513], [-0.016039195797577604, 0.0288491957975776], [-0.015894771299909325, 0.028811646299909326], [-0.01577220959406654, 0.028777209594066544], [-0.01564259436468614, 0.02870009436468614], [-0.015494096729141833, 0.028542221729141833], [-0.015321928201526034, 0.028284428201526036], [-0.015141591474046014, 0.02792971647404601], [-0.014983124917233161, 0.02749312491723316], [-0.014893165186189192, 0.02701629018618919], [-0.014893308885125746, 0.026525183885125744], [-0.015005350269383131, 0.02604097526938313], [-0.015205165461357516, 0.025566415461357517], [-0.015477730464552282, 0.02509273046455228], [-0.015764327038068543, 0.024587452038068544], [-0.016003453462392347, 0.024025953462392342], [-0.0161680499732053, 0.023403674973205304], [-0.01621968566188263, 0.022723435661882625], [-0.016162490014402835, 0.022017490014402834], [-0.016023158030890963, 0.021341283030890962], [-0.015845786646416803, 0.0207589116464168], [-0.01569263328551246, 0.020348258285512455], [-0.015594981746192402, 0.020153106746192403], [-0.015595432431927446, 0.020205432431927448], [-0.015695162776436462, 0.020497037776436466], [-0.015877475589894337, 0.020992475589894338], [-0.016093826101258365, 0.021620701101258365], [-0.01631060911633407, 0.022312484116334068], [-0.016498137966657136, 0.023011262966657137], [-0.016627616598061314, 0.023655116598061313], [-0.016696522350849653, 0.024217772350849653], [-0.01671467100159018, 0.02467779600159018], [-0.016681901608578686, 0.025032526608578687], [-0.016614765518784203, 0.025276015518784205], [-0.01652684358098709, 0.025427468580987093], [-0.01642030501358744, 0.025491555013587443], [-0.0162960274498826, 0.025471027449882597], [-0.016144268531277102, 0.025376768531277107], [-0.015969679247991888, 0.025225304247991884], [-0.01575823179357756, 0.02503135679357756], [-0.015509353828164756, 0.024804353828164753], [-0.0152479232181379, 0.0245941732181379], [-0.014959212742292349, 0.02439858774229235], [-0.014675974088108168, 0.024268474088108165], [-0.014415899445869455, 0.024224024445869456], [-0.014179583689370914, 0.02428145868937091], [-0.01399063771621728, 0.024453762716217284], [-0.013836594108523368, 0.02473034410852337], [-0.013719088564294097, 0.025093463564294094], [-0.013625344990931543, 0.025517219990931546], [-0.013538554901586706, 0.025968554901586702], [-0.013455770047825114, 0.02642077004782511], [-0.013366275141652107, 0.026835650141652106], [-0.013288391555980564, 0.027209641555980567], [-0.01322850562563373, 0.027526630625633725], [-0.01321332237072971, 0.027797697370729714], [-0.013263388823922789, 0.028027138823922788], [-0.013418119014989315, 0.028254994014989314], [-0.013687281107159499, 0.0284885311071595], [-0.014081995398199633, 0.028754495398199638], [-0.014569917353290399, 0.0290361673532904], [-0.015141302752835232, 0.02934317775283523], [-0.015733081257448427, 0.02963620625744843], [-0.016290725114489785, 0.02988697511448979], [-0.016758548684960826, 0.03006229868496083]], 'C502': [[-0.017095228781793037, 0.030134603781793036], [-0.017275271002723955, 0.030104021002723955], [-0.017301088723121957, 0.02996921372312196], [-0.017184445791507758, 0.02975694579150776], [-0.016972606362708413, 0.02950760636270841], [-0.016718333958153996, 0.029267083958153993], [-0.016453702731043345, 0.029063702731043348], [-0.01622278542622513, 0.02892653542622513], [-0.016039195797577604, 0.0288491957975776], [-0.015894771299909325, 0.028811646299909326], [-0.01577220959406654, 0.028777209594066544], [-0.01564259436468614, 0.02870009436468614], [-0.015494096729141833, 0.028542221729141833], [-0.015321928201526034, 0.028284428201526036], [-0.015141591474046014, 0.02792971647404601], [-0.014983124917233161, 0.02749312491723316], [-0.014893165186189192, 0.02701629018618919], [-0.014893308885125746, 0.026525183885125744], [-0.015005350269383131, 0.02604097526938313], [-0.015205165461357516, 0.025566415461357517], [-0.015477730464552282, 0.02509273046455228], [-0.015764327038068543, 0.024587452038068544], [-0.016003453462392347, 0.024025953462392342], [-0.0161680499732053, 0.023403674973205304], [-0.01621968566188263, 0.022723435661882625], [-0.016162490014402835, 0.022017490014402834], [-0.016023158030890963, 0.021341283030890962], [-0.015845786646416803, 0.0207589116464168], [-0.01569263328551246, 0.020348258285512455], [-0.015594981746192402, 0.020153106746192403], [-0.015595432431927446, 0.020205432431927448], [-0.015695162776436462, 0.020497037776436466], [-0.015877475589894337, 0.020992475589894338], [-0.016093826101258365, 0.021620701101258365], [-0.01631060911633407, 0.022312484116334068], [-0.016498137966657136, 0.023011262966657137], [-0.016627616598061314, 0.023655116598061313], [-0.016696522350849653, 0.024217772350849653], [-0.01671467100159018, 0.02467779600159018], [-0.016681901608578686, 0.025032526608578687], [-0.016614765518784203, 0.025276015518784205], [-0.01652684358098709, 0.025427468580987093], [-0.01642030501358744, 0.025491555013587443], [-0.0162960274498826, 0.025471027449882597], [-0.016144268531277102, 0.025376768531277107], [-0.015969679247991888, 0.025225304247991884], [-0.01575823179357756, 0.02503135679357756], [-0.015509353828164756, 0.024804353828164753], [-0.0152479232181379, 0.0245941732181379], [-0.014959212742292349, 0.02439858774229235], [-0.014675974088108168, 0.024268474088108165], [-0.014415899445869455, 0.024224024445869456], [-0.014179583689370914, 0.02428145868937091], [-0.01399063771621728, 0.024453762716217284], [-0.013836594108523368, 0.02473034410852337], [-0.013719088564294097, 0.025093463564294094], [-0.013625344990931543, 0.025517219990931546], [-0.013538554901586706, 0.025968554901586702], [-0.013455770047825114, 0.02642077004782511], [-0.013366275141652107, 0.026835650141652106], [-0.013288391555980564, 0.027209641555980567], [-0.01322850562563373, 0.027526630625633725], [-0.01321332237072971, 0.027797697370729714], [-0.013263388823922789, 0.028027138823922788], [-0.013418119014989315, 0.028254994014989314], [-0.013687281107159499, 0.0284885311071595], [-0.014081995398199633, 0.028754495398199638], [-0.014569917353290399, 0.0290361673532904], [-0.015141302752835232, 0.02934317775283523], [-0.015733081257448427, 0.02963620625744843], [-0.016290725114489785, 0.02988697511448979], [-0.016758548684960826, 0.03006229868496083]], 'C503': [[-0.017095228781793037, 0.030134603781793036], [-0.017275271002723955, 0.030104021002723955], [-0.017301088723121957, 0.02996921372312196], [-0.017184445791507758, 0.02975694579150776], [-0.016972606362708413, 0.02950760636270841], [-0.016718333958153996, 0.029267083958153993], [-0.016453702731043345, 0.029063702731043348], [-0.01622278542622513, 0.02892653542622513], [-0.016039195797577604, 0.0288491957975776], [-0.015894771299909325, 0.028811646299909326], [-0.01577220959406654, 0.028777209594066544], [-0.01564259436468614, 0.02870009436468614], [-0.015494096729141833, 0.028542221729141833], [-0.015321928201526034, 0.028284428201526036], [-0.015141591474046014, 0.02792971647404601], [-0.014983124917233161, 0.02749312491723316], [-0.014893165186189192, 0.02701629018618919], [-0.014893308885125746, 0.026525183885125744], [-0.015005350269383131, 0.02604097526938313], [-0.015205165461357516, 0.025566415461357517], [-0.015477730464552282, 0.02509273046455228], [-0.015764327038068543, 0.024587452038068544], [-0.016003453462392347, 0.024025953462392342], [-0.0161680499732053, 0.023403674973205304], [-0.01621968566188263, 0.022723435661882625], [-0.016162490014402835, 0.022017490014402834], [-0.016023158030890963, 0.021341283030890962], [-0.015845786646416803, 0.0207589116464168], [-0.01569263328551246, 0.020348258285512455], [-0.015594981746192402, 0.020153106746192403], [-0.015595432431927446, 0.020205432431927448], [-0.015695162776436462, 0.020497037776436466], [-0.015877475589894337, 0.020992475589894338], [-0.016093826101258365, 0.021620701101258365], [-0.01631060911633407, 0.022312484116334068], [-0.016498137966657136, 0.023011262966657137], [-0.016627616598061314, 0.023655116598061313], [-0.016696522350849653, 0.024217772350849653], [-0.01671467100159018, 0.02467779600159018], [-0.016681901608578686, 0.025032526608578687], [-0.016614765518784203, 0.025276015518784205], [-0.01652684358098709, 0.025427468580987093], [-0.01642030501358744, 0.025491555013587443], [-0.0162960274498826, 0.025471027449882597], [-0.016144268531277102, 0.025376768531277107], [-0.015969679247991888, 0.025225304247991884], [-0.01575823179357756, 0.02503135679357756], [-0.015509353828164756, 0.024804353828164753], [-0.0152479232181379, 0.0245941732181379], [-0.014959212742292349, 0.02439858774229235], [-0.014675974088108168, 0.024268474088108165], [-0.014415899445869455, 0.024224024445869456], [-0.014179583689370914, 0.02428145868937091], [-0.01399063771621728, 0.024453762716217284], [-0.013836594108523368, 0.02473034410852337], [-0.013719088564294097, 0.025093463564294094], [-0.013625344990931543, 0.025517219990931546], [-0.013538554901586706, 0.025968554901586702], [-0.013455770047825114, 0.02642077004782511], [-0.013366275141652107, 0.026835650141652106], [-0.013288391555980564, 0.027209641555980567], [-0.01322850562563373, 0.027526630625633725], [-0.01321332237072971, 0.027797697370729714], [-0.013263388823922789, 0.028027138823922788], [-0.013418119014989315, 0.028254994014989314], [-0.013687281107159499, 0.0284885311071595], [-0.014081995398199633, 0.028754495398199638], [-0.014569917353290399, 0.0290361673532904], [-0.015141302752835232, 0.02934317775283523], [-0.015733081257448427, 0.02963620625744843], [-0.016290725114489785, 0.02988697511448979], [-0.016758548684960826, 0.03006229868496083]], 'C504': [[-0.017095228781793037, 0.030134603781793036], [-0.017275271002723955, 0.030104021002723955], [-0.017301088723121957, 0.02996921372312196], [-0.017184445791507758, 0.02975694579150776], [-0.016972606362708413, 0.02950760636270841], [-0.016718333958153996, 0.029267083958153993], [-0.016453702731043345, 0.029063702731043348], [-0.01622278542622513, 0.02892653542622513], [-0.016039195797577604, 0.0288491957975776], [-0.015894771299909325, 0.028811646299909326], [-0.01577220959406654, 0.028777209594066544], [-0.01564259436468614, 0.02870009436468614], [-0.015494096729141833, 0.028542221729141833], [-0.015321928201526034, 0.028284428201526036], [-0.015141591474046014, 0.02792971647404601], [-0.014983124917233161, 0.02749312491723316], [-0.014893165186189192, 0.02701629018618919], [-0.014893308885125746, 0.026525183885125744], [-0.015005350269383131, 0.02604097526938313], [-0.015205165461357516, 0.025566415461357517], [-0.015477730464552282, 0.02509273046455228], [-0.015764327038068543, 0.024587452038068544], [-0.016003453462392347, 0.024025953462392342], [-0.0161680499732053, 0.023403674973205304], [-0.01621968566188263, 0.022723435661882625], [-0.016162490014402835, 0.022017490014402834], [-0.016023158030890963, 0.021341283030890962], [-0.015845786646416803, 0.0207589116464168], [-0.01569263328551246, 0.020348258285512455], [-0.015594981746192402, 0.020153106746192403], [-0.015595432431927446, 0.020205432431927448], [-0.015695162776436462, 0.020497037776436466], [-0.015877475589894337, 0.020992475589894338], [-0.016093826101258365, 0.021620701101258365], [-0.01631060911633407, 0.022312484116334068], [-0.016498137966657136, 0.023011262966657137], [-0.016627616598061314, 0.023655116598061313], [-0.016696522350849653, 0.024217772350849653], [-0.01671467100159018, 0.02467779600159018], [-0.016681901608578686, 0.025032526608578687], [-0.016614765518784203, 0.025276015518784205], [-0.01652684358098709, 0.025427468580987093], [-0.01642030501358744, 0.025491555013587443], [-0.0162960274498826, 0.025471027449882597], [-0.016144268531277102, 0.025376768531277107], [-0.015969679247991888, 0.025225304247991884], [-0.01575823179357756, 0.02503135679357756], [-0.015509353828164756, 0.024804353828164753], [-0.0152479232181379, 0.0245941732181379], [-0.014959212742292349, 0.02439858774229235], [-0.014675974088108168, 0.024268474088108165], [-0.014415899445869455, 0.024224024445869456], [-0.014179583689370914, 0.02428145868937091], [-0.01399063771621728, 0.024453762716217284], [-0.013836594108523368, 0.02473034410852337], [-0.013719088564294097, 0.025093463564294094], [-0.013625344990931543, 0.025517219990931546], [-0.013538554901586706, 0.025968554901586702], [-0.013455770047825114, 0.02642077004782511], [-0.013366275141652107, 0.026835650141652106], [-0.013288391555980564, 0.027209641555980567], [-0.01322850562563373, 0.027526630625633725], [-0.01321332237072971, 0.027797697370729714], [-0.013263388823922789, 0.028027138823922788], [-0.013418119014989315, 0.028254994014989314], [-0.013687281107159499, 0.0284885311071595], [-0.014081995398199633, 0.028754495398199638], [-0.014569917353290399, 0.0290361673532904], [-0.015141302752835232, 0.02934317775283523], [-0.015733081257448427, 0.02963620625744843], [-0.016290725114489785, 0.02988697511448979], [-0.016758548684960826, 0.03006229868496083]], 'C505': [[-0.017095228781793037, 0.030134603781793036], [-0.017275271002723955, 0.030104021002723955], [-0.017301088723121957, 0.02996921372312196], [-0.017184445791507758, 0.02975694579150776], [-0.016972606362708413, 0.02950760636270841], [-0.016718333958153996, 0.029267083958153993], [-0.016453702731043345, 0.029063702731043348], [-0.01622278542622513, 0.02892653542622513], [-0.016039195797577604, 0.0288491957975776], [-0.015894771299909325, 0.028811646299909326], [-0.01577220959406654, 0.028777209594066544], [-0.01564259436468614, 0.02870009436468614], [-0.015494096729141833, 0.028542221729141833], [-0.015321928201526034, 0.028284428201526036], [-0.015141591474046014, 0.02792971647404601], [-0.014983124917233161, 0.02749312491723316], [-0.014893165186189192, 0.02701629018618919], [-0.014893308885125746, 0.026525183885125744], [-0.015005350269383131, 0.02604097526938313], [-0.015205165461357516, 0.025566415461357517], [-0.015477730464552282, 0.02509273046455228], [-0.015764327038068543, 0.024587452038068544], [-0.016003453462392347, 0.024025953462392342], [-0.0161680499732053, 0.023403674973205304], [-0.01621968566188263, 0.022723435661882625], [-0.016162490014402835, 0.022017490014402834], [-0.016023158030890963, 0.021341283030890962], [-0.015845786646416803, 0.0207589116464168], [-0.01569263328551246, 0.020348258285512455], [-0.015594981746192402, 0.020153106746192403], [-0.015595432431927446, 0.020205432431927448], [-0.015695162776436462, 0.020497037776436466], [-0.015877475589894337, 0.020992475589894338], [-0.016093826101258365, 0.021620701101258365], [-0.01631060911633407, 0.022312484116334068], [-0.016498137966657136, 0.023011262966657137], [-0.016627616598061314, 0.023655116598061313], [-0.016696522350849653, 0.024217772350849653], [-0.01671467100159018, 0.02467779600159018], [-0.016681901608578686, 0.025032526608578687], [-0.016614765518784203, 0.025276015518784205], [-0.01652684358098709, 0.025427468580987093], [-0.01642030501358744, 0.025491555013587443], [-0.0162960274498826, 0.025471027449882597], [-0.016144268531277102, 0.025376768531277107], [-0.015969679247991888, 0.025225304247991884], [-0.01575823179357756, 0.02503135679357756], [-0.015509353828164756, 0.024804353828164753], [-0.0152479232181379, 0.0245941732181379], [-0.014959212742292349, 0.02439858774229235], [-0.014675974088108168, 0.024268474088108165], [-0.014415899445869455, 0.024224024445869456], [-0.014179583689370914, 0.02428145868937091], [-0.01399063771621728, 0.024453762716217284], [-0.013836594108523368, 0.02473034410852337], [-0.013719088564294097, 0.025093463564294094], [-0.013625344990931543, 0.025517219990931546], [-0.013538554901586706, 0.025968554901586702], [-0.013455770047825114, 0.02642077004782511], [-0.013366275141652107, 0.026835650141652106], [-0.013288391555980564, 0.027209641555980567], [-0.01322850562563373, 0.027526630625633725], [-0.01321332237072971, 0.027797697370729714], [-0.013263388823922789, 0.028027138823922788], [-0.013418119014989315, 0.028254994014989314], [-0.013687281107159499, 0.0284885311071595], [-0.014081995398199633, 0.028754495398199638], [-0.014569917353290399, 0.0290361673532904], [-0.015141302752835232, 0.02934317775283523], [-0.015733081257448427, 0.02963620625744843], [-0.016290725114489785, 0.02988697511448979], [-0.016758548684960826, 0.03006229868496083]], 'C506': [[-0.017095228781793037, 0.030134603781793036], [-0.017275271002723955, 0.030104021002723955], [-0.017301088723121957, 0.02996921372312196], [-0.017184445791507758, 0.02975694579150776], [-0.016972606362708413, 0.02950760636270841], [-0.016718333958153996, 0.029267083958153993], [-0.016453702731043345, 0.029063702731043348], [-0.01622278542622513, 0.02892653542622513], [-0.016039195797577604, 0.0288491957975776], [-0.015894771299909325, 0.028811646299909326], [-0.01577220959406654, 0.028777209594066544], [-0.01564259436468614, 0.02870009436468614], [-0.015494096729141833, 0.028542221729141833], [-0.015321928201526034, 0.028284428201526036], [-0.015141591474046014, 0.02792971647404601], [-0.014983124917233161, 0.02749312491723316], [-0.014893165186189192, 0.02701629018618919], [-0.014893308885125746, 0.026525183885125744], [-0.015005350269383131, 0.02604097526938313], [-0.015205165461357516, 0.025566415461357517], [-0.015477730464552282, 0.02509273046455228], [-0.015764327038068543, 0.024587452038068544], [-0.016003453462392347, 0.024025953462392342], [-0.0161680499732053, 0.023403674973205304], [-0.01621968566188263, 0.022723435661882625], [-0.016162490014402835, 0.022017490014402834], [-0.016023158030890963, 0.021341283030890962], [-0.015845786646416803, 0.0207589116464168], [-0.01569263328551246, 0.020348258285512455], [-0.015594981746192402, 0.020153106746192403], [-0.015595432431927446, 0.020205432431927448], [-0.015695162776436462, 0.020497037776436466], [-0.015877475589894337, 0.020992475589894338], [-0.016093826101258365, 0.021620701101258365], [-0.01631060911633407, 0.022312484116334068], [-0.016498137966657136, 0.023011262966657137], [-0.016627616598061314, 0.023655116598061313], [-0.016696522350849653, 0.024217772350849653], [-0.01671467100159018, 0.02467779600159018], [-0.016681901608578686, 0.025032526608578687], [-0.016614765518784203, 0.025276015518784205], [-0.01652684358098709, 0.025427468580987093], [-0.01642030501358744, 0.025491555013587443], [-0.0162960274498826, 0.025471027449882597], [-0.016144268531277102, 0.025376768531277107], [-0.015969679247991888, 0.025225304247991884], [-0.01575823179357756, 0.02503135679357756], [-0.015509353828164756, 0.024804353828164753], [-0.0152479232181379, 0.0245941732181379], [-0.014959212742292349, 0.02439858774229235], [-0.014675974088108168, 0.024268474088108165], [-0.014415899445869455, 0.024224024445869456], [-0.014179583689370914, 0.02428145868937091], [-0.01399063771621728, 0.024453762716217284], [-0.013836594108523368, 0.02473034410852337], [-0.013719088564294097, 0.025093463564294094], [-0.013625344990931543, 0.025517219990931546], [-0.013538554901586706, 0.025968554901586702], [-0.013455770047825114, 0.02642077004782511], [-0.013366275141652107, 0.026835650141652106], [-0.013288391555980564, 0.027209641555980567], [-0.01322850562563373, 0.027526630625633725], [-0.01321332237072971, 0.027797697370729714], [-0.013263388823922789, 0.028027138823922788], [-0.013418119014989315, 0.028254994014989314], [-0.013687281107159499, 0.0284885311071595], [-0.014081995398199633, 0.028754495398199638], [-0.014569917353290399, 0.0290361673532904], [-0.015141302752835232, 0.02934317775283523], [-0.015733081257448427, 0.02963620625744843], [-0.016290725114489785, 0.02988697511448979], [-0.016758548684960826, 0.03006229868496083]], 'C507': [[-0.017095228781793037, 0.030134603781793036], [-0.017275271002723955, 0.030104021002723955], [-0.017301088723121957, 0.02996921372312196], [-0.017184445791507758, 0.02975694579150776], [-0.016972606362708413, 0.02950760636270841], [-0.016718333958153996, 0.029267083958153993], [-0.016453702731043345, 0.029063702731043348], [-0.01622278542622513, 0.02892653542622513], [-0.016039195797577604, 0.0288491957975776], [-0.015894771299909325, 0.028811646299909326], [-0.01577220959406654, 0.028777209594066544], [-0.01564259436468614, 0.02870009436468614], [-0.015494096729141833, 0.028542221729141833], [-0.015321928201526034, 0.028284428201526036], [-0.015141591474046014, 0.02792971647404601], [-0.014983124917233161, 0.02749312491723316], [-0.014893165186189192, 0.02701629018618919], [-0.014893308885125746, 0.026525183885125744], [-0.015005350269383131, 0.02604097526938313], [-0.015205165461357516, 0.025566415461357517], [-0.015477730464552282, 0.02509273046455228], [-0.015764327038068543, 0.024587452038068544], [-0.016003453462392347, 0.024025953462392342], [-0.0161680499732053, 0.023403674973205304], [-0.01621968566188263, 0.022723435661882625], [-0.016162490014402835, 0.022017490014402834], [-0.016023158030890963, 0.021341283030890962], [-0.015845786646416803, 0.0207589116464168], [-0.01569263328551246, 0.020348258285512455], [-0.015594981746192402, 0.020153106746192403], [-0.015595432431927446, 0.020205432431927448], [-0.015695162776436462, 0.020497037776436466], [-0.015877475589894337, 0.020992475589894338], [-0.016093826101258365, 0.021620701101258365], [-0.01631060911633407, 0.022312484116334068], [-0.016498137966657136, 0.023011262966657137], [-0.016627616598061314, 0.023655116598061313], [-0.016696522350849653, 0.024217772350849653], [-0.01671467100159018, 0.02467779600159018], [-0.016681901608578686, 0.025032526608578687], [-0.016614765518784203, 0.025276015518784205], [-0.01652684358098709, 0.025427468580987093], [-0.01642030501358744, 0.025491555013587443], [-0.0162960274498826, 0.025471027449882597], [-0.016144268531277102, 0.025376768531277107], [-0.015969679247991888, 0.025225304247991884], [-0.01575823179357756, 0.02503135679357756], [-0.015509353828164756, 0.024804353828164753], [-0.0152479232181379, 0.0245941732181379], [-0.014959212742292349, 0.02439858774229235], [-0.014675974088108168, 0.024268474088108165], [-0.014415899445869455, 0.024224024445869456], [-0.014179583689370914, 0.02428145868937091], [-0.01399063771621728, 0.024453762716217284], [-0.013836594108523368, 0.02473034410852337], [-0.013719088564294097, 0.025093463564294094], [-0.013625344990931543, 0.025517219990931546], [-0.013538554901586706, 0.025968554901586702], [-0.013455770047825114, 0.02642077004782511], [-0.013366275141652107, 0.026835650141652106], [-0.013288391555980564, 0.027209641555980567], [-0.01322850562563373, 0.027526630625633725], [-0.01321332237072971, 0.027797697370729714], [-0.013263388823922789, 0.028027138823922788], [-0.013418119014989315, 0.028254994014989314], [-0.013687281107159499, 0.0284885311071595], [-0.014081995398199633, 0.028754495398199638], [-0.014569917353290399, 0.0290361673532904], [-0.015141302752835232, 0.02934317775283523], [-0.015733081257448427, 0.02963620625744843], [-0.016290725114489785, 0.02988697511448979], [-0.016758548684960826, 0.03006229868496083]], 'C508': [[-0.017095228781793037, 0.030134603781793036], [-0.017275271002723955, 0.030104021002723955], [-0.017301088723121957, 0.02996921372312196], [-0.017184445791507758, 0.02975694579150776], [-0.016972606362708413, 0.02950760636270841], [-0.016718333958153996, 0.029267083958153993], [-0.016453702731043345, 0.029063702731043348], [-0.01622278542622513, 0.02892653542622513], [-0.016039195797577604, 0.0288491957975776], [-0.015894771299909325, 0.028811646299909326], [-0.01577220959406654, 0.028777209594066544], [-0.01564259436468614, 0.02870009436468614], [-0.015494096729141833, 0.028542221729141833], [-0.015321928201526034, 0.028284428201526036], [-0.015141591474046014, 0.02792971647404601], [-0.014983124917233161, 0.02749312491723316], [-0.014893165186189192, 0.02701629018618919], [-0.014893308885125746, 0.026525183885125744], [-0.015005350269383131, 0.02604097526938313], [-0.015205165461357516, 0.025566415461357517], [-0.015477730464552282, 0.02509273046455228], [-0.015764327038068543, 0.024587452038068544], [-0.016003453462392347, 0.024025953462392342], [-0.0161680499732053, 0.023403674973205304], [-0.01621968566188263, 0.022723435661882625], [-0.016162490014402835, 0.022017490014402834], [-0.016023158030890963, 0.021341283030890962], [-0.015845786646416803, 0.0207589116464168], [-0.01569263328551246, 0.020348258285512455], [-0.015594981746192402, 0.020153106746192403], [-0.015595432431927446, 0.020205432431927448], [-0.015695162776436462, 0.020497037776436466], [-0.015877475589894337, 0.020992475589894338], [-0.016093826101258365, 0.021620701101258365], [-0.01631060911633407, 0.022312484116334068], [-0.016498137966657136, 0.023011262966657137], [-0.016627616598061314, 0.023655116598061313], [-0.016696522350849653, 0.024217772350849653], [-0.01671467100159018, 0.02467779600159018], [-0.016681901608578686, 0.025032526608578687], [-0.016614765518784203, 0.025276015518784205], [-0.01652684358098709, 0.025427468580987093], [-0.01642030501358744, 0.025491555013587443], [-0.0162960274498826, 0.025471027449882597], [-0.016144268531277102, 0.025376768531277107], [-0.015969679247991888, 0.025225304247991884], [-0.01575823179357756, 0.02503135679357756], [-0.015509353828164756, 0.024804353828164753], [-0.0152479232181379, 0.0245941732181379], [-0.014959212742292349, 0.02439858774229235], [-0.014675974088108168, 0.024268474088108165], [-0.014415899445869455, 0.024224024445869456], [-0.014179583689370914, 0.02428145868937091], [-0.01399063771621728, 0.024453762716217284], [-0.013836594108523368, 0.02473034410852337], [-0.013719088564294097, 0.025093463564294094], [-0.013625344990931543, 0.025517219990931546], [-0.013538554901586706, 0.025968554901586702], [-0.013455770047825114, 0.02642077004782511], [-0.013366275141652107, 0.026835650141652106], [-0.013288391555980564, 0.027209641555980567], [-0.01322850562563373, 0.027526630625633725], [-0.01321332237072971, 0.027797697370729714], [-0.013263388823922789, 0.028027138823922788], [-0.013418119014989315, 0.028254994014989314], [-0.013687281107159499, 0.0284885311071595], [-0.014081995398199633, 0.028754495398199638], [-0.014569917353290399, 0.0290361673532904], [-0.015141302752835232, 0.02934317775283523], [-0.015733081257448427, 0.02963620625744843], [-0.016290725114489785, 0.02988697511448979], [-0.016758548684960826, 0.03006229868496083]], 'C509': [[-0.017095228781793037, 0.030134603781793036], [-0.017275271002723955, 0.030104021002723955], [-0.017301088723121957, 0.02996921372312196], [-0.017184445791507758, 0.02975694579150776], [-0.016972606362708413, 0.02950760636270841], [-0.016718333958153996, 0.029267083958153993], [-0.016453702731043345, 0.029063702731043348], [-0.01622278542622513, 0.02892653542622513], [-0.016039195797577604, 0.0288491957975776], [-0.015894771299909325, 0.028811646299909326], [-0.01577220959406654, 0.028777209594066544], [-0.01564259436468614, 0.02870009436468614], [-0.015494096729141833, 0.028542221729141833], [-0.015321928201526034, 0.028284428201526036], [-0.015141591474046014, 0.02792971647404601], [-0.014983124917233161, 0.02749312491723316], [-0.014893165186189192, 0.02701629018618919], [-0.014893308885125746, 0.026525183885125744], [-0.015005350269383131, 0.02604097526938313], [-0.015205165461357516, 0.025566415461357517], [-0.015477730464552282, 0.02509273046455228], [-0.015764327038068543, 0.024587452038068544], [-0.016003453462392347, 0.024025953462392342], [-0.0161680499732053, 0.023403674973205304], [-0.01621968566188263, 0.022723435661882625], [-0.016162490014402835, 0.022017490014402834], [-0.016023158030890963, 0.021341283030890962], [-0.015845786646416803, 0.0207589116464168], [-0.01569263328551246, 0.020348258285512455], [-0.015594981746192402, 0.020153106746192403], [-0.015595432431927446, 0.020205432431927448], [-0.015695162776436462, 0.020497037776436466], [-0.015877475589894337, 0.020992475589894338], [-0.016093826101258365, 0.021620701101258365], [-0.01631060911633407, 0.022312484116334068], [-0.016498137966657136, 0.023011262966657137], [-0.016627616598061314, 0.023655116598061313], [-0.016696522350849653, 0.024217772350849653], [-0.01671467100159018, 0.02467779600159018], [-0.016681901608578686, 0.025032526608578687], [-0.016614765518784203, 0.025276015518784205], [-0.01652684358098709, 0.025427468580987093], [-0.01642030501358744, 0.025491555013587443], [-0.0162960274498826, 0.025471027449882597], [-0.016144268531277102, 0.025376768531277107], [-0.015969679247991888, 0.025225304247991884], [-0.01575823179357756, 0.02503135679357756], [-0.015509353828164756, 0.024804353828164753], [-0.0152479232181379, 0.0245941732181379], [-0.014959212742292349, 0.02439858774229235], [-0.014675974088108168, 0.024268474088108165], [-0.014415899445869455, 0.024224024445869456], [-0.014179583689370914, 0.02428145868937091], [-0.01399063771621728, 0.024453762716217284], [-0.013836594108523368, 0.02473034410852337], [-0.013719088564294097, 0.025093463564294094], [-0.013625344990931543, 0.025517219990931546], [-0.013538554901586706, 0.025968554901586702], [-0.013455770047825114, 0.02642077004782511], [-0.013366275141652107, 0.026835650141652106], [-0.013288391555980564, 0.027209641555980567], [-0.01322850562563373, 0.027526630625633725], [-0.01321332237072971, 0.027797697370729714], [-0.013263388823922789, 0.028027138823922788], [-0.013418119014989315, 0.028254994014989314], [-0.013687281107159499, 0.0284885311071595], [-0.014081995398199633, 0.028754495398199638], [-0.014569917353290399, 0.0290361673532904], [-0.015141302752835232, 0.02934317775283523], [-0.015733081257448427, 0.02963620625744843], [-0.016290725114489785, 0.02988697511448979], [-0.016758548684960826, 0.03006229868496083]], 'C510': [[-0.017095228781793037, 0.030134603781793036], [-0.017275271002723955, 0.030104021002723955], [-0.017301088723121957, 0.02996921372312196], [-0.017184445791507758, 0.02975694579150776], [-0.016972606362708413, 0.02950760636270841], [-0.016718333958153996, 0.029267083958153993], [-0.016453702731043345, 0.029063702731043348], [-0.01622278542622513, 0.02892653542622513], [-0.016039195797577604, 0.0288491957975776], [-0.015894771299909325, 0.028811646299909326], [-0.01577220959406654, 0.028777209594066544], [-0.01564259436468614, 0.02870009436468614], [-0.015494096729141833, 0.028542221729141833], [-0.015321928201526034, 0.028284428201526036], [-0.015141591474046014, 0.02792971647404601], [-0.014983124917233161, 0.02749312491723316], [-0.014893165186189192, 0.02701629018618919], [-0.014893308885125746, 0.026525183885125744], [-0.015005350269383131, 0.02604097526938313], [-0.015205165461357516, 0.025566415461357517], [-0.015477730464552282, 0.02509273046455228], [-0.015764327038068543, 0.024587452038068544], [-0.016003453462392347, 0.024025953462392342], [-0.0161680499732053, 0.023403674973205304], [-0.01621968566188263, 0.022723435661882625], [-0.016162490014402835, 0.022017490014402834], [-0.016023158030890963, 0.021341283030890962], [-0.015845786646416803, 0.0207589116464168], [-0.01569263328551246, 0.020348258285512455], [-0.015594981746192402, 0.020153106746192403], [-0.015595432431927446, 0.020205432431927448], [-0.015695162776436462, 0.020497037776436466], [-0.015877475589894337, 0.020992475589894338], [-0.016093826101258365, 0.021620701101258365], [-0.01631060911633407, 0.022312484116334068], [-0.016498137966657136, 0.023011262966657137], [-0.016627616598061314, 0.023655116598061313], [-0.016696522350849653, 0.024217772350849653], [-0.01671467100159018, 0.02467779600159018], [-0.016681901608578686, 0.025032526608578687], [-0.016614765518784203, 0.025276015518784205], [-0.01652684358098709, 0.025427468580987093], [-0.01642030501358744, 0.025491555013587443], [-0.0162960274498826, 0.025471027449882597], [-0.016144268531277102, 0.025376768531277107], [-0.015969679247991888, 0.025225304247991884], [-0.01575823179357756, 0.02503135679357756], [-0.015509353828164756, 0.024804353828164753], [-0.0152479232181379, 0.0245941732181379], [-0.014959212742292349, 0.02439858774229235], [-0.014675974088108168, 0.024268474088108165], [-0.014415899445869455, 0.024224024445869456], [-0.014179583689370914, 0.02428145868937091], [-0.01399063771621728, 0.024453762716217284], [-0.013836594108523368, 0.02473034410852337], [-0.013719088564294097, 0.025093463564294094], [-0.013625344990931543, 0.025517219990931546], [-0.013538554901586706, 0.025968554901586702], [-0.013455770047825114, 0.02642077004782511], [-0.013366275141652107, 0.026835650141652106], [-0.013288391555980564, 0.027209641555980567], [-0.01322850562563373, 0.027526630625633725], [-0.01321332237072971, 0.027797697370729714], [-0.013263388823922789, 0.028027138823922788], [-0.013418119014989315, 0.028254994014989314], [-0.013687281107159499, 0.0284885311071595], [-0.014081995398199633, 0.028754495398199638], [-0.014569917353290399, 0.0290361673532904], [-0.015141302752835232, 0.02934317775283523], [-0.015733081257448427, 0.02963620625744843], [-0.016290725114489785, 0.02988697511448979], [-0.016758548684960826, 0.03006229868496083]], 'C511': [[-0.017095228781793037, 0.030134603781793036], [-0.017275271002723955, 0.030104021002723955], [-0.017301088723121957, 0.02996921372312196], [-0.017184445791507758, 0.02975694579150776], [-0.016972606362708413, 0.02950760636270841], [-0.016718333958153996, 0.029267083958153993], [-0.016453702731043345, 0.029063702731043348], [-0.01622278542622513, 0.02892653542622513], [-0.016039195797577604, 0.0288491957975776], [-0.015894771299909325, 0.028811646299909326], [-0.01577220959406654, 0.028777209594066544], [-0.01564259436468614, 0.02870009436468614], [-0.015494096729141833, 0.028542221729141833], [-0.015321928201526034, 0.028284428201526036], [-0.015141591474046014, 0.02792971647404601], [-0.014983124917233161, 0.02749312491723316], [-0.014893165186189192, 0.02701629018618919], [-0.014893308885125746, 0.026525183885125744], [-0.015005350269383131, 0.02604097526938313], [-0.015205165461357516, 0.025566415461357517], [-0.015477730464552282, 0.02509273046455228], [-0.015764327038068543, 0.024587452038068544], [-0.016003453462392347, 0.024025953462392342], [-0.0161680499732053, 0.023403674973205304], [-0.01621968566188263, 0.022723435661882625], [-0.016162490014402835, 0.022017490014402834], [-0.016023158030890963, 0.021341283030890962], [-0.015845786646416803, 0.0207589116464168], [-0.01569263328551246, 0.020348258285512455], [-0.015594981746192402, 0.020153106746192403], [-0.015595432431927446, 0.020205432431927448], [-0.015695162776436462, 0.020497037776436466], [-0.015877475589894337, 0.020992475589894338], [-0.016093826101258365, 0.021620701101258365], [-0.01631060911633407, 0.022312484116334068], [-0.016498137966657136, 0.023011262966657137], [-0.016627616598061314, 0.023655116598061313], [-0.016696522350849653, 0.024217772350849653], [-0.01671467100159018, 0.02467779600159018], [-0.016681901608578686, 0.025032526608578687], [-0.016614765518784203, 0.025276015518784205], [-0.01652684358098709, 0.025427468580987093], [-0.01642030501358744, 0.025491555013587443], [-0.0162960274498826, 0.025471027449882597], [-0.016144268531277102, 0.025376768531277107], [-0.015969679247991888, 0.025225304247991884], [-0.01575823179357756, 0.02503135679357756], [-0.015509353828164756, 0.024804353828164753], [-0.0152479232181379, 0.0245941732181379], [-0.014959212742292349, 0.02439858774229235], [-0.014675974088108168, 0.024268474088108165], [-0.014415899445869455, 0.024224024445869456], [-0.014179583689370914, 0.02428145868937091], [-0.01399063771621728, 0.024453762716217284], [-0.013836594108523368, 0.02473034410852337], [-0.013719088564294097, 0.025093463564294094], [-0.013625344990931543, 0.025517219990931546], [-0.013538554901586706, 0.025968554901586702], [-0.013455770047825114, 0.02642077004782511], [-0.013366275141652107, 0.026835650141652106], [-0.013288391555980564, 0.027209641555980567], [-0.01322850562563373, 0.027526630625633725], [-0.01321332237072971, 0.027797697370729714], [-0.013263388823922789, 0.028027138823922788], [-0.013418119014989315, 0.028254994014989314], [-0.013687281107159499, 0.0284885311071595], [-0.014081995398199633, 0.028754495398199638], [-0.014569917353290399, 0.0290361673532904], [-0.015141302752835232, 0.02934317775283523], [-0.015733081257448427, 0.02963620625744843], [-0.016290725114489785, 0.02988697511448979], [-0.016758548684960826, 0.03006229868496083]], 'C512': [[-0.017095228781793037, 0.030134603781793036], [-0.017275271002723955, 0.030104021002723955], [-0.017301088723121957, 0.02996921372312196], [-0.017184445791507758, 0.02975694579150776], [-0.016972606362708413, 0.02950760636270841], [-0.016718333958153996, 0.029267083958153993], [-0.016453702731043345, 0.029063702731043348], [-0.01622278542622513, 0.02892653542622513], [-0.016039195797577604, 0.0288491957975776], [-0.015894771299909325, 0.028811646299909326], [-0.01577220959406654, 0.028777209594066544], [-0.01564259436468614, 0.02870009436468614], [-0.015494096729141833, 0.028542221729141833], [-0.015321928201526034, 0.028284428201526036], [-0.015141591474046014, 0.02792971647404601], [-0.014983124917233161, 0.02749312491723316], [-0.014893165186189192, 0.02701629018618919], [-0.014893308885125746, 0.026525183885125744], [-0.015005350269383131, 0.02604097526938313], [-0.015205165461357516, 0.025566415461357517], [-0.015477730464552282, 0.02509273046455228], [-0.015764327038068543, 0.024587452038068544], [-0.016003453462392347, 0.024025953462392342], [-0.0161680499732053, 0.023403674973205304], [-0.01621968566188263, 0.022723435661882625], [-0.016162490014402835, 0.022017490014402834], [-0.016023158030890963, 0.021341283030890962], [-0.015845786646416803, 0.0207589116464168], [-0.01569263328551246, 0.020348258285512455], [-0.015594981746192402, 0.020153106746192403], [-0.015595432431927446, 0.020205432431927448], [-0.015695162776436462, 0.020497037776436466], [-0.015877475589894337, 0.020992475589894338], [-0.016093826101258365, 0.021620701101258365], [-0.01631060911633407, 0.022312484116334068], [-0.016498137966657136, 0.023011262966657137], [-0.016627616598061314, 0.023655116598061313], [-0.016696522350849653, 0.024217772350849653], [-0.01671467100159018, 0.02467779600159018], [-0.016681901608578686, 0.025032526608578687], [-0.016614765518784203, 0.025276015518784205], [-0.01652684358098709, 0.025427468580987093], [-0.01642030501358744, 0.025491555013587443], [-0.0162960274498826, 0.025471027449882597], [-0.016144268531277102, 0.025376768531277107], [-0.015969679247991888, 0.025225304247991884], [-0.01575823179357756, 0.02503135679357756], [-0.015509353828164756, 0.024804353828164753], [-0.0152479232181379, 0.0245941732181379], [-0.014959212742292349, 0.02439858774229235], [-0.014675974088108168, 0.024268474088108165], [-0.014415899445869455, 0.024224024445869456], [-0.014179583689370914, 0.02428145868937091], [-0.01399063771621728, 0.024453762716217284], [-0.013836594108523368, 0.02473034410852337], [-0.013719088564294097, 0.025093463564294094], [-0.013625344990931543, 0.025517219990931546], [-0.013538554901586706, 0.025968554901586702], [-0.013455770047825114, 0.02642077004782511], [-0.013366275141652107, 0.026835650141652106], [-0.013288391555980564, 0.027209641555980567], [-0.01322850562563373, 0.027526630625633725], [-0.01321332237072971, 0.027797697370729714], [-0.013263388823922789, 0.028027138823922788], [-0.013418119014989315, 0.028254994014989314], [-0.013687281107159499, 0.0284885311071595], [-0.014081995398199633, 0.028754495398199638], [-0.014569917353290399, 0.0290361673532904], [-0.015141302752835232, 0.02934317775283523], [-0.015733081257448427, 0.02963620625744843], [-0.016290725114489785, 0.02988697511448979], [-0.016758548684960826, 0.03006229868496083]], 'C513': [[-0.017095228781793037, 0.030134603781793036], [-0.017275271002723955, 0.030104021002723955], [-0.017301088723121957, 0.02996921372312196], [-0.017184445791507758, 0.02975694579150776], [-0.016972606362708413, 0.02950760636270841], [-0.016718333958153996, 0.029267083958153993], [-0.016453702731043345, 0.029063702731043348], [-0.01622278542622513, 0.02892653542622513], [-0.016039195797577604, 0.0288491957975776], [-0.015894771299909325, 0.028811646299909326], [-0.01577220959406654, 0.028777209594066544], [-0.01564259436468614, 0.02870009436468614], [-0.015494096729141833, 0.028542221729141833], [-0.015321928201526034, 0.028284428201526036], [-0.015141591474046014, 0.02792971647404601], [-0.014983124917233161, 0.02749312491723316], [-0.014893165186189192, 0.02701629018618919], [-0.014893308885125746, 0.026525183885125744], [-0.015005350269383131, 0.02604097526938313], [-0.015205165461357516, 0.025566415461357517], [-0.015477730464552282, 0.02509273046455228], [-0.015764327038068543, 0.024587452038068544], [-0.016003453462392347, 0.024025953462392342], [-0.0161680499732053, 0.023403674973205304], [-0.01621968566188263, 0.022723435661882625], [-0.016162490014402835, 0.022017490014402834], [-0.016023158030890963, 0.021341283030890962], [-0.015845786646416803, 0.0207589116464168], [-0.01569263328551246, 0.020348258285512455], [-0.015594981746192402, 0.020153106746192403], [-0.015595432431927446, 0.020205432431927448], [-0.015695162776436462, 0.020497037776436466], [-0.015877475589894337, 0.020992475589894338], [-0.016093826101258365, 0.021620701101258365], [-0.01631060911633407, 0.022312484116334068], [-0.016498137966657136, 0.023011262966657137], [-0.016627616598061314, 0.023655116598061313], [-0.016696522350849653, 0.024217772350849653], [-0.01671467100159018, 0.02467779600159018], [-0.016681901608578686, 0.025032526608578687], [-0.016614765518784203, 0.025276015518784205], [-0.01652684358098709, 0.025427468580987093], [-0.01642030501358744, 0.025491555013587443], [-0.0162960274498826, 0.025471027449882597], [-0.016144268531277102, 0.025376768531277107], [-0.015969679247991888, 0.025225304247991884], [-0.01575823179357756, 0.02503135679357756], [-0.015509353828164756, 0.024804353828164753], [-0.0152479232181379, 0.0245941732181379], [-0.014959212742292349, 0.02439858774229235], [-0.014675974088108168, 0.024268474088108165], [-0.014415899445869455, 0.024224024445869456], [-0.014179583689370914, 0.02428145868937091], [-0.01399063771621728, 0.024453762716217284], [-0.013836594108523368, 0.02473034410852337], [-0.013719088564294097, 0.025093463564294094], [-0.013625344990931543, 0.025517219990931546], [-0.013538554901586706, 0.025968554901586702], [-0.013455770047825114, 0.02642077004782511], [-0.013366275141652107, 0.026835650141652106], [-0.013288391555980564, 0.027209641555980567], [-0.01322850562563373, 0.027526630625633725], [-0.01321332237072971, 0.027797697370729714], [-0.013263388823922789, 0.028027138823922788], [-0.013418119014989315, 0.028254994014989314], [-0.013687281107159499, 0.0284885311071595], [-0.014081995398199633, 0.028754495398199638], [-0.014569917353290399, 0.0290361673532904], [-0.015141302752835232, 0.02934317775283523], [-0.015733081257448427, 0.02963620625744843], [-0.016290725114489785, 0.02988697511448979], [-0.016758548684960826, 0.03006229868496083]], 'D402': [[-0.017095228781793037, 0.030134603781793036], [-0.017275271002723955, 0.030104021002723955], [-0.017301088723121957, 0.02996921372312196], [-0.017184445791507758, 0.02975694579150776], [-0.016972606362708413, 0.02950760636270841], [-0.016718333958153996, 0.029267083958153993], [-0.016453702731043345, 0.029063702731043348], [-0.01622278542622513, 0.02892653542622513], [-0.016039195797577604, 0.0288491957975776], [-0.015894771299909325, 0.028811646299909326], [-0.01577220959406654, 0.028777209594066544], [-0.01564259436468614, 0.02870009436468614], [-0.015494096729141833, 0.028542221729141833], [-0.015321928201526034, 0.028284428201526036], [-0.015141591474046014, 0.02792971647404601], [-0.014983124917233161, 0.02749312491723316], [-0.014893165186189192, 0.02701629018618919], [-0.014893308885125746, 0.026525183885125744], [-0.015005350269383131, 0.02604097526938313], [-0.015205165461357516, 0.025566415461357517], [-0.015477730464552282, 0.02509273046455228], [-0.015764327038068543, 0.024587452038068544], [-0.016003453462392347, 0.024025953462392342], [-0.0161680499732053, 0.023403674973205304], [-0.01621968566188263, 0.022723435661882625], [-0.016162490014402835, 0.022017490014402834], [-0.016023158030890963, 0.021341283030890962], [-0.015845786646416803, 0.0207589116464168], [-0.01569263328551246, 0.020348258285512455], [-0.015594981746192402, 0.020153106746192403], [-0.015595432431927446, 0.020205432431927448], [-0.015695162776436462, 0.020497037776436466], [-0.015877475589894337, 0.020992475589894338], [-0.016093826101258365, 0.021620701101258365], [-0.01631060911633407, 0.022312484116334068], [-0.016498137966657136, 0.023011262966657137], [-0.016627616598061314, 0.023655116598061313], [-0.016696522350849653, 0.024217772350849653], [-0.01671467100159018, 0.02467779600159018], [-0.016681901608578686, 0.025032526608578687], [-0.016614765518784203, 0.025276015518784205], [-0.01652684358098709, 0.025427468580987093], [-0.01642030501358744, 0.025491555013587443], [-0.0162960274498826, 0.025471027449882597], [-0.016144268531277102, 0.025376768531277107], [-0.015969679247991888, 0.025225304247991884], [-0.01575823179357756, 0.02503135679357756], [-0.015509353828164756, 0.024804353828164753], [-0.0152479232181379, 0.0245941732181379], [-0.014959212742292349, 0.02439858774229235], [-0.014675974088108168, 0.024268474088108165], [-0.014415899445869455, 0.024224024445869456], [-0.014179583689370914, 0.02428145868937091], [-0.01399063771621728, 0.024453762716217284], [-0.013836594108523368, 0.02473034410852337], [-0.013719088564294097, 0.025093463564294094], [-0.013625344990931543, 0.025517219990931546], [-0.013538554901586706, 0.025968554901586702], [-0.013455770047825114, 0.02642077004782511], [-0.013366275141652107, 0.026835650141652106], [-0.013288391555980564, 0.027209641555980567], [-0.01322850562563373, 0.027526630625633725], [-0.01321332237072971, 0.027797697370729714], [-0.013263388823922789, 0.028027138823922788], [-0.013418119014989315, 0.028254994014989314], [-0.013687281107159499, 0.0284885311071595], [-0.014081995398199633, 0.028754495398199638], [-0.014569917353290399, 0.0290361673532904], [-0.015141302752835232, 0.02934317775283523], [-0.015733081257448427, 0.02963620625744843], [-0.016290725114489785, 0.02988697511448979], [-0.016758548684960826, 0.03006229868496083]], 'D403': [[-0.017095228781793037, 0.030134603781793036], [-0.017275271002723955, 0.030104021002723955], [-0.017301088723121957, 0.02996921372312196], [-0.017184445791507758, 0.02975694579150776], [-0.016972606362708413, 0.02950760636270841], [-0.016718333958153996, 0.029267083958153993], [-0.016453702731043345, 0.029063702731043348], [-0.01622278542622513, 0.02892653542622513], [-0.016039195797577604, 0.0288491957975776], [-0.015894771299909325, 0.028811646299909326], [-0.01577220959406654, 0.028777209594066544], [-0.01564259436468614, 0.02870009436468614], [-0.015494096729141833, 0.028542221729141833], [-0.015321928201526034, 0.028284428201526036], [-0.015141591474046014, 0.02792971647404601], [-0.014983124917233161, 0.02749312491723316], [-0.014893165186189192, 0.02701629018618919], [-0.014893308885125746, 0.026525183885125744], [-0.015005350269383131, 0.02604097526938313], [-0.015205165461357516, 0.025566415461357517], [-0.015477730464552282, 0.02509273046455228], [-0.015764327038068543, 0.024587452038068544], [-0.016003453462392347, 0.024025953462392342], [-0.0161680499732053, 0.023403674973205304], [-0.01621968566188263, 0.022723435661882625], [-0.016162490014402835, 0.022017490014402834], [-0.016023158030890963, 0.021341283030890962], [-0.015845786646416803, 0.0207589116464168], [-0.01569263328551246, 0.020348258285512455], [-0.015594981746192402, 0.020153106746192403], [-0.015595432431927446, 0.020205432431927448], [-0.015695162776436462, 0.020497037776436466], [-0.015877475589894337, 0.020992475589894338], [-0.016093826101258365, 0.021620701101258365], [-0.01631060911633407, 0.022312484116334068], [-0.016498137966657136, 0.023011262966657137], [-0.016627616598061314, 0.023655116598061313], [-0.016696522350849653, 0.024217772350849653], [-0.01671467100159018, 0.02467779600159018], [-0.016681901608578686, 0.025032526608578687], [-0.016614765518784203, 0.025276015518784205], [-0.01652684358098709, 0.025427468580987093], [-0.01642030501358744, 0.025491555013587443], [-0.0162960274498826, 0.025471027449882597], [-0.016144268531277102, 0.025376768531277107], [-0.015969679247991888, 0.025225304247991884], [-0.01575823179357756, 0.02503135679357756], [-0.015509353828164756, 0.024804353828164753], [-0.0152479232181379, 0.0245941732181379], [-0.014959212742292349, 0.02439858774229235], [-0.014675974088108168, 0.024268474088108165], [-0.014415899445869455, 0.024224024445869456], [-0.014179583689370914, 0.02428145868937091], [-0.01399063771621728, 0.024453762716217284], [-0.013836594108523368, 0.02473034410852337], [-0.013719088564294097, 0.025093463564294094], [-0.013625344990931543, 0.025517219990931546], [-0.013538554901586706, 0.025968554901586702], [-0.013455770047825114, 0.02642077004782511], [-0.013366275141652107, 0.026835650141652106], [-0.013288391555980564, 0.027209641555980567], [-0.01322850562563373, 0.027526630625633725], [-0.01321332237072971, 0.027797697370729714], [-0.013263388823922789, 0.028027138823922788], [-0.013418119014989315, 0.028254994014989314], [-0.013687281107159499, 0.0284885311071595], [-0.014081995398199633, 0.028754495398199638], [-0.014569917353290399, 0.0290361673532904], [-0.015141302752835232, 0.02934317775283523], [-0.015733081257448427, 0.02963620625744843], [-0.016290725114489785, 0.02988697511448979], [-0.016758548684960826, 0.03006229868496083]]}}
```

In [3]:

```
data_df = pd.DataFrame.from_dict(data)
data_df
```

Out[3]:

|  | chl\_id | loc\_res\_Mg | scan\_amp\_C2 | scan\_amp\_C7 | scan\_angles | ci |
| --- | --- | --- | --- | --- | --- | --- |
| A405 | A405 | 2.18 | [0.01096, 0.01136, 0.01185, 0.0124, 0.01301, 0... | [0.0101, 0.00925, 0.00841, 0.0076, 0.00682, 0.... | [0.0, 5.0, 10.0, 15.0, 20.0, 25.0, 30.0, 35.0,... | [[-0.017095228781793037, 0.030134603781793036]... |
| A407 | A407 | 2.18 | [0.01362, 0.01394, 0.01404, 0.01393, 0.01359, ... | [0.00427, 0.00445, 0.00483, 0.00537, 0.00606, ... | [0.0, 5.0, 10.0, 15.0, 20.0, 25.0, 30.0, 35.0,... | [[-0.017095228781793037, 0.030134603781793036]... |
| A410 | A410 | 2.36 | [0.01437, 0.01299, 0.0117, 0.01052, 0.00946, 0... | [-0.00086, -0.00038, 7e-05, 0.00049, 0.00091, ... | [0.0, 5.0, 10.0, 15.0, 20.0, 25.0, 30.0, 35.0,... | [[-0.017095228781793037, 0.030134603781793036]... |
| B603 | B603 | 2.37 | [0.00214, 0.00039, -0.00138, -0.00312, -0.0047... | [0.01075, 0.00985, 0.00901, 0.00828, 0.00771, ... | [0.0, 5.0, 10.0, 15.0, 20.0, 25.0, 30.0, 35.0,... | [[-0.017095228781793037, 0.030134603781793036]... |
| B604 | B604 | 2.29 | [0.00302, 0.00199, 0.00105, 0.0002, -0.00056, ... | [0.00504, 0.00596, 0.00687, 0.00773, 0.00852, ... | [0.0, 5.0, 10.0, 15.0, 20.0, 25.0, 30.0, 35.0,... | [[-0.017095228781793037, 0.030134603781793036]... |
| B605 | B605 | 2.25 | [0.0173, 0.01517, 0.01321, 0.01146, 0.00994, 0... | [0.01748, 0.01751, 0.01742, 0.01721, 0.0169, 0... | [0.0, 5.0, 10.0, 15.0, 20.0, 25.0, 30.0, 35.0,... | [[-0.017095228781793037, 0.030134603781793036]... |
| B606 | B606 | 2.29 | [0.01064, 0.01021, 0.00963, 0.0089, 0.00805, 0... | [0.00035, 0.00022, 0.00022, 0.00033, 0.00052, ... | [0.0, 5.0, 10.0, 15.0, 20.0, 25.0, 30.0, 35.0,... | [[-0.017095228781793037, 0.030134603781793036]... |
| B607 | B607 | 2.39 | [0.00714, 0.0085, 0.00991, 0.01131, 0.01261, 0... | [0.01662, 0.01572, 0.01471, 0.01359, 0.01239, ... | [0.0, 5.0, 10.0, 15.0, 20.0, 25.0, 30.0, 35.0,... | [[-0.017095228781793037, 0.030134603781793036]... |
| B608 | B608 | 2.26 | [0.03414, 0.03551, 0.03655, 0.0372, 0.03746, 0... | [0.00953, 0.00946, 0.00944, 0.00947, 0.0095, 0... | [0.0, 5.0, 10.0, 15.0, 20.0, 25.0, 30.0, 35.0,... | [[-0.017095228781793037, 0.030134603781793036]... |
| B609 | B609 | 2.30 | [0.01031, 0.00975, 0.00918, 0.00861, 0.00804, ... | [0.01116, 0.01191, 0.01246, 0.0128, 0.01289, 0... | [0.0, 5.0, 10.0, 15.0, 20.0, 25.0, 30.0, 35.0,... | [[-0.017095228781793037, 0.030134603781793036]... |
| B610 | B610 | 2.52 | [0.01314, 0.01235, 0.01144, 0.01047, 0.00948, ... | [-0.00747, -0.00776, -0.00826, -0.00893, -0.00... | [0.0, 5.0, 10.0, 15.0, 20.0, 25.0, 30.0, 35.0,... | [[-0.017095228781793037, 0.030134603781793036]... |
| B611 | B611 | 2.37 | [0.00119, 0.00142, 0.00183, 0.00241, 0.00311, ... | [0.00325, 0.00318, 0.00311, 0.00303, 0.00291, ... | [0.0, 5.0, 10.0, 15.0, 20.0, 25.0, 30.0, 35.0,... | [[-0.017095228781793037, 0.030134603781793036]... |
| B612 | B612 | 2.33 | [0.00985, 0.00932, 0.00859, 0.00773, 0.0068, 0... | [0.01135, 0.01159, 0.01187, 0.01217, 0.01247, ... | [0.0, 5.0, 10.0, 15.0, 20.0, 25.0, 30.0, 35.0,... | [[-0.017095228781793037, 0.030134603781793036]... |
| B613 | B613 | 2.36 | [-0.00771, -0.00874, -0.00963, -0.01037, -0.01... | [0.00577, 0.00639, 0.00709, 0.00786, 0.00865, ... | [0.0, 5.0, 10.0, 15.0, 20.0, 25.0, 30.0, 35.0,... | [[-0.017095228781793037, 0.030134603781793036]... |
| B614 | B614 | 2.31 | [0.03265, 0.03387, 0.03504, 0.03617, 0.03723, ... | [0.02189, 0.02118, 0.02036, 0.01944, 0.01844, ... | [0.0, 5.0, 10.0, 15.0, 20.0, 25.0, 30.0, 35.0,... | [[-0.017095228781793037, 0.030134603781793036]... |
| B616 | B616 | 2.49 | [0.01137, 0.01188, 0.01238, 0.01283, 0.01321, ... | [0.00653, 0.00669, 0.00689, 0.00711, 0.0073, 0... | [0.0, 5.0, 10.0, 15.0, 20.0, 25.0, 30.0, 35.0,... | [[-0.017095228781793037, 0.030134603781793036]... |
| B617 | B617 | 2.59 | [-0.0024, -0.0022, -0.00183, -0.00131, -0.0006... | [-0.00123, -0.0023, -0.00303, -0.00338, -0.003... | [0.0, 5.0, 10.0, 15.0, 20.0, 25.0, 30.0, 35.0,... | [[-0.017095228781793037, 0.030134603781793036]... |
| C501 | C501 | 2.42 | [0.00788, 0.00695, 0.00612, 0.00542, 0.00486, ... | [0.01483, 0.01423, 0.01359, 0.01296, 0.01238, ... | [0.0, 5.0, 10.0, 15.0, 20.0, 25.0, 30.0, 35.0,... | [[-0.017095228781793037, 0.030134603781793036]... |
| C502 | C502 | 2.34 | [0.01259, 0.01218, 0.01164, 0.011, 0.0103, 0.0... | [0.0007, 0.00081, 0.00101, 0.00132, 0.00169, 0... | [0.0, 5.0, 10.0, 15.0, 20.0, 25.0, 30.0, 35.0,... | [[-0.017095228781793037, 0.030134603781793036]... |
| C503 | C503 | 2.46 | [0.00717, 0.00729, 0.00746, 0.00767, 0.00789, ... | [-0.00259, -0.00401, -0.00527, -0.00636, -0.00... | [0.0, 5.0, 10.0, 15.0, 20.0, 25.0, 30.0, 35.0,... | [[-0.017095228781793037, 0.030134603781793036]... |
| C504 | C504 | 2.38 | [0.00041, 0.0003, 0.00035, 0.00055, 0.00087, 0... | [-0.00826, -0.00767, -0.0067, -0.00537, -0.003... | [0.0, 5.0, 10.0, 15.0, 20.0, 25.0, 30.0, 35.0,... | [[-0.017095228781793037, 0.030134603781793036]... |
| C505 | C505 | 2.34 | [0.01056, 0.00985, 0.009, 0.008, 0.00689, 0.00... | [0.00412, 0.00438, 0.00463, 0.00486, 0.0051, 0... | [0.0, 5.0, 10.0, 15.0, 20.0, 25.0, 30.0, 35.0,... | [[-0.017095228781793037, 0.030134603781793036]... |
| C506 | C506 | 2.50 | [0.00927, 0.00915, 0.00914, 0.00924, 0.00945, ... | [0.01941, 0.0204, 0.02133, 0.02221, 0.02301, 0... | [0.0, 5.0, 10.0, 15.0, 20.0, 25.0, 30.0, 35.0,... | [[-0.017095228781793037, 0.030134603781793036]... |
| C507 | C507 | 2.40 | [0.00989, 0.00999, 0.01015, 0.01035, 0.01059, ... | [0.00862, 0.00794, 0.00734, 0.00685, 0.00651, ... | [0.0, 5.0, 10.0, 15.0, 20.0, 25.0, 30.0, 35.0,... | [[-0.017095228781793037, 0.030134603781793036]... |
| C508 | C508 | 2.34 | [0.0034, 0.00376, 0.0042, 0.00472, 0.00533, 0.... | [-0.00443, -0.00439, -0.00415, -0.0037, -0.003... | [0.0, 5.0, 10.0, 15.0, 20.0, 25.0, 30.0, 35.0,... | [[-0.017095228781793037, 0.030134603781793036]... |
| C509 | C509 | 2.38 | [0.00092, -0.00064, -0.00203, -0.00325, -0.004... | [0.00443, 0.00524, 0.00616, 0.00718, 0.00827, ... | [0.0, 5.0, 10.0, 15.0, 20.0, 25.0, 30.0, 35.0,... | [[-0.017095228781793037, 0.030134603781793036]... |
| C510 | C510 | 2.37 | [-0.00302, -0.00303, -0.00288, -0.00255, -0.00... | [0.02062, 0.02036, 0.0196, 0.01838, 0.01675, 0... | [0.0, 5.0, 10.0, 15.0, 20.0, 25.0, 30.0, 35.0,... | [[-0.017095228781793037, 0.030134603781793036]... |
| C511 | C511 | 2.47 | [0.01257, 0.01149, 0.01025, 0.00888, 0.00744, ... | [-0.00336, -0.00385, -0.00415, -0.00426, -0.00... | [0.0, 5.0, 10.0, 15.0, 20.0, 25.0, 30.0, 35.0,... | [[-0.017095228781793037, 0.030134603781793036]... |
| C512 | C512 | 2.49 | [0.0096, 0.01009, 0.01057, 0.011, 0.01137, 0.0... | [0.00153, 0.00015, -0.00115, -0.00231, -0.0032... | [0.0, 5.0, 10.0, 15.0, 20.0, 25.0, 30.0, 35.0,... | [[-0.017095228781793037, 0.030134603781793036]... |
| C513 | C513 | 2.64 | [0.01438, 0.0144, 0.01435, 0.01421, 0.01399, 0... | [0.00585, 0.00658, 0.00737, 0.0082, 0.00904, 0... | [0.0, 5.0, 10.0, 15.0, 20.0, 25.0, 30.0, 35.0,... | [[-0.017095228781793037, 0.030134603781793036]... |
| D402 | D402 | 2.17 | [0.00833, 0.00855, 0.00887, 0.00924, 0.00962, ... | [0.01168, 0.01233, 0.0129, 0.01344, 0.01398, 0... | [0.0, 5.0, 10.0, 15.0, 20.0, 25.0, 30.0, 35.0,... | [[-0.017095228781793037, 0.030134603781793036]... |
| D403 | D403 | 2.35 | [0.00734, 0.00705, 0.00668, 0.00626, 0.00582, ... | [0.01095, 0.00984, 0.00871, 0.00759, 0.00651, ... | [0.0, 5.0, 10.0, 15.0, 20.0, 25.0, 30.0, 35.0,... | [[-0.017095228781793037, 0.030134603781793036]... |

#### Chlorophyll Resolution¶

The ESP map local resolution at the central Mg of each chlorophyll is shown in the graph below.

In [4]:

```
data_df.sort_values(by = ['loc_res_Mg'], inplace = True)
res_Mg = np.array(data_df['loc_res_Mg'].to_list())
x = np.arange(len(res_Mg))

fig, ax = plt.subplots(figsize = (6, 4))
ax.plot(x, res_Mg, c = 'tab:blue', ls = 'none', marker = 'o', ms = 4)
ax.set_xlabel('chlorophyll index')
ax.set_ylabel('Resolution ($\AA$)')
ax.set_title('Chlorophyll Resolutions', fontweight = 'bold')
```

Out[4]:

```
Text(0.5, 1.0, 'Chlorophyll Resolutions')
```

#### Cone Scans for all chlorophyll¶

The C2 cone scans for each chlorophyll compared to the null distribution is shown below.

In [5]:

```
fig, axs = plt.subplots(8, 4, figsize=(6.5*2, 9*2), sharex = True, sharey = True)
plt.setp(fig, tight_layout = True)


all_chl_ids = data_df.index.to_list()
for chl_id, ax in zip(all_chl_ids, axs.flat):
    # data
    row = data_df.loc[chl_id]
    scan_angles = np.array(row['scan_angles'])
    scan_amp_C2 = np.array(row['scan_amp_C2'])
    scan_amp_C7 = np.array(row['scan_amp_C7'])
    ci = np.array(row['ci'])

    # Plotting
    l_c2 = ax.plot(scan_angles, scan_amp_C2*10)
    ci_fill = ax.fill_between(scan_angles, *ci.T*10)
    h_l = ax.axhline()

    # Making things pretty
    plt.setp(l_c2, label = 'C2', ls = '-', color = "tab:blue")
    plt.setp(ci_fill, label = 'ci', color = 'tab:orange', alpha = 0.2)
    plt.setp(h_l, color = 'black', ls = '--')
    plt.setp(ax, xlim = [0, 355])
    subplot_title = f"{chl_id} | {str(row['loc_res_Mg'])[:4]} $\AA$"
    ax.set_title(subplot_title)
    plt.setp(ax, ylabel = 'ESP', xlabel = 'Scan Angle ($^\circ$)')
    ax.label_outer()
    
# custom shared legend
ax_label = fig.add_subplot(111) # label subplot
ax_label.axis('off')
fill = mpatches.Patch(color = 'tab:orange', alpha = 0.2, label = 'Null Distribution')
line = mlines.Line2D([], [], color = 'tab:blue', label = 'C2 Cone Scan')
ax_label.legend(handles = [line, fill], prop = {"size":15}, ncol = 2, loc = 'upper center', bbox_to_anchor = (0.5, 1.05))
```

Out[5]:

```
<matplotlib.legend.Legend at 0x7f64a3d93898>
```
